# Stable in Four Oxidation States: Exploring the Redox-Variability of Molybdenum and Tungsten Triazolylidene Complexes

**DOI:** 10.1021/acs.inorgchem.6c00876

**Published:** 2026-05-28

**Authors:** Florian R. Neururer, Florian Heim, Lena Gschnell, Daniel Leitner, Michael Seidl, Alexander Pöthig, Stephan Hohloch

**Affiliations:** † 151267 Leopold-Franzens-University Innsbruck, Faculty of Chemistry and Pharmacy, Institute of General, Inorganic and Theoretical Chemistry, Innrain 80-82, 6020 Innsbruck, Austria; ‡ Catalysis Research Center (CRC) & TUM School of Natural Sciences, Department of Chemistry, Technical University of Munich, Ernst-Otto-Fischer Str. 1, 85747 Garching, Germany

## Abstract

The stability and (redox-)­chemistry of molybdenum and tungsten triazolylidene complexes in four oxidation states is examined, starting from the oxo complexes L^1^MO_2_ and L^1^ MoO­(N^*t*^Bu) (**1-M**, M = Mo, W and **2-Mo**, L^1^= bisphenolate triazolylidene). Deoxygenative chlorination using either TMS-Cl or thionyl chloride gave access to the dichloro complexes L^1^MOCl_2_ **3-M** (M = Mo, W) and L^1^ M­(NtBu)­Cl_2_ (**4-Mo**). The oxo-chlorido complexes **3-M** can be deoxygenated or reduced, giving access to a plethora of halide complexes. Reduction with potassium graphite yielded the Mo­(V) complexes L^1^ MoOCl **6a-Mo**, which can be further chlorinated using thionyl chloride to trichloro complex **8-Mo**. Deoxygenation of the M­(VI) complexes **3-M** using trimethylphosphine forms the neutral M­(IV) complexes L^1^MCl_2_(PMe_3_) **9-M**. Alternatively, starting from **3-M** cationic M­(IV) complexes [L^1^MCl­(PMe_3_)_2_]­[BArF_24_] **11-M** are accessible, by halide abstraction followed by deoxygenation. Cyclic voltammetry suggests a rich redox chemistry of these cations and L^1^MCl­(PMe_3_)_2_ **12-M** can be obtained after one electron reduction. During all transformations, the M_carbene_ bond stays intact, proving that MIC ligands are valuable ligands for stabilizing tungsten and molybdenum complexes in a large variety of oxidation states with potential applications in catalysis and small molecule activation.

## Graphical abstract

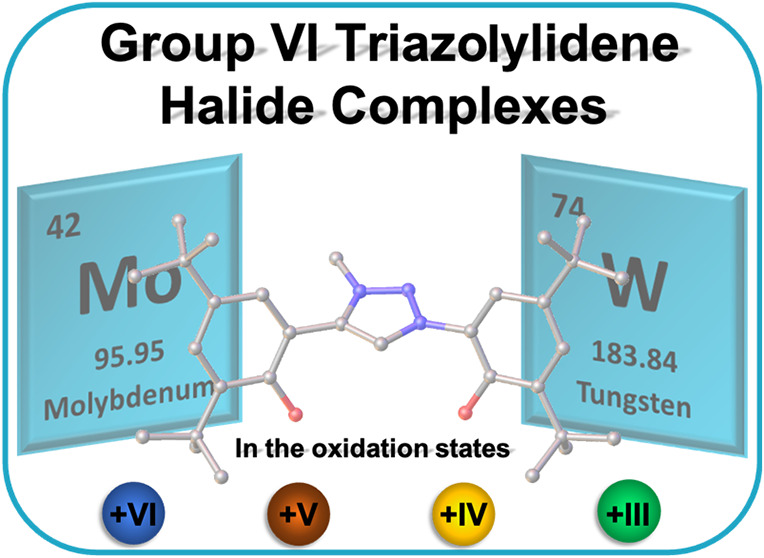

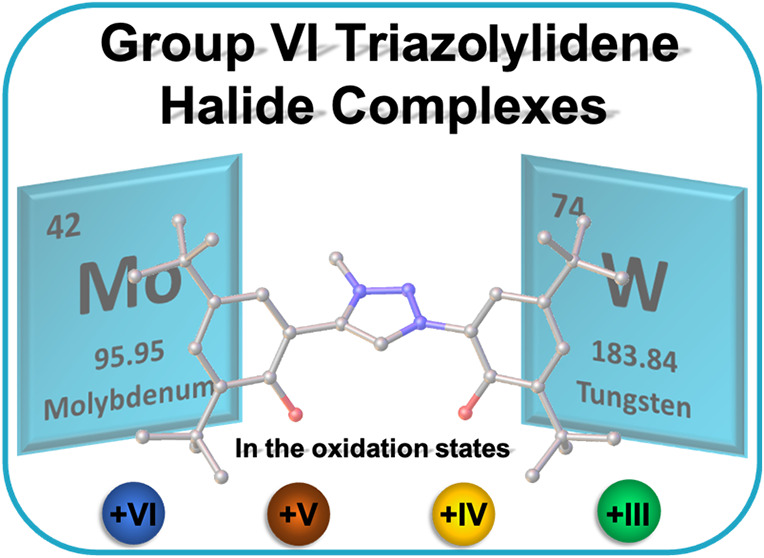

## Introduction

In the past two decades, mesoionic carbenes (MICs) have developed into a versatile ligand class in (organometallic) chemistry.^1–10^ “Typical” imidazole-4-ylidene based MICs have first been isolated in a complex by Crabtree in 2001 (named abnormal carbenes at that time),^11^ while Bertrand and co-workers have isolated them in the free form a few years later.^12^ Since then, imidazole-4-ylidene type ligands^13^ have found applications in transition metal chemistry,^14,15^ main group chemistry,^16–21^ in the stabilization of (bi)­radical species^20–25^ and in catalysis.^26,27^ However, contrasting NHC chemistry,^28,29^ the vast success of MICs is not attributed to imidazol-based but to 1,2,3-triazol-based carbenes.^3–5,30^ First reported by Albrecht and co-workers^31^ in 2008 and isolated in the free form by Bertrand in 2010,^32^ 1,2,3-triazolylidenes have found widespread use in organometallic chemistry,^33–37^ (electro- and organo-)­catalysis,^38–56^ supramolecular chemistry,^57,58^ magnetism^59^ and photochemistry.^60–68^ This success is closely related to their modular synthesis using a cycloaddition between alkynes and azides,^69,70^ to give selective access to 1,4- ^71,72^ or 1,5-distubstituted^73–75^ triazoles respectively, which can be further alkylated or arylated to the corresponding triazolium salts,^31,45^ which are acting as direct precursors to triazolylidene-based mesoionic carbenes. Alternatively, triazolium salts can also be accessed using a cycloaddition reaction between *in situ* generated *N*-chlorotriazines and alkynes.^76,77^ These synthetic strategies allow the synthesis of a plethora of differently substituted triazolylidene ligands giving access to mono-,^31,43,78–80^ bi-,^59,81–83^ tridentate^61,84–86^ or tripodal^87^ ligand frameworks. Additionally, the modular synthesis also allows the isolation of anionic triazolylidenes, which can be accessed using two different strategies: The addition of a borane^59,88^ in the triazole-backbone or the installation of anionic linkers (e.g., carbazoles^85,86,89,90^ or phenolates^84,91^ ). Following the latter strategy, we recently synthesized the dianionic bis-phenolate triazolylidene ligand **L** ^**1**^ (Figure 1, top),^84^ and studied its application in early transition metal chemistry;^92,93^ a region of the periodic table, where NHC chemistry in general is still underrepresented.^94^ Targeting this gap, we recently synthesized a variety of group IV^84^ and group V^92,93,95^ complexes coordinated by this and related bis-phenolate ligands, while Albrecht and co-workers utilized this ligand to synthesize Mn­(III) complexes for oxidation catalysis.^96^ Focusing on group VI, molybdenum and tungsten in particular, we recently reported the application of dioxomolybdenum complex **1** in the catalytic deoxygenation of nitroarenes,^97^ revealing an influence of the triazolylidene donor on the catalytic potential of the complexes surpassing (benz-)imidazolylidene analogues **A** and **B**.^98^ Additionally, we recently reported the first molybdenum triazolylidene nitrido complex **C**.^99^ A similar nitride complex based on the 2-imidazolylidene framework was also recently studied in the context of reductive nitrogen silylation.^100^ Reduction of **C** under protic conditions cleanly forms the dimeric Mo­(III) complex **D**.^99^ Both studies suggest a strong molybdenum-triazolylidene bond under protic and/or reductive conditions. This stability under protic conditions was further demonstrated by Royo et al. reporting the utility of Mo(0) triazolylidene complex **E** (Figure 1) in the acceptorless dehydrogenation of alcohols.^101^ A similar Mo(0) triazolylidene complex **G** was also reported by Schulzke et al.^102^ The tungsten analogue **F** was also used in alcohol dehydrogenation and displayed unique cooperative effects between the ligand backbone and the tungsten center in this reaction.^103^ Notably, related pyridyl-triazolylidene Mo(0) complexes (and group VI triazolylidene complexes in general) have been found to be good NIR emitters (complex **H**, Figure 1).^66^ Finally Buchmeiser et al. reported triazolylidene complex **I** (Figure 1) for the polymerization and thermal curing of *endo*-DCPD (dicyclopentadiene).^104^ However, to the best of our knowledge, only limited studies on the redox stability and variability of triazolylidene molybdenum and tungsten complexes have been reported so far. Given the shown stability, and huge application potential of triazolylidene ligands in group VI chemistry,^62,66–68,94,97,99,101,102,104–106^ we were further interested in the scope of transformations that the triazolylidene moiety can facilitate/tolerate in group VI chemistry, with special emphasis on different oxidation states of molybdenum and tungsten.

**Figure 1:**
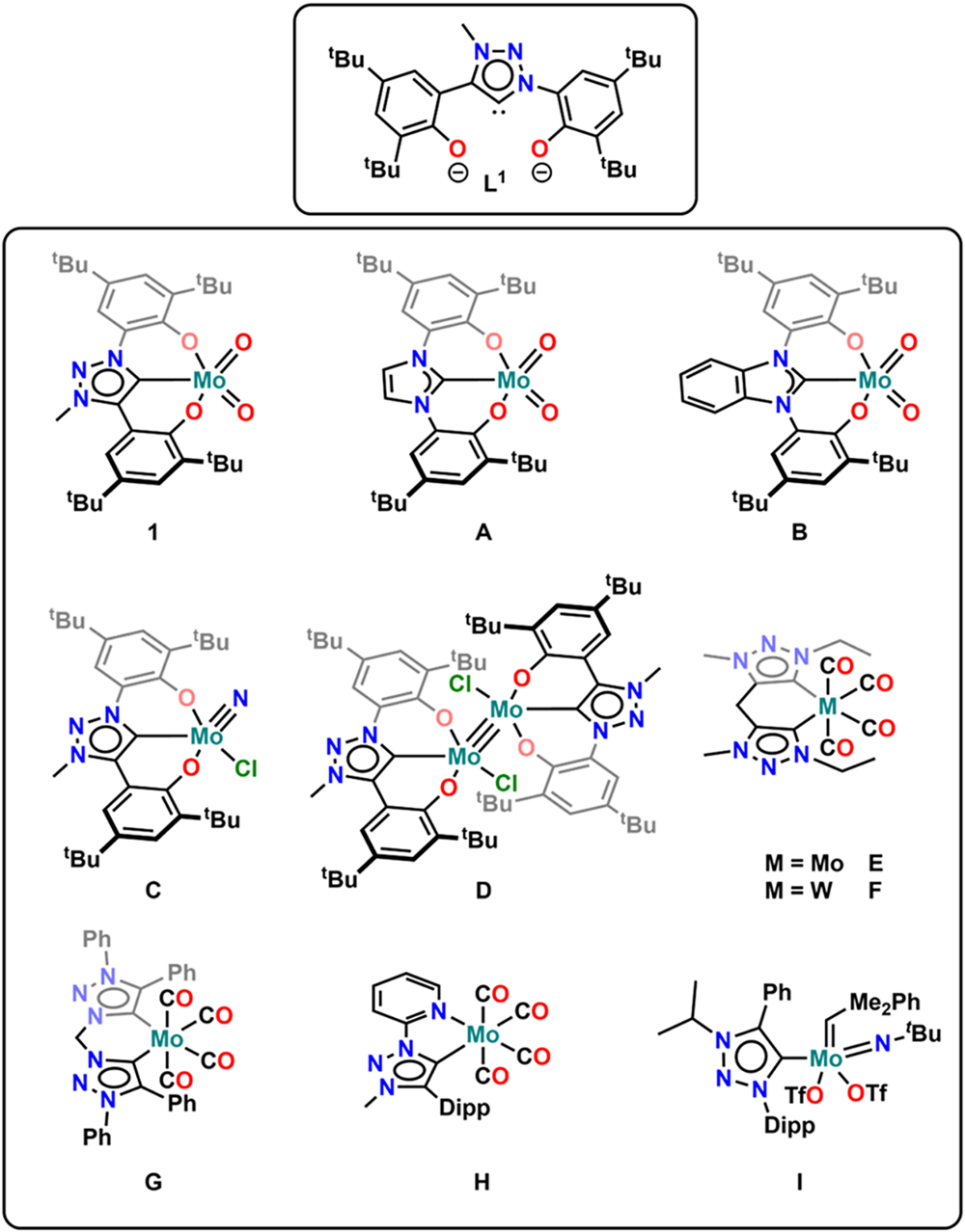
Ligand **L** ^**1**^ used in this study (top) and selected molybdenum complexes used in deoxygenation catalysis (**1, B**, **C**) and stoichiometric ammonia evolution (**D**, **E**) recently reported by our group.

## Results and Discussion

Starting from the dioxo complexes **1-Mo** and **1-W**, we were interested in their reactivity beyond reductive deoxygenation catalysis.^97^ We anticipated that the substitution of an oxo moiety with halides would afford interesting precursors for follow-up chemistry. Initially, this was attempted by the addition of chlorotrimethylsilane (TMS-Cl) to a toluene solution of **1-Mo** (Scheme 1). This was accompanied by a color change from bright yellow to dark red to dark green within 2 days. The dichlorido complex **3-Mo** could be isolated in almost quantitative yields. Given the smooth reaction, we were interested whether this reaction could be adapted to other oxomolybdenum complexes. Applying similar conditions to the oxo-imido complex **2-Mo**, which can be prepared from the reaction between **[H**_**3**_ **L** ^**1**^ **]­[Cl]** ^84^ and (DME)­Mo­(N^*t*^Bu)­OCl_2_ ^107^ using triethylamine as a base, gives clean access to the dichlorido complex **4-Mo** as a dark blue solid. Formation of the dichlorido complexes is indicated by the redistribution of the aromatic protons and the shift of the *N*-CH_3_ protons of the triazolylidene ligand from 3.24 ^97^/3.14 ppm (Figure S1) in **1-Mo**/**2-Mo** to 2.90/3.05 ppm in **3-Mo**/**4-Mo** respectively (Figures S11/S21). In contrast, **1-W** could not be fully deoxygenated using TMS-chloride. Instead, the mono chloride complex **3-W**′ was isolated. Formation of a mixed chloride siloxide complex **3-W**′ is evident by ^1^H NMR spectroscopy. The spectrum shows an intense signal at δ 0.68 ppm, which is indicative to the presence of an O-TMS group (Figure S6). Unambiguous proof for the formation of **3-W**′ is given by X-ray diffraction analysis of single crystals grown by vapor diffusion of hexane into a concentrated toluene solution (Figure 2). The tungsten atom in the complex is hexacoordinated by the OCO atoms of the triazolylidene ligand, a terminal oxo group, the siloxide ligand and the chloride ion. The W1–O10 distance is 1.691(3) Å, comparable to the parent dioxo complex^97^ and the O-TMS ligand is orientated *trans* to the triazolylidene carbon atom. The W1–C1 distance is found at 2.163(2) Å and is also similar to previously reported triazolylidene tungsten complexes.^66,97^ Heating **3-W**′ in an excess of TMS-chloride does not induce a change to the ^1^H NMR, suggesting that it is not capable to fully deoxygenate/chlorinate the tungsten atom. Thus, we attempted the use of thionyl chloride. Indeed, addition of excess thionyl chloride at low-temperatures gives clean access to **3-W**. Formation of a dichloro complex is indicated by ^1^H NMR spectroscopy, showing a complete redistribution of the aromatic protons as well as a shift of the *N*-CH_3_ triazolylidene resonance from 4.71 ppm in **1-W** to 2.96 ppm in **3-W**. Unambiguous proof for the formation of the dichloro complexes **3-Mo**, **3-W**, and **4-Mo** was given by X-ray diffraction analysis (Figure 2). The complexes were crystallized from C_6_D_6_ (**3-Mo**), THF/pentane (**3-W**) and C_6_D_6_/hexane (**4-Mo**) and show hexacoordinated central ions, being coordinated by the OCO donor atoms of the triazolylidene ligand, a terminal oxo/imido ligand and two chloride donors. The M1-O10/M1-N40 distances were found to be 1.680(5), 1.691(8), and 1.711(3) Å for **3-Mo**, **3-W**, and **4-Mo** respectively. For the chloride ligands Cl1 and Cl2, M1-Cl distances of 2.3917(18), 2.347(3) and 2.4073(13) Å were observed for M1–Cl1 for **3-Mo**, **3-W**, and **4-Mo** respectively, while the corresponding M1–Cl2 distances are slightly longer at 2.5061 (19), 2.530(4), 2.5167(13) Å. This is in line with a stronger *trans*-influence of the oxo group, compared to the triazolylidene ligand.

**Figure 2:**
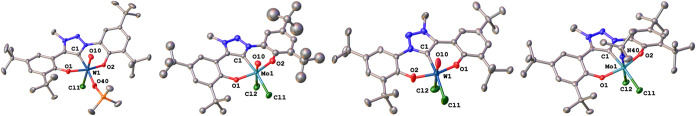
Molecular structures of the halide complexes **3-W′**, **3-Mo**, **3-W** and **4-Mo** (f.l.t.r.). Ellipsoids are shown at a probability level of 50%. Lattice solvent molecules and hydrogen atoms are omitted for clarity.

**Scheme 1:**
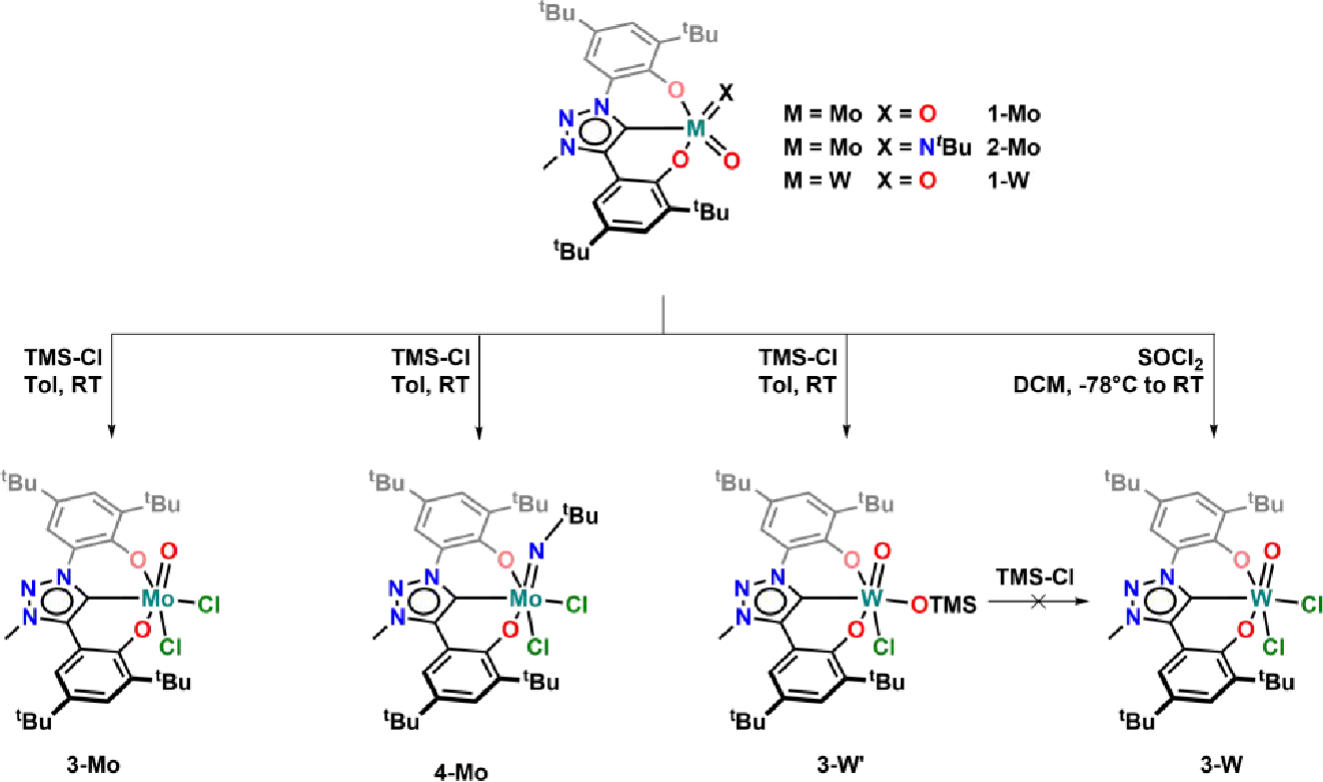
Oxo-Chloro exchange reactions starting from dioxo and oxoimido precursors **1-M** (M = Mo, W) and **2-Mo** using TMS-Cl or SOCl_2_ as deoxygenation-halogenation reagent

With the halide complexes **3-M** (M = Mo, W) in hand, we were further interested in their reactivity. Since **1-Mo** readily reacts with pinacol to form a pinacolate complex, we initially tested catechols. Synthesis of the catecholate complexes is straightforward, by mixing the dihalide complexes **3-M**, triethylamine and 3,5-di-*tert*-butylcatehcol in THF giving access to the catecholate complexes **5-Mo** and **5-W** after workup (Scheme 2). Alternatively, the catecholate complexes can also be accessed *via* protonolysis between the protonated catechol and the dioxo complexes **1-M** in the presence of molecular sieves. Formation of the new catecholate complexes is evident by ^1^H NMR spectroscopy showing a new set of catecholate resonances (Figures S26/S31). The M1-C1 distances at 2.150(5) and 2.154(6) Å for **5-Mo** and **5-W**, respectively are comparable to the halide precursors **3-Mo** and **3-W** (Figure 3). However, the clear assignment of the oxidation state in the catecholate complexes is not trivial, as has been shown by Lehtonen^108^ and Brown.^109–114^

**Figure 3:**
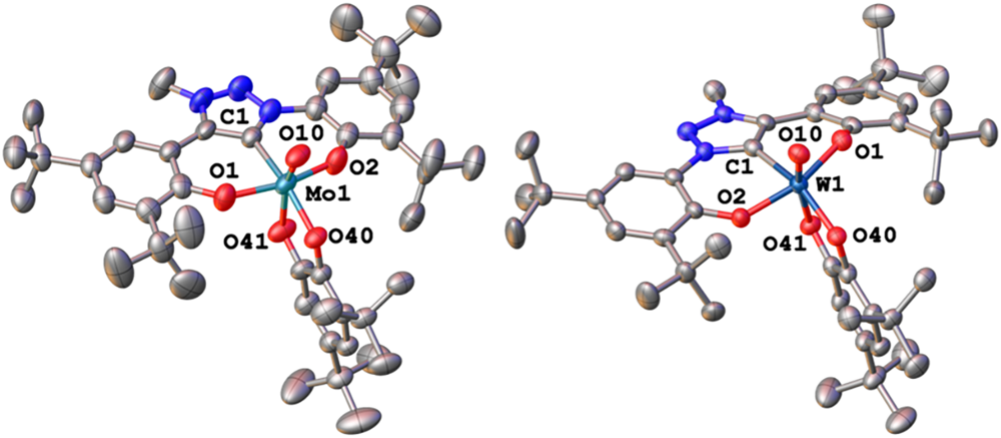
Molecular structures of the catecholate complexes **5-M** (M = Mo, W). Ellipsoids are shown at a probability level of 50%. Lattice solvent molecules and hydrogen atoms are omitted for clarity.

**Scheme 2:**
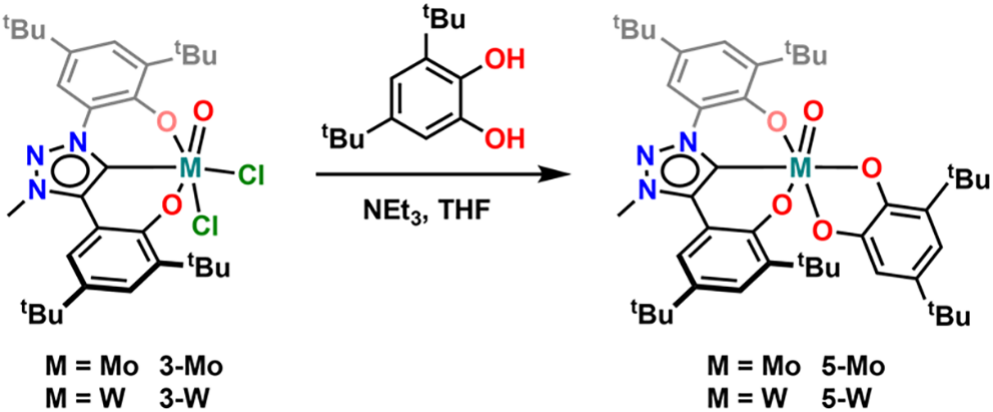
Synthesis of catecholate complexes via salt metathesis from **3-M** (M = Mo, W)

To further investigate the chemistry of “low-valent” M­(V) compounds, we attempted one-electron reduction using KC_8_ starting from **3-Mo** and **3-W** (Scheme 3). While for the molybdenum complex **3-Mo**, this results in the clean reduction to the corresponding Mo­(V) complex **6a-Mo**, starting from the tungsten complex **3-W** gave only access to intractable mixtures from which no clean material could be obtained. Alternatively, **6a-Mo** can be directly synthesized starting from **[H**_**3**_ **L** ^**1**^ **]­[Cl]** and MoOCl_3_ using triethylamine as a base. Similarly, this direct metalation route also gives access to the tungsten complex **6a-W** and the imido complex **6b-Mo** if WOCl_3_(THF)_2_ ^115^ or Mo­(NtBu)­Cl_3_(DME)^116^ is applied as starting material (Scheme 3). All complexes, **6a-Mo, 6a-W** and **6b-Mo** are paramagnetic showing only broad resonances in their ^1^H NMR spectra. They display magnetic moments of 1.49, 1.19, and 1.78 μ_B_ respectively (determined by Evans method, Figure S36–S38), which correspond well to a d^1^ configured M­(V) metal center.^117^ This assumption is supported by EPR measurements (Figure 4, bottom), which display the characteristic pattern for molybdenum and g values of 1.950/1.958 with *a*_iso_(Mo) values of 137/135 MHz for **6a-Mo** and **6b-Mo** respectively. In addition, the EPR signal of **6b-Mo** revealed additional hyperfine coupling with the nitrogen nucleus with *a*_iso_(N) = 9.25 MHz. Likewise, **6a-W** showed the expected tungsten centered signal (Figure S105) with g value of 1.813 and *a*_iso_(W) = 267 MHz.^117^ The molecular structures revealed that upon reduction the Mo–C distances contract from 2.124(6) Å in **3-Mo**/2.122­(4) Å in **4-Mo** to 2.103(4) Å in **6a-Mo** and 2.097(4) Å in **6b-Mo**. This shortening of the Mo–C could be explained by a weak back bonding effect, as it was observed in similar d^1^ configured group VI and group V complexes.^93,95,98,99^ The crystal structure of complex **6b-Mo** showed the weak coordination of an additional diethyl ether molecule to the molybdenum center. However, upon exposure of crystalline samples to vacuo the crystals quickly decompose and analysis of the obtained amorphous material matches the structure depicted in Scheme 3. Unfortunately, for the tungsten complex **6a-W** no crystals suitable for X-ray diffraction analysis could be grown (Scheme 4).

**Figure 4:**
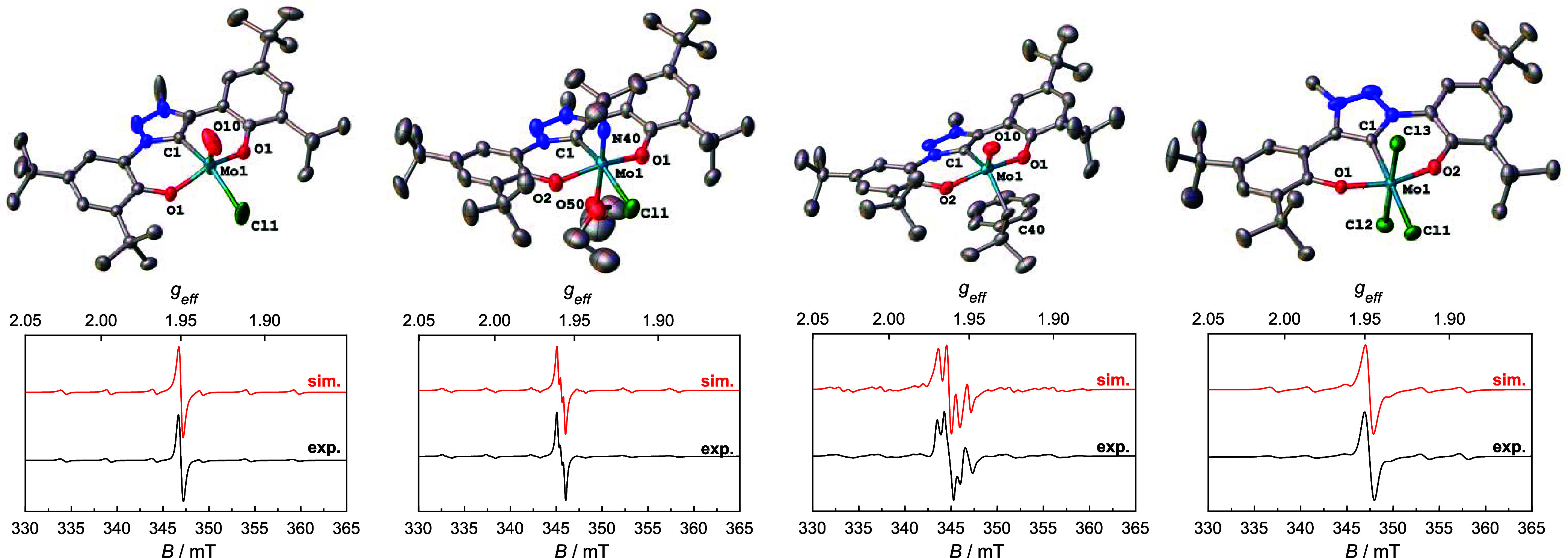
Molecular structures of the complexes **6a-Mo**, **6b-Mo** (as its diethyl ether adduct **6b-Mo**(**OEt**_**2**_)), **7a** and **8-Mo** (top: f.l.t.r) Ellipsoids are shown at a probability level of 50%. Lattice solvent molecules and hydrogen atoms are omitted for clarity. The corresponding X-band EPR spectra (20 °C, dichloromethane solution) are depicted below.

**Scheme 3:**
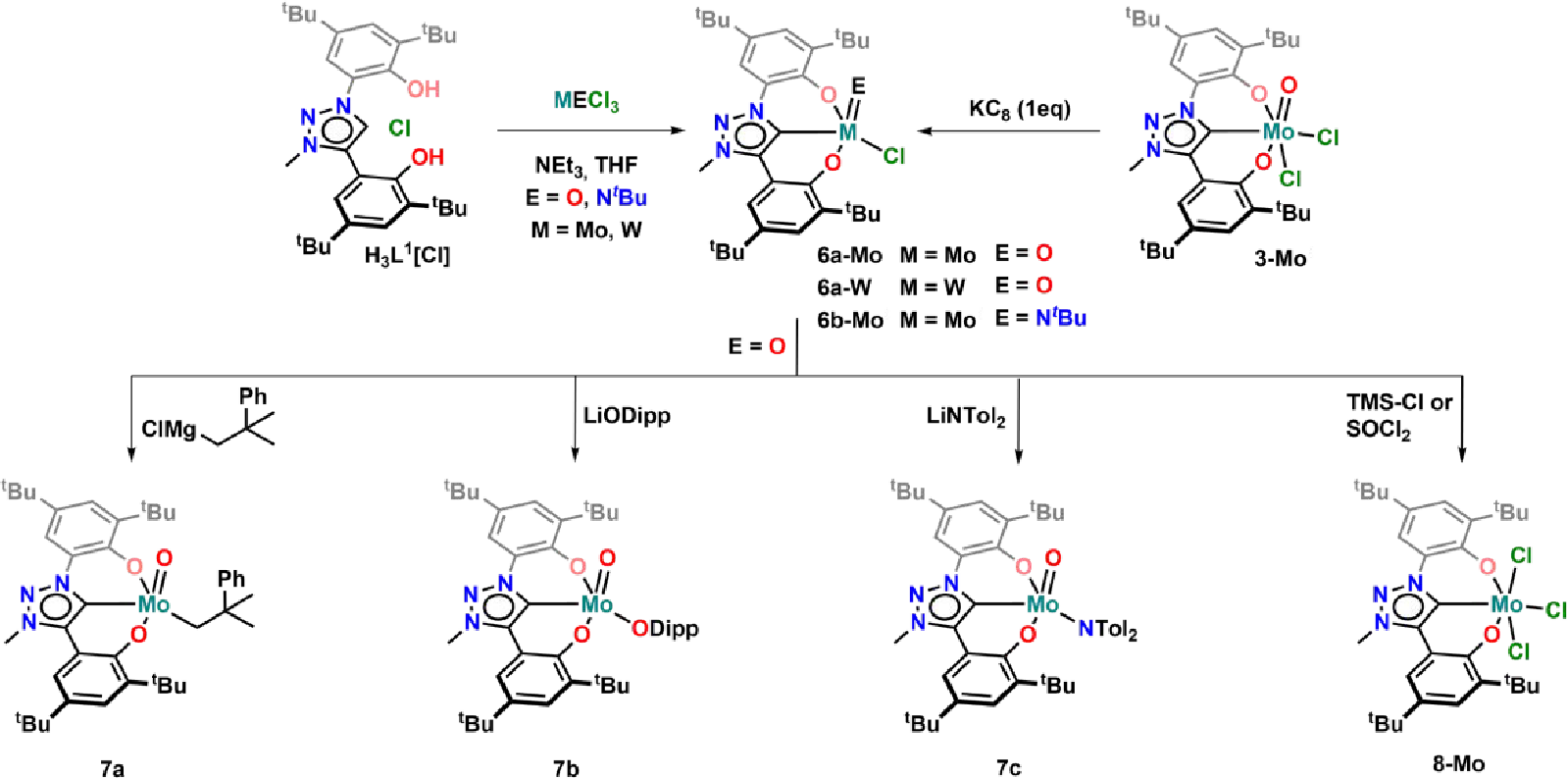
Synthesis of M­(V) complexes via one electron reduction of **3-M** (M = Mo, W) or direct synthesis from the proligand **[H**_**3**_ **L** ^**1**^ **]­[Cl]** and **MOCl**_**3**_/**Mo­(N** ^**t**^ **Bu)­Cl**_**3**_ **(DME)** precursors and subsequent functionalization reactions using grignard, phenolate, amide and chlorination Reagents

**Scheme 4:**
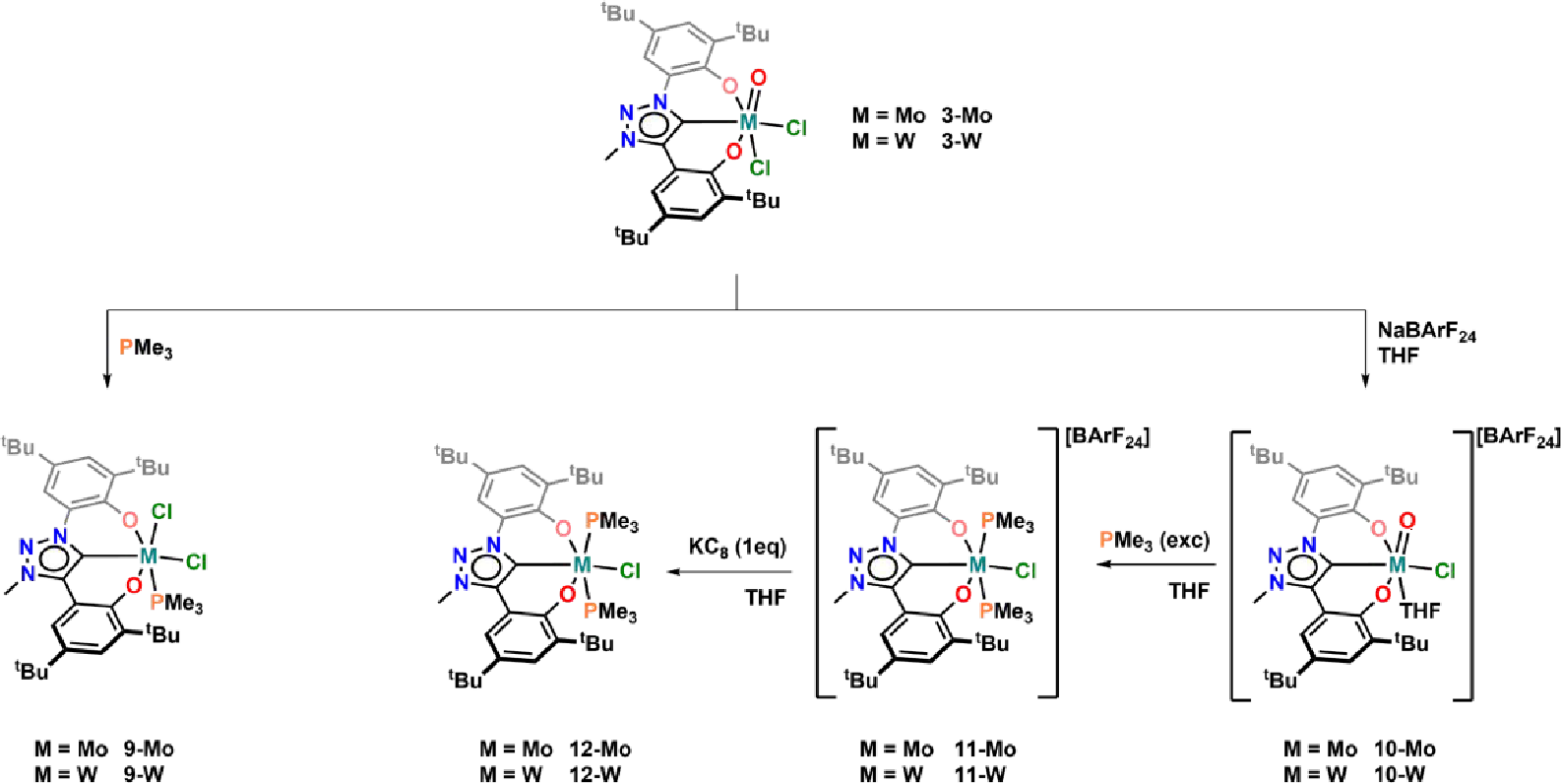
Diverging reactivity in phosphine mediated reduction of M­(VI) complexes prior and after halide abstraction yielding either neutral M­(IV) dichloro complexes **9-M** or cationic M­(IV) diphosphine complexes **11-M**, of which the latter can be further reduced to the neutral M­(III) complexes **12-M**

**6a-Mo** can undergo various salt metathesis reactions, i.e., with phenolates (**7b**), anilides (**7c**) or alkyls (**7a**). This type of reactivity was also observed with vanadium­(V) oxo complexes, with the exception of compound **7a**.^95^ Since structurally related vanadium diisopropylphenolate or ditolylanilide complexes have been already reported in the literature a detailed discussion of the complexes will be omitted here (EPR: Figures S103 and S104, X-ray structures: Figures S109 and S108 and Tables S2 and S3).^93,95^ With an effective magnetic moment of 1.74 μ_B_ (Figure S39) the alkyl complex **7a** is well in line with d^1^-configured Mo­(V) metal complexes. The addition of an alkyl group toward **6a-Mo** is further indicated by EPR spectroscopy, showing a g-value of 1.962 and an *a*_iso_(Mo) value of 127 MHz (Figure 4). In addition, an intense hyperfine coupling with the additional alkyl protons was observed (*a*_iso_(H) = 25 MHz), strongly supporting the assignment as an alkyl complex. Structural investigations showed a 5-fold coordinate molybdenum center, which is in between a trigonal bipyramidal and a square pyramidal coordination environment (τ_5_ = 0.43). The metal carbene distance Mo–C1 in **7a** is 2.128(6) Å, which is shorter compared to the Mo-alkyl distance of 2.175(6) Å for Mo1–C40. Given the presence of an oxo ligand in **6a-Mo**, we became curious if the last oxo group can be exchanged by chloride atoms. Treatment of **6a-Mo** with an excess of TMS-Cl or SOCl_2_ resulted in an oxygen atom exchange reaction and the triazolylidene complex **8-Mo** could be isolated quantitatively from the reaction mixture after workup. Assignment of a d^1^-configured oxidation state was again deduced from EPR spectroscopy showing the expected seven-line pattern for molybdenum (Figure 4, right, *g* = 1.947, *a*_iso_(Mo) = 111 MHz) along with an effective magnetic moment of 1.42 μ_B_ determined by NMR Evans method (Figure S42). Compared to the Mo–C distance in the oxo complexes **6a-Mo** the Mo1–C1 distance in **8-Mo** is 2.118(6) Å. Although the direct synthesis of **8-Mo** was attempted from **[H**_**3**_ **L** ^**1**^ **]­[Cl]** and MoCl_5_, no product formation was observed. This could be reasoned by the Lewis-acidity of MoCl_5_, which could result in ligand decomposition.

Attempts of obtaining M­(IV) complexes through the addition of two equivalents of potassium graphite to **3-M** failed. Also one electron reduction of the trichloro complexes **8-Mo** using tin shots did not facilitate the formation of a M­(IV) dichloro complexes as previously reported by Hu and colleagues.^100^ Thus, the Mo­(IV) oxidation state was targeted *via* deoxygenation chemistry using phosphines as oxygen atom acceptor reagents. Addition of trimethylphosphine PMe_3_ to **3-M** yielded a defined product, which after workup and crystallization was identified as the anticipated Mo­(IV) complexes **9-M** (M = Mo, W; Figure 5). ^1^H NMR investigations of these complexes show a paramagnetic nature for the molybdenum complex **9-Mo** (Figure S43, μ_eff_ = 2.34 μ_B,_ d^2^), while the tungsten analogue **9-W** was found to be diamagnetic (Figure S44). Such difference in the magnetic behavior is not entirely unexpected and larger spin coupling effects (SOC) in W­(IV) (5d metals) often give access to diamagnetic complexes, while their Mo­(IV) counterparts (4d complexes) are paramagnetic.^118–121^ ^31^P­{^1^H} NMR spectroscopy further showed a signal at −157.8 ppm for **9-W** (Figure S46) and a ^1^ *J*_WP_ coupling of 370 Hz, indicating the presence of a coordinated trimethylphosphine ligand. Proof of the successful synthesis of molybdenum­(IV) and tungsten­(IV) complexes was given by X-ray diffraction studies, performed on single crystals grown from THF/pentane and THF/Et_2_O for **9-Mo** and **9-W** respectively. Both complexes are 6-fold coordinated by the three donor atoms of the triazolylidene ligand, two chloride ligands and the trimethylphosphine donor. The metal triazolylidene distances M1–C1 were found to be 2.112(2) and 2.094(8) Å in **9-Mo** and **9-W** respectively. The metal phosphine distances M1–P40 can be found at 2.5074(10) and 2.45(2) Å, which is in the range of previously reported molybdenum/tungsten­(IV) trimethylphosphine complexes.^122–129^

**Figure 5:**
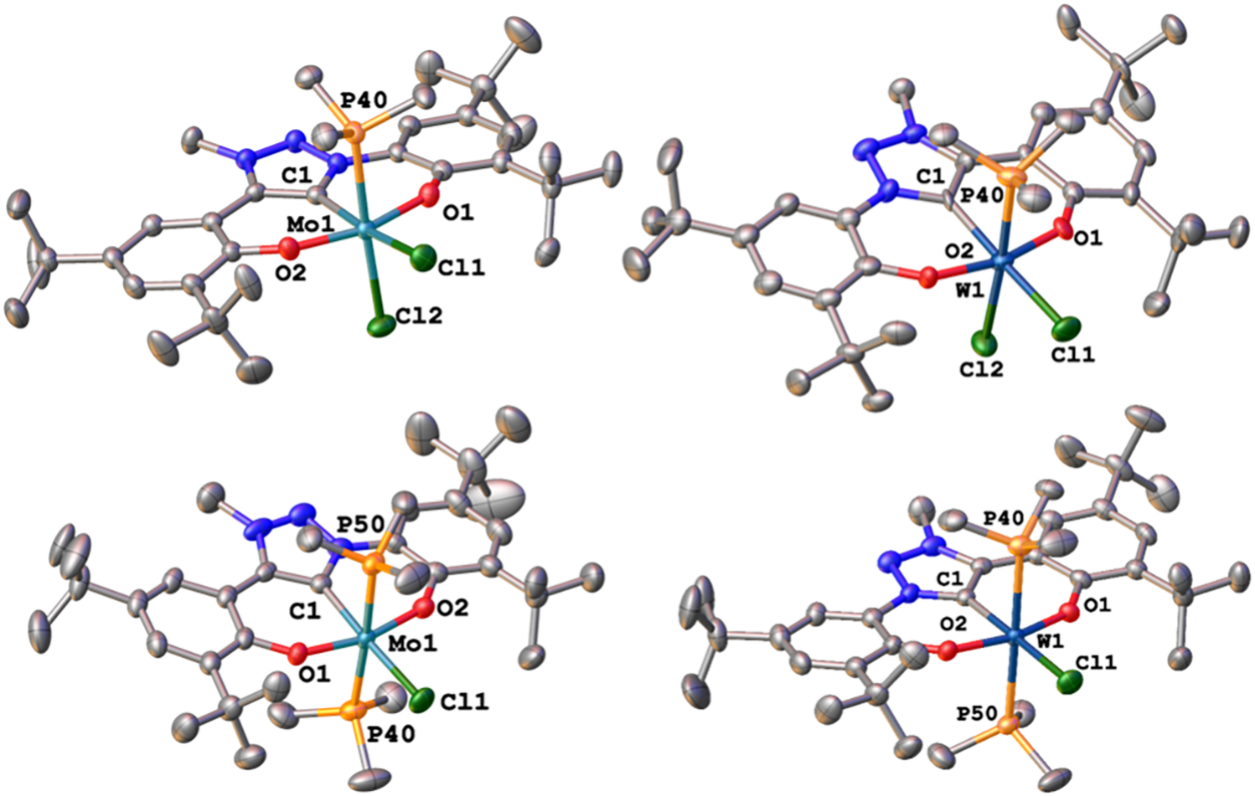
Molecular structures of the neutral M­(IV) dichloro complexes **9-Mo** and **9-W** (top) and the cationic M­(IV) complexes **11-Mo** and **11-W** (bottom). Hydrogen atoms, lattice solvent molecules and [BArF_24_]^−^ counterions have been omitted for clarity.

However, given the fact that the deoxgenation reactions to complexes **9-M** are rather slow (up to 2 weeks for **9-W**), we sought for more reactive conditions, to achieve the desired M­(VI) to M­(IV) reduction chemistry. Thereby we found that halide abstraction vastly speeds up the reaction and addition of sodium tetrakis­[(3,5-trifluoromethyl)­phenyl]­borate (NaBArF_24_) prior to trimethylphosphine addition gives access to the cationic M­(IV) complexes **11-M** (M = Mo, W) in good yields. We propose that this reaction proceeds *via* the intermediate formation of the Mo­(VI) cations **10-M**, which however were not isolated. ^31^P NMR spectroscopy displayed new resonances at −66.9 and −66.2 ppm for **11-Mo** and **11-W** respectively (Figure S52/S58). In the case of the tungsten complex **11-W** a ^1^ *J*_PW_ coupling of 285 Hz is observed (Figure S58), which is comparable to **9-W** (*vide supra*). Examination of the ^1^H NMR spectra of the complexes indicated the presence of a BArF_24_ counterion (Figures S50/S56), while a clear assignment of the triazolylidene carbon NMR signal was not possible. Proof for the formation of a cationic M­(IV) species is given by X-ray diffraction analysis of crystals of **11-M** grown by concentrated diethyl ether solutions at −40 °C. The metal centers are in a distorted octahedral coordination environment being coordinated by the OCO donor atoms of the triazolylidene ligand, two *trans* standing trimethylphosphine donors and a chloride ligand, while the BArF_24_ counterion shows no direct interaction with the metal centers. The metal carbene distances were found at 2.090(3) and 2.087(3) Å for **11-Mo** and **11-W** respectively, which is comparable to the neutral complexes **9-M** (*vide supra*). Surprisingly both solutions and solid material of complexes **11-Mo** and **11-W** can be handled in air. With the M­(IV) complexes in hand we were interested in their electrochemical properties and if further reduction of the complexes would be possible. Cyclic voltammograms recorded in THF at room temperature (Figure 6) revealed the presence of three quasi-reversible redox processes for both **11-Mo** and **11-W** respectively (Figures S106 and S107), one at 0.30/0.01 V *vs.* [Fc]/[Fc]^+^ (**11-Mo**/**11-W**) and two at −0.93/–1.39 and −2.22/–2.50 V *vs*. [Fc]/[Fc]^+^ (Table S1).

**Figure 6:**
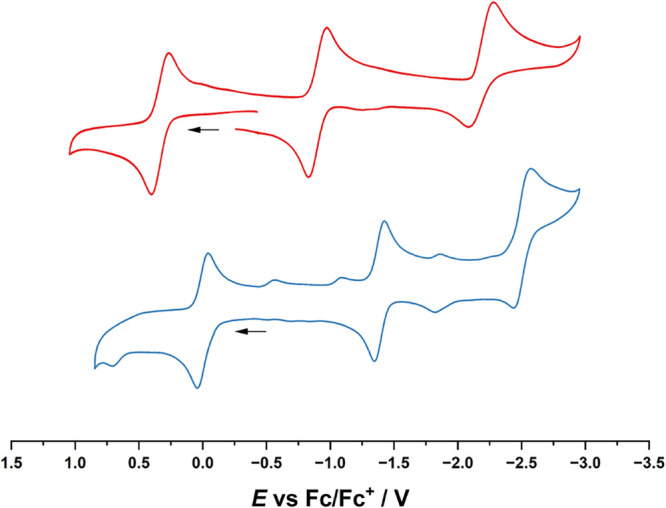
Cyclic voltammograms of complexes **11-Mo** (red trace) and **11-W** (blue trace) measured in 0.15 M NBu_4_PF_6_ in THF. Please note that for the tungsten complex **11-W** the 2nd cycle of the CV is shown and that the small waves in the CV of **11-W** result from follow-up processes induced by degradation.

The first redox process at a positive potential can most likely be attributed to a ligand centered oxidation process,^34,84^ However, given the comparably low potentials of 0.3/0.01 V vs [Fc]/[Fc]^+^ a metal centered oxidation process cannot be fully excluded. Unfortunately, all attempts to isolate the oxidized species have failed so far for both **11-Mo** and **11-W**. Contrarily, the two reductive processes are most likely metal-centered processes giving access to M­(III) and M­(II) complexes (M = Mo, W). Indeed, chemical reduction with one equivalent of potassium graphite yielded M­(III) complexes **12-M** (M = Mo, W) as green crystals after crystallization from pentane (Figure 7). Both complexes are sensitive toward air and moisture and crystals of **12-W** were observed to decompose within a few minutes, even when covered with a layer of perfluorinated oil. Both **12-Mo** and **12-W**, could be structurally characterized showing the same coordination environment around the metal centers as in **11-M** but without the presence of the BArF_24_ counterion. The M-C distances were found to be 2.099(10) and 2.067(8) Å in **12-Mo** and **12-W** respectively, being in the same range as in the corresponding **11-M** complexes. Since the M­(IV) cations **11-M** were diamagnetic in nature, the M­(III) complexes are proposed to be spin 1/2-systems (d^3^ with one unpaired electron), which is in line with their effective magnetic moments of 1.69 and 1.37 μ_B_ (Figures S62 and S63). The isolation of monomeric M­(III) complexes supported by the OCO ligand framework is highly remarkable as we^99^ and Hu^100^ have shown, that reduction of suitable precursors in the absence of PMe_3_ leads to the formation Mo–Mo dimers with a Mo–Mo triple bond. Notably, reduction of the oxo complexes **3-Mo** with 1,4-diTMS-2,3,5,6-tetramethyl-dihydropyrazine also leads to the formation of the dimeric Mo­(III) complexes **E** (Figure 1) along with many other unidentified reaction products. Unfortunately, all attempts to reduce the M­(IV) complexes to a putative M­(II) oxidation state (as indicated by CV, *vide supra*) have failed so far.

**Figure 7:**
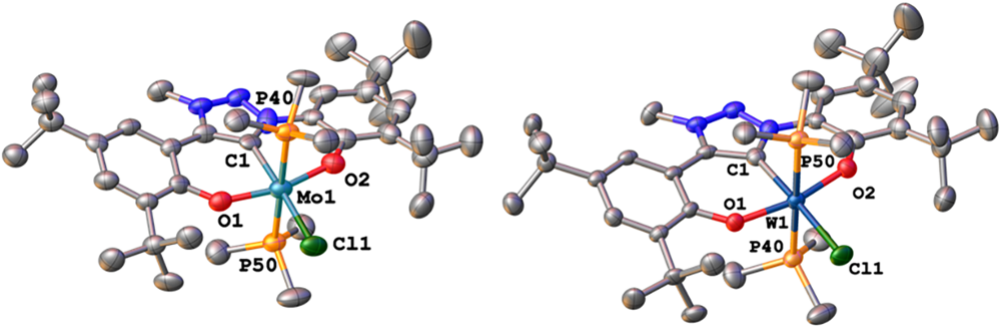
Molecular structures of the neutral M­(III) complexes **12-Mo** and **12-W**. Hydrogen atoms and lattice solvent molecules have been omitted for clarity.

## Conclusion

We have presented the synthesis of 22 new triazolylidene molybdenum and tungsten complexes spanning four oxidation states between +III and +VI. The work shows an unexpected, yet rich coordination and redox chemistry supported by the dianionic triazolylidene framework. Despite the commonly questioned stability of early transition metal carbene bonds, this work shows that anionic tethering vastly enhances their stability, leading to metal carbene bonds that even withstand treatment with thionyl chloride. The newly reported Mo­(V) trichloride complex is an excellent precursor to further study the coordination chemistry of molybdenum and opens the possibility to synthesize novel alkylidene and alkylidyne complexes. Contrarily, the low-valent M­(IV) and M­(III) complexes in combination with their rich redox-chemistry are well-suited precursors for small molecule activation chemistry. Overall, the study highlights the versatility and flexibility of triazolylidene ligands in early transition metal chemistry and lays the foundation for a rich and modular early transition metal chemistry, supported by triazolylidenes and other mesoionic carbenes with potential applications in (metathesis) catalysis, small molecule activation and atom transfer reactions.

## Experimental Section

### General Information

Unless stated otherwise, all transformations were conducted under an inert atmosphere using standard Schlenk techniques or an argon filled glovebox. High temperature reactions were carried out in preheated aluminum blocks. Low temperature reactions were performed using a precooled aluminum block or dewar vessels filled with cooling mixtures consisting of acetone/liquid nitrogen. Solvents for synthetic purposes were purified using an *MBraun SPS* system and stored over activated molecular sieves. Triethylamine was dried by refluxing over calcium hydride, subsequent distillation, and storage over activated molecular sieves. Solvents used for aqueous workup or chromatography (technical grade) were used as received by a commercial supplier. Chemicals and reagents were purchased from *Sigma-Aldrich*, *BLD* or *Alfa Aesar* and used as received. Deuterated solvents (benzene-*d*_6_ and dichloromethane-*d*_2_) were stored over activated molecular sieves. The ligand precursor **H**_**3**_ **L** ^**1**^ and complexes **1-Mo** and **1-W** were prepared following literature known procedures^84,97^ NMR spectra were recorded on a *Bruker Ascend 400* spectrometer. ^1^H and ^13^C­{^1^H} chemical shifts are reported in ppm and calibrated using residual solvent resonances. Elemental analyses (C, H, N) were performed on a *Vario Microtube* instrument. ATR-Infrared spectroscopy was conducted on a *Bruker α* IR spectrometer. X-ray diffraction crystallography was performed at the University of Innsbruck. All crystals were kept at 120(2) K or 153(2) K throughout data collection. Data collection was performed using the *ApexIV* software package on a Bruker D8 Quest instrument. Data refinement and reduction was performed using the *Bruker ApexIV* suite 2021. All structures were solved with SHELXT^130^ and refined using the *OLEX 2* software package^131^ and SHELXL.^132^ Strongly disordered solvent molecules have been removed using the SQUEEZE operation.^133^ All nonhydrogen atoms were refined anisotropically, and hydrogen atoms were included at the geometrically calculated positions and refined using a riding model.

### Synthetic Procedures

#### Mo^VI^L^1^O­(N*^t^* Bu) (**2-Mo**)

To a 100 mL Schlenk flask containing a solution of the ligand precursor **H**_**3**_ **L** ^**1**^ **Cl** (789 mg, 1.0 equiv, 1.49 mmol) in THF (10 mL), triethylamine (0.90 mL, 3.5 equiv, 5.2 mmol) was added via syringe at ambient temperature. The resulting bright orange suspension was kept at room temperature for approximately 60 min, before it was added dropwise to a 100 mL J. Young Schlenk flask containing a solution of [MoO­(N^*t*^Bu)­Cl_2_(dme)] (511 mg, 1 equiv, 1.49 mmol) in THF (10 mL). The suspension gradually turned pale green. After 24 h, insoluble salts were removed by cannula filtration, and the residue was washed with THF (3 × 5 mL). The filtrate was concentrated to a fifth of the original volume, or until the product started to precipitate. Precipitation was induced by slow addition of *n*-hexane via cannula and vigorous stirring for approximately 90 min. The pale-yellow precipitate was collected by filtration through a medium porosity Schlenk-frit, washed with *n*-hexane (3 × 10 mL) and dried *in vacuo* to obtain an off-white to pale green powder. The product is sensitive to moisture and should be kept under an atmosphere of argon. Yield: 53% (531 mg, 0.79 mmol). ^1^H NMR (400 MHz, Benzene-*d*_6_) δ 8.15 (d, *J* = 2.5 Hz, 1H), 7.69 (d, *J* = 2.4 Hz, 1H), 7.67 (d, *J* = 2.5 Hz, 1H), 7.05 (d, *J* = 2.4 Hz, 1H), 3.14 (s, 3H), 1.76 (s, 9H), 1.76 (s, 9H), 1.57 (s, 9H), 1.39 (s, 9H), 1.34 (s, 9H). ^13^C­{^1^H} NMR (101 MHz, Benzene-*d*_6_) δ 164.3, 160.3, 153.5, 143.6, 143.0, 141.3, 140.3, 139.3, 126.0, 125.3, 119.1, 114.0, 71.1, 39.0, 36.4, 36.2, 34.7, 34.5, 31.8, 31.8, 31.5, 30.5, 30.4. IR (ATR, neat, cm^–1^): 2961, 2869, 1525, 1482, 1448, 1423, 1360, 1296, 1258, 1221, 1151, 1129, 1076, 1031, 923, 880, 853, 800, 774, 759, 708, 692, 639, 549, 490, 469, 447, 412. UV–vis–NIR: λ_max_ = 327 nm (ε = 31,930 L mol^–1^ cm^–1^), 362 nm (ε = 34,210 L mol^–1^ cm^–1^).

#### Mo^VI^L^1^OCl_2_ (**3-Mo**)

A 100 mL Schlenk tube was charged with **1-Mo** (1.24 g, 1.0 equiv, 2.00 mmol) and dry dichloromethane (30 mL) under an atmosphere of argon. Chlorotrimethylsilane (2.54 mL, 10 equiv, 20.0 mmol) was added dropwise via syringe, resulting in a color change to wine-red. The mixture gradually turned dark green over the course of several hours. After 2 days at room temperature, volatiles were removed *in vacuo*. The residue was suspended in dry hexane (50 mL), stirred for 30 min and filtered through a medium porosity Schlenk frit. A moisture sensitive, blackish-green residue was dried *in vacuo* and brought inside an argon filled glovebox. Exposure to moist air results in HCl evolution and reisolation of **1-Mo**. Yield: 92% (1.24 g, 1.84 mmol). ^1^H NMR (400 MHz, Dichloromethane-*d*_2_) δ 8.24 (d, *J* = 2.2 Hz, 1H), 7.85 (d, *J* = 2.1 Hz, 1H), 7.71 (d, *J* = 2.1 Hz, 1H), 7.65 (d, *J* = 2.3 Hz, 1H), 4.71 (s, 3H), 1.53 (s, 9H), 1.52 (s, 9H), 1.43 (s, 9H), 1.42 (s, 9H). ^13^C­{^1^H} NMR (101 MHz, Dichloromethane-*d*_2_) δ 159.6, 156.9, 149.4, 147.4, 147.1, 139.4, 138.9, 138.7, 127.7, 126.5, 124.8, 119.4, 114.4, 114.3, 42.1, 36.1, 36.0, 35.4, 35.3, 31.5, 31.4, 30.1, 30.0. IR (ATR, neat, cm^–1^): 2957, 2908, 2869, 1525, 1480, 1466, 1445, 1433, 1413, 1396, 1364, 1323, 1296, 1260, 1233, 1211, 1190, 1156, 1129, 1088, 1078, 1053, 917, 878, 864, 798, 759, 749, 714, 694, 645, 569, 537, 474, 418. UV–vis–NIR: λ_max_ = 327 nm (ε = 63,640 L mol^–1^ cm^–1^), 352 nm (ε = 31,370 L mol^–1^ cm^–1^). Elemental analysis calcd (%) for C, 55.36 H, 6.44 N, 6.25 found C, 53.67 H, 6.55 N, 6.02. The low carbon value most likely results from metal carbide formation which is typical for group VI metals.

#### Mo^VI^L^1^(N*^t^* Bu)­Cl_2_ (**4-Mo**)

In an argon filled glovebox, a 100 mL Schlenk tube was charged with **2-Mo** (247 mg, 1.0 equiv, 0.367 mmol) and dry toluene (30 mL) (alternatively dichloromethane). The flask was brought outside the glovebox and chlorotrimethylsilane (1.00 mL, 22 equiv, 7.88 mmol) was added dropwise via syringe, resulting in a color change to dark brown. A dark blue precipitate started to form after 2 h. After 2 days at room temperature, the mixture was concentrated *in vacuo* to approximately 5 mL of volume. Dry hexane (50 mL) was added and the suspension stirred at ambient temperature for 30 min, before it was filtered through a medium porosity Schlenk frit. A dark blue residue was dried *in vacuo*. Yield: 89% (237 mg, 0.325 mmol). ^1^H NMR (400 MHz, Benzene-*d*_6_) δ 8.31 (d, 1H), 7.69 (d, *J* = 1.5 Hz, 1H), 7.68 (m, 1H), 7.10 (d, 1H), 3.05 (s, 3H), 1.70 (s, 9H), 1.68 (s, 9H), 1.32 (s, 9H), 1.29 (s, 9H), 0.93 (s, 9H). ^13^C­{^1^H} NMR (101 MHz, Benzene-*d*_6_) δ 161.7, 161.5, 153.8, 143.2, 142.7, 140.0, 138.4, 138.3, 126.4, 125.9, 123.0, 118.9, 113.6, 112.4, 74.6, 36.2, 36.1, 34.8, 34.7, 31.7, 31.6, 30.3, 28.7. IR (ATR, neat, cm^–1^): 2955, 2906, 2869, 1482, 1450, 1433, 1417, 1394, 1362, 1329, 1294, 1237, 1209, 1158, 1127, 1086, 1060, 921, 878, 849, 802, 757, 719, 692, 643, 602, 590, 561, 480, 447, 414. UV–vis–NIR: λ_max_ = 327 nm (ε = 41940 L mol^–1^ cm^–1^), 361 nm (ε = 43750 L mol^–1^ cm^–1^), 579 nm (ε = 9120 L mol^–1^ cm^–1^). Elemental analysis calcd (%) for C_35_H_52_N_4_O_2_Cl_2_Mo_1_ C, 57.77 H, 7.20 N, 7.70 found C, 57.49 H, 7.36 N, 7.58.

#### [W^VI^L^1^O­(OTMS)­Cl] (**3-W**′)

Inside an argon filled glovebox, a 10 mL scintillation vial was charged with **1-W** (176 mg, 1.0 equiv, 0.250 mmol) and toluene (2 mL). Chlorotrimethylsilane (140 mg, 5.0 equiv, 1.25 mmol) was added dropwise, resulting in a color change to bright yellow. After 24 h at room temperature, the mixture was filtered through a pipet equipped with a glass fiber filter and the filtrate was evaporated to dryness, leaving a bright yellow solid, which was washed with hexane (2 × 2 mL) and dried *in vacuo*. Yield: 94% (191 mg, 0.235 mmol) ^1^H NMR (400 MHz, Benzene-*d*_6_) δ 8.25 (d, *J* = 2.4 Hz, 1H), 7.72 (d, *J* = 2.3 Hz, 1H), 7.70 (d, *J* = 2.4 Hz, 1H), 7.03 (d, *J* = 2.3 Hz, 1H), 2.90 (s, 3H), 1.73 (s, 9H), 1.72 (s, 9H), 1.35 (s, 9H), 1.32 (s, 9H), 0.68 (s, 9H). ^13^C­{^1^H} NMR (101 MHz, Benzene-*d*_6_) δ 168.8, 156.3, 149.3, 144.2, 143.3, 141.6, 140.4, 139.8, 126.3, 125.8, 119.3, 116.0, 114.2, 39.1, 36.3, 36.1, 34.9, 34.7, 31.7, 31.6, 30.6, 30.5, 3.0. IR (ATR, neat, cm^–1^): 2961, 2908, 2869, 1535, 1482, 1468, 1437, 1423, 1388, 1362, 1333, 1292, 1239, 1217, 1205, 1160, 1131, 1084, 1060, 1031, 960, 913, 876, 837, 802, 755, 717, 694, 643, 557, 478, 451, 420, 406. UV–vis–NIR: λ_max_ = 327 nm (ε = 29,970 L mol^–1^ cm^–1^), 360 nm (ε = 31,370 L mol^–1^ cm^–1^). Elemental analysis calcd (%) for C_34_H_52_N_3_O_4_Si_1_Cl_1_W_1_ C, 50.16 H, 6.44 N, 5.16 found C, 50.98 H, 7.24 N, 5.02.

#### W^VI^L^1^OCl_2_ (**3-W**)

A 100 mL Schlenk tube was charged with **1-W** (822 mg, 1.0 equiv, 1.17 mmol) and dry dichloromethane (20 mL) under atmosphere of argon. The light-yellow solution was cooled to −94 °C using an acetone/N_2(l)_ cooling mixture and thionyl chloride (0.10 mL, 1.2 equiv, 1.4 mmol) was added at once, resulting in a color change to dark orange. The mixture was warmed to ambient temperature, and the mixture kept stirring for another 60 min. Volatiles were removed *in vacuo*, and the residue was washed with hexanes (3 × 50 mL) to give an orange solid. Yield: 92% (812 mg, 1.07 mmol). ^1^H NMR (400 MHz, Benzene-*d*_6_) δ 8.17 (d, *J* = 2.4 Hz, 1H), 7.72 (d, *J* = 2.1 Hz, 1H), 7.70 (d, *J* = 2.3 Hz, 1H), 7.05 (d, *J* = 2.1 Hz, 1H), 2.96 (s, 3H), 1.70 (s, 8H), 1.69 (s, 8H), 1.29 (s, 8H), 1.28 (s, 8H). ^13^C­{^1^H} NMR (101 MHz, Benzene-*d*_6_) δ 163.4, 155.6, 148.6, 145.8, 144.9, 141.8, 141.5, 140.7, 139.1, 127.1, 126.4, 125.4, 119.0, 114.8, 113.8, 39.7, 36.1, 36.0, 35.0, 34.8, 31.6, 31.5, 30.2. IR (ATR, neat, cm^–1^): 2957, 2871, 1527, 1484, 1466, 1435, 1417, 1396, 1364, 1325, 1296, 1260, 1237, 1205, 1158, 1129, 1088, 1078, 1055, 949, 925, 886, 866, 800, 759, 749, 714, 696, 645, 563, 537, 471, 418. UV–vis–NIR: λ_max_ = 326 nm (ε = 39,600 L mol^–1^ cm^–1^), 351 nm (ε = 36,800 L mol^–1^ cm^–1^). Elemental analysis calcd. (%) for C_31_H_43_N_3_O_3_Cl_2_W_1_ C, 48.96 H, 5.70 N, 5.53 found C, 48.52 H, 5.86 N, 5.52.

#### General Procedure for M^VI^L^1^O­(Cat) (**5-M**, M = Mo, W)

In a 20 mL scintillation vial, a solution of 4,6-di-*tert*-butylcatechol in THF (1 mL) was treated with triethylamine. This mixture was added dropwise to a solution of complex **3** in THF (1 mL). After 2 h at room temperature, the mixture was filtered to remove all insoluble salts, and the filtrate was evaporated to dryness. The dark residue was redissolved in hexane, filtered, concentrated to approximately 2 mL and stored at −40 °C. Crystalline material was obtained within a few days, separated and dried *in vacuo*.

#### Mo^VI^L^1^O­(Cat) (**5-Mo**)

From **3-Mo** (100 mg, 1 equiv, 0.15 mmol), 4,6-di-*tert*-butylcatechol (33 mg, 1.0 equiv, 0.15 mmol) and triethylamine (60 mg, 2.5 equiv, 0.59 mmol). Dark blue, almost black needles. Yield: 86% (106 mg, 0.13 mmol). ^1^H NMR (400 MHz, Benzene-*d*_6_) δ 8.18 (d, *J* = 2.3 Hz, 1H), 7.66 (d, *J* = 2.3 Hz, 1H), 7.62 (d, *J* = 2.4 Hz, 1H), 7.03 (d, *J* = 2.4 Hz, 1H), 6.83 (s, 1H), 3.02 (s, 3H), 1.75 (s, 9H), 1.57 (s, 9H), 1.54 (s, 9H), 1.35 (s, 9H), 1.32 (s, 9H), 1.13 (s, 9H). ^13^C­{^1^H} NMR (101 MHz, Benzene-*d*_6_) δ 163.3, 160.2, 153.0, 142.3, 142.2, 141.5, 141.2, 140.0, 129.3, 126.6, 125.8, 123.9, 118.9, 113.7, 112.1, 109.9, 39.1, 36.3, 36.2, 35.2, 34.8, 34.6, 34.5, 34.4, 31.8, 31.7, 31.6, 30.2, 30.1, 30.0. IR (ATR, neat, cm^–1^): 2955, 2908, 2869, 1584, 1519, 1480, 1462, 1417, 1392, 1362, 1294, 1256, 1229, 1198, 1156, 1129, 1102, 1078, 1027, 996, 923, 853, 798, 757, 741, 712, 692, 639, 555, 510, 476. UV–vis–NIR: λ_max_ = 304 nm (ε = 61,930 L mol^–1^ cm^–1^), 487 nm (ε = 35,080 L mol^–1^ cm^–1^), 719 nm (ε = 46,850 L mol^–1^ cm^–1^). Elemental analysis calcd (%) for C_45_H_63_N_3_O_5_Mo_1_ C, 65.76 H, 7.73 N, 5.11 found C, 66.14 H, 7.83 N, 4.96.

#### W^VI^L^1^O­(Cat) (**5-W**)

From **3-W** (76 mg, 1 equiv, 0.10 mmol), 4,6-di-*tert*-butylcatechol (22 mg, 1.0 equiv, 0.10 mmol) and triethylamine (25 mg, 2.5 equiv, 0.25 mmol). Blood red needles. Yield: 88% (80 mg, 0.087 mmol). ^1^H NMR (400 MHz, Benzene-*d*_6_) δ 8.16 (d, *J* = 2.4 Hz, 1H), 7.65 (d, *J* = 2.3 Hz, 1H), 7.61 (d, *J* = 2.4 Hz, 1H), 7.04 (d, *J* = 2.3 Hz, 1H), 6.91 (d, *J* = 2.1 Hz, 1H), 6.87 (s, 1H), 3.05 (s, 3H), 1.79 (s, 9H), 1.56 (s, 9H), 1.52 (s, 9H), 1.31 (s, 9H), 1.29 (s, 9H), 1.19 (s, 9H). ^13^C NMR (101 MHz, Benzene-*d*_6_) δ 157.5, 150.6, 143.4, 142.4, 142.1, 141.5, 140.7, 126.9, 126.1, 125.0, 118.8, 113.6, 113.6, 110.9, 39.2, 36.1, 36.0, 35.0, 34.8, 34.6, 34.4, 32.1, 31.7, 31.6, 30.4, 30.2, 30.1. IR (ATR, neat, cm^–1^): 2955, 1482, 1415, 1362, 1296, 1239, 1202, 1160, 1033, 994, 939, 857, 800, 759, 723, 694, 608, 559, 471. UV–vis–NIR: λ_max_ = 327 nm (ε = 42,780 L mol^–1^ cm^–1^), 351 nm (ε = 42,580 L mol^–1^ cm^–1^), 486 nm (ε = 27,940 L mol^–1^ cm^–1^). Elemental analysis calcd. (%) for C_45_H_63_N_3_O_5_W_1_ C, 59.40 H, 6.98 N, 4.62 found C, 57.86 H, 7.55 N, 4.35. The low carbon value most likely results from metal carbide formation which is typical for group VI metals.

#### General Procedure for M^V^L^1^ECl (E = O, N*^t^* Bu)

In a 20 mL scintillation vial, a solution of **H**_**3**_ **L** ^**1**^ **Cl** in THF was treated with triethylamine. The bright orange suspension was cooled to −40 °C and added dropwise to a suspension of MOCl_3_L_2_ or Mo­(N^*t*^Bu)­Cl_3_(DME) in THF, giving a dark brown suspension. The mixture was warmed to room temperature and stirred overnight. Insoluble materials were filtered off, and the filtrate was evaporated to dryness. The residue was dissolved in diethyl ether and filtered again; insoluble residues were discarded. The solvent was removed *in vacuo*, and the residue was washed successively with hexane and pentane and dried *in vacuo*.

#### Mo^V^L^1^OCl (**6a-Mo**)

From **H**_**3**_ **L** ^**1**^ **Cl** (500 mg, 1.0 equiv, 0.947 mmol), triethylamine (335 mg, 3.5 equiv, 3.31 mmol) and MoOCl_3_ (207 mg, 1.0 equiv, 0.947 mmol). Orange-brown powder. Yield: 87% (527 mg, 0.824 mmol). μ_eff_ (Evans’ method, Dichloromethane-*d*_2_) = 1.49 μ_B_, EPR (298 K): *g*_iso_ = 1.950, *a*_iso_ = 137 MHz; Elemental analysis (%) calc’d for C_31_H_43_ClMoN_3_O_3_: C, 58.44; H, 6.80; N, 6.60; found C, 58.72; H, 7.13; N, 6.14.

#### W^V^L^1^OCl (**6a-WO**)

From **H**_**3**_ **L** ^**1**^ **Cl** (200 mg, 1.0 equiv, 0.379 mmol), triethylamine (134 mg, 3.5 equiv, 1.33 mmol) and WOCl_3_(THF)_2_ (171 mg, 1.0 equiv, 0.379 mmol). The residue was extracted using dichloromethane instead of diethyl ether. Dark purple powder. Yield: 48% (133 mg, 0.182 mmol). μ_eff_ (Evans’ method, Benzene-*d*_6_) = 1.19 μ_B_, EPR (298 K): *g*_iso_ = 1.813, *a*_iso_ = 267 MHz, Elemental analysis (%) calc’d for C_31_H_43_ClN_3_O_3_W·0.6 CH_2_Cl_2_: C, 48.91; H, 5.74; N, 5.42; found C, 48.56; H, 6.14; N, 5.32.

#### MoVL^1^(N*^t^* Bu)Cl (**6b-Mo**)

From **H**_**3**_ **L** ^**1**^ **Cl** (145 mg, 1 equiv, 0.275 mmol), triethylamine (100 mg, 3.5 equiv, 0.96 mmol) and Mo­(N^*t*^Bu)­Cl_3_(DME) (100 mg, 1 equiv, 0.275 mmol). Orange brown powder. Yield: 78% (149 mg, 0.215 mmol). μ_eff_ (Evans’ method, Benzene-*d*_6_) = 1.78 μ_B_, EPR (298 K): *g*_iso_ = 1.958, *a*_iso_(Mo) = 135 MHz, *a*_iso_(N) = 9.25 MHz, Elemental analysis (%) calc’d for C_35_H_52_N_4_O_2_Cl_1_Mo_1_ C, 60.73 H, 7.57 N, 8.09; found C, 60.08 H, 7.57 N 7.82.

#### Mo^V^L^1^O­(Neophyl) (**7a**)

Complex **6a-Mo** (64 mg, 1 equiv, 0.1 mmol) and (Neophyl)­MgCl­(THF)_2_ (34 mg, 1 equiv, 0.1 mmol) were dissolved in −40° cold toluene (15 mL) and stirred for 24 h at room temperature. The reaction mixture was filtered and carefully washed with cold pentane (5 mL) to give complex **7a** as a yellow brown powder. Yield: 66% (48 mg, 0.066 mmol) X-ray quality crystals were grown from a concentrated dichloromethane/hexane solutions at −40 °C. μ_eff_ (Evans’ method, Benzene-*d*_6_) = 1.74 μ_B_, EPR (298 K): *g*_iso_ = 1.962, *a*_iso_(Mo) = 127 MHz, *a*_iso_(H) = 25 MHz, Elemental analysis (%) calc’d for C_41_H_56_MoN_3_O_3_·0.5 CH_2_Cl_2_: C, 63.96; H, 7.63; N, 5.39; found C, 63.86; H, 7.52; N, 5.48.

#### Mo^V^L^1^O­(ODipp) (**7b**)

Complex **6a-Mo** (64 mg, 1 equiv 0.1 mmol) and lithium diisopropylphenolate (18.4 mg, 1 equiv, 0.1 mmol) were dissolved in a −40 °C cold mixture of diethyl ether and pentane (1:1) and stirred overnight. Filtration, evaporation and washing with −40 °C cold pentane affords the desired phenolate complex **7b** as a brown powder. X-ray quality crystals were grown from a concentrated DCM/hexane solution at −40 °C. Yield: 77% (60 mg, 0.077 mmol). μ_eff_ (Evans’ method, Benzene-*d*_6_) = 1.71 μ_B_, EPR (298 K): *g*_iso_ = 1.949, *a*_iso_(Mo) = 140 MHz, Elemental analysis (%) calc’d for C_43_H_60_MoN_3_O_4_·0.15 CH_2_Cl_2_: C, 65.47; H, 7.68; N, 5.31; found C, 65.33; H, 7.90; N, 5.15

#### Mo^V^L^1^O­(NTol_2_) (**7c**)

Complex **6a-Mo** (64 mg, 1 equiv 0.1 mmol) and lithium ditolylanilide (20.3 mg, 1 equiv, 0.1 mmol) were dissolved in a −40 °C cold mixture of diethyl ether and pentane (1:1) and stirred overnight. Filtration, evaporation and washing with −40 °C cold pentane affords the desired anilide complex **7c** as a blue powder. X-ray quality crystals were grown from a concentrated DCM/hexane solution at −40 °C. Yield: 84% (67 mg, 0.084 mmol). μ_eff_ (Evans’ method, Benzene-*d*_6_) = 1.72 μ_B_, EPR (298 K): *g*_iso_ = 1.950, *a*_iso_(Mo) = 130 MHz, Elemental analysis (%) calc’d for C_45_H_57_MoN_4_O_3_·0.25 CH_2_Cl_2_: C, 66.35; H, 7.08; N, 6.84; found C, 66.32; H, 7.10; N, 6.71.

#### Mo^V^L^1^Cl_3_ (**8-Mo**)

Complex **6a-Mo** (50 mg, 1 equiv, 0.0785 mmol) was dissolved in dichloromethane (10 mL) and stirred for 5 min at room temperature. To this solution an excess of TMS-Cl or thionyl chloride is added (3–5 equiv) and the reaction mixture is stirred overnight at room temperature. The mixture gradually darkens over this time. The next day, the volume is concentrated to about 5 mL and hexane is slowly added to the reaction mixture, until the desired product begins to precipitate. Storage of the reaction mixture at −40 °C affords dark purple/blocks, which are filtered off to yield the desired trichloride complex **8-Mo**. Yield: 59% (32 mg, 0.0463 mmol) μ_eff_ (Evans’ method, Dichloromethane-*d*_2_) = 1.42 μ_B_, EPR (298 K): *g*_iso_ = 1.947, *a*_iso_ = 111 MHz; Elemental analysis (%) calc’d for C_31_H_43_Cl_3_MoN_3_O_2_·0.1 CH_2_Cl_2_: C, 53.33; H, 6.22; N, 6.00; found C, 53.06; H, 6.67; N, 5.61.

#### [Mo^IV^L^1^(PMe_3_)­Cl_2_] (**9-Mo**)

In a 20 mL scintillation vial, a suspension of [Mo^VI^L^1^OCl_2_] (100 mg, 0.149 equiv) in diethyl ether was treated with trimethylphoshine (exc.) at ambient temperature. After 24 h, solid materials were filtered off, washed with diethyl ether and dried *in vacuo*. Reddish-brown powder. Yield: 85% (93 mg, 127 mmol). ^1^H NMR (400 MHz, Dichloromethane-*d*_2_) δ 10.65 (d, *J* = 2.2 Hz, 1H), 7.76 (d, *J* = 2.1 Hz, 1H), 5.32 (s, 3H), 3.89 (d, *J* = 2.1 Hz, 1H), 1.25 (s, 9H), 1.15 (s, 9H), 0.54 (s, 9H), 0.53 (s, 9H), 0.35 (s, 1H), −15.73 (s, 9H). μ_eff_ (Evans’ method, Dichloromethane-*d*_2_) = 2.34 μ_B_. IR (ATR, neat, cm^–1^): 2952, 2908, 2869, 1480, 1445, 1421, 1394, 1362, 1294, 1245, 1202, 1151, 1127, 1078, 949, 921, 874, 851, 800, 757, 717, 694, 641, 557, 474, 404. UV–vis–NIR: λ_max_ = 327 nm (ε = 31,650 L mol^–1^ cm^–1^), 362 nm (ε = 33,500 L mol^–1^ cm^–1^), 443 nm (ε = 33,730 L mol^–1^ cm^–1^). Elemental analysis calcd. (%) for C_34_H_52_N_3_O_2_P_1_Cl_2_Mo_1_ C, 55.74 H, 7.15 N, 5.74 found C, 55.19 H, 7.24 N, 5.64. The low carbon value most likely results from metal carbide formation which is typical for group VI metals.

#### [W^IV^L^1^(PMe_3_)­Cl_2_] (**9-W**)

A 20 mL scintillation vial was charged with a suspension of [W^VI^L^1^OCl_2_] (52 mg, 1.00 equiv, 0.068 mmol) in a mixture of diethyl ether and THF (3:2, approximately 2 mL). Trimethylphosphine (exc.) was added to the orange suspension at room temperature. The vial was tightly capped and kept at room temperature for 7–9 days or until the mixture turned dark green. Diethyl ether was added while the mixture was vigorously stirred to precipitate the product. Precipitates were collected, washed with diethyl ether, and dried *in vacuo*. Dark green powder. Yield: 75% (42 mg, 0.051 mmol). ^1^H NMR (500 MHz, Dichloromethane-*d*_2_) δ 8.19 (d, *J* = 2.2 Hz, 1H), 7.85 (d, *J* = 2.3 Hz, 1H), 7.35 (d, *J* = 2.1 Hz, 1H), 7.30 (d, *J* = 2.3 Hz, 1H), 4.77 (s, 3H), 1.58 (s, 18H), 1.45 (s, 9H), 1.45 (s, 9H), 0.65 (d, *J* = 9.3 Hz, 9H). ^13^C NMR (126 MHz, Dichloromethane-*d*_2_) δ 159.6, 153.8, 145.0, 143.8, 138.0, 136.7, 136.3, 135.0, 126.6, 125.7, 121.1, 120.8, 116.1, 109.4, 40.7, 35.8, 35.6, 34.7, 34.3, 32.5, 32.2, 30.2, 30.0, 26.9, 26.7. ^31^P­{H} NMR (202 MHz, Dichloromethane-*d*_2_) δ −158.1 (^1^ *J*_P–W_ = 366.3 Hz). IR (ATR, neat, cm^–1^): 2963, 2908, 2871, 1480, 1460, 1433, 1421, 1390, 1364, 1337, 1288, 1241, 1200, 1184, 1121, 1086, 1074, 951, 921, 861, 802, 761, 753, 723, 708, 696, 676, 645, 561, 492, 469, 416. UV–vis–NIR: λ_max_ = 326 nm (ε = 78,880 L mol^–1^ cm^–1^), 398 nm (ε = 55,600 L mol^–1^ cm^–1^), 625 nm (ε = 28,090 L mol^–1^ cm^–1^). Elemental analysis calcd. (%) for C_34_H_52_N_3_O_2_P_1_Cl_2_W_1_ C, 49.77 H, 6.39 N, 5.12 found C, 49.89 H, 6.65 N, 5.19.

#### [Mo^IV^L^1^(PMe_3_)_2_Cl]­[BArF_24_] (**11-Mo**)

In a 20 mL scintillation vial, **3-Mo** (100 mg, 1.0 equiv, 0.100 mmol) was suspended in diethyl ether. Sodium tetrakis­[3,5-bis­(trifluoromethyl)­phenyl]­borate (132 mg, 1.00 equiv, 0.100 mmol) was added at once, resulting in a dark green solution. After 60 min at room temperature, trimethylphosphine (57 mg, 7.5 equiv, 0.74 mmol) was added, giving a blood red solution. The mixture was stirred overnight, filtered and concentrated to approximately a quarter of its original volume. Blood red blocks were obtained within a few days, separated, and dried *in vacuo*. Yield: 63% (152 mg, 0.093 mmol). ^1^H NMR (400 MHz, Dichloromethane-*d*_2_) δ 8.49 (d, *J* = 2.2 Hz, 1H), 8.06 (d, *J* = 2.1 Hz, 1H), 7.71 (dd, *J* = 5.2, 2.7 Hz, 8H), 7.55 (s, 4H), 7.51 (t, *J* = 2.0 Hz, 2H), 4.93 (s, 3H), 1.42 (s, 9H), 1.40 (s, 9H), 1.37 (s, 9H), 1.37 (s, 9H), 0.71 (s, 18H). ^13^C­{^1^H} NMR (101 MHz, Dichloromethane-*d*_2_) δ 162.9, 162.4, 161.9, 161.4, 154.5, 149.7, 149.4, 147.1, 138.5, 138.0, 135.2, 133.5, 129.4, 129.4, 129.4, 129.1, 129.1, 129.1, 128.8, 127.5, 126.4, 126.3, 125.8, 123.6, 120.9, 117.9, 117.8, 115.7, 115.4, 42.1, 35.8, 35.7, 35.3, 35.1, 31.9, 31.8, 30.0, 29.9, 14.4. ^31^P NMR (162 MHz, Dichloromethane-*d*_2_) δ −66.9. IR (ATR, neat, cm^–1^): 2967, 2914, 2877, 1609, 1482, 1429, 1354, 1274, 1243, 1182, 1162, 1125, 1115, 1072, 947, 898, 882, 864, 839, 800, 761, 745, 712, 696, 682, 670, 647, 618, 574, 471, 449. UV–vis–NIR: λ_max_ = 329 nm (ε = 71,160 L mol^–1^ cm^–1^), 351 nm (ε = 72,030 L mol^–1^ cm^–1^), 399 nm (ε = 68,340 L mol^–1^ cm^–1^), 504 nm (ε = 72,900 L mol^–1^ cm^–1^). Elemental analysis calcd. (%) for C_69_H_73_N_3_O_2_P_2_Cl_1_Mo_1_B_1_F_24_·1 CH_2_Cl_2_ C, 48.84 H, 4.539 N, 2.44 found C, 48.41 H, 4.51 N, 2.38.

#### [W^IV^L^1^(PMe_3_)_2_Cl]­[BArF_24_] (**11-W**)

Inside an argon filled glovebox, a 10 mL Schlenk tube was charged with tungsten complex **3-W** (100 mg, 1.0 equiv, 0.132 mmol), sodium tetrakis­[3,5-bis­(trifluoromethyl)­phenyl]­borate (117 mg, 1.0 equiv, 0.132 mmol), trimethylphosphine (50 mg, 5 equiv, 0.658 mmol) and THF (3 mL). The flask was closed, brought outside of the glovebox and heated to 50 °C, the mixture turned dark orange. After 48 h at 50 °C, the flask was cooled to room temperature and brought back inside the glovebox. The mixture was filtered and evaporated, redissolved in diethyl ether (5–10 mL) and filtered again. The residue was washed with diethyl ether until the washings were no longer colored orange. The filtrate was concentrated to a quarter of its original volume and left to crystallize at room temperature in a capped 20 mL vial. Crystalline material was obtained within a few days at room temperature. Dark orange needles. Yield: 45% (102 mg, 0.059 mmol). ^1^H NMR (400 MHz, Dichloromethane-*d*_2_) δ 8.25 (d, *J* = 2.3 Hz, 1H), 7.86 (d, *J* = 2.1 Hz, 1H), 7.72 (q, *J* = 2.6 Hz, 9H), 7.55 (s, 4H), 7.47 (d, *J* = 2.1 Hz, 1H), 7.45 (d, *J* = 2.3 Hz, 1H), 4.76 (s, 3H), 1.45 (s, 9H), 1.44 (s, 9H), 1.41 (s, 9H), 1.40 (s, 9H), 0.86 (t, *J* = 3.6 Hz, 18H). ^13^C­{^1^H} NMR (101 MHz, Dichloromethane-*d*_2_) δ 162.9, 162.4, 161.9, 161.4, 154.4 (triazolylidene-C), 148.0, 147.3, 146.9, 138.0, 137.9, 135.2, 131.8, 129.4, 129.1, 128.1, 127.0, 126.3, 123.6, 123.3, 121.7, 120.9, 117.9, 116.9, 112.4, 41.8, 35.9, 35.7, 35.4, 35.2, 31.7, 31.5, 30.0, 29.8, 15.4, 15.2, 15.1. ^31^P NMR (162 MHz, Dichloromethane-*d*_2_) δ −66.26 (^1^ *J*_P–W_ = 281.4 Hz). IR (ATR, neat, cm^–1^): 2965, 2916, 1609, 1482, 1431, 1354, 1274, 1235, 1180, 1162, 1115, 1068, 947, 898, 886, 866, 839, 802, 761, 743, 712, 696, 682, 670, 618, 565, 469, 449, 416. UV–vis–NIR: λ_max_ = 322 nm (ε = 110,230 L mol^–1^ cm^–1^), 415 nm (ε = 66,430 L mol^–1^ cm^–1^), 451 nm (ε = 59,490 L mol^–1^ cm^–1^), 544 nm (ε = 21,300 L mol^–1^ cm^–1^). Elemental analysis calcd. (%) for C_69_H_73_N_3_O_2_P_2_Cl_1_W_1_B_1_F_24_ C, 48.06 H, 4.27 N, 2.44 found C, 48.06 H, 4.22 N, 2.42.

#### Representative Procedure for [M^III^L^1^(PMe_3_)_2_Cl]

A solution of [M^IV^L^1^(PMe_3_)_2_Cl]­[BArF_24_] (1 equiv) in THF was added to a suspension of potassium graphite (1.1 equiv) in THF at −40 °C. The mixture immediately turned dark green. After 30 min at −40 °C, the mixture was filtered through a pipet equipped with a glass fiber filter. The dark green filtrate was evaporated to dryness, redissolved in diethyl ether, and filtered again. The filtrate was concentrated to approximately a quarter of its original volume and left to crystallize at −40 °C.

#### [Mo^III^L^1^(PMe_3_)_2_Cl] (**12-Mo**)

From **11-Mo** (50 mg, 1.0 equiv, 0.034 mmol) and potassium graphite (5 mg, 1.1 equiv, 0.037 mmol). Dark green needles. Yield: 81% (17 mg, 0.027 mmol). μ_eff_ (Evans’ method, Benzene-*d*_6_) = 1.69 μ_B_. IR (ATR, neat, cm^–1^): 2952, 2903, 2867, 1603, 1564, 1478, 1443, 1419, 1382, 1356, 1323, 1294, 1278, 1252, 1202, 1178, 1164, 1117, 1060, 939, 874, 847, 798, 768, 753, 729, 714, 690, 682, 667, 549, 531, 465, 410. UV–vis–NIR: λ_max_ = 332 nm (ε = 37,150 L mol^–1^ cm^–1^), 406 nm (ε = 14,960 L mol^–1^ cm^–1^), 650 nm (ε = 8300 L mol^–1^ cm^–1^). Elemental analysis calcd (%) for C_37_H_61_N_3_O_2_P_2_Cl_2_Mo_1_ C, 57.47 H, 7.95 N, 5.43 found C, 56.89 H, 7.69 N 5.13.

#### [W^III^L^1^(PMe_3_)_2_Cl] (**12-W**)

From **11-W** (50 mg, 1.0 equiv, 0.032 mmol) and potassium graphite (5 mg, 1.1 equiv, 0.037 mmol). Dark green needles. Yield: 71% (16 mg, 0.023 mmol). μ_eff_ (Evans’ method, Benzene-*d*_6_) = 1.37 μ_B_ IR (ATR, neat, cm^–1^): 2955, 2903, 1478, 1443, 1419, 1354, 1321, 1276, 1164, 1125, 1053, 1033, 939, 874, 849, 800, 751, 727, 714, 692, 682, 670, 547, 465. Due to the extremely high sensitivity of this sample toward air and moisture, no useful elemental analysis could be obtained.

## Supplementary Material



## Notes

One and multidimensional NMR data for all complexes (^1^H, ^13^C, ^31^P, ^1^H–^1^H COSY, ^1^H–^13^C HSQC, ^1^H–^13^C HMBC), EPR spectra, UV–vis–NIR spectra, IR spectra and electrochemistry for selected complexes, X-ray diffraction details (PDF)

## References

[1] Sau S. C., Hota P. K., Mandal S. K., Soleilhavoup M., Bertrand G. (2020). Stable abnormal N-heterocyclic carbenes and their applications. Chem. Soc. Rev..

[2] Crabtree R. H. (2013). Abnormal,
mesoionic and remote N-heterocyclic carbene complexes. Coord. Chem. Rev..

[3] Schweinfurth D., Hettmanczyk L., Suntrup L., Sarkar B. (2017). Metal Complexes of
Click-Derived Triazoles and Mesoionic Carbenes: Electron Transfer,
Photochemistry, Magnetic Bistability, and Catalysis. Z. Anorg. Allg. Chem..

[4] Maity R., Sarkar B. (2022). Chemistry of Compounds
Based on 1,2,3-Triazolylidene-Type
Mesoionic Carbenes. JACS Au.

[5] Stroek W., Albrecht M. (2024). Application of first-row
transition metal complexes
bearing 1,2,3-triazolylidene ligands in catalysis and beyond. Chem. Soc. Rev..

[6] Vivancos Á., Segarra C., Albrecht M. (2018). Mesoionic
and Related Less Heteroatom-Stabilized
N-Heterocyclic Carbene Complexes: Synthesis, Catalysis, and Other
Applications. Chem. Rev..

[7] Dong Z., Blaskovits J. T., Fadaei-Tirani F., Scopelliti R., Sienkiewicz A., Corminboeuf C., Severin K. (2021). Tuning the π-Accepting
Properties of Mesoionic Carbenes: A Combined Computational and Experimental
Study. Chem. - Eur. J..

[8] Advances in Organometallic Chemistry; Elsevier, 2025.

[9] Fosu, E. ; Cecil, A. C. ; Hollis, T. K. Organometallic pincer complexes with abnormal, mesoionic, or remote N-heterocyclic carbenes: A review of scope and synthetic methods. In Advances in Organometallic Chemistry; Elsevier, 2025; pp 57–127 10.1016/bs.adomc.2025.09.002.

[10] Albrecht, M. Normal and Abnormal N-Heterocyclic Carbene Ligands. In Advances in Organometallic Chemistry; Elsevier, 2014; pp 111–158 10.1016/B978-0-12-800976-5.00002-3.

[11] Gründemann S., Kovacevic A., Albrecht M., Faller J. W., Crabtree R. H. (2001). Abnormal
binding in a carbene complex formed from an imidazolium salt and a
metal hydride complex. Chem. Commun..

[12] Aldeco-Perez E., Rosenthal A. J., Donnadieu B., Parameswaran P., Frenking G., Bertrand G. (2009). Isolation of a C5-deprotonated imidazolium,
a crystalline ″abnormal″ N-heterocyclic carbene. Science.

[13] Albrecht M. (2008). C4-bound imidazolylidenes:
from curiosities to high-impact carbene ligands. Chem. Commun..

[14] Krüger A., Kluser E., Müller-Bunz H., Neels A., Albrecht M. (2012). Chelating
C4-Bound Imidazolylidene Complexes through Oxidative Addition of Imidazolium
Salts to Palladium(0). Eur. J. Inorg. Chem..

[15] Albrecht M. (2009). Abnormal Carbenes
as Ligands in Transition Metal Chemistry: Curiosities with Exciting
Perspectives. Chimia.

[16] Bates J. I., Kennepohl P., Gates D. P. (2009). Abnormal reactivity of an N-heterocyclic
carbene (NHC) with a phosphaalkene: a route to a 4-phosphino-substituted
NHC. Angew. Chem., Int. Ed..

[17] Gope B., Gautam N., Dutta S., Maji S., Mandal S. K. (2025). Coordination
Chemistry of Phosphinidene Stabilized by Mesoionic N-heterocyclic
Carbene and Catalysis. Chem. - Eur. J..

[18] Mandal P., Logdi R., Maji S., Mandal S. K., Tiwari A. K. (2025). Quantum
Chemical Insights of Mesoionic N -Heterocyclic Olefin-Catalyzed S
-Formylation of Thiols Using CO 2. ACS Catal..

[19] Maji S., Gope B., Sharma M., Das A., Jose A., Biswas A., Bhattacharyya K., Mandal S. K. (2025). Independent LUMO
Reactivity in Mesoionic N-Heterocyclic Thiones: Synthesis of a Stable
Radical Anion. Angew. Chem., Int. Ed..

[20] Weber L., Ebeler F., Ghadwal R. S. (2022). Advances and recent trends in dipnictenes
chemistry. Coord. Chem. Rev..

[21] Ghadwal R. S. (2023). 1,3-Imidazole-Based
Mesoionic Carbenes and Anionic Dicarbenes: Pushing the Limit of Classical
N-Heterocyclic Carbenes. Angew. Chem., Int.
Ed..

[22] Ebeler F., Vishnevskiy Y. V., Lamm J.-H., Neumann B., Stammler H.-G., Ghadwal R. S. (2025). Bicyclo1.1.0tetragermane-2,4-diide Diradicaloid. Angew. Chem., Int. Ed..

[23] Ebeler F., Vishnevskiy Y. V., Neumann B., Stammler H.-G., Szczepanik D. W., Ghadwal R. S. (2024). Annulated 1,4-Disilabenzene-1,4-diide and Dihydrogen
Splitting. J. Am. Chem. Soc..

[24] Merschel A., Vishnevskiy Y. V., Neumann B., Stammler H.-G., Ghadwal R. S. (2024). Boosting
the π-Acceptor Property of Mesoionic Carbenes by Carbonylation
with Carbon Monoxide. Angew. Chem., Int. Ed..

[25] Steffenfauseweh H., Vishnevskiy Y. V., Neumann B., Stammler H.-G., Snabilié D. D., Bruin B. de., Ghadwal R. S. (2025). Annulated Radical Cations with a
C4E2-Core (E = P, As, Sb): Stable Pnictogen Analogs of Elusive Aryl
Radical Anions of Birch Reduction Reactions. Angew. Chem., Int. Ed..

[26] Gautam N., P S., Soni K., Chakraborty S., Maji S., Bhattacharyya K., K Mandal S. (2025). aNHC-Stabilized Low-Valent Phosphorus Compound: Exploring
Dual Catalytic Activity via Nucleophilicity and P­(I)/P­(III) Redox
Reactivity. J. Am. Chem. Soc..

[27] Ho N. K. T., Reichmann S. O., Rottschäfer D., Herbst-Irmer R., Ghadwal R. S. (2017). Expanding the Scope
of Cu­(I) Catalyzed “Click
Chemistry” with Abnormal NHCs: Three-Fold Click to Tris-Triazoles. Catalysts.

[28] Bellotti P., Koy M., Hopkinson M. N., Glorius F. (2021). Recent advances in the chemistry
and applications of N-heterocyclic carbenes. Nat. Rev. Chem..

[29] Hopkinson M. N., Richter C., Schedler M., Glorius F. (2014). An overview of N-heterocyclic
carbenes. Nature.

[30] Guisado-Barrios G., Soleilhavoup M., Bertrand G. (2018). 1 H-1,2,3-Triazol-5-ylidenes: Readily
Available Mesoionic Carbenes. Acc. Chem. Res..

[31] Mathew P., Neels A., Albrecht M. (2008). 1,2,3-Triazolylidenes as versatile
abnormal carbene ligands for late transition metals. J. Am. Chem. Soc..

[32] Guisado-Barrios G., Bouffard J., Donnadieu B., Bertrand G. (2010). Crystalline 1H-1,2,3-triazol-5-ylidenes:
new stable mesoionic carbenes (MICs). Angew.
Chem., Int. Ed..

[33] Pavun A., Niess R., Scheibel L. A., Seidl M., Hohloch S. (2024). A mesoionic
carbene stabilized nickel­(II) hydroxide complex: a facile precursor
for C-H activation chemistry. Dalton Trans..

[34] Wittwer B., Leitner D., Neururer F. R., Schoch R., Seidl M., Pecak J., Podewitz M., Hohloch S. (2024). Scrutinizing the redox
chemistry of group 10 complexes supported by a redox-active bis-phenolate
mesoionic carbene. Polyhedron.

[35] Watt F. A., Sieland B., Dickmann N., Schoch R., Herbst-Irmer R., Ott H., Paradies J., Kuckling D., Hohloch S. (2021). Coupling of CO2 and
epoxides catalysed by novel N-fused mesoionic carbene complexes of
nickel­(II). Dalton Trans..

[36] Vanicek S., Beerhues J., Bens T., Levchenko V., Wurst K., Bildstein B., Tilset M., Sarkar B. (2019). Oxidative
access via aqua regia to an electrophilic, mesoionic dicobaltoceniumyltriazolylidene
gold­(III) catalyst. Organometallics.

[37] Poulain A., Canseco-Gonzalez D., Hynes-Roche R., Müller-Bunz H., Schuster O., Stoeckli-Evans H., Neels A., Albrecht M. (2011). Synthesis
and Tunability of Abnormal 1,2,3-Triazolylidene Palladium and Rhodium
Complexes. Organometallics.

[38] Vanicek S., Podewitz M., Stubbe J., Schulze D., Kopacka H., Wurst K., Müller T., Lippmann P., Haslinger S., Schottenberger H., Liedl K. R., Ott I., Sarkar B., Bildstein B. (2018). Highly Electrophilic,
Catalytically Active and Redox-Responsive
Cobaltoceniumyl and Ferrocenyl Triazolylidene Coinage Metal Complexes. Chem. - Eur. J..

[39] Abdellaoui M., Oppel K., Vianna A., Soleilhavoup M., Yan X., Melaimi M., Bertrand G. (2024). 1H-1,2,3-Triazol-5-ylidenes as Catalytic
Organic Single-Electron Reductants. J. Am. Chem.
Soc..

[40] Liu W., Vianna A., Zhang Z., Huang S., Huang L., Melaimi M., Bertrand G., Yan X. (2021). Mesoionic carbene-Breslow
intermediates as super electron donors: Application to the metal-free
arylacylation of alkenes. Chem. Catal..

[41] Mulks F. F., Melaimi M., Yan X., Baik M.-H., Bertrand G. (2023). How To Enhance
the Efficiency of Breslow Intermediates for SET Catalysis. J. Org. Chem..

[42] Hohloch S., Suntrup L., Sarkar B. (2016). Exploring
potential cooperative effects
in dicopper­(i)-di-mesoionic carbene complexes: applications in click
catalysis. Inorg. Chem. Front..

[43] Hohloch S., Frey W., Su C.-Y., Sarkar B. (2013). Abnormal carbenes derived
from the 1,5-cycloaddition product between azides and alkynes: structural
characterization of Pd­(II) complexes and their catalytic properties. Dalton Trans..

[44] Hohloch S., Scheiffele D., Sarkar B. (2013). Activating Azides and Alkynes for
the Click Reaction with [Cu­(a NHC) 2 I] or [Cu­(a NHC) 2] + (a NHC
= Triazole-Derived Abnormal Carbenes): Structural Characterization
and Catalytic Properties. Eur. J. Inorg. Chem..

[45] Suntrup L., Hohloch S., Sarkar B. (2016). Expanding the Scope of Chelating
Triazolylidenes: Mesoionic Carbenes from the 1,5-″Click″-Regioisomer
and Catalytic Synthesis of Secondary Amines from Nitroarenes. Chem. - Eur. J..

[46] Suntrup L., Stein F., Klein J., Wilting A., Parlane F. G. L., Brown C. M., Fiedler J., Berlinguette C. P., Siewert I., Sarkar B. (2020). Rhenium Complexes of Pyridyl-Mesoionic
Carbenes: Photochemical Properties and Electrocatalytic CO2 Reduction. Inorg. Chem..

[47] van
der Meer M., Glais E., Siewert I., Sarkar B. (2015). Electrocatalytic
Dihydrogen Production with a Robust Mesoionic Pyridylcarbene Cobalt
Catalyst. Angew. Chem., Int. Ed..

[48] Klenk S., Rupf S., Suntrup L., van der Meer M., Sarkar B. (2017). The Power of Ferrocene, Mesoionic Carbenes, and Gold:
Redox-Switchable Catalysis. Organometallics.

[49] Hohloch S., Suntrup L., Sarkar B. (2013). Arene–Ruthenium­(II) and –
Iridium­(III) Complexes with “Click”-Based Pyridyl-triazoles,
Bis-triazoles, and Chelating Abnormal Carbenes: Applications in Catalytic
Transfer Hydrogenation of Nitrobenzene. Organometallics.

[50] Hettmanczyk L., Suntrup L., Klenk S., Hoyer C., Sarkar B. (2017). Heteromultimetallic
Complexes with Redox-Active Mesoionic Carbenes: Control of Donor Properties
and Redox-Induced Catalysis. Chem. - Eur. J..

[51] Stroek W., Keilwerth M., Pividori D. M., Meyer K., Albrecht M. (2021). An Iron-Mesoionic
Carbene Complex for Catalytic Intramolecular C-H Amination Utilizing
Organic Azides. J. Am. Chem. Soc..

[52] Canseco-Gonzalez D., Gniewek A., Szulmanowicz M., Müller-Bunz H., Trzeciak A. M., Albrecht M. (2012). PEPPSI-type palladium complexes containing
basic 1,2,3-triazolylidene ligands and their role in Suzuki-Miyaura
catalysis. Chem. - Eur. J..

[53] Canseco-Gonzalez D., Petronilho A., Mueller-Bunz H., Ohmatsu K., Ooi T., Albrecht M. (2013). Carbene transfer from
triazolylidene gold complexes
as a potent strategy for inducing high catalytic activity. J. Am. Chem. Soc..

[54] Delgado-Rebollo M., Canseco-Gonzalez D., Hollering M., Mueller-Bunz H., Albrecht M. (2014). Synthesis and catalytic
alcohol oxidation and ketone
transfer hydrogenation activity of donor-functionalized mesoionic
triazolylidene ruthenium­(II) complexes. Dalton
Trans..

[55] Wei Y., Liu S.-X., Mueller-Bunz H., Albrecht M. (2016). Synthesis of Triazolylidene
Nickel Complexes and Their Catalytic Application in Selective Aldehyde
Hydrosilylation. ACS Catal..

[56] Knörr P., Lentz N., Albrecht M. (2023). Immobilization
of Iridium Triazolylidene
Complexes into Polymer Scaffolds and Their Application in Water Oxidation. Eur. J. Inorg. Chem..

[57] Mejuto C., Guisado-Barrios G., Gusev D., Peris E. (2015). First homoleptic MIC
and heteroleptic NHC-MIC coordination cages from 1,3,5-triphenylbenzene-bridged
tris-MIC and tris-NHC ligands. Chem. Commun..

[58] AL-Shnani F., Guisado-Barrios G., Sainz D., Peris E. (2019). Tris-triazolium Salts
as Anion Receptors and as Precursors for the Preparation of Cylinder-like
Coordination Cages. Organometallics.

[59] Stubbe J., Neuman N. I., McLellan R., Sommer M. G., Nößler M., Beerhues J., Mulvey R. E., Sarkar B. (2021). Isomerization Reactions
in Anionic Mesoionic Carbene-Borates and Control of Properties and
Reactivities in the Resulting CoII Complexes through Agostic Interactions. Angew. Chem., Int. Ed..

[60] Hettmanczyk L., Spall S. J. P., Klenk S., van der Meer M., Hohloch S., Weinstein J. A., Sarkar B. (2017). Structural, Electrochemical,
and Photochemical Properties of Mono- and Digold­(I) Complexes Containing
Mesoionic Carbenes. Eur. J. Inorg. Chem..

[61] Dierks P., Kruse A., Bokareva O. S., Al-Marri M. J., Kalmbach J., Baltrun M., Neuba A., Schoch R., Hohloch S., Heinze K., Seitz M., Kühn O., Lochbrunner S., Bauer M. (2021). Distinct photodynamics of κ-N
and κ-C pseudoisomeric iron­(II) complexes. Chem. Commun..

[62] Yaltseva, P. ; Wittwer, B. ; Leitner, D. ; Neururer, F. R. ; Tambornino, F. ; Schmidt, A. ; Munz, D. ; Wenger, O. S. ; Hohloch, S. Interplay between ligand field strength and the nephelauxetic effect in chromium­(iii) complexes with anionic amido ligands Chem. Sci. 2026 10.1039/D5SC09069E. PMC12984063 41836535

[63] Liu Y., Kjaer K. S., Fredin L. A., Chábera P., Harlang T., Canton S. E., Lidin S., Zhang J., Lomoth R., Bergquist K.-E., Persson P., Wärnmark K., Sundström V. (2015). A heteroleptic ferrous complex with mesoionic bis­(1,2,3-triazol-5-ylidene)
ligands: taming the MLCT excited state of iron­(II). Chem. - Eur. J..

[64] White, M. ; Yao, Z. ; Scattergood, P. ; Ross, D. ; Dixon, I. ; Yartsev, A. ; Persson, P. ; Elliott, P. A Cyclometalated Cobalt­(III) Triazolylidene Photosensitiser Complex Exhibiting Enhanced Light-Harvesting and Low Temperature 3MC State Luminescence ChemRxiv 2025 10.26434/chemrxiv-2025-8v9r2.

[65] Chábera P., Liu Y., Prakash O., Thyrhaug E., Nahhas A. E., Honarfar A., Essén S., Fredin L. A., Harlang T. C. B., Kjær K. S., Handrup K., Ericson F., Tatsuno H., Morgan K., Schnadt J., Häggström L., Ericsson T., Sobkowiak A., Lidin S., Huang P., Styring S., Uhlig J., Bendix J., Lomoth R., Sundström V., Persson P., Wärnmark K. (2017). A low-spin Fe­(iii) complex with 100-ps
ligand-to-metal charge transfer photoluminescence. Nature.

[66] Boden P., Di Martino-Fumo P., Bens T., Steiger S., Albold U., Niedner-Schatteburg G., Gerhards M., Sarkar B. (2021). NIR-Emissive Chromium(0),
Molybdenum(0), and Tungsten(0) Complexes in the Solid State at Room
Temperature. Chem. - Eur. J..

[67] Bens T., Marhöfer D., Boden P., Steiger S. T., Suntrup L., Niedner-Schatteburg G., Sarkar B. (2023). A Different Perspective
on Tuning
the Photophysical and Photochemical Properties: The Influence of Constitutional
Isomers in Group 6 Carbonyl Complexes with Pyridyl-Mesoionic Carbenes. Inorg. Chem..

[68] Boden P. J., Di Martino-Fumo P., Bens T., Steiger S. T., Marhöfer D., Niedner-Schatteburg G., Sarkar B. (2022). Mechanistic and Kinetic Investigations
of ON/OFF (Photo)­Switchable Binding of Carbon Monoxide by Chromium(0),
Molybdenum(0) and Tungsten(0) Carbonyl Complexes with a Pyridyl-Mesoionic
Carbene Ligand. Chem. - Eur. J..

[69] Breugst M., Reißig H.-U. (2020). Die Huisgen-Reaktion: Meilensteine
der 1,3-dipolaren
Cycloaddition. Angew. Chem..

[70] Binder W., Kluger C. (2006). Azide/Alkyne-“Click”
Reactions: Applications
in Material Science and Organic Synthesis. Curr.
Org. Chem..

[71] Tornøe C. W., Christensen C., Meldal M. (2002). Peptidotriazoles on
solid phase:
1,2,3-triazoles by regiospecific copper­(i)-catalyzed 1,3-dipolar cycloadditions
of terminal alkynes to azides. J. Org. Chem..

[72] Rostovtsev V. V., Green L. G., Fokin V. V., Sharpless K. B. (2002). A Stepwise
Huisgen Cycloaddition Process: Copper­(I)-Catalyzed Regioselective
“Ligation” of Azides and Terminal Alkynes. Angew. Chem., Int. Ed..

[73] Johansson J. R., Beke-Somfai T., Said Stålsmeden A., Kann N. (2016). Ruthenium-Catalyzed
Azide Alkyne Cycloaddition Reaction: Scope, Mechanism, and Applications. Chem. Rev..

[74] Kwok S. W., Fotsing J. R., Fraser R. J., Rodionov V. O., Fokin V. V. (2010). Transition-metal-free
catalytic synthesis of 1,5-diaryl-1,2,3-triazoles. Org. Lett..

[75] Oakdale J. S., Fokin V. V., Umezaki S., Fukuyama T. (2013). Preparation of 1,5-Disubstituted
1,2,3-Triazoles via Ruthenium-catalyzed Azide Alkyne Cycloaddition. Org. Synth..

[76] Bouffard J., Keitz B. K., Tonner R., Guisado-Barrios G., Frenking G., Grubbs R. H., Bertrand G. (2011). Synthesis
of Highly
Stable 1,3-Diaryl-1H-1,2,3-triazol-5-ylidenes and their Applications
in Ruthenium-Catalyzed Olefin Metathesis. Organometallics.

[77] Keitz B. K., Bouffard J., Bertrand G., Grubbs R. H. (2011). Protonolysis of
a ruthenium-carbene bond and applications in olefin metathesis. J. Am. Chem. Soc..

[78] Flores-Jarillo M., Mendoza-Espinosa D., Salazar-Pereda V., González-Montiel S. (2017). Synthesis
and Catalytic Benefits of Tetranuclear Gold­(I) Complexes with a C4
-Symmetric Tetratriazol-5-ylidene. Organometallics.

[79] Hettmanczyk L., Schmid B., Hohloch S., Sarkar B. (2016). Palladium­(ii)-Acetylacetonato
Complexes with Mesoionic Carbenes: Synthesis, Structures and Their
Application in the Suzuki-Miyaura Cross Coupling Reaction. Molecules.

[80] Hohloch S., Duecker F. L., van der Meer M., Sarkar B. (2015). Copper­(I) complexes
of mesoionic carbene: structural characterization and catalytic hydrosilylation
reactions. Molecules.

[81] Guisado-Barrios G., Bouffard J., Donnadieu B., Bertrand G. (2011). Bis­(1,2,3-triazol-5-ylidenes)
(i-bitz) as Stable 1,4-Bidentate Ligands Based on Mesoionic Carbenes
(MICs). Organometallics.

[82] Mendoza-Espinosa D., Alvarez-Hernández A., Angeles-Beltrán D., Negrón-Silva G. E., Suárez-Castillo O. R., Vásquez-Pérez J. M. (2017). Bridged
N-Heterocyclic/Mesoionic
(NHC/MIC) Heterodicarbenes as Ligands for Transition Metal Complexes. Inorg. Chem..

[83] Suntrup L., Klenk S., Klein J., Sobottka S., Sarkar B. (2017). Gauging Donor/Acceptor
Properties and Redox Stability of Chelating Click-Derived Triazoles
and Triazolylidenes: A Case Study with Rhenium­(I) Complexes. Inorg. Chem..

[84] Baltrun M., Watt F. A., Schoch R., Wölper C., Neuba A. G., Hohloch S. (2019). A new bis-phenolate
mesoionic carbene
ligand for early transition metal chemistry. Dalton Trans..

[85] Bezuidenhout D. I., Kleinhans G., Guisado-Barrios G., Liles D. C., Ung G., Bertrand G. (2014). Isolation
of a potassium bis­(1,2,3-triazol-5-ylidene)­carbazolide:
a stabilizing pincer ligand for reactive late transition metal complexes. Chem. Commun..

[86] Pinter P., Schüßlbauer C. M., Watt F. A., Dickmann N., Herbst-Irmer R., Morgenstern B., Grünwald A., Ullrich T., Zimmer M., Hohloch S., Guldi D. M., Munz D. (2021). Bright luminescent
lithium and magnesium carbene complexes. Chem.
Sci..

[87] Ang Z. Z., Laxmi S., León F., Kooij J. E. M., García F., England J. (2021). Mechanochemical Synthesis of Tripodal Tris4-(1,2,3-triazol-5-ylidene)­methylamine
Mesoionic Carbene Ligands and Their Complexation with Silver­(I). Inorg. Chem..

[88] Rudolf R., Todorovski A., Lederer V., Neuman N. I., Schubert H., Sarkar B. (2025). An Anionic
Mesoionic Carbene (anMIC) and its Transformation
to Metallo MIC-Boranes: Synthesis and Properties. Angew. Chem., Int. Ed..

[89] Kleinhans G., Guisado-Barrios G., Liles D. C., Bertrand G., Bezuidenhout D. I. A. (2016). rhodium­(I)-oxygen
adduct as a selective catalyst for one-pot sequential alkyne dimerization-hydrothiolation
tandem reactions. Chem. Commun..

[90] Kleinhans G., Hansmann M. M., Guisado-Barrios G., Liles D. C., Bertrand G., Bezuidenhout D. I. (2016). Nucleophilic
T-Shaped (LXL)­Au­(I)-Pincer Complexes:
Protonation and Alkylation. J. Am. Chem. Soc..

[91] Frey J., Azar S., Dagorne S., Bellemin-Laponnaz S. (2026). Bis­(phenolate)
N-Heterocyclic Carbene [OCO] Pincer Ligands: A Unique Family for the
Stabilization of Transition Metal Centers and Main Group Elements. ChemistryEurope.

[92] Neururer F. R., Huter K., Seidl M., Hohloch S. (2023). Reactivity and Structure
of a Bis-phenolate Niobium NHC Complex. ACS
Org. Inorg. Au.

[93] Neururer F. R., Leitner D., Liu S., Wurst K., Kopacka H., Seidl M., Hohloch S. (2023). The Chemistry of Vanadium
bis-Phenolate
NHC Complexes in Three Oxidation States. Eur.
J. Inorg. Chem..

[94] Baltrun M., Watt F. A., Schoch R., Hohloch S. (2019). Dioxo-, Oxo-imido-,
and Bis-imido-Molybdenum­(VI) Complexes with a Bis-phenolate-NHC Ligand. Organometallics.

[95] Neururer F. R., Liu S., Leitner D., Baltrun M., Fisher K. R., Kopacka H., Wurst K., Daumann L. J., Munz D., Hohloch S. (2021). Mesoionic
Carbenes in Low- to High-Valent Vanadium Chemistry. Inorg. Chem..

[96] Rigoni G., Nylund P. V. S., Albrecht M. (2023). Manganese­(III)
complexes stabilized
with N-heterocyclic carbene ligands for alcohol oxidation catalysis. Dalton Trans..

[97] Neururer F. R., Heim F., Baltrun M., Boos P., Beerhues J., Seidl M., Hohloch S. (2025). Probing the
Influence of Imidazolylidene-
and Triazolylidene-Based Carbenes on the Catalytic Potential of Dioxomolybdenum
and Dioxotungsten Complexes in Dexoxygenation Catalysis. Inorg. Chem. Front..

[98] Liu S., Amaro-Estrada J. I., Baltrun M., Douair I., Schoch R., Maron L., Hohloch S. (2021). Catalytic Deoxygenation of Nitroarenes
Mediated by High-Valent Molybdenum­(VI)–NHC Complexes. Organometallics.

[99] Leitner D., Neururer F. R., Hohloch S. (2025). Synthesis
and electrochemical properties
of molybdenum nitrido complexes supported by redox-active NHC and
MIC ligands. Dalton Trans..

[100] Li Z., Liu C., An J., Wang X., Hu S. (2024). Catalytic
Dinitrogen Reduction to Silylamines by Molybdenum Nitride Complexes
Bearing a Diphenolate N-Heterocyclic Carbene Ligand. ACS Catal..

[101] Garcia B., Royo B. (2024). Molybdenum Catalyzed
Acceptorless
Dehydrogenation of Alcohols for the Synthesis of Quinolines. ChemCatChem.

[102] Elvers B. J., Schulan P., Pätsch S., Fischer C., Schulzke C. (2022). Combining a Low Valent Molybdenum(0)
Center with a Strongly σ-Donating Mesoionic Carbene Chelate
Ligand—Synthesis and Structural Characterization. Inorganics.

[103] Garcia B., Batuecas M., Royo B. (2026). Cooperative Reactivity
of Methylene-Linked Bis-Triazolylidene Ligands in Tungsten-Catalyzed
Alcohol Dehydrogenation. ChemistryEurope.

[104] Beerhues J., Sen S., Schowner R., Mate Nagy G., Wang D., Buchmeiser M. R. (2017). Tailored molybdenum imido alkylidene
N -heterocyclic carbene complexes as latent catalysts for the polymerization
of dicyclopentadiene. J. Polym. Sci., Part A:
Polym. Chem..

[105] Bens T., Walter R. R. M., Beerhues J., Schmitt M., Krossing I., Sarkar B. (2023). The Best of Both Worlds: Combining
the Power of MICs and WCAs To Generate Stable and Crystalline CrI
-Tetracarbonyl Complexes with π-Accepting Ligands. Chem. - Eur. J..

[106] Bens T., Sarkar B. (2024). Investigations of the Influence of
Two Pyridyl-Mesoionic Carbene Constitutional Isomers on the Electrochemical
and Spectroelectrochemical Properties of Group 6 Metal Carbonyl Complexes. Inorganics.

[107] Ramnauth R., Al-Juaid S., Motevalli M., Parkin B. C., Sullivan A. C. (2004). Synthesis, structure, and catalytic
oxidation chemistry from the first oxo-imido Schiff base metal complexes. Inorg. Chem..

[108] Hänninen M. M., Paturi P., Tuononen H. M., Sillanpää R., Lehtonen A. (2013). Heptacoordinated molybdenum­(VI)
complexes of phenylenediamine
bis­(phenolate): a stable molybdenum amidophenoxide radical. Inorg. Chem..

[109] Brown S. N. (2012). Metrical oxidation states of 2-amidophenoxide
and catecholate
ligands: structural signatures of metal-ligand π bonding in
potentially noninnocent ligands. Inorg. Chem..

[110] Erickson A. N., Brown S. N. (2018). Molybdenum­(vi)
tris­(amidophenoxide)
complexes. Dalton Trans..

[111] Marshall-Roth T., Brown S. N. (2015). Redox activity and
π bonding
in a tripodal seven-coordinate molybdenum­(VI) tris­(amidophenolate). Dalton Trans..

[112] Marshall-Roth T., Liebscher S. C., Rickert K., Seewald N. J., Oliver A. G., Brown S. N. (2012). Nonclassical
oxygen atom transfer
reactions of oxomolybdenum­(VI) bis­(catecholate). Chem. Commun..

[113] Ranis L. G., Gianino J., Hoffman J. M., Brown S. N. (2021). Nonclassical
oxygen atom transfer reactions of an eight-coordinate dioxomolybdenum­(vi)
complex. Inorg. Chem. Front..

[114] Shekar S., Brown S. N. (2014). Mixed amidophenolate-catecholates
of molybdenum­(VI). Dalton Trans..

[115] Persson C., Andersson C. (1993). Reduction of tungsten­(VI) and molybdenum­(V)
by allyltrimethylsilane and cyclopentene. Simple high yield syntheses
of MoCl4­(OEt2)­2, MoCl4­(dme), WCl4­(thf)­2, WCl4­(dme) and WOCl3­(thf)­2. Inorg. Chim. Acta.

[116] Leitner D., Wurst K., Hohloch S. (2024). A Versatile
Comproportionation
Route to Construct Mono-Imido Trichlorido Complexes of Molybdenum­(V)
and Tungsten­(V). Z. Anorg. Allg. Chem..

[117] Leitner D., Wittwer B., Neururer F. R., Seidl M., Wurst K., Tambornino F., Hohloch S. (2023). Expanding the Utility
of β-Diketiminate Ligands in Heavy Group VI Chemistry of Molybdenum
and Tungsten. Organometallics.

[118] Johansson E. M. J., Odelius M., Plogmaker S., Gorgoi M., Svensson S., Siegbahn H., Rensmo H. (2010). Spin–Orbit
Coupling and Metal–Ligand Interactions in Fe­(II), Ru­(II), and
Os­(II) Complexes. J. Phys. Chem. C.

[119] Soriaga R.
A. D., Nguyen J. M., Albright T. A., Hoffman D. M. (2010). Diamagnetic
group 6 tetrakis­(di-tert-butylketimido)­metal­(IV) complexes. J. Am. Chem. Soc..

[120] Yang T., Liu X., Fang J., Liu Z., Qiao Z., Zhu Z., Cheng Q., Zhang Y., Chen X. (2024). Tuning d-orbitals to control spin-orbit coupling in terminated MXenes. Phys. Chem. Chem. Phys..

[121] Zhang Y., Qiao S.-Q., Wu C.-L., Ji Z.-Q., Wu H., Li F. (2025). Strong spin-orbit coupling
effect induced large valley
splitting in Janus MSeXH (M = Cr, Mo, and W; X = N and P). Phys. Chem. Chem. Phys..

[122] Jayarathne U., Chandrasekaran P., Jacobsen H., Mague J. T., Donahue J. P. (2010). A tungsten-mediated
closed cycle of reactivity for
the reduction of CO(2) to CO. Dalton Trans..

[123] Ćorović M. Z., Belaj F., Mösch-Zanetti N. C. (2023). Dioxygen
Activation by a Bioinspired Tungsten­(IV) Complex. Inorg. Chem..

[124] Cotton F. A., Vidyasagar K. (1995). Complexes of molybdenum­(III) and
-(IV) with chloride and tertiary phosphine ligands; an omniumgatherum
of new and old results. Polyhedron.

[125] Ehweiner M. A., Belaj F., Kirchner K., Mösch-Zanetti N. C. (2021). Synthesis
and Reactivity of a Bioinspired Molybdenum­(IV) Acetylene Complex. Organometallics.

[126] Manojlović-Muir L. (1976). Crystal and molecular
structure of
tetrachlorotris­(dimethylphenylphosphine)­molybdenum­(IV)–ethanol. J. Chem. Soc., Dalton Trans..

[127] Rogers R. D., Carmona E., Galindo A., Atwood J. L., Canada L. G. (1984). Trimethylphosphine complexes of molybdenum and tungsten.
The synthesis and chemical properties of MoCl4­(PMe3)­3 and the crystal
and molecular structures of WCl4­(PMe3)­3 and MoO­(acac)­2PMe3. J. Organomet. Chem..

[128] Leppin J., Förster C., Heinze K. (2014). Molybdenum complex
with bulky chelates as a functional model for molybdenum oxidases. Inorg. Chem..

[129] Heinze K. (2015). Bioinspired functional analogs of
the active site of
molybdenum enzymes: Intermediates and mechanisms. Coord. Chem. Rev..

[130] Sheldrick G. M. (2015). SHELXT - integrated space-group and crystal-structure
determination. Acta Crystallogr., Sect. A: Found.
Adv..

[131] Dolomanov O. V., Bourhis L. J., Gildea R. J., Howard J. A. K., Puschmann H. (2009). OLEX2: a complete structure solution,
refinement and
analysis program. J. Appl. Crystallogr..

[132] Sheldrick G. M. (2015). Crystal
structure refinement with SHELXL. Acta Crystallogr.,
Sect. C: Struct. Chem..

[133] Spek A. L. (2015). PLATON SQUEEZE: a tool for the calculation of the disordered
solvent contribution to the calculated structure factors. Acta Crystallogr., Sect. C: Struct. Chem..

