# Supplementary material for: Stable in Four Oxidation States: Exploring the Redox-Variability of Molybdenum and Tungsten Triazolylidene Complexes
Source: Inorg Chem. 2026 May 28;65(23):12921–34. doi: 10.1021/acs.inorgchem.6c00876 (PMC13273806; doi:10.1021/acs.inorgchem.6c00876)
Supplement: Supplementary file 1 [file ic6c00876_si_001.pdf]

Supporting Information

Stable in Four Oxidation States: Exploring the Redox-Variability of Molybdenum and Tungsten Triazolylidene Complexes

Florian R. Neururer,[a] Florian Heim,[a] Lena Gschnell,[a] Daniel Leitner,[a] Michael Seidl,[a] Alexander Pöthig,[b] Stephan Hohloch,*[a]

[a] Leopold-Franzens-University Innsbruck, Faculty of Chemistry and Pharmacy, Institute of General, Inorganic and Theoretical Chemistry, Innrain 80-82, 6020 Innsbruck, Austria, E-Mail: Stephan.Hohloch@uibk.ac.at

[b] Catalysis Research Center (CRC) & TUM School of Natural Sciences, Department of Chemistry, Technical University of Munich, Ernst-Otto-Fischer Str. 1, 85747 Garching, Germany

1. NMR spectra 2
2. IR spectroscopy 34
3. UV-Vis-NIR spectra 41
4. EPR spectra 51
5. Cyclic voltammetry 53
6. Crystallographic details 54

1. NMR spectra

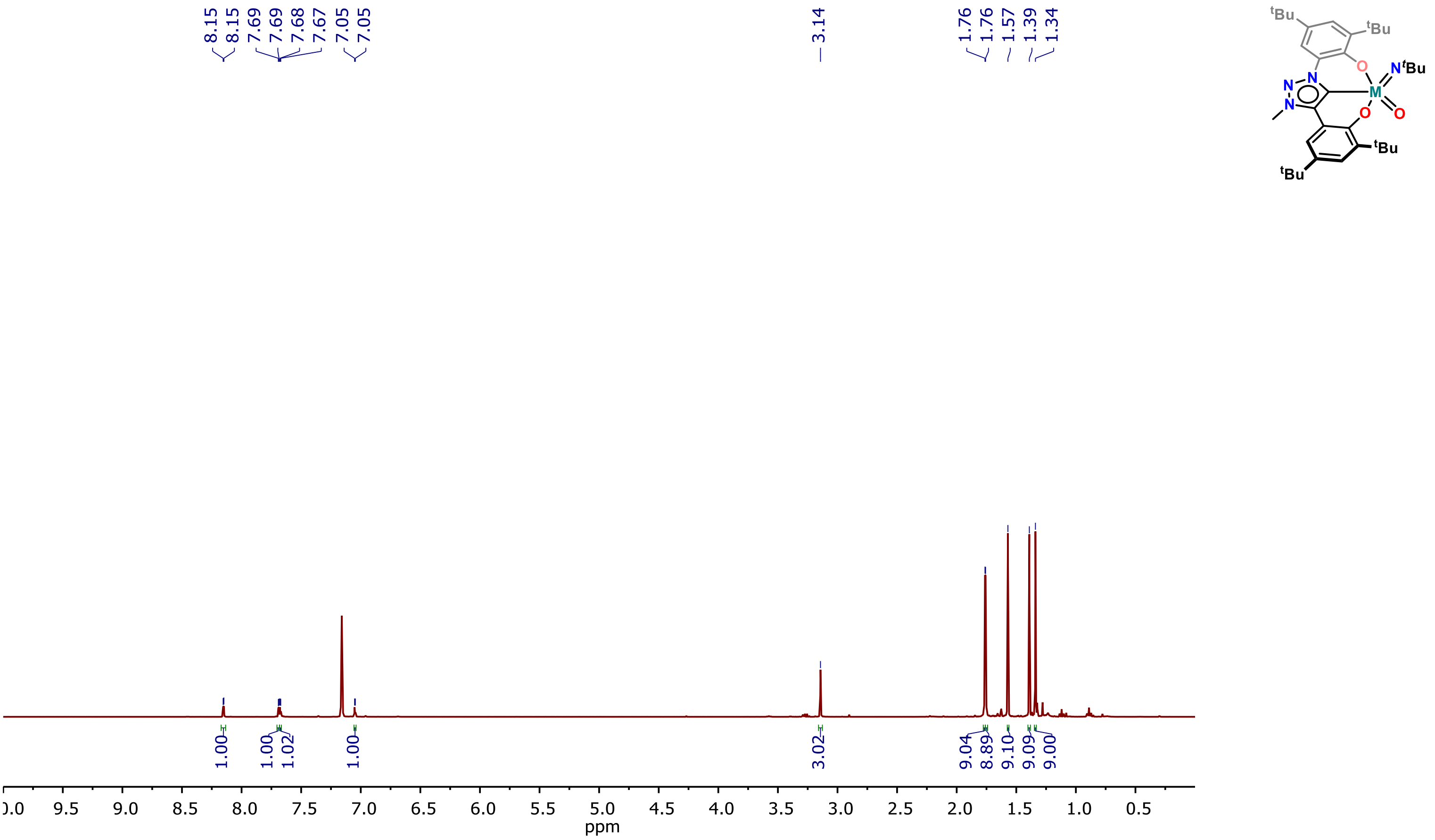

Figure S 1: ^{1}H NMR of **2-Mo** in C_6D_6 at 298K.

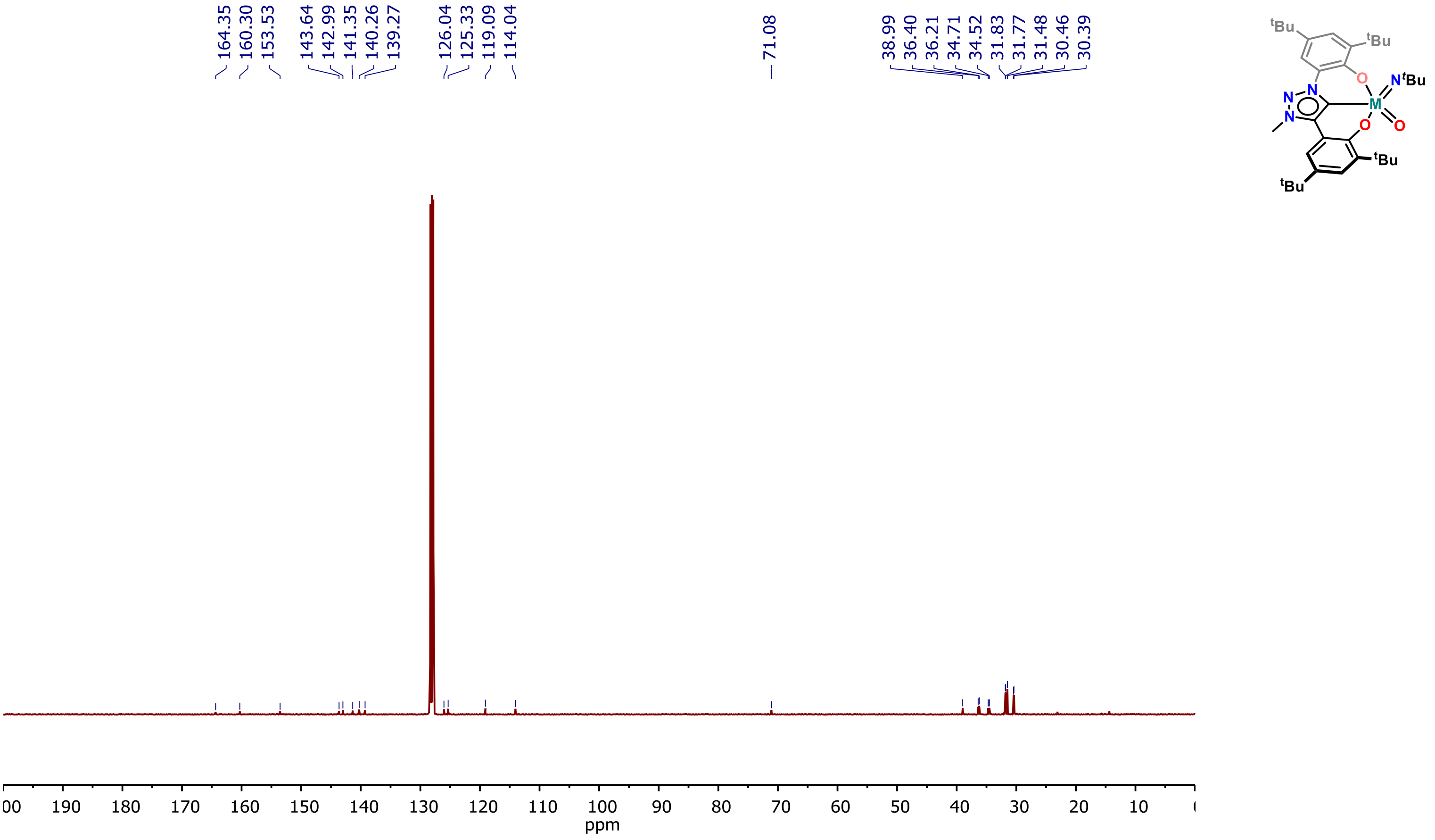

Figure S 2: ^{13}C NMR of **2-Mo** in C_6D_6 at 298K.

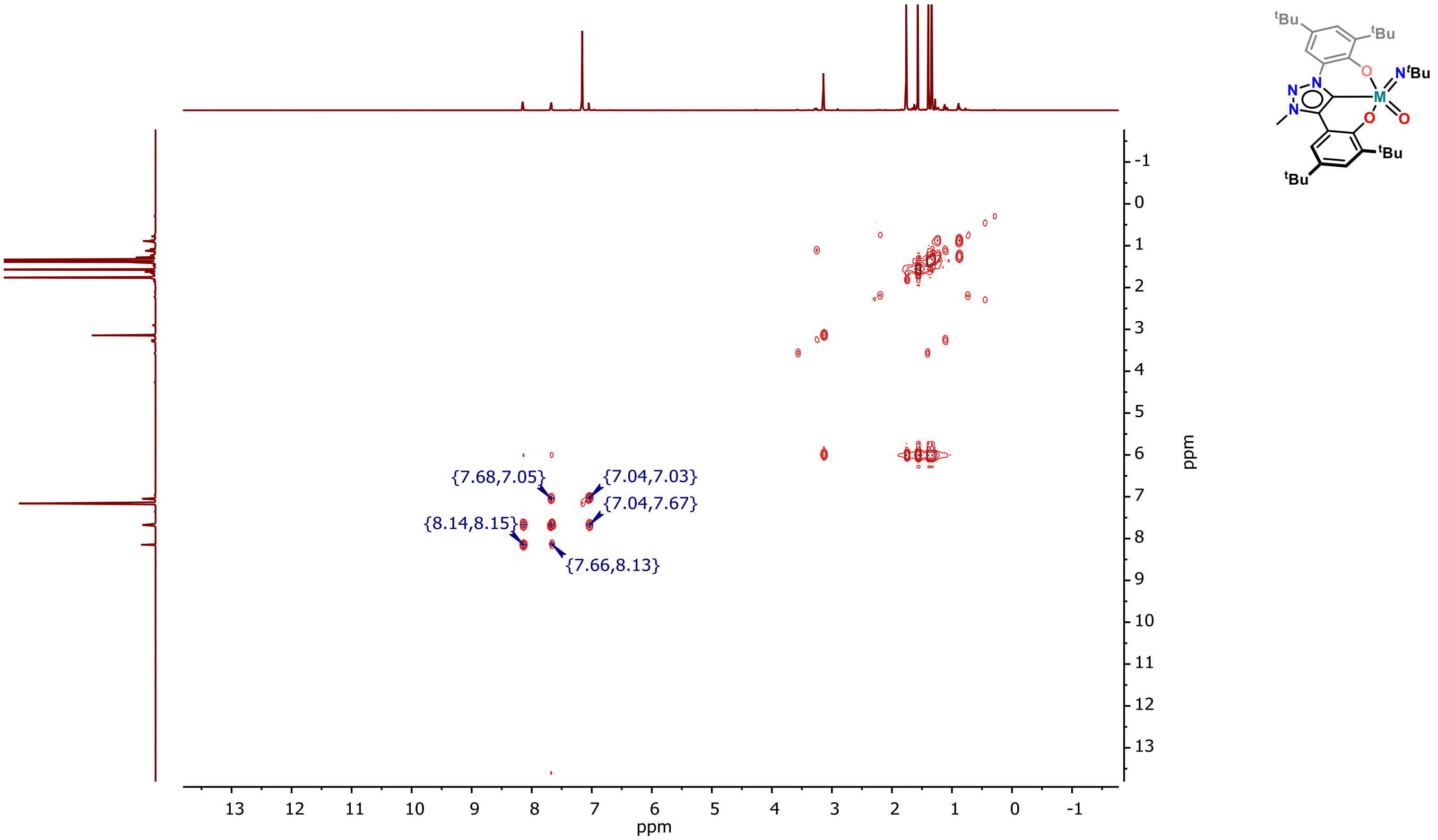

Figure S 3: ^{1}H-^{1}H COSY NMR of **2-Mo** in C_6D_6 at 298K.

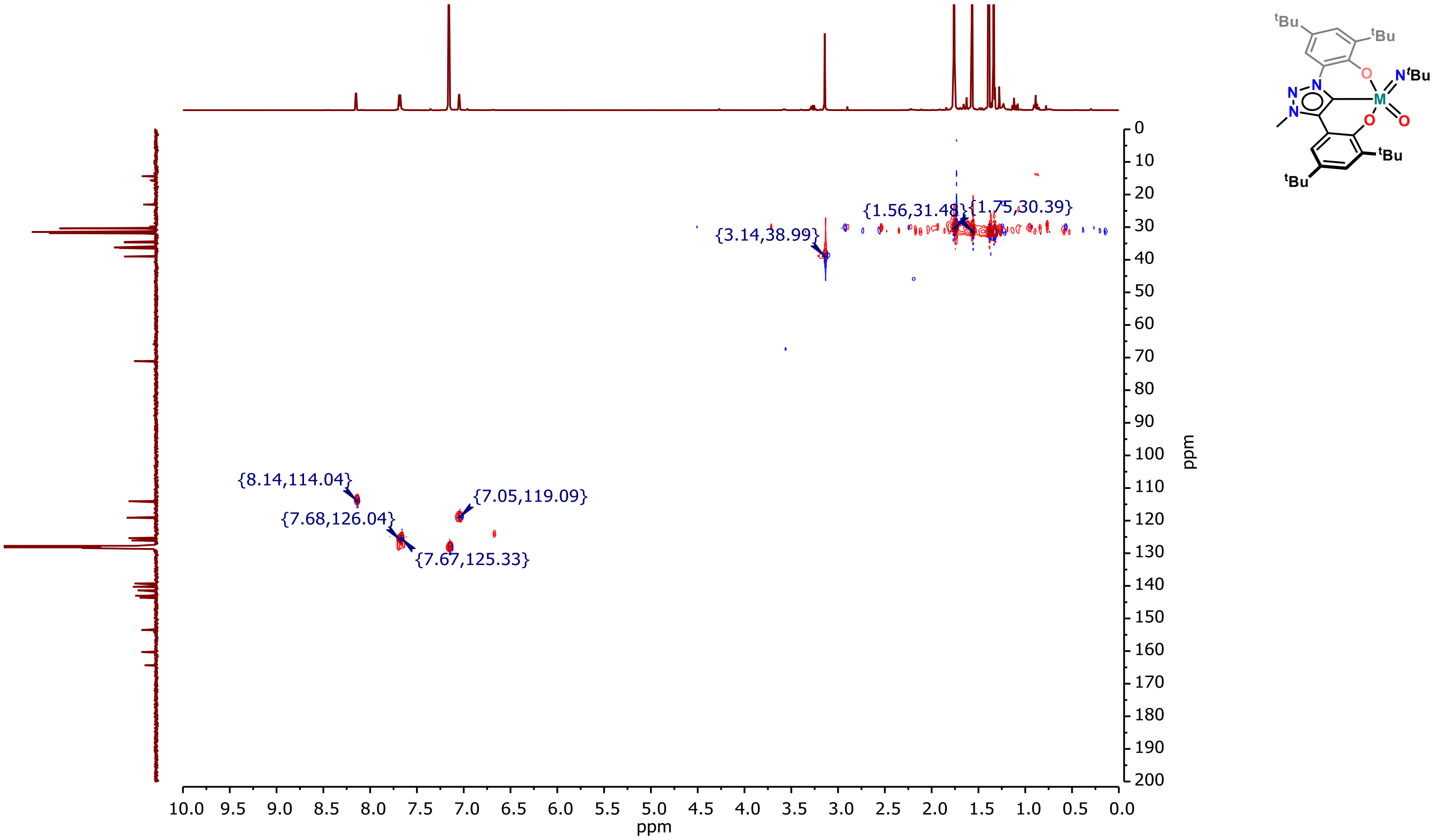

Figure S 4: ^{1}H-^{13}C HSQC NMR of **2-Mo** in C_6D_6 at 298K.

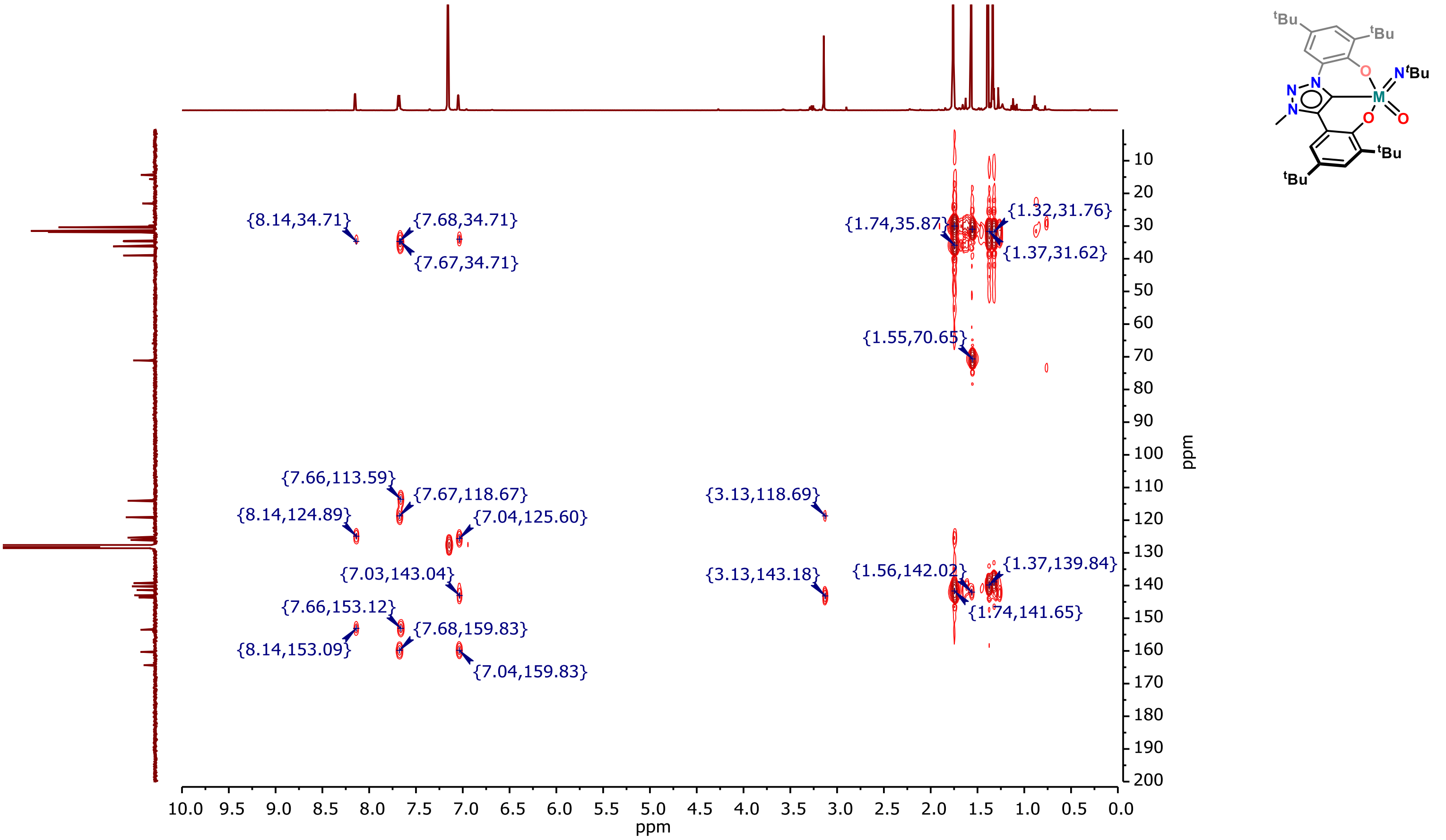

Figure S 5: ^{1}H-^{13}C HMBC NMR of **2-Mo** in C_6D_6 at 298K.

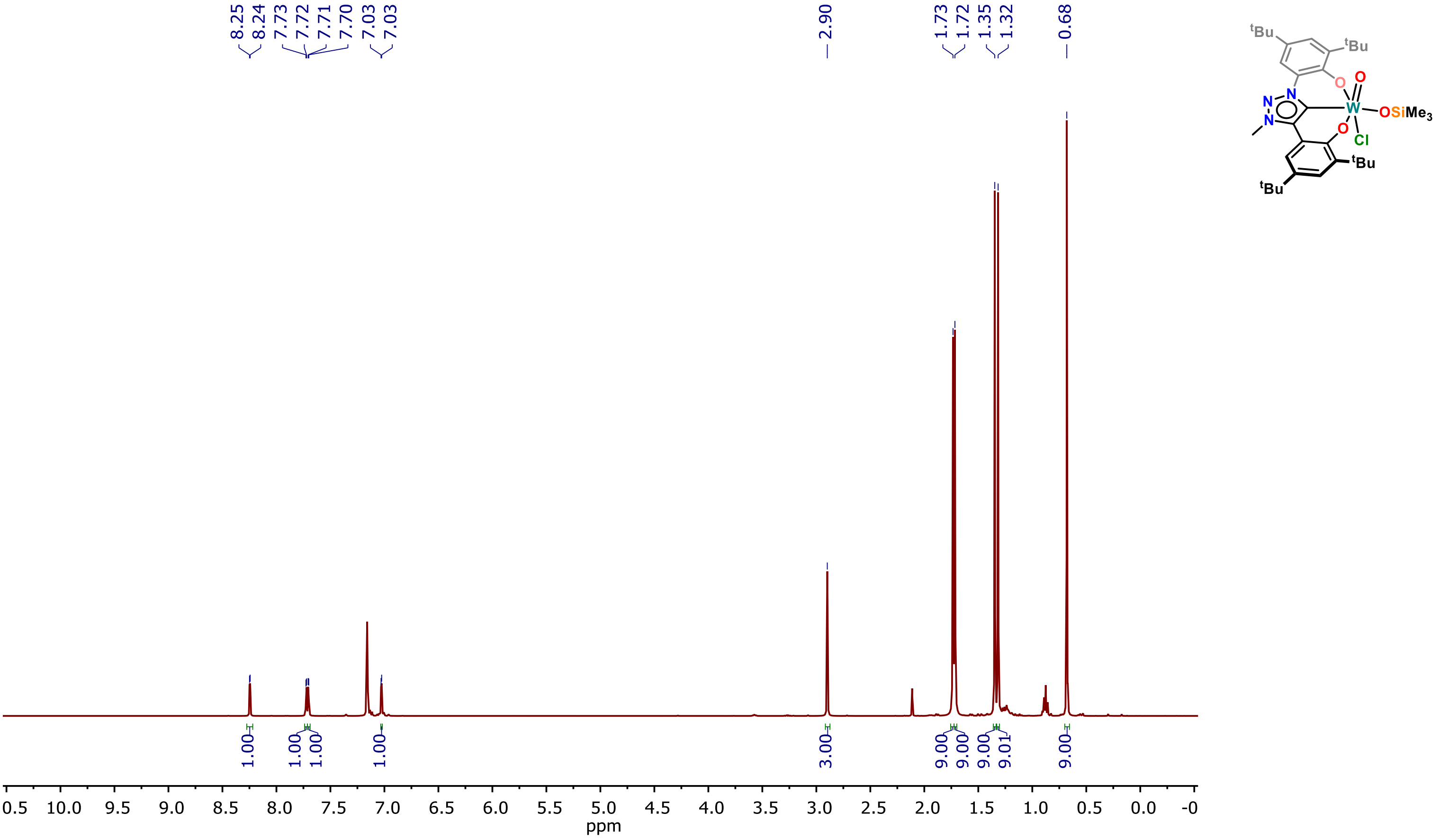

Figure S 6: ^{1}H NMR of **3-W’** in C_6D_6 at 298K.

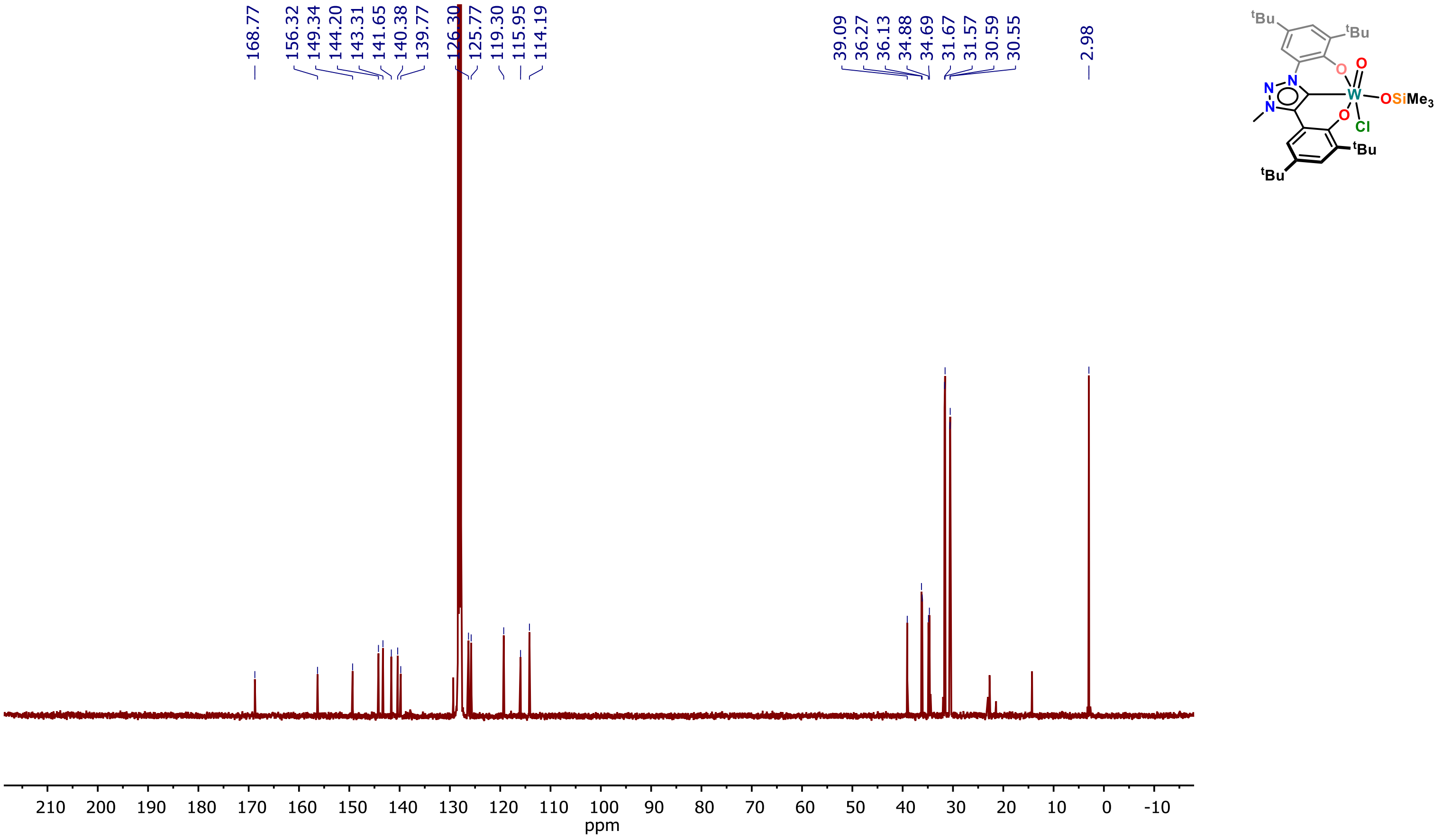

Figure S 7: ^{13}C NMR of **3-W’** in C_6D_6 at 298K.

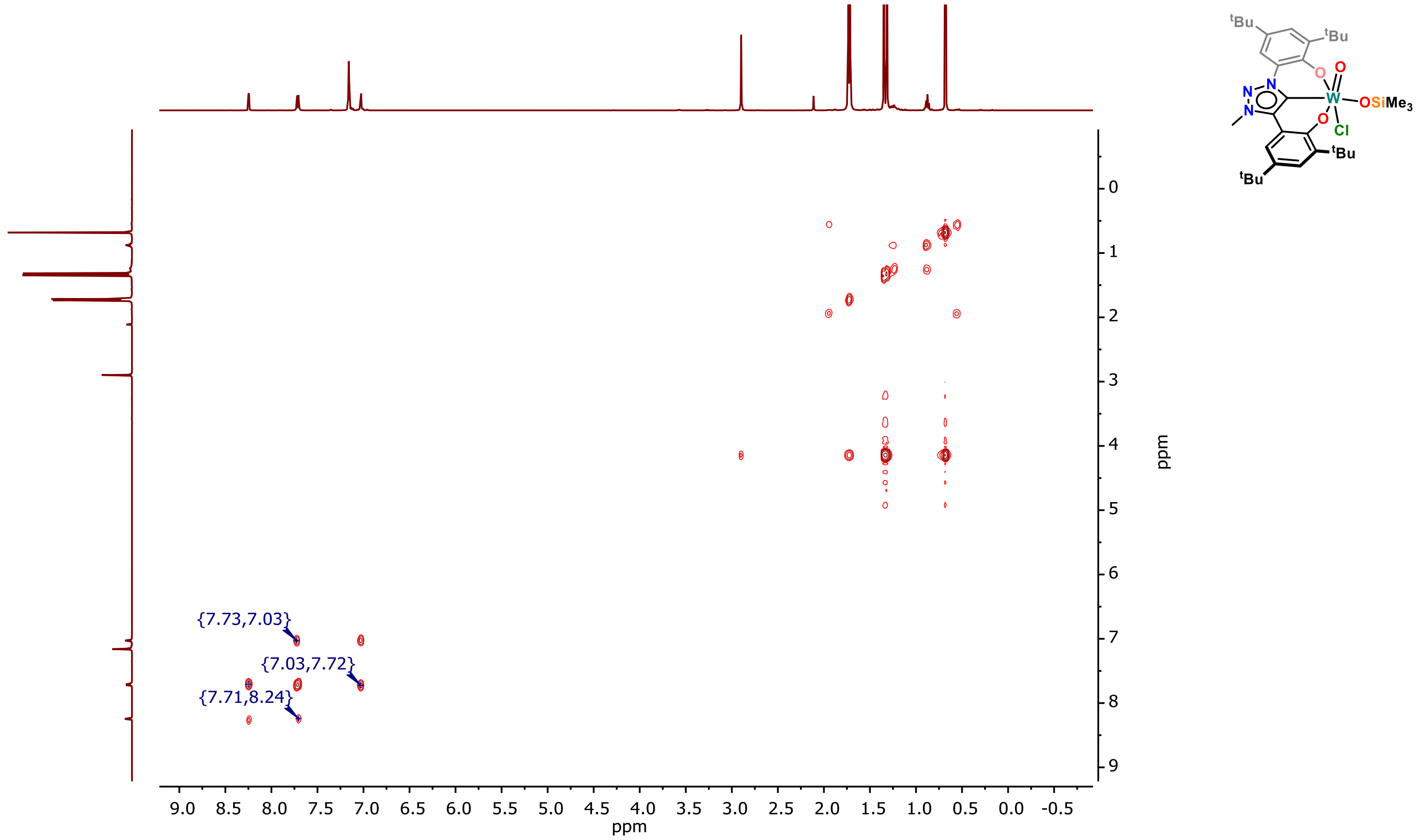

Figure S 8: ^{1}H-^{1}H COSY NMR of **3-W’** in C_6D_6 at 298K.

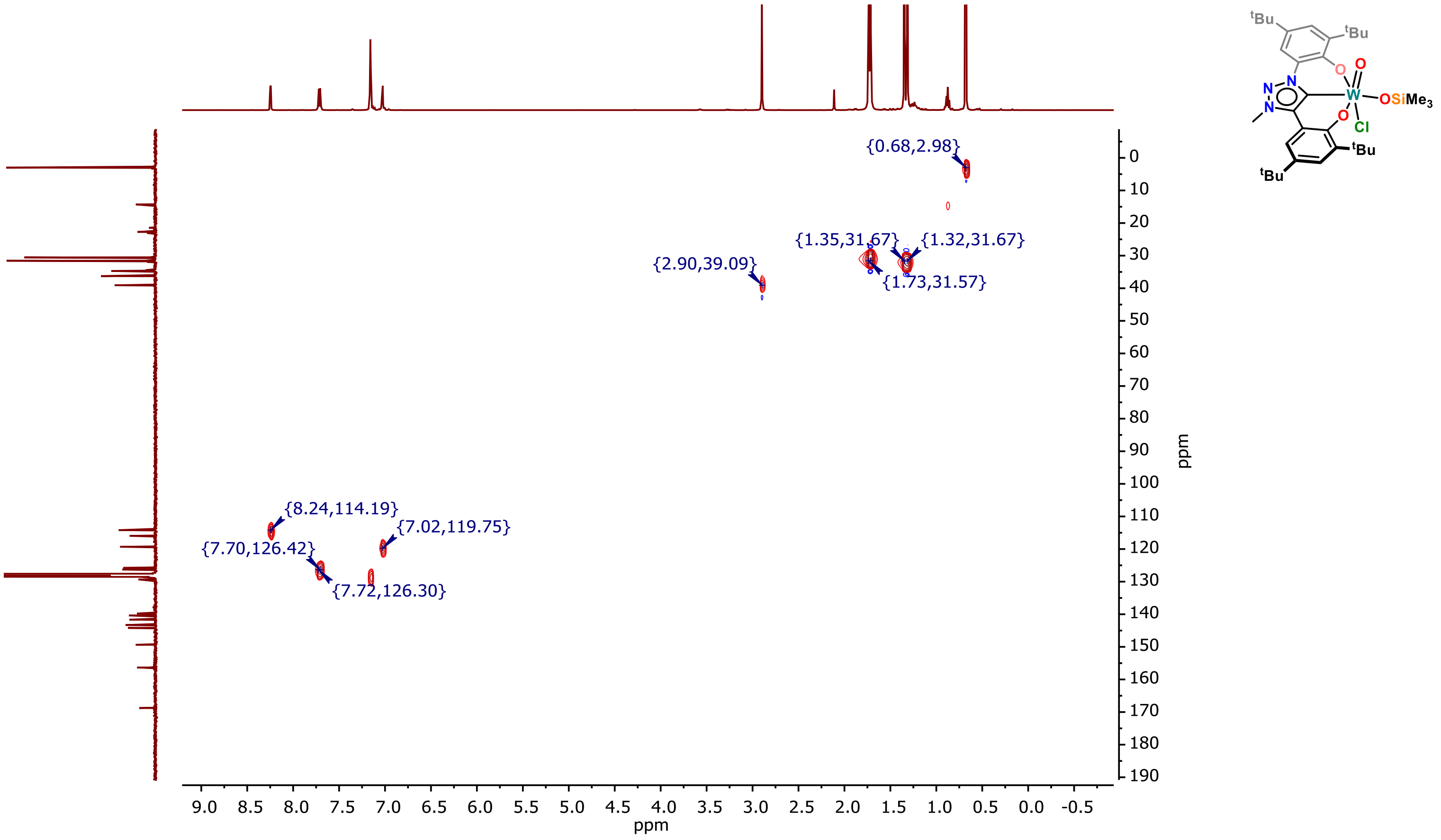

Figure S 9: ^{1}H-^{13}C HSQC NMR of **3-W’** in C_6D_6 at 298K.

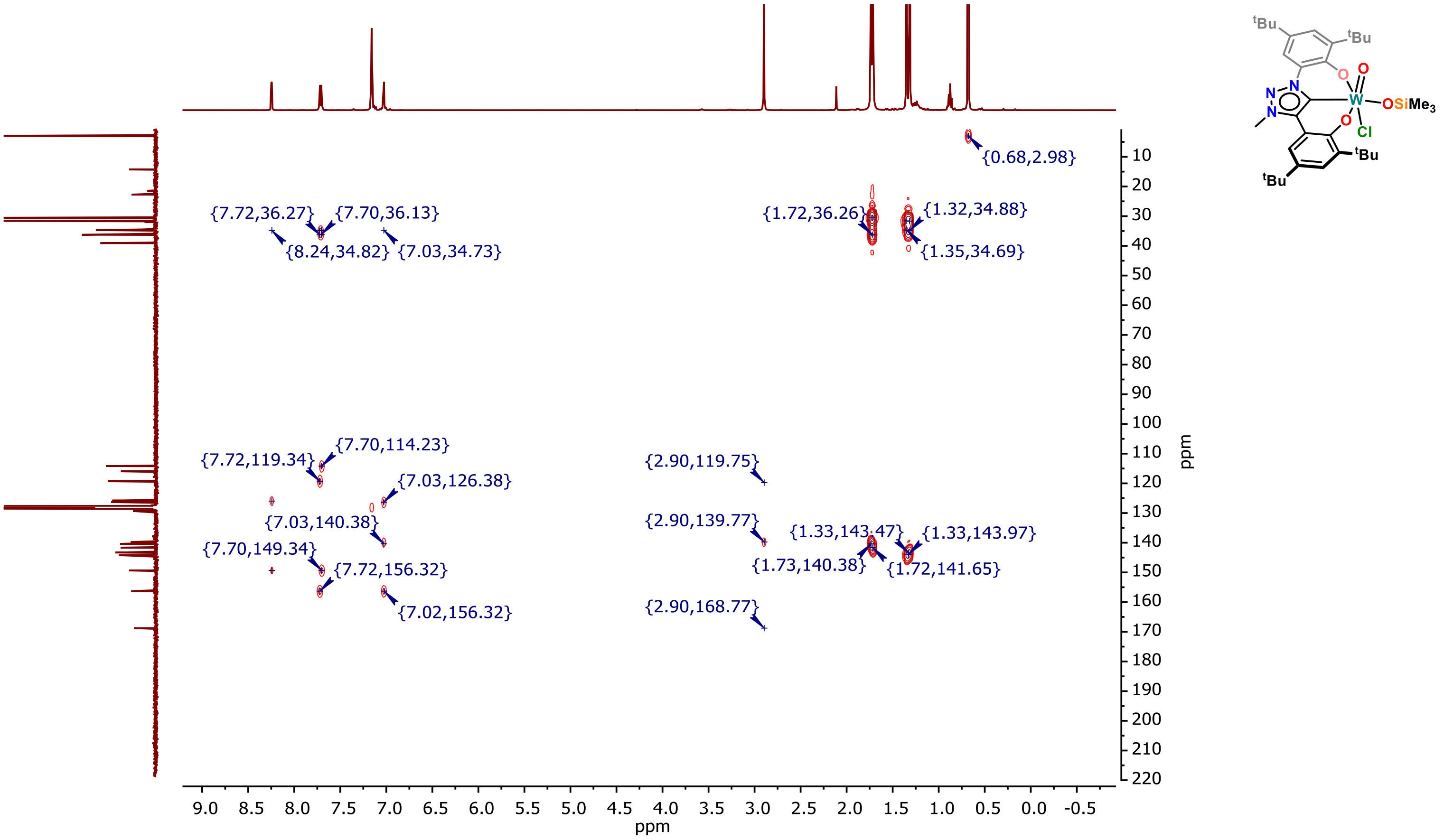

Figure S 10: ^{1}H-^{13}C HMBC NMR of **3-W’** in C_6D_6 at 298K.

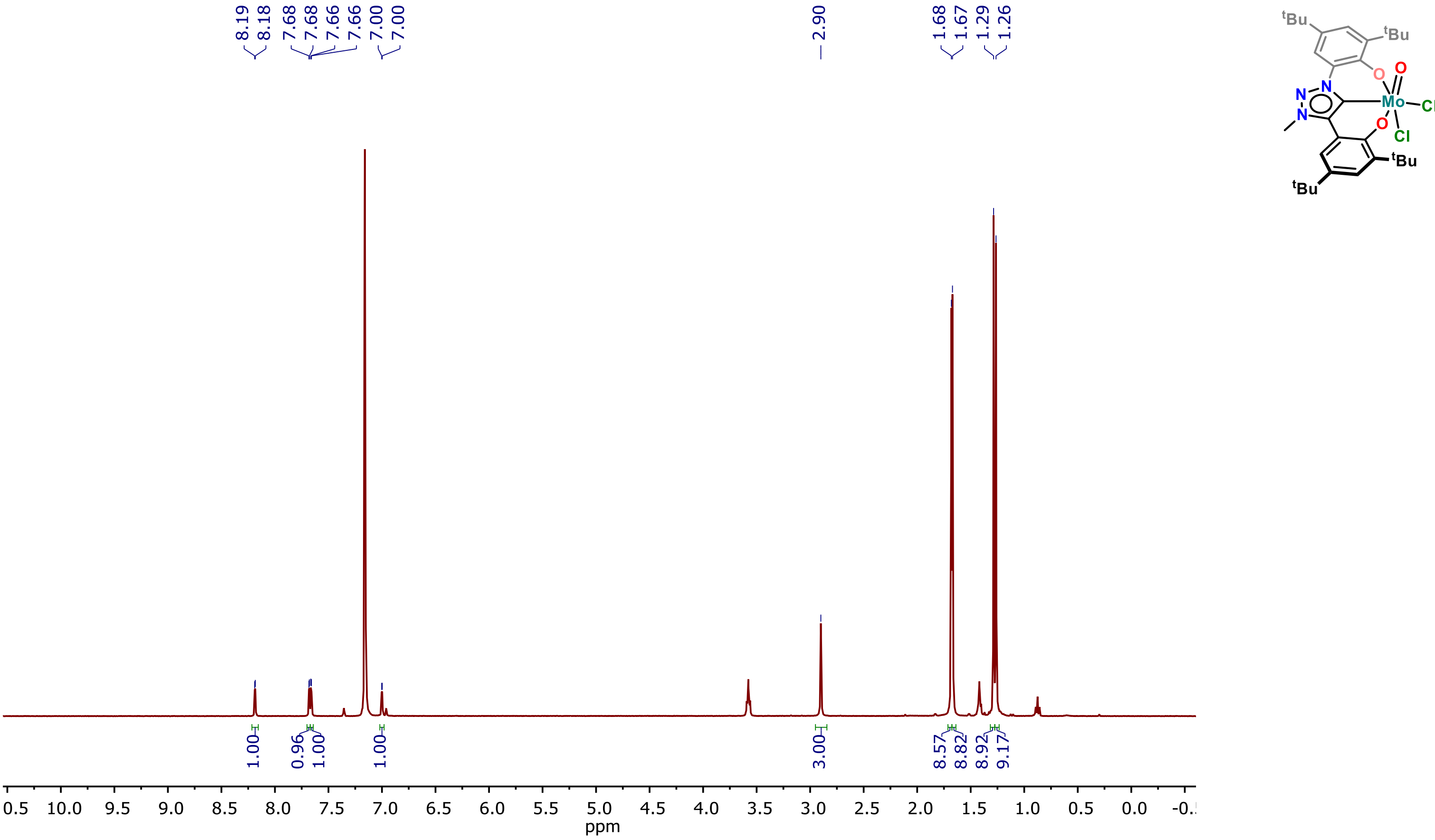

Figure S 11: ^{1}H NMR of **3-Mo** in C_6D_6 at 298K.

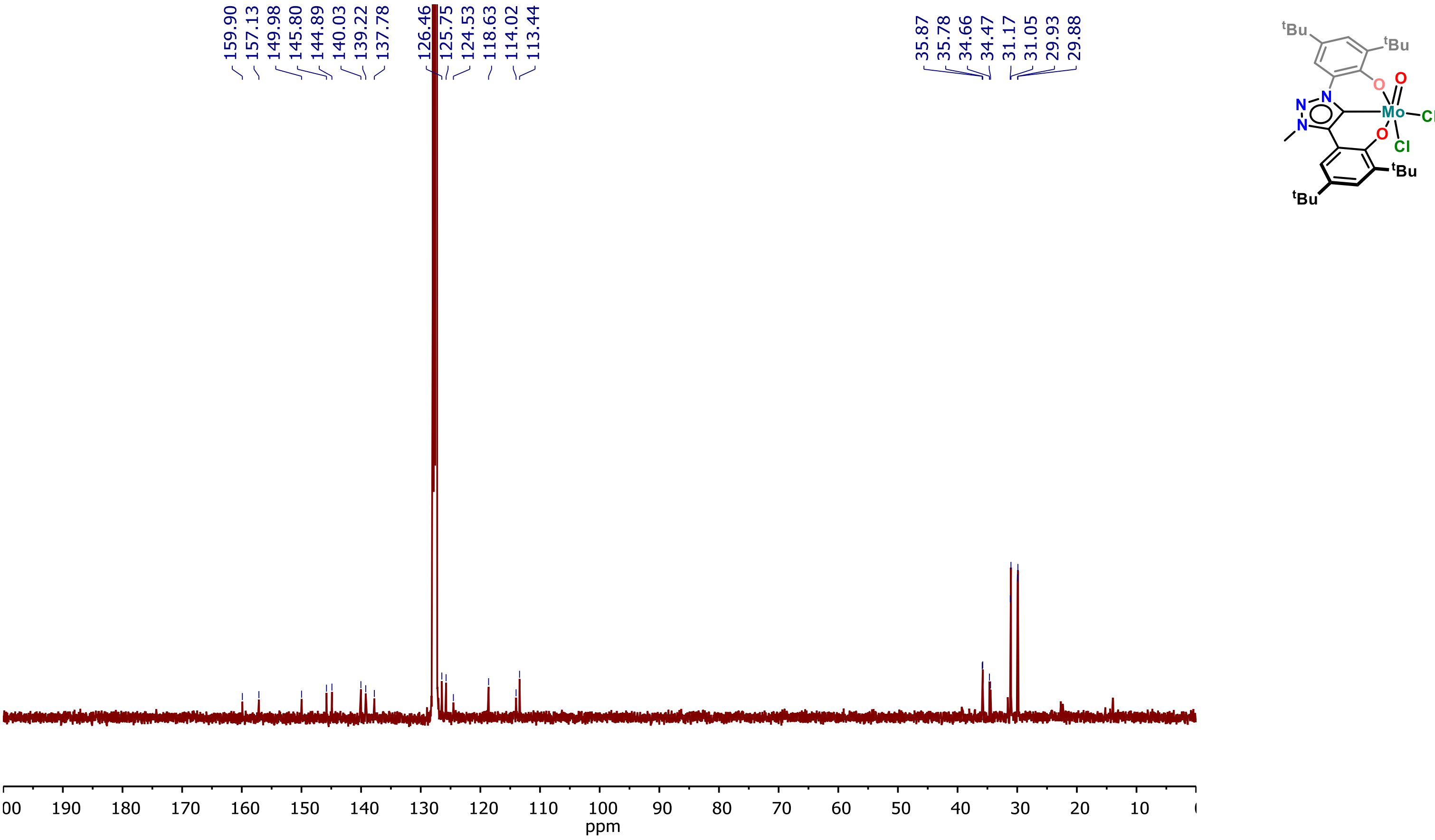

Figure S 12: ^{13}C NMR of **3-Mo** in C_6D_6 at 298K.

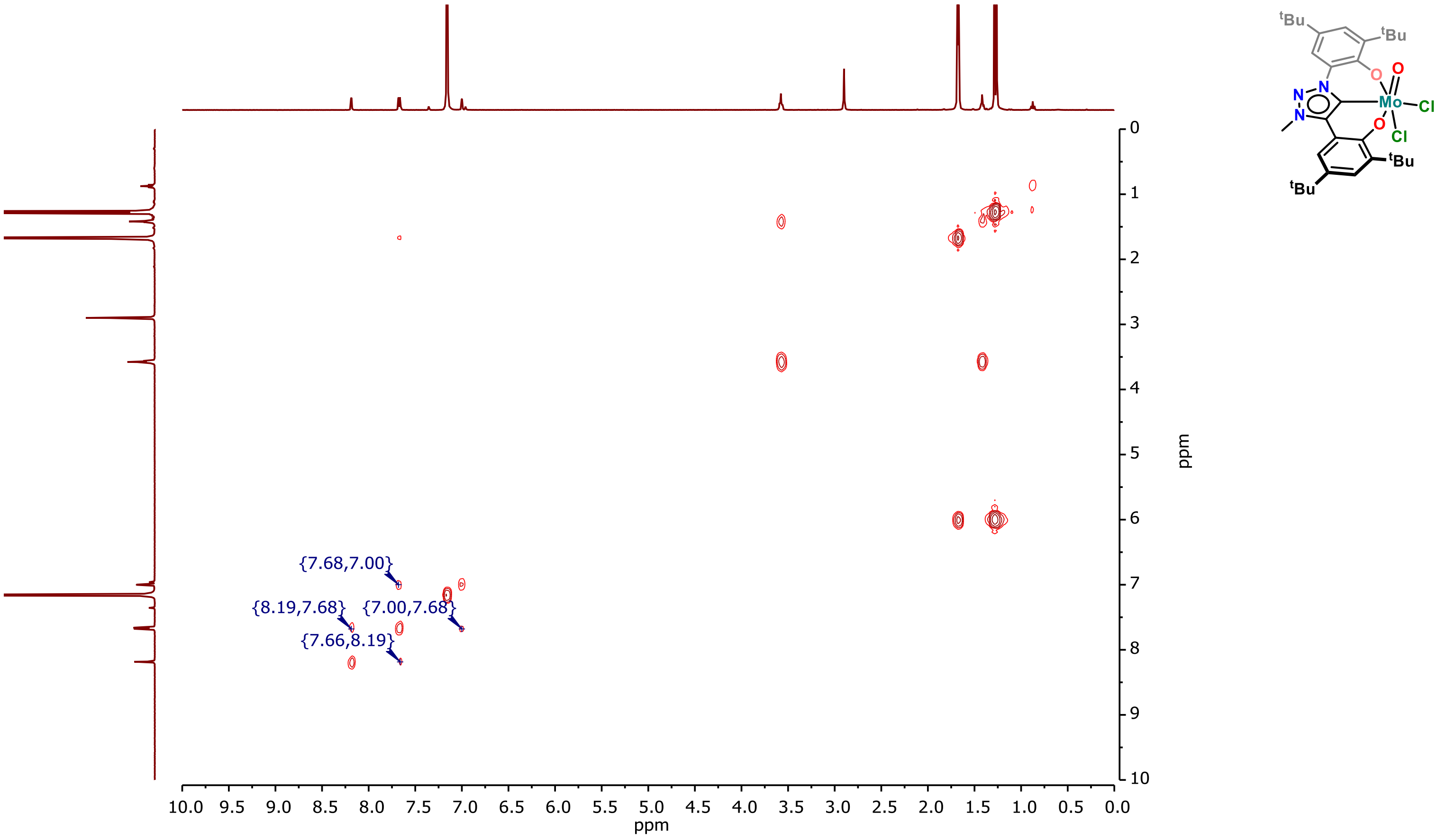

Figure S 13: ^{1}H-^{1}H COSY NMR of **3-Mo** in C_6D_6 at 298K.

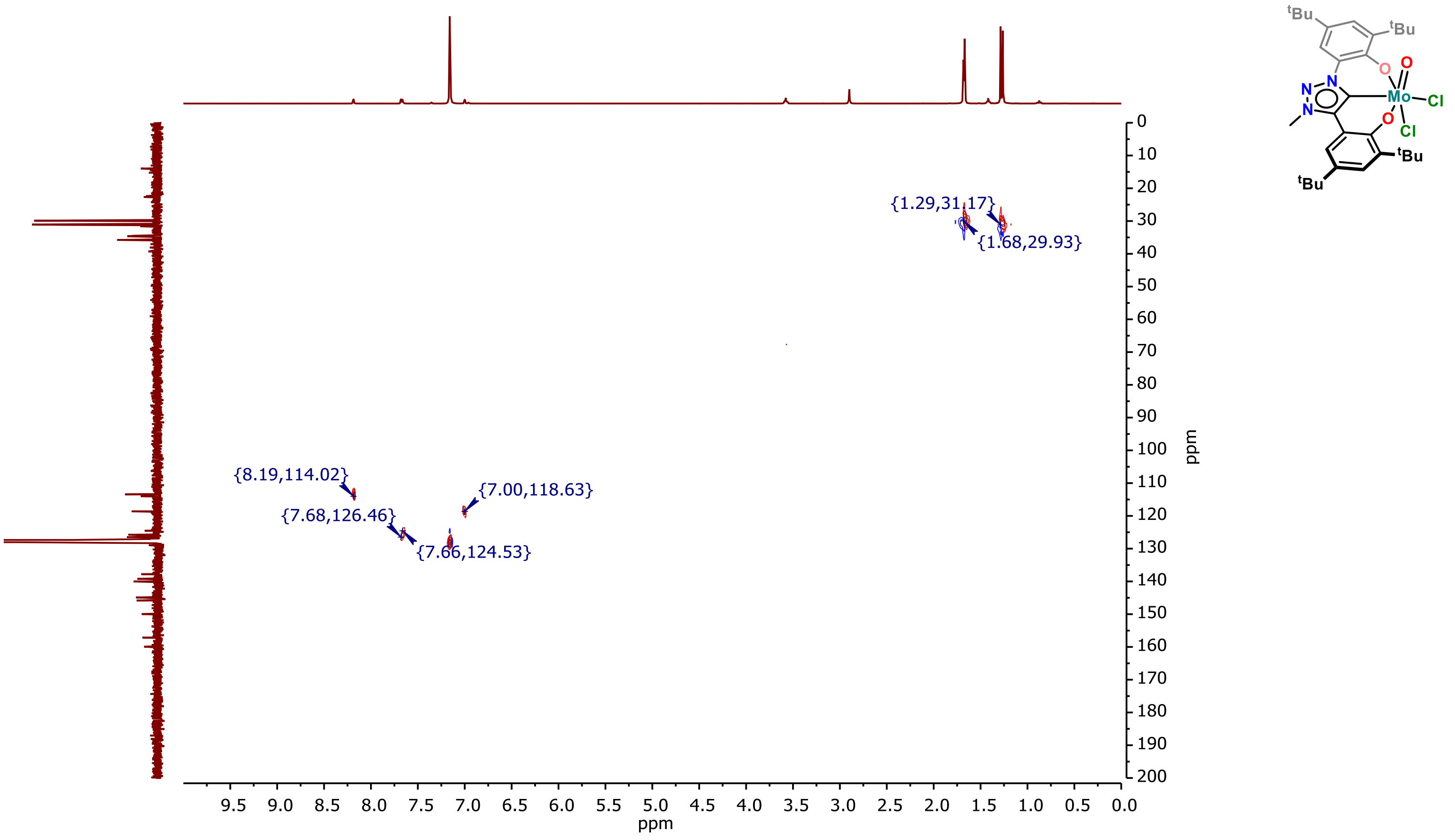

Figure S 14: ^{1}H-^{13}C HSQC NMR of **3-Mo** in C_6D_6 at 298K.

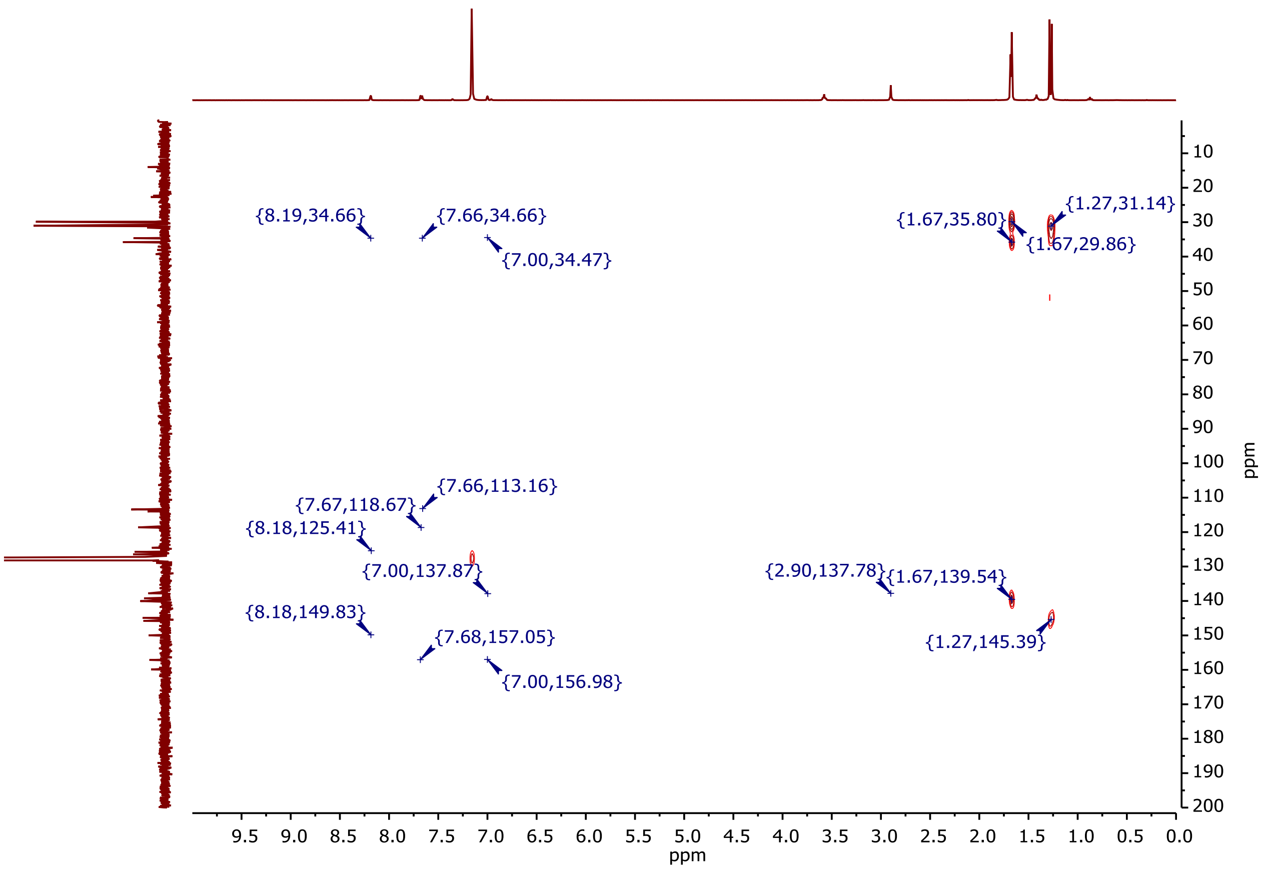

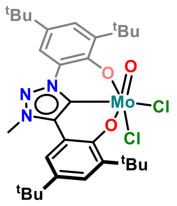

Figure S 15: ^{1}H-^{13}C HMBC NMR of **3-Mo** in C_6D_6 at 298K.

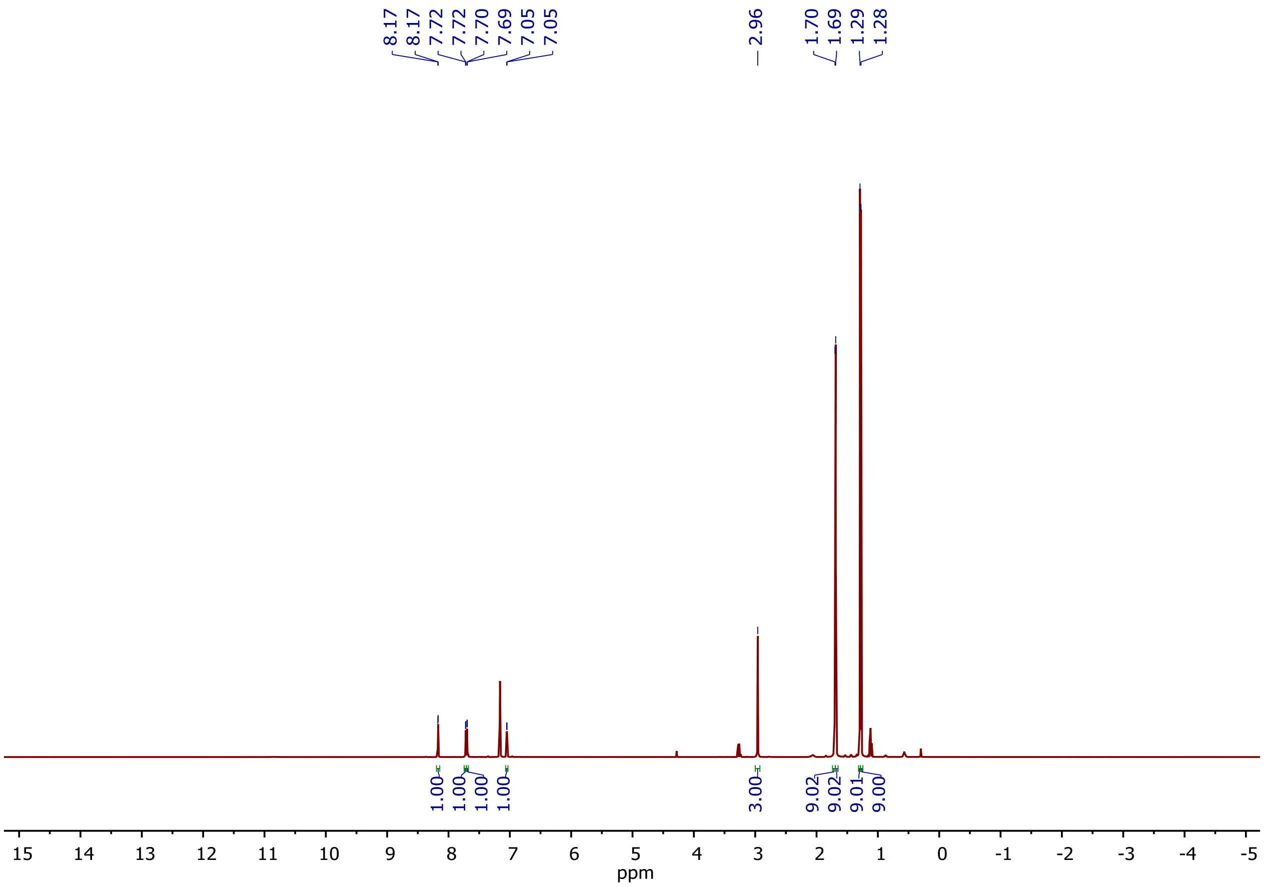

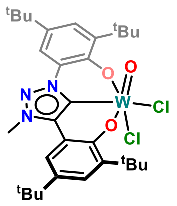

Figure S 16: ^{1}H NMR of **3-W** in C_6D_6 at 298K.

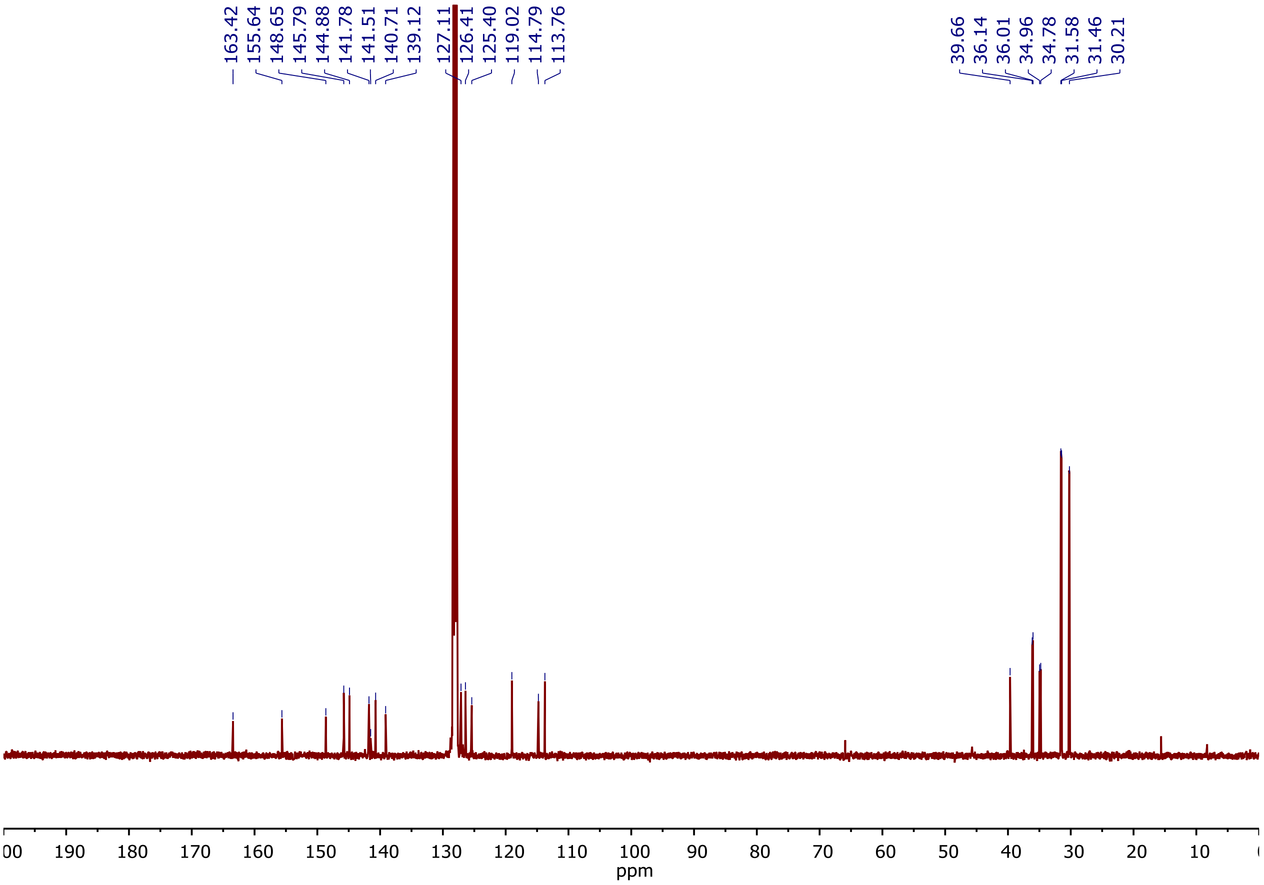

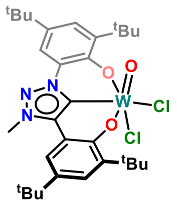

Figure S 17: ^{13}C NMR of **3-W** in C_6D_6 at 298K.

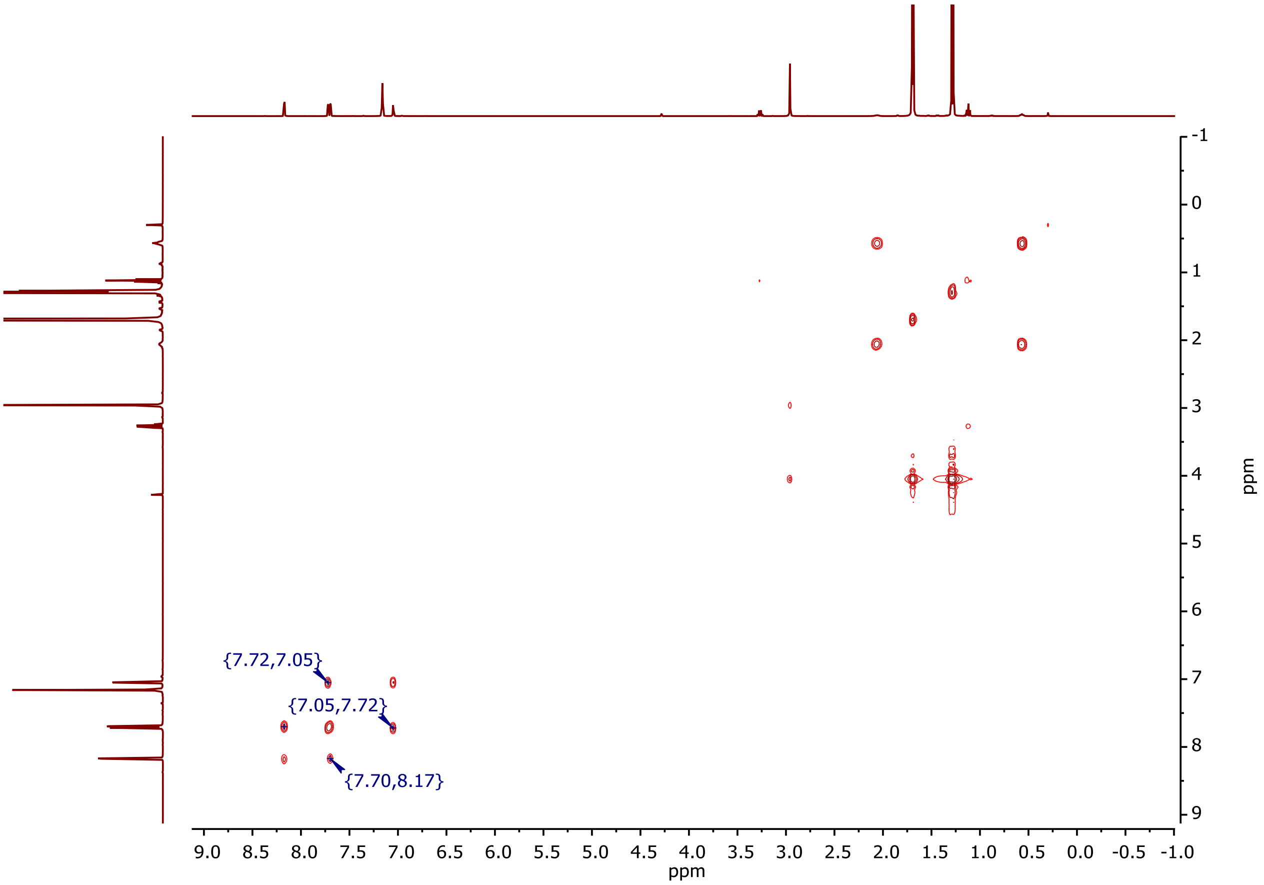

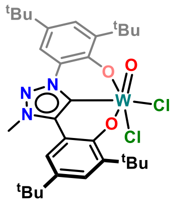

Figure S 18: ^{1}H-^{1}H COSY NMR of **3-W** in C_6D_6 at 298K.

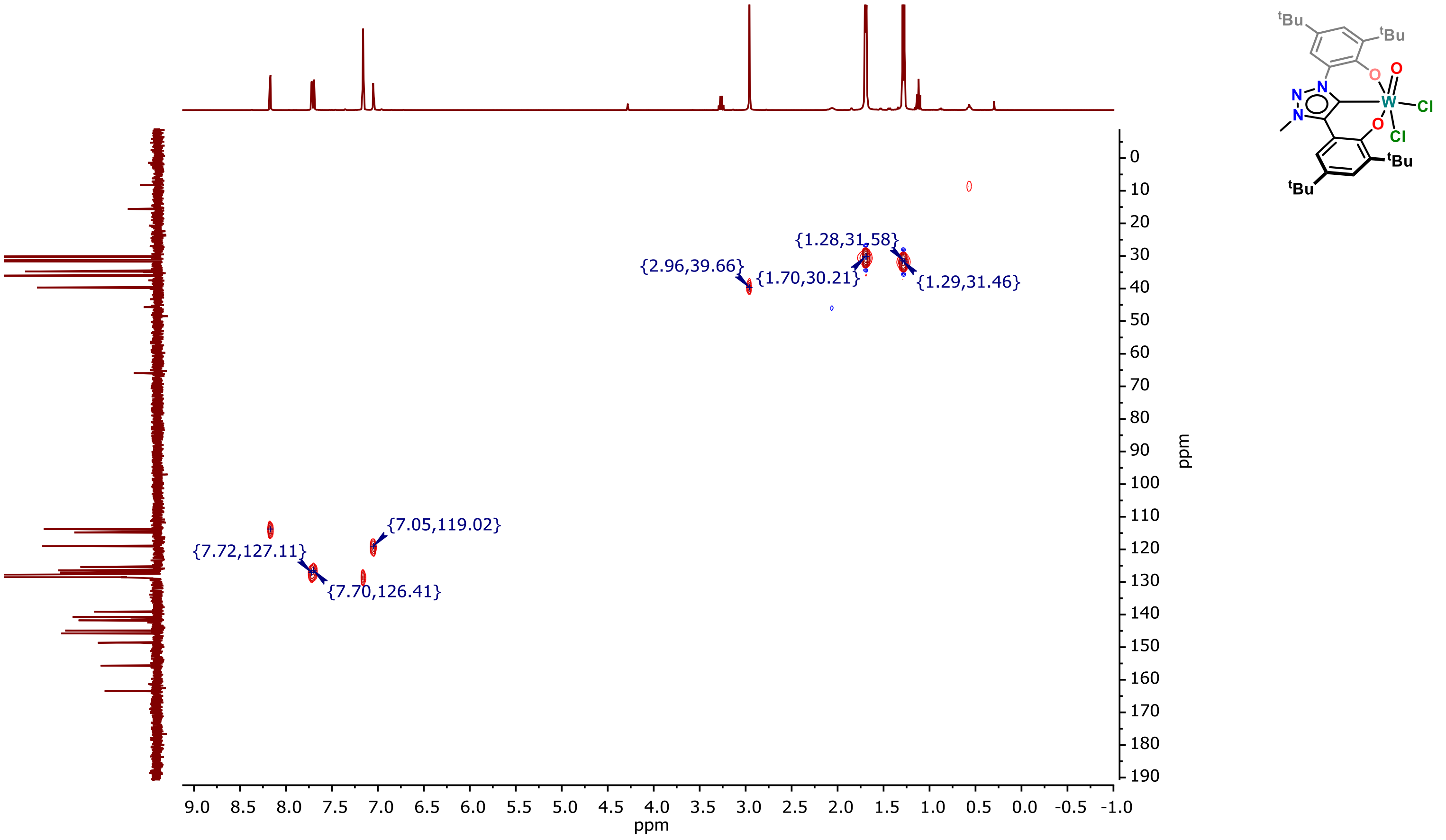

Figure S 19: ^{1}H-^{13}C HSQC NMR of **3-W** in C_6D_6 at 298K.

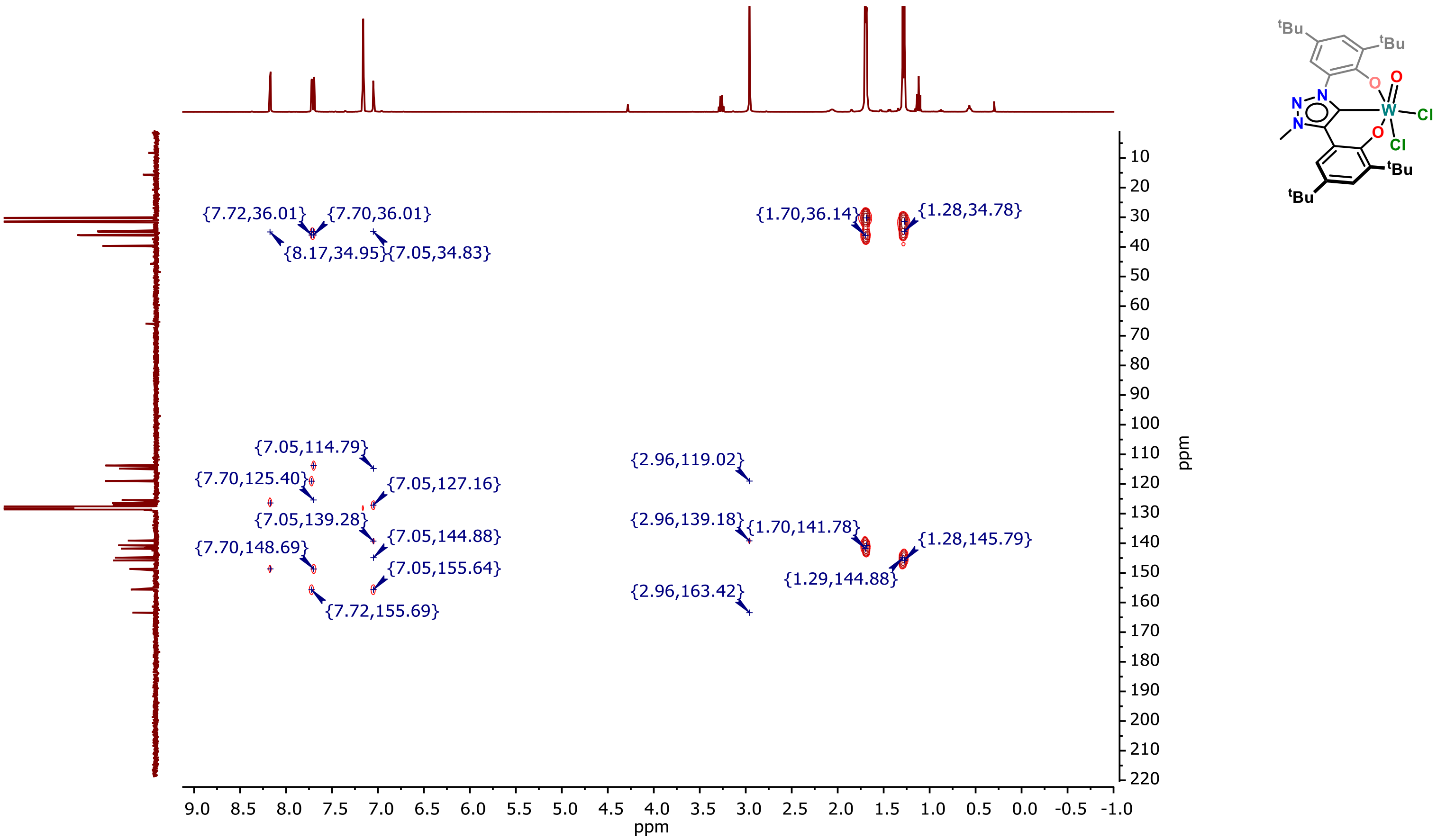

Figure S 20: ^{1}H-^{13}C HMBC NMR of **3-W** in C_6D_6 at 298K.

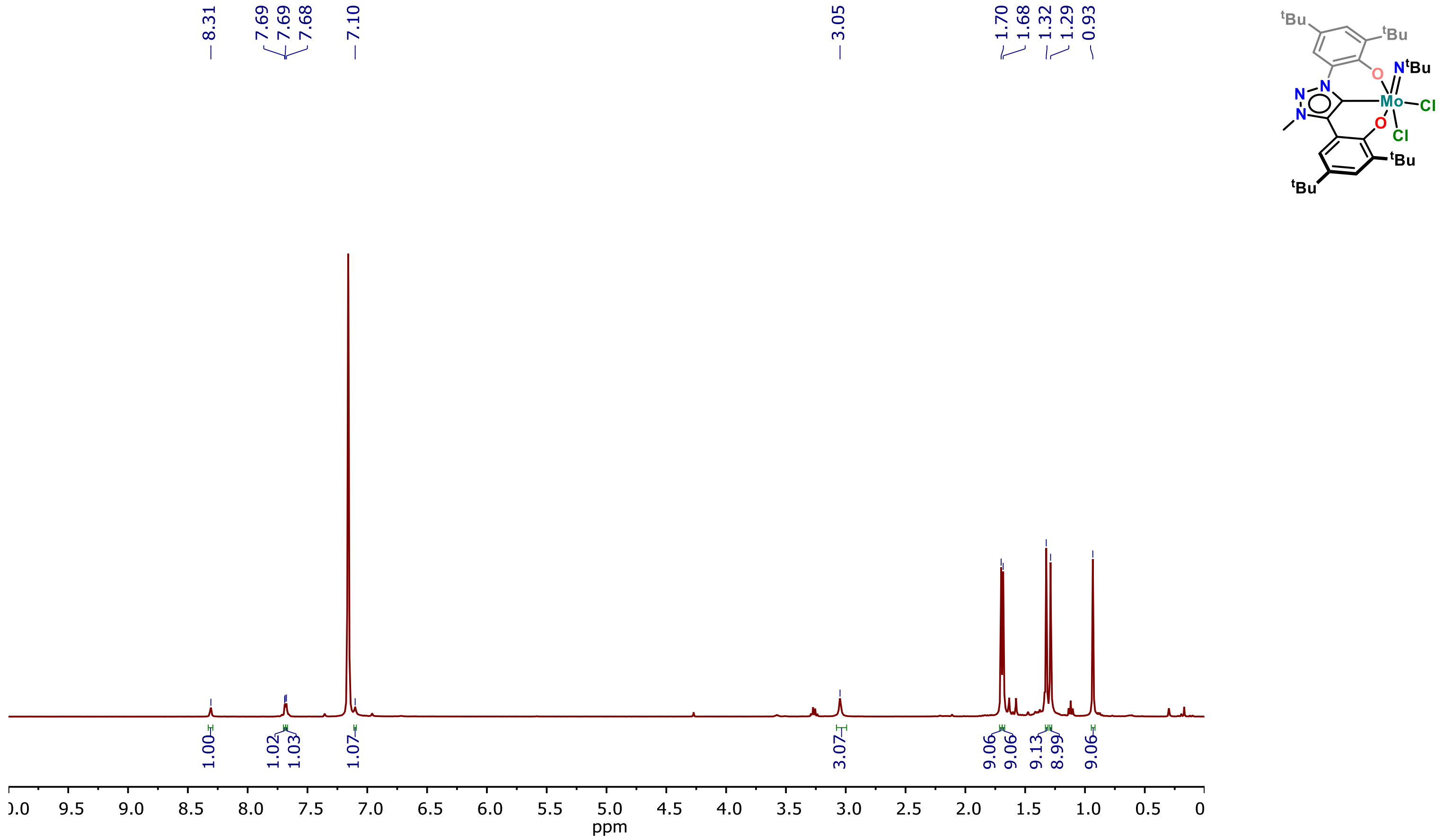

Figure S 21: ^{1}H NMR of **4-Mo** in C_6D_6 at 298K.

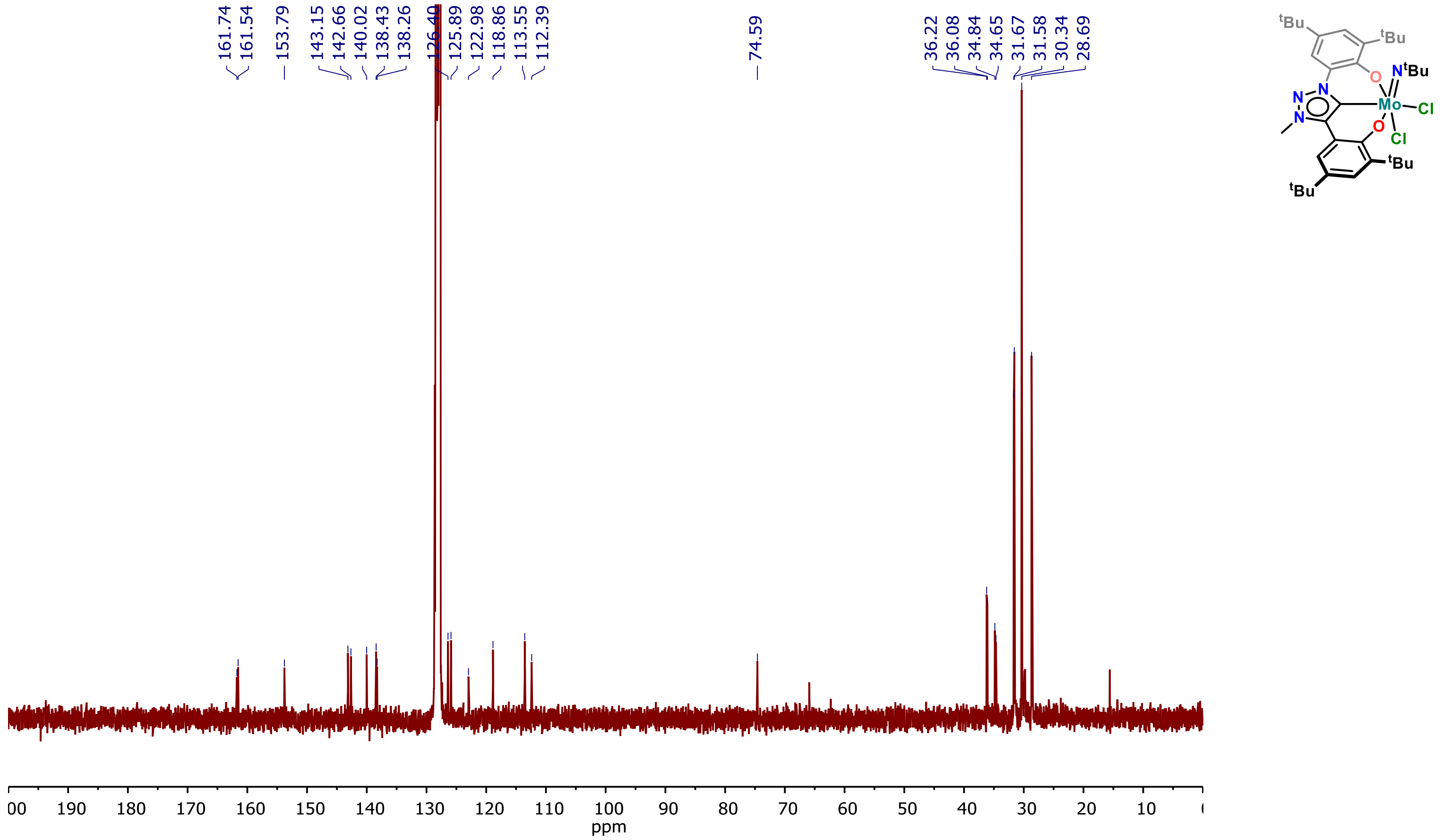

Figure S 22: ^{13}C NMR of **4-Mo** in C_6D_6 at 298K.

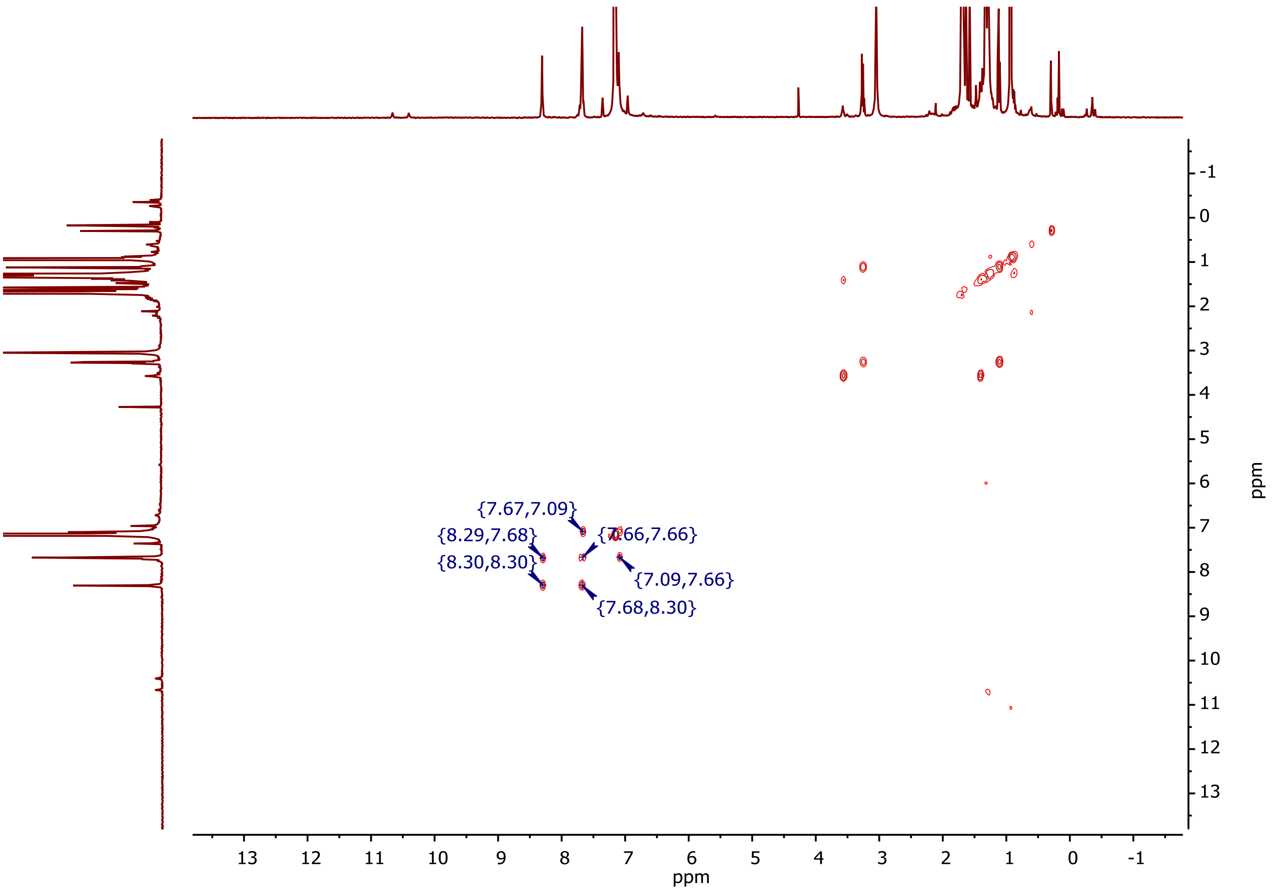

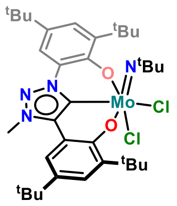

Figure S 23: ^{1}H-^{1}H COSY NMR of **4-Mo** in C_6D_6 at 298K.

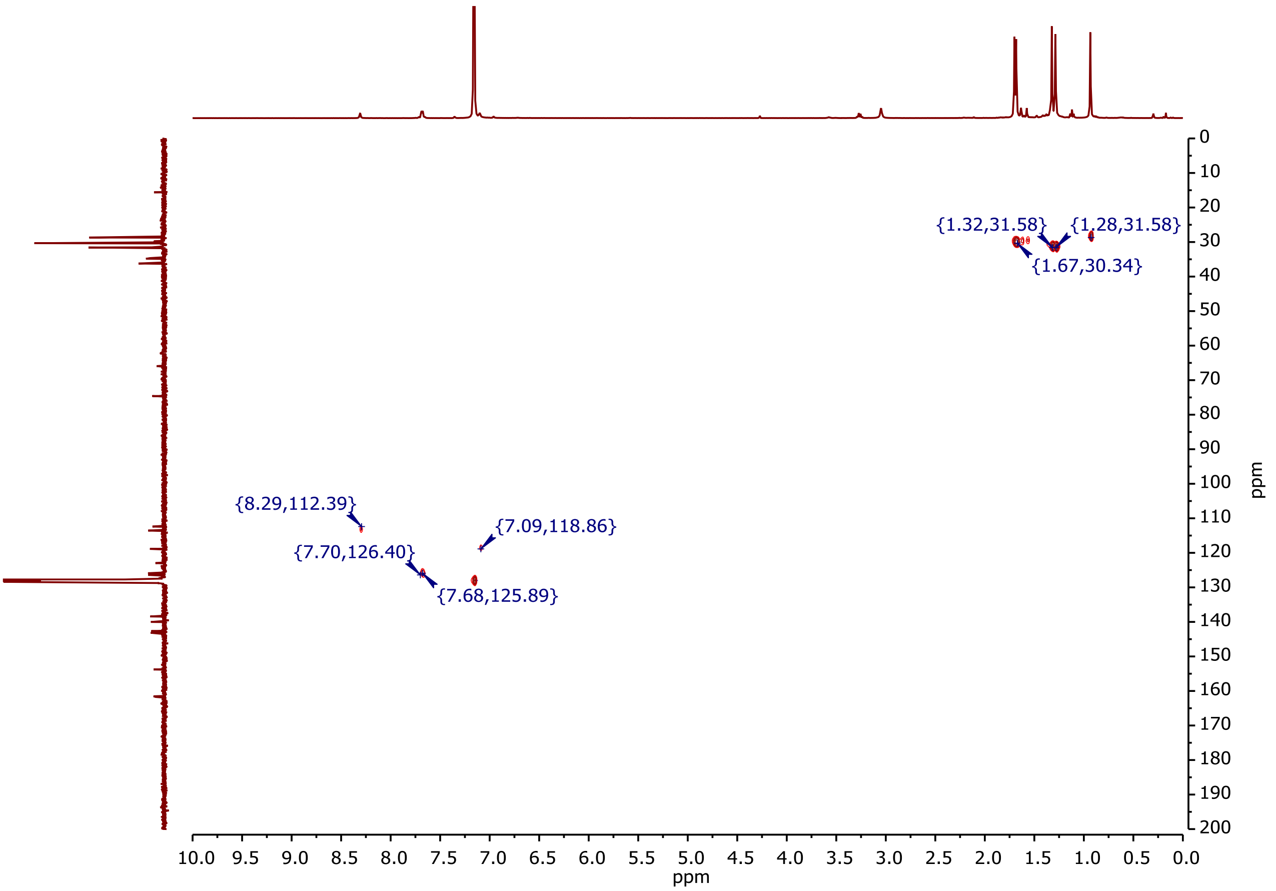

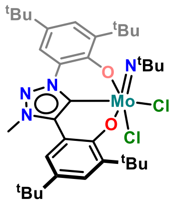

Figure S 24: ^{1}H-^{13}C HSQC NMR of **4-Mo** in C_6D_6 at 298K.

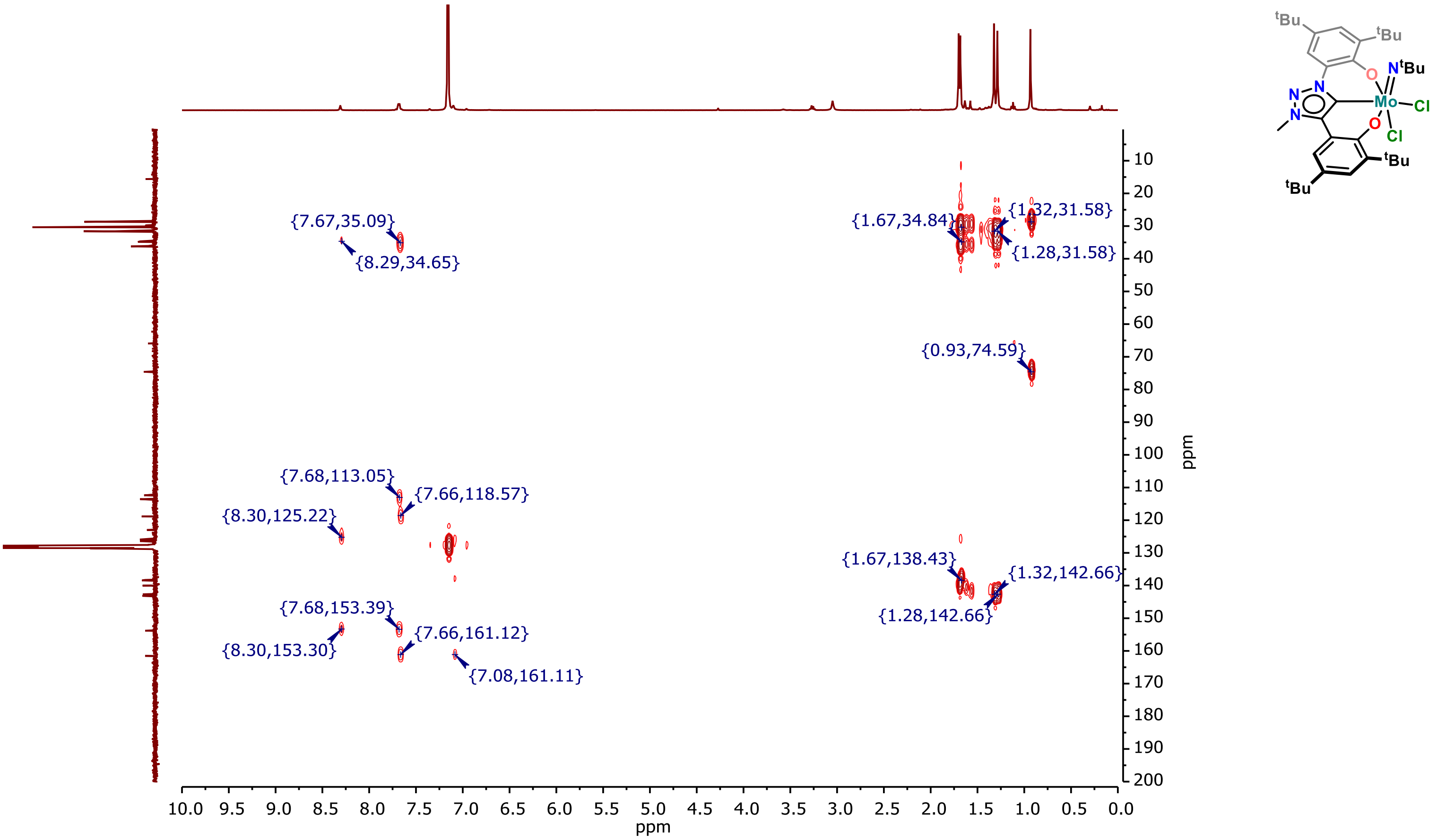

Figure S 25: ^{1}H-^{13}C HMBC NMR of **4-Mo** in C_6D_6 at 298K.

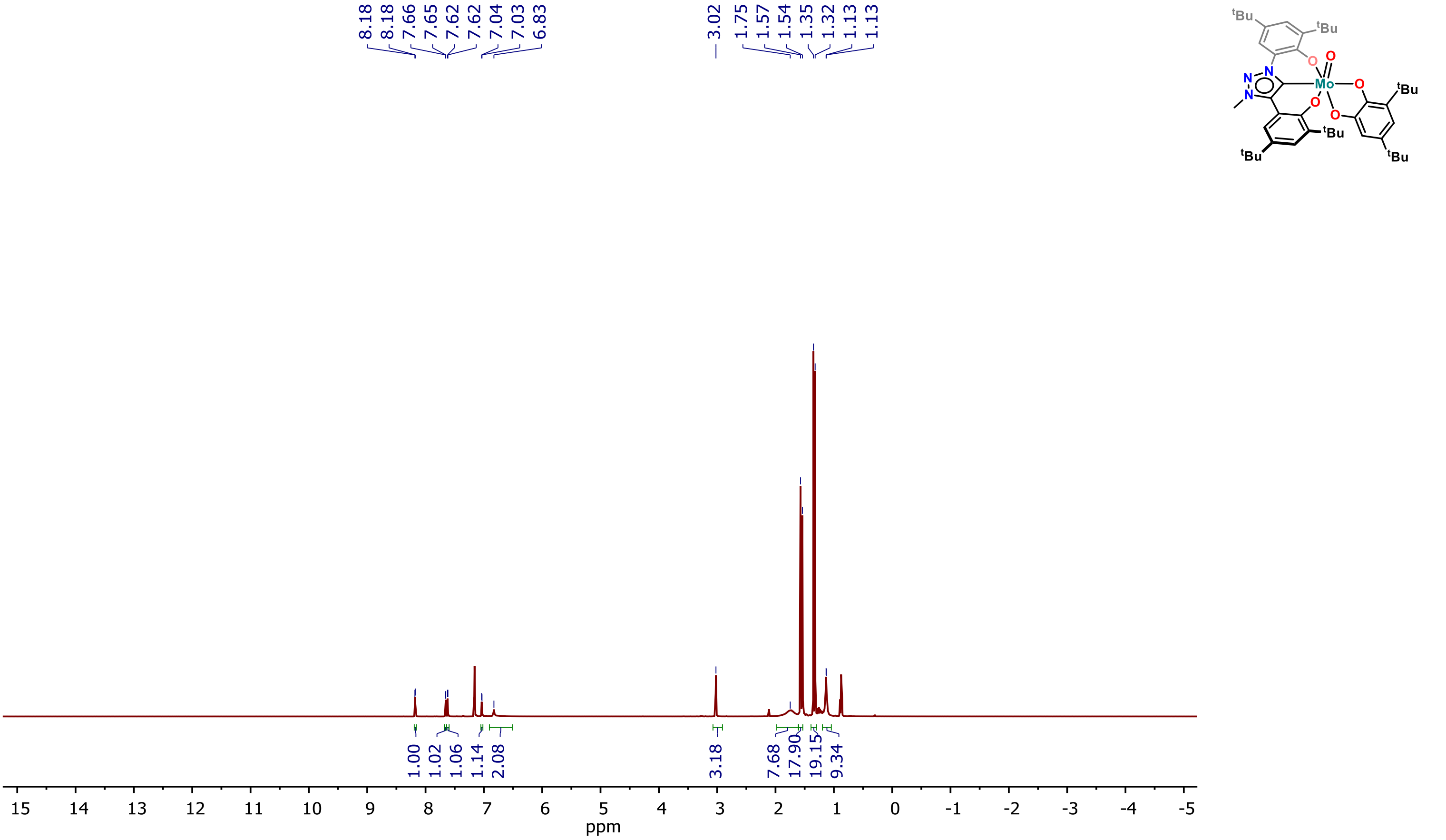

Figure S 26: ^{1}H NMR of **5-Mo** in C_6D_6 at 298K.

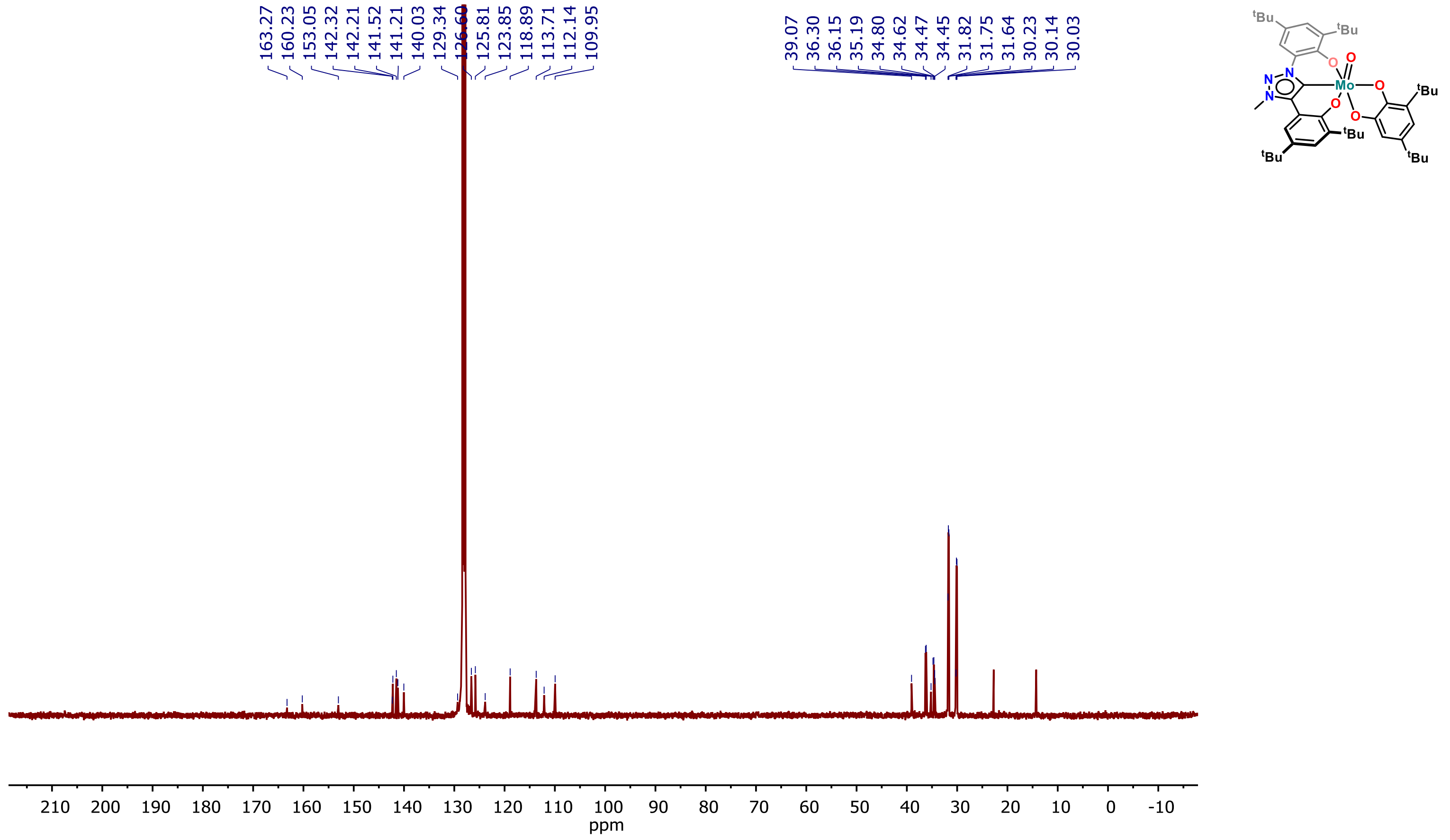

Figure S 27: ^{13}C NMR of **5-Mo** in C_6D_6 at 298K.

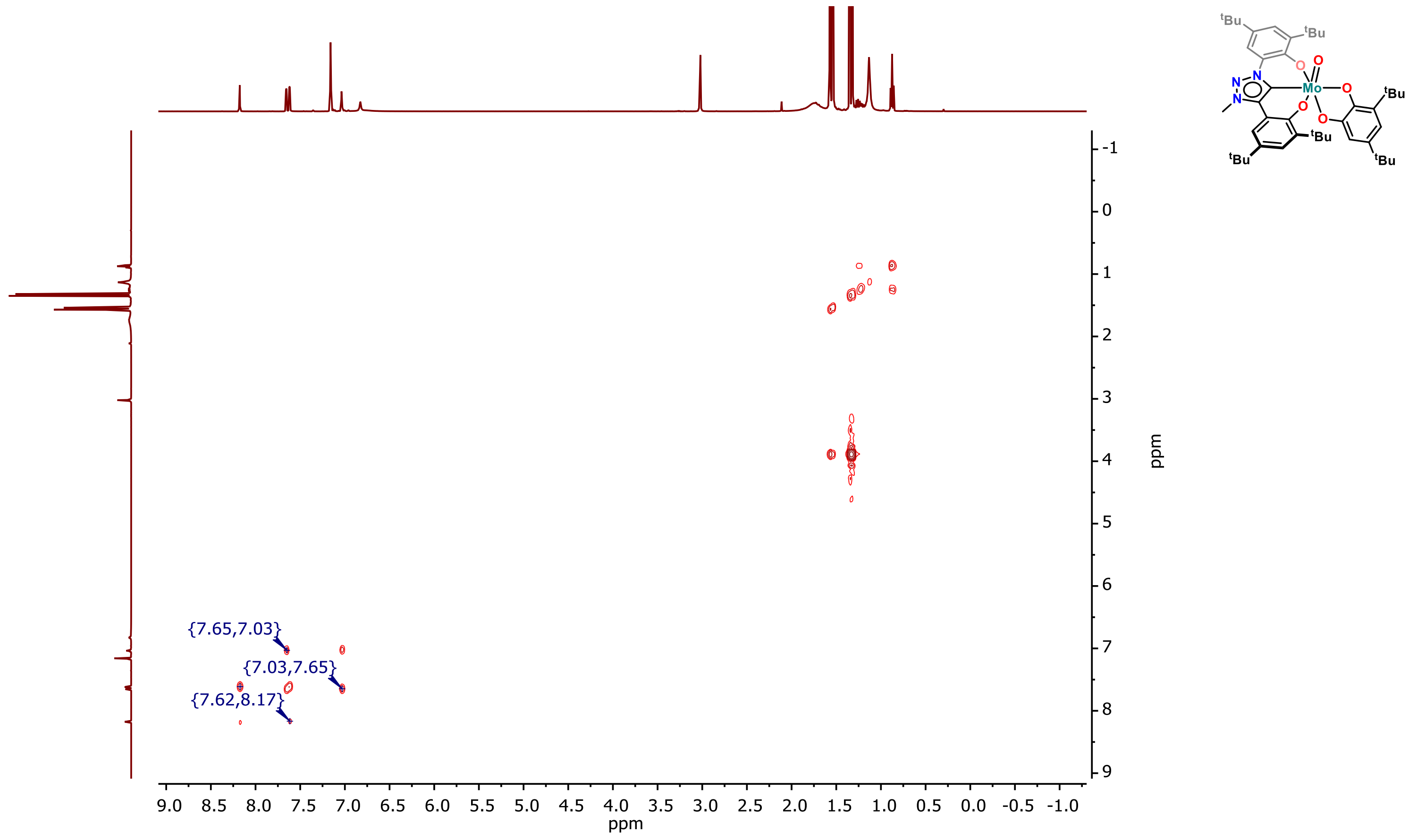

Figure S 28: ^{1}H-^{1}H COSY NMR of **5-Mo** in C_6D_6 at 298K.

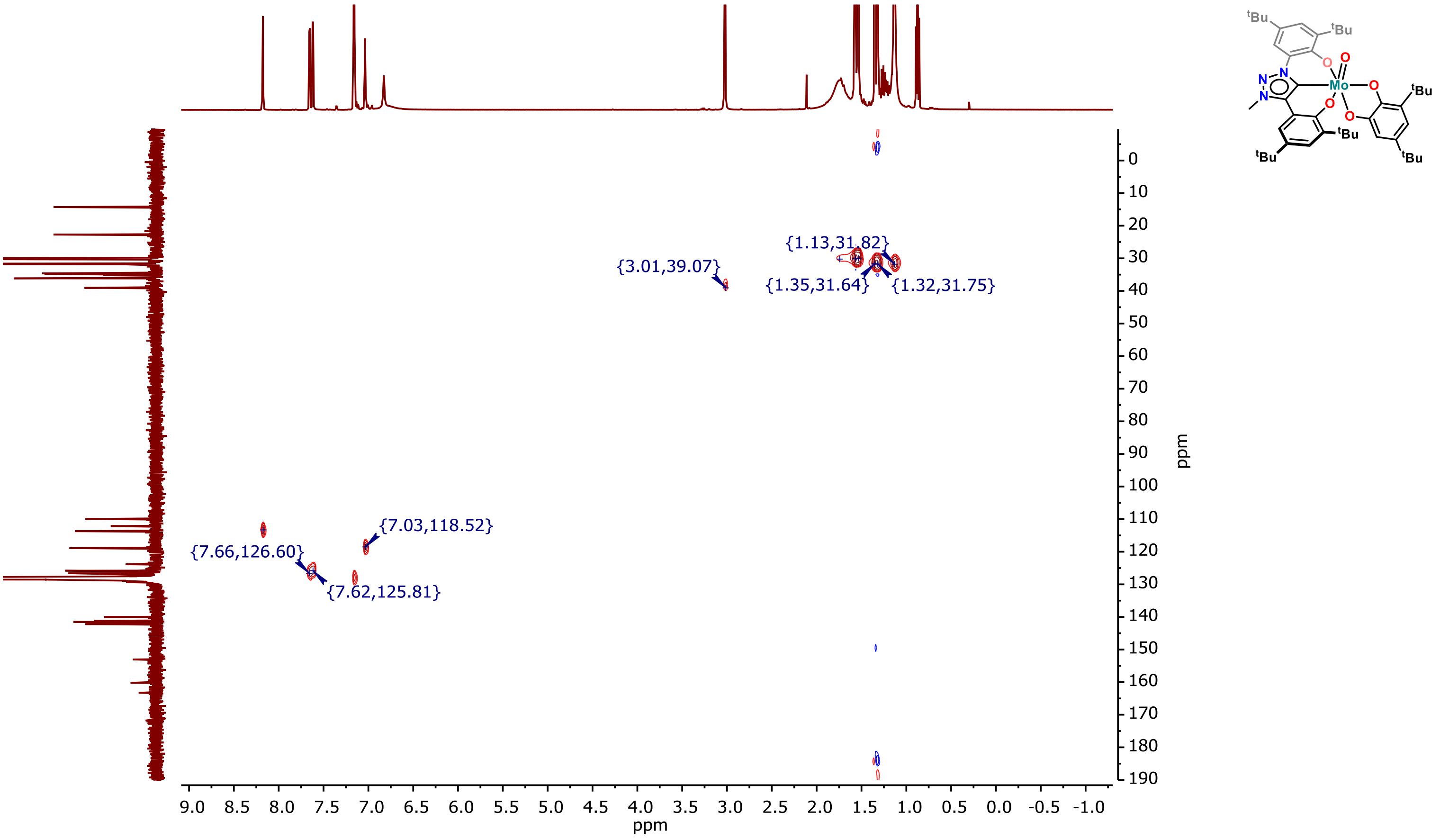

Figure S 29: ^{1}H-^{13}C HSQC NMR of **5-Mo** in C_6D_6 at 298K.

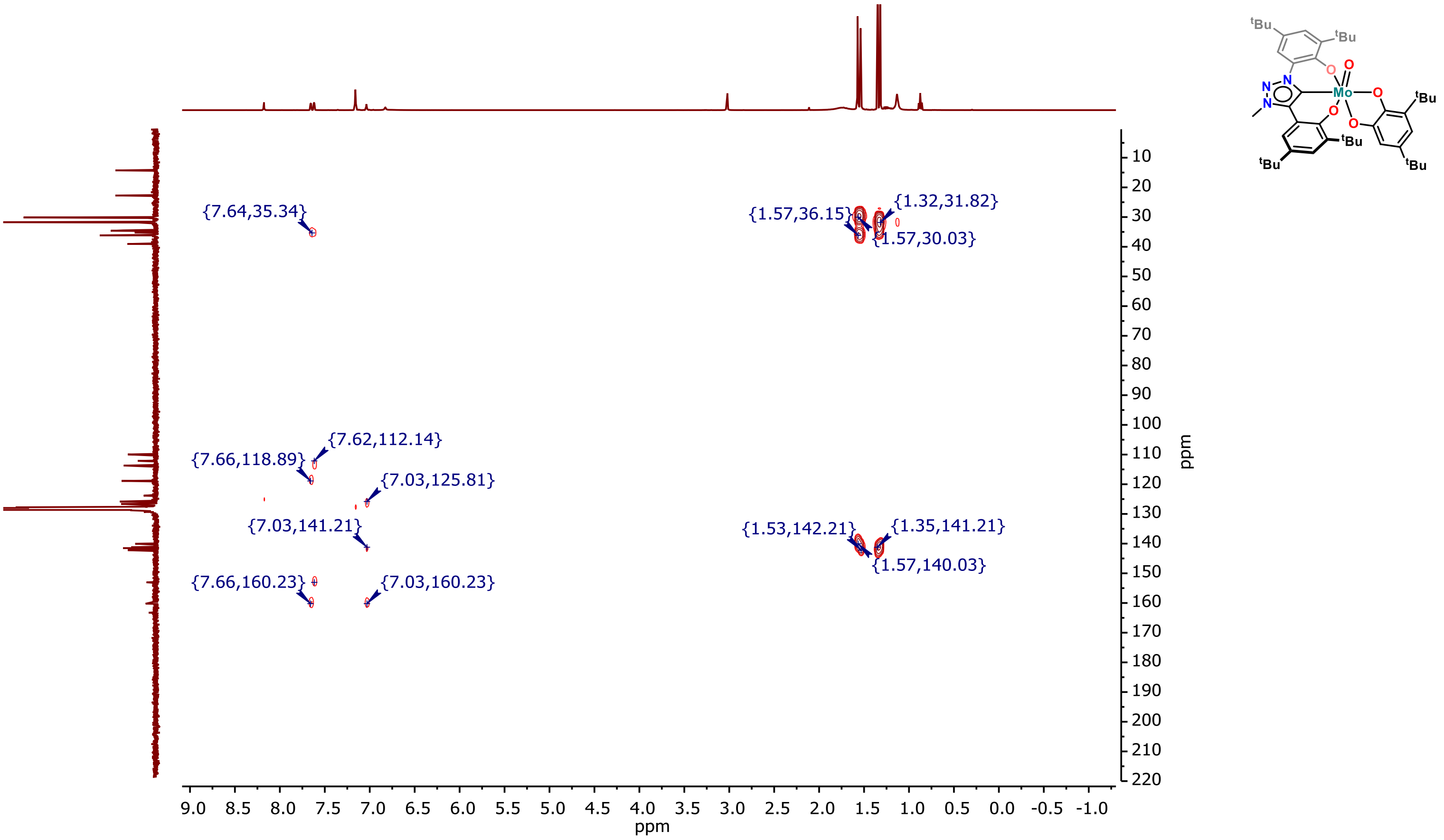

Figure S 30: ^{1}H-^{13}C HMBC NMR of **5-Mo** in C_6D_6 at 298K.

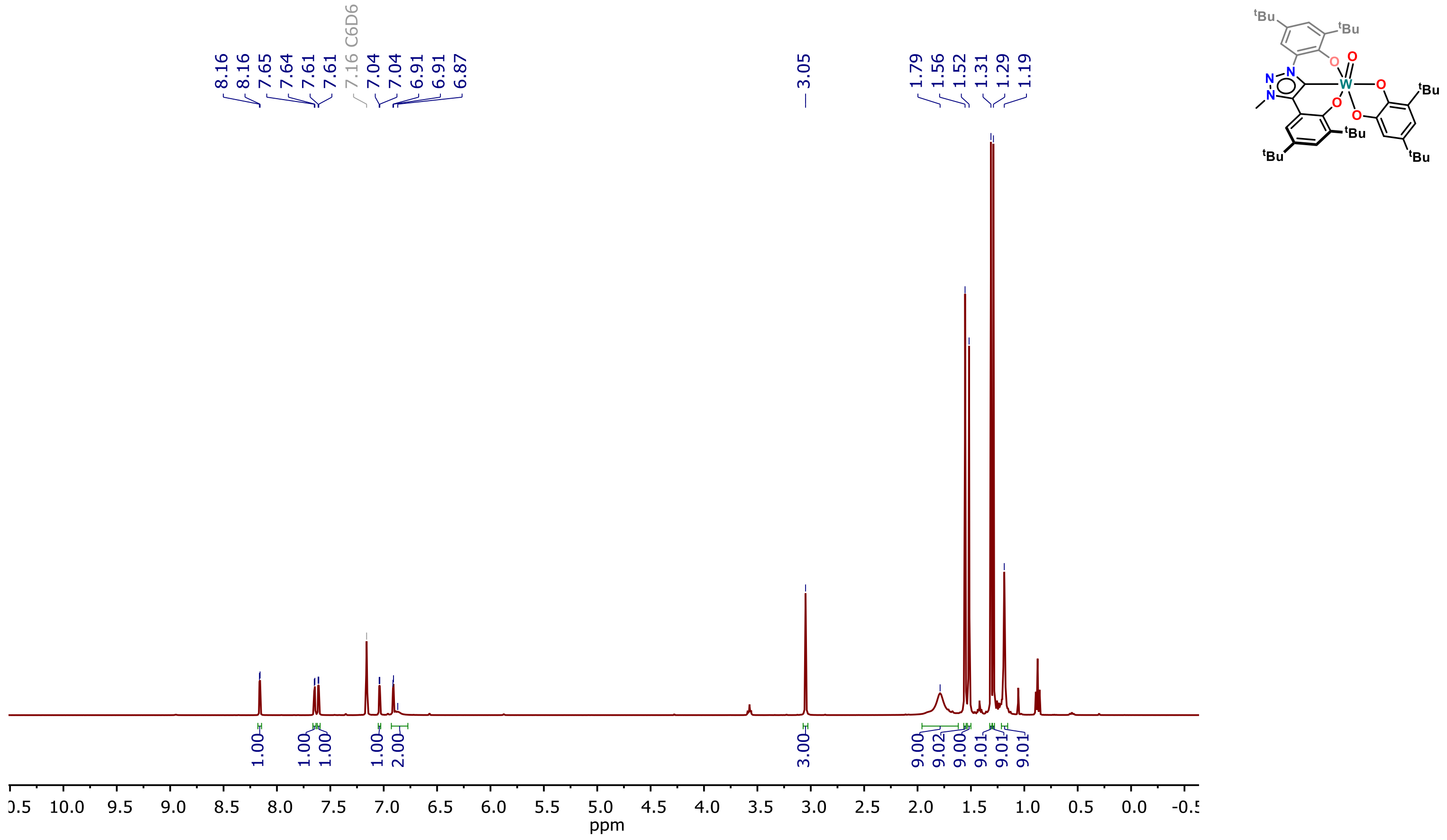

Figure S 31: ^{1}H NMR of **5-W** in C_6D_6 at 298K.

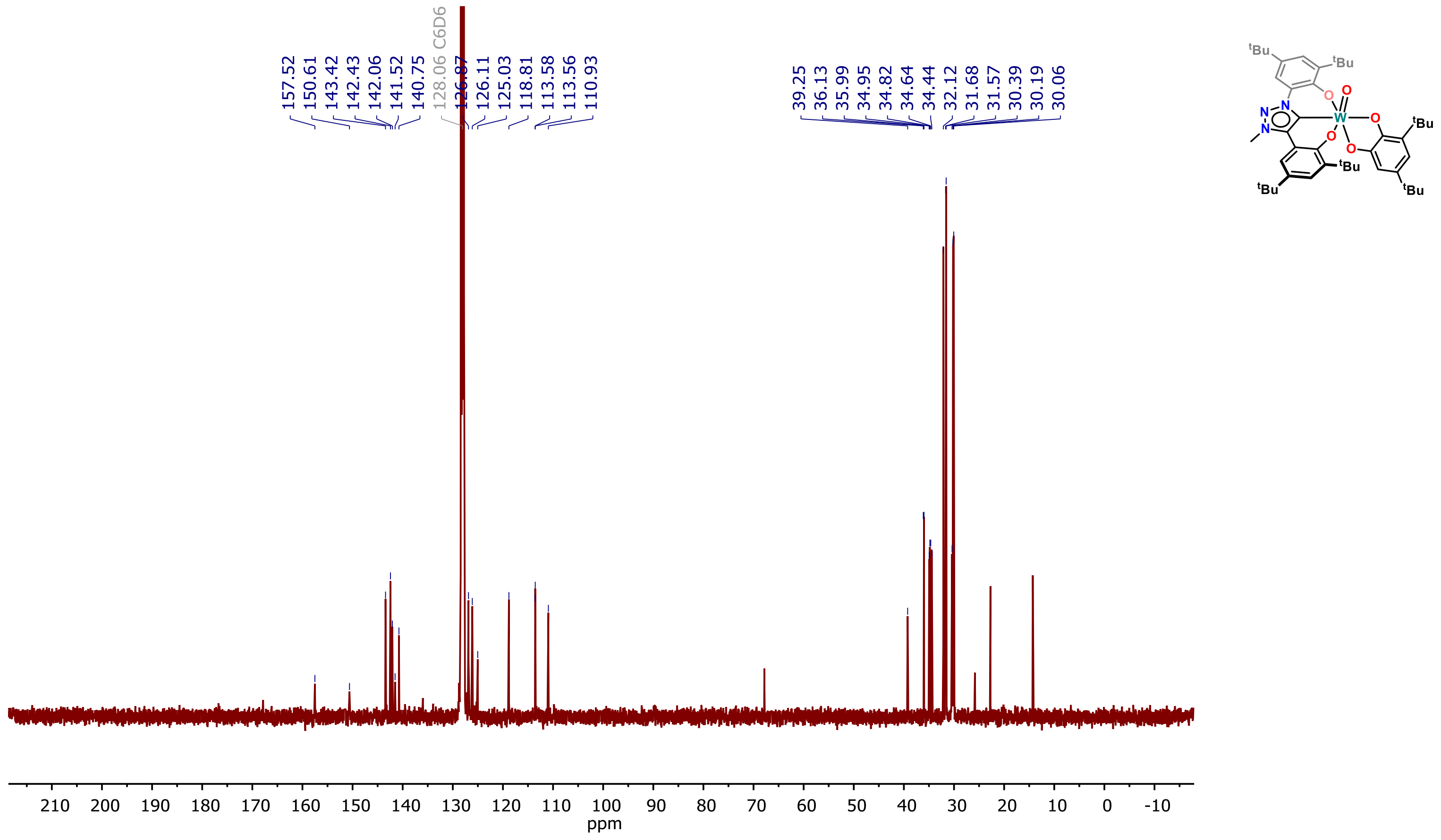

Figure S 32: ^{13}C NMR of **5-W** in C_6D_6 at 298K.

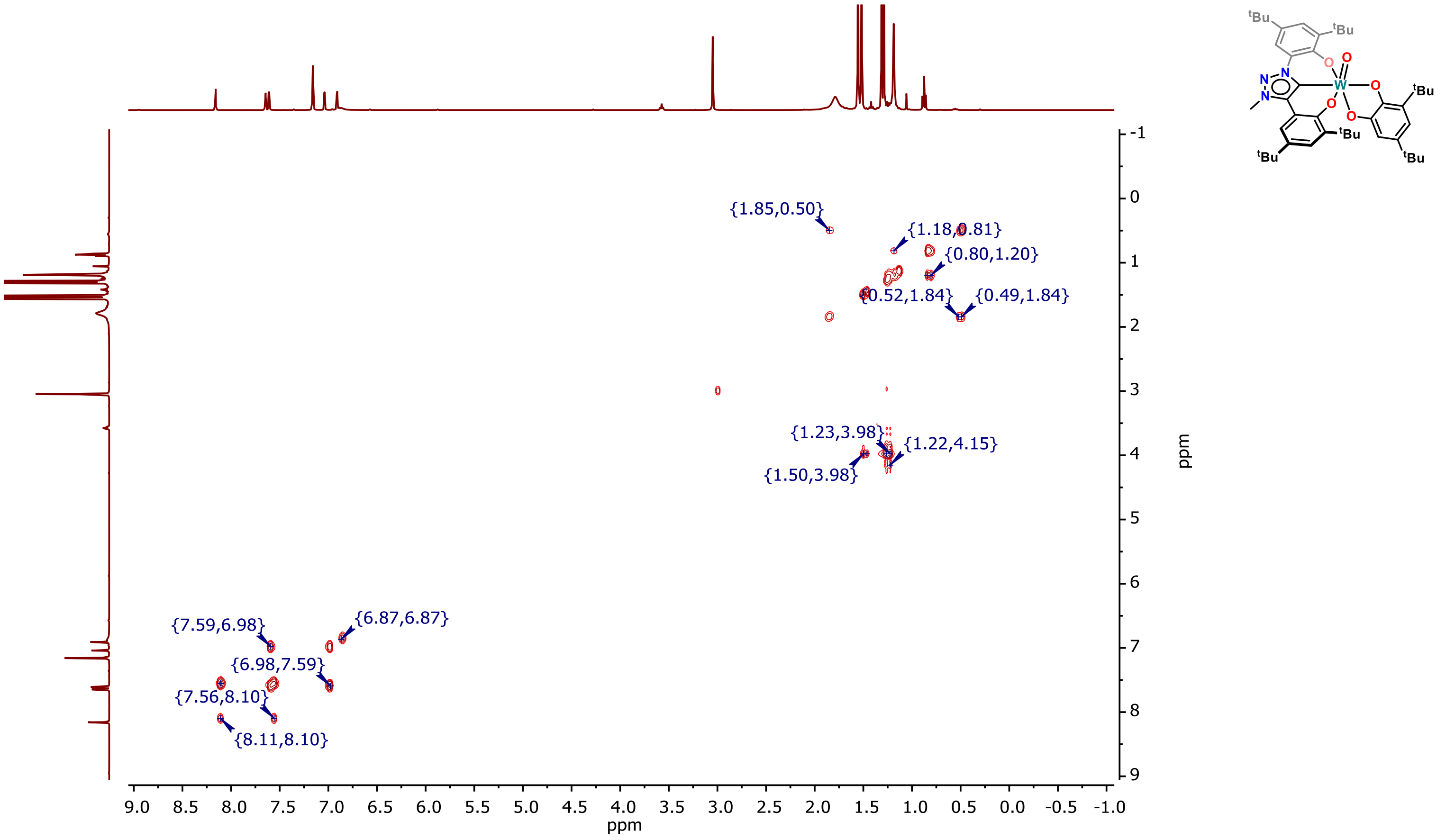

Figure S 33: ^{1}H-^{1}H COSY NMR of **5-W** in C_6D_6 at 298K.

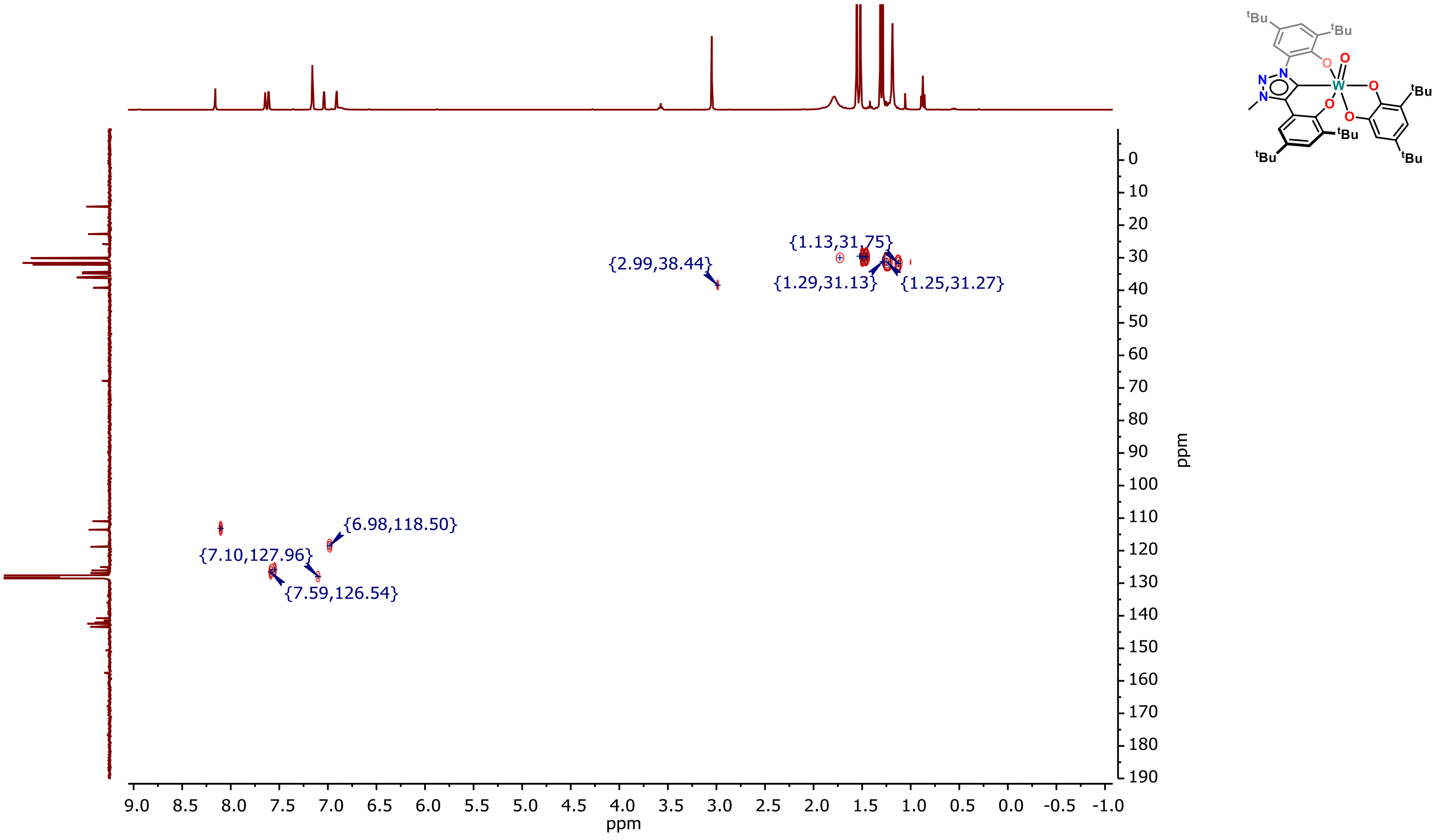

Figure S 34: ^{1}H-^{13}C HSQC NMR of **5-W** in C_6D_6 at 298K.

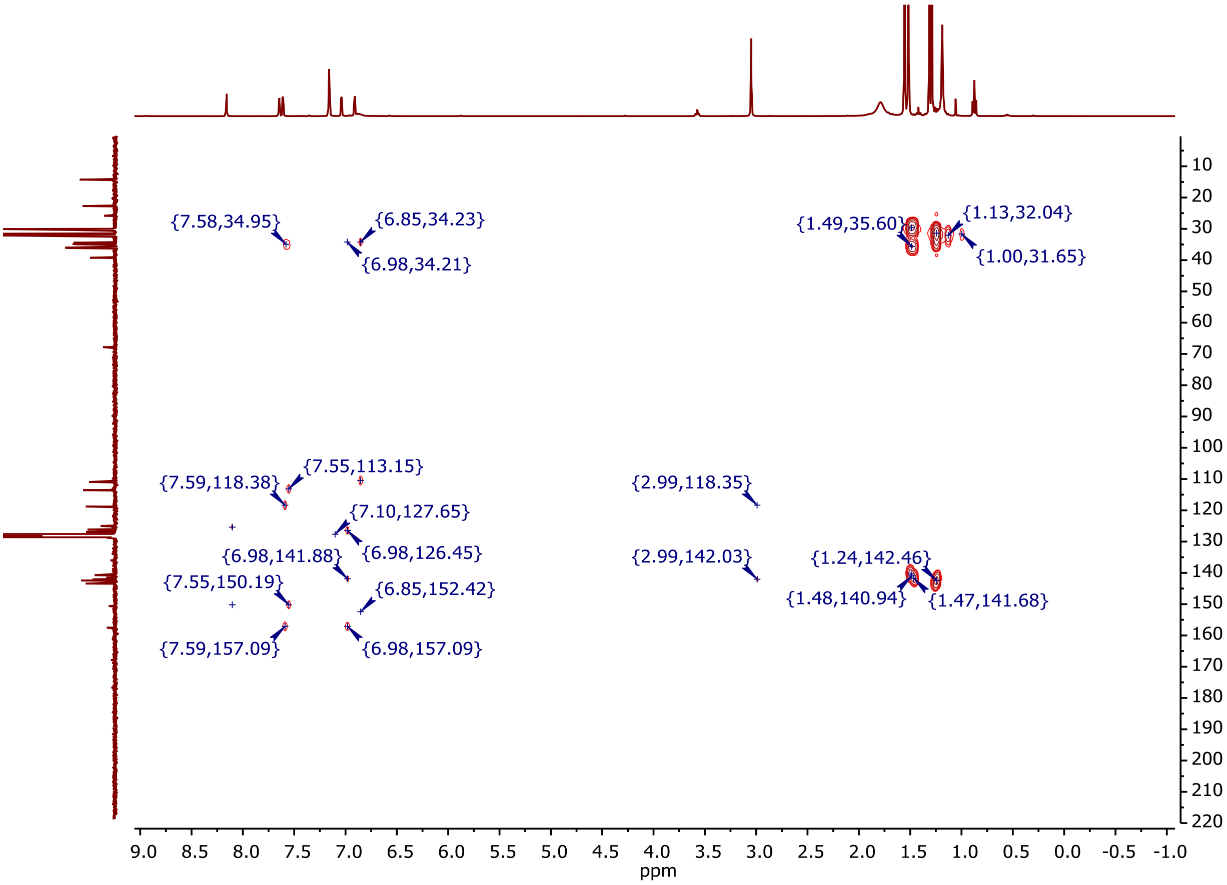

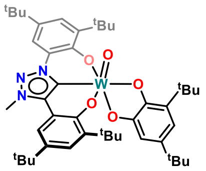

Figure S 35: ^{1}H-^{13}C HMBC NMR of **5-W** in C_6D_6 at 298K.

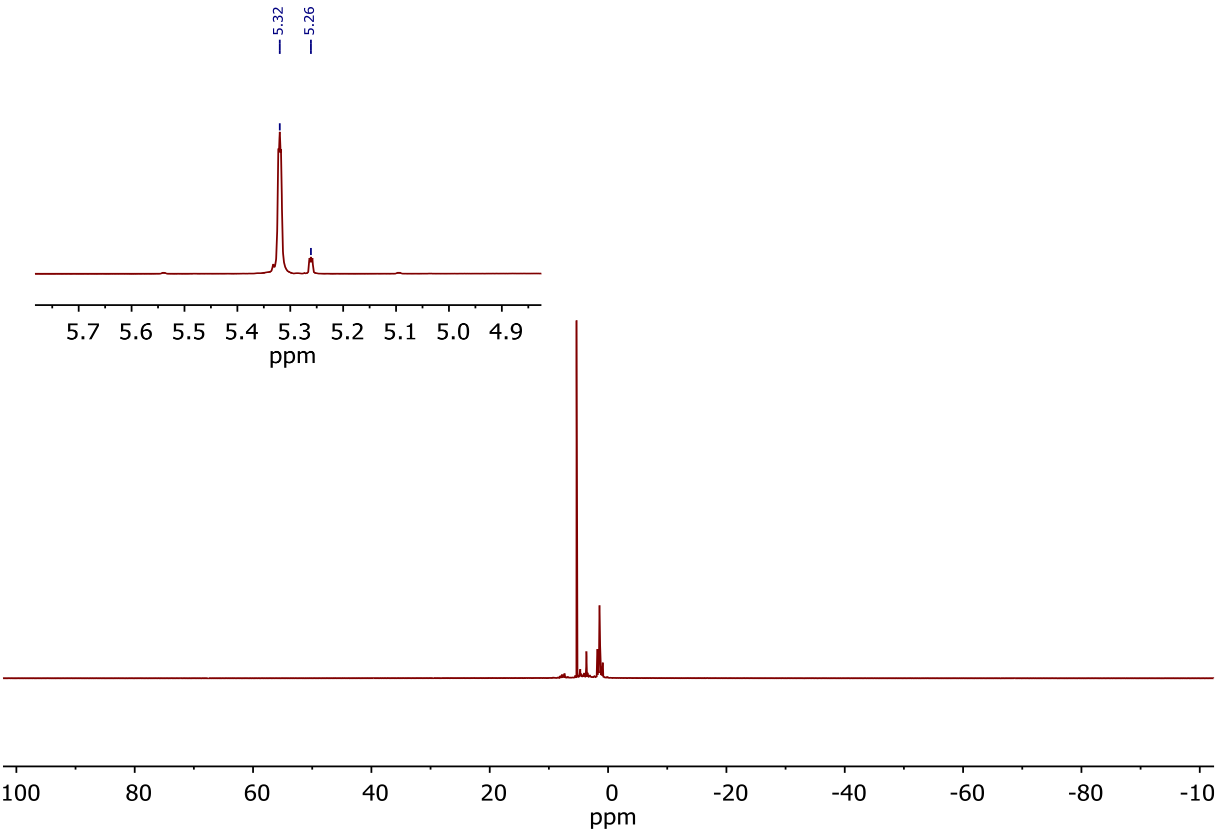

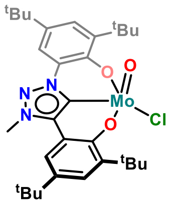

Figure S 36: Evans NMR of **6a-Mo** in CD_2Cl_2 at 298K.

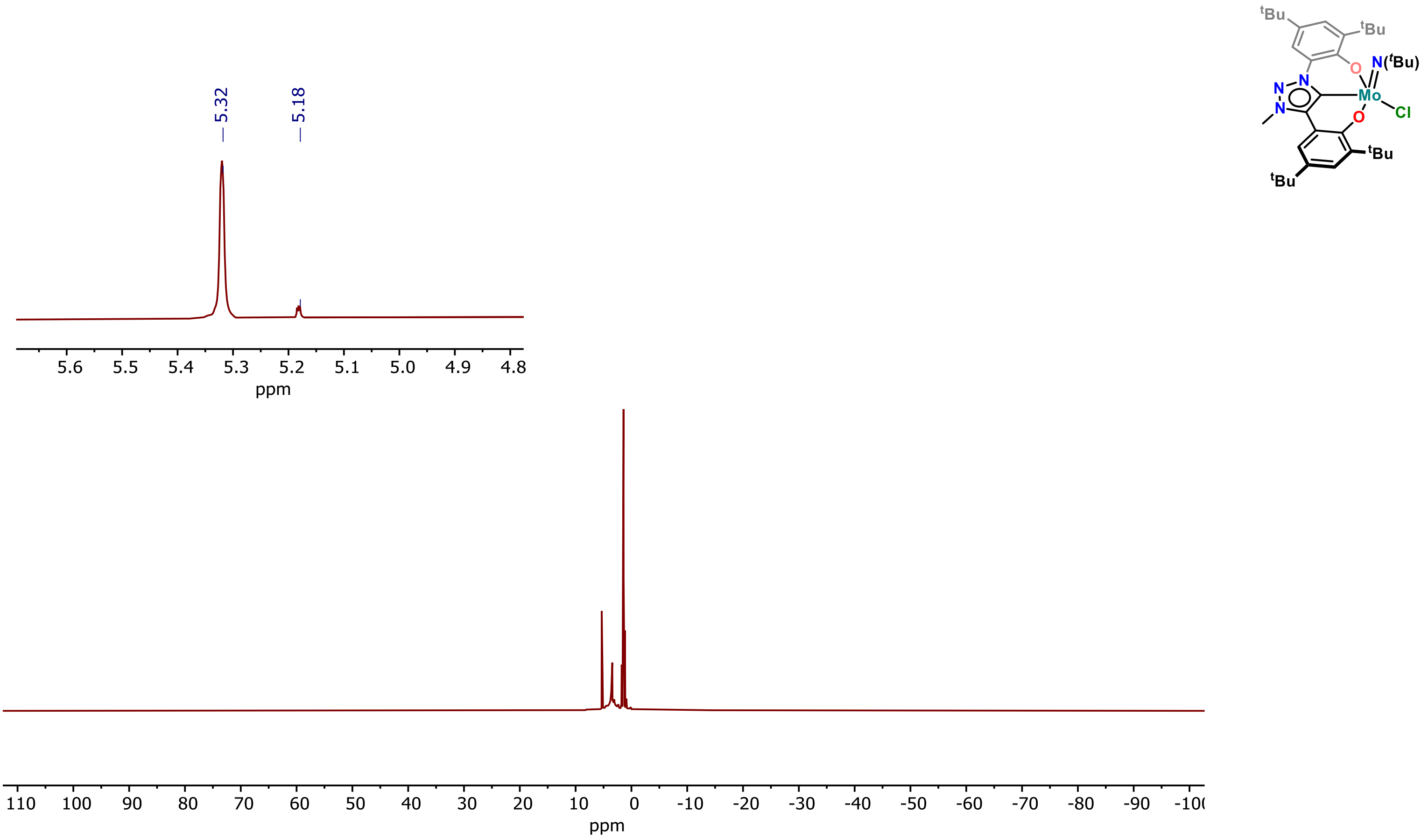

Figure S 37: Evans NMR of **6b-Mo** in CD_2Cl_2 at 298K.

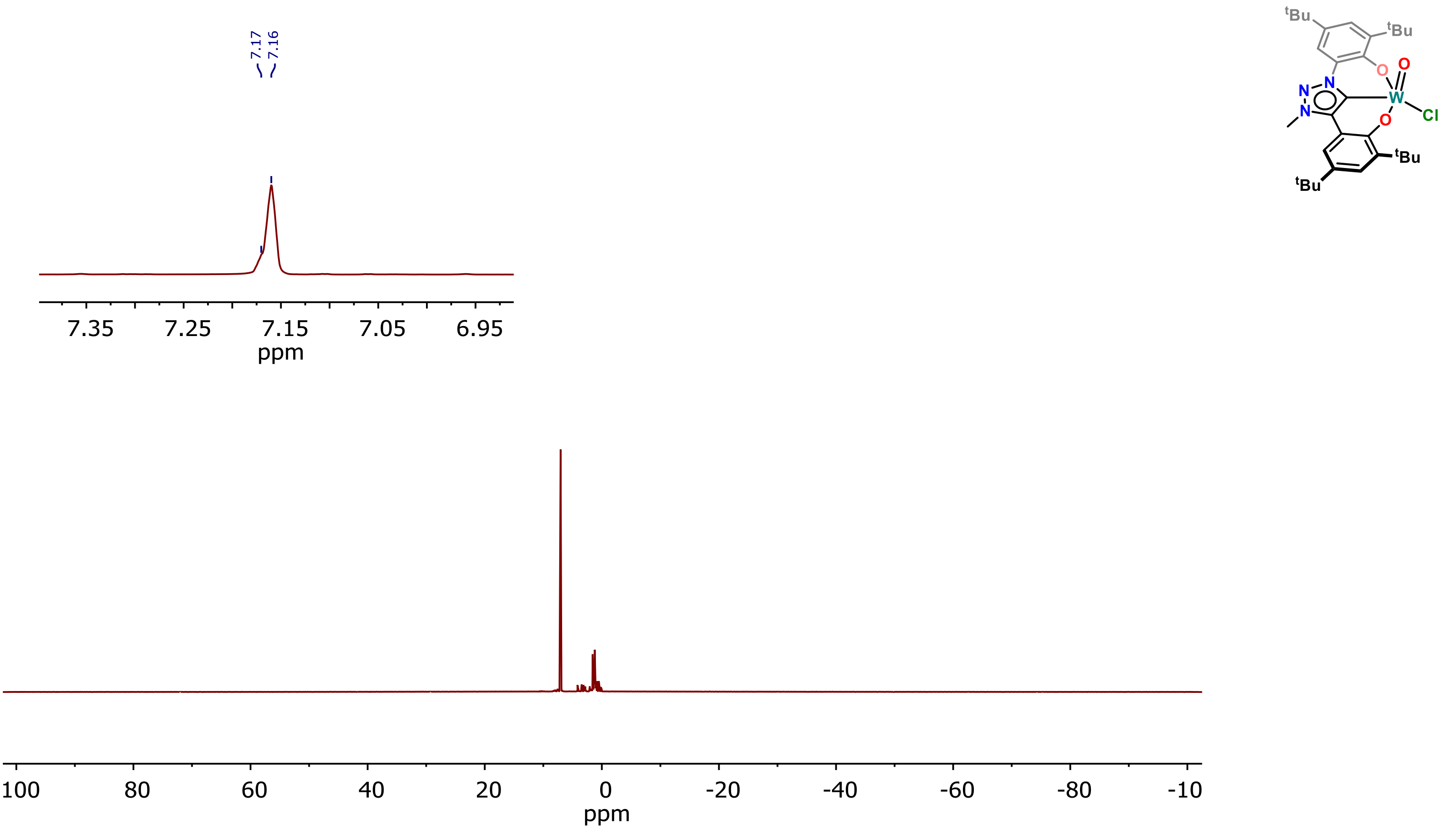

Figure S 38: Evans NMR of **6a-W** in C_6D_6 at 298K.

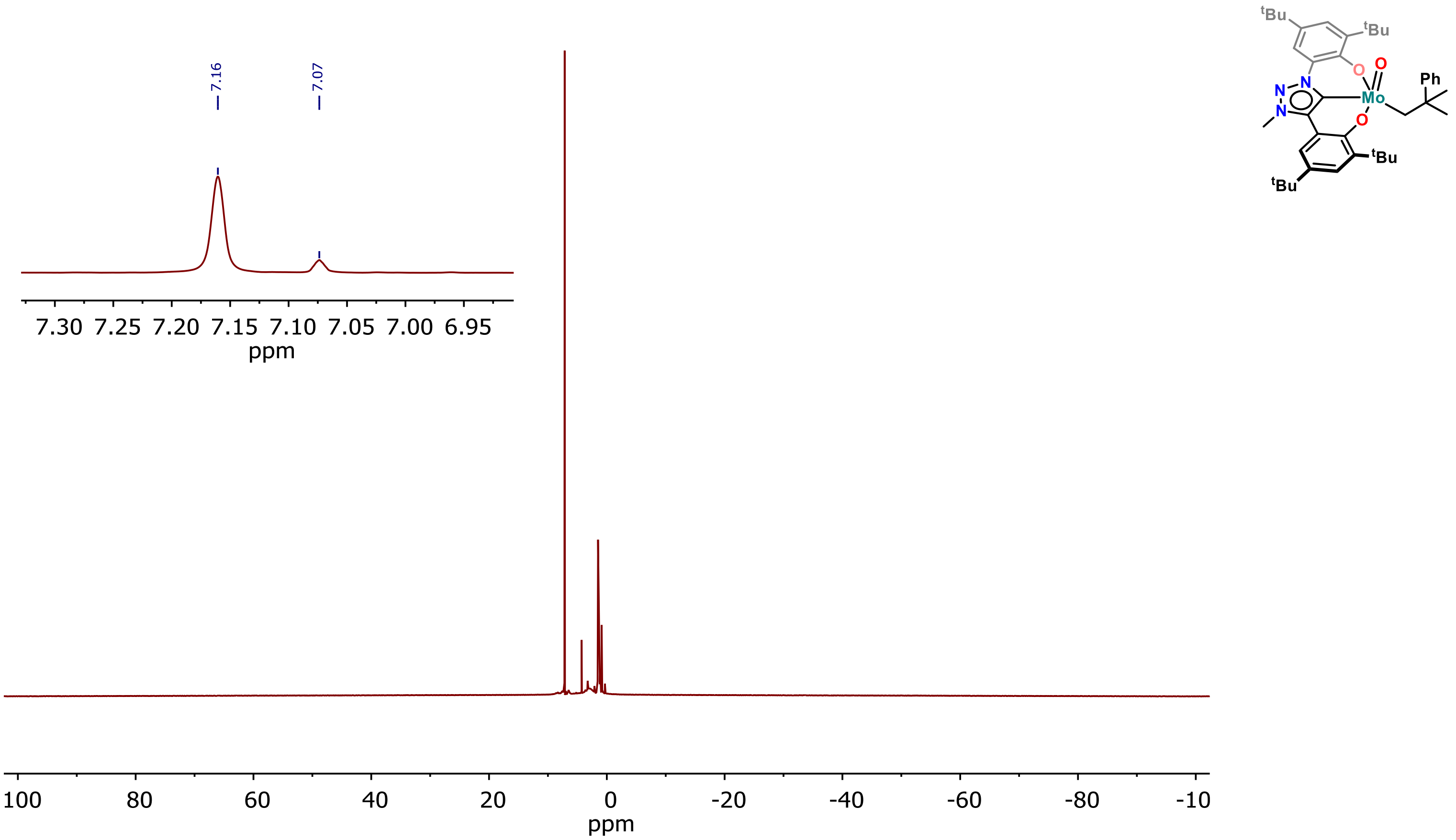

Figure S 39: Evans NMR of **7a** in C_6D_6 at 298K.

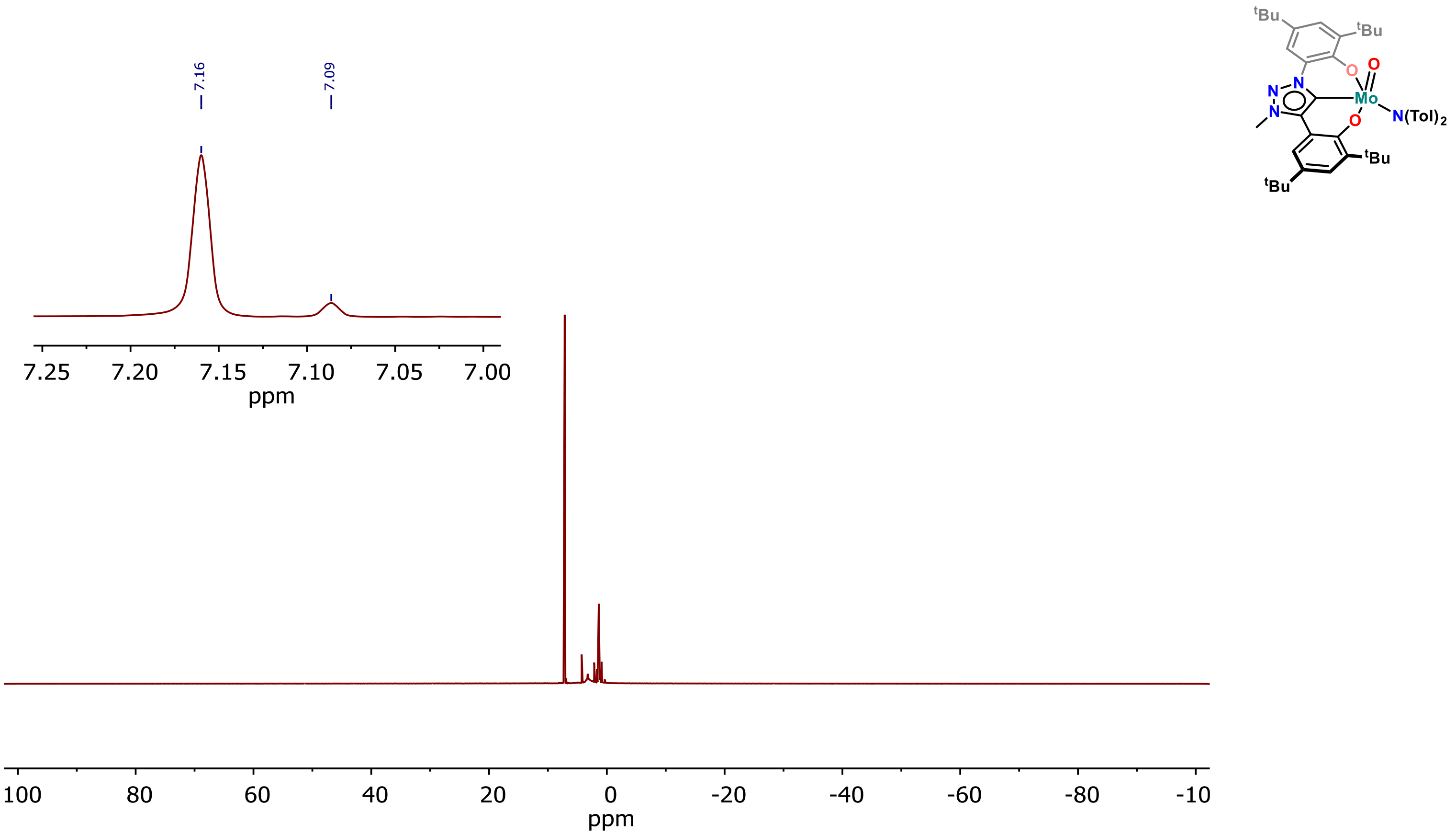

Figure S 40: Evans NMR of **7c** in C_6D_6 at 298K.

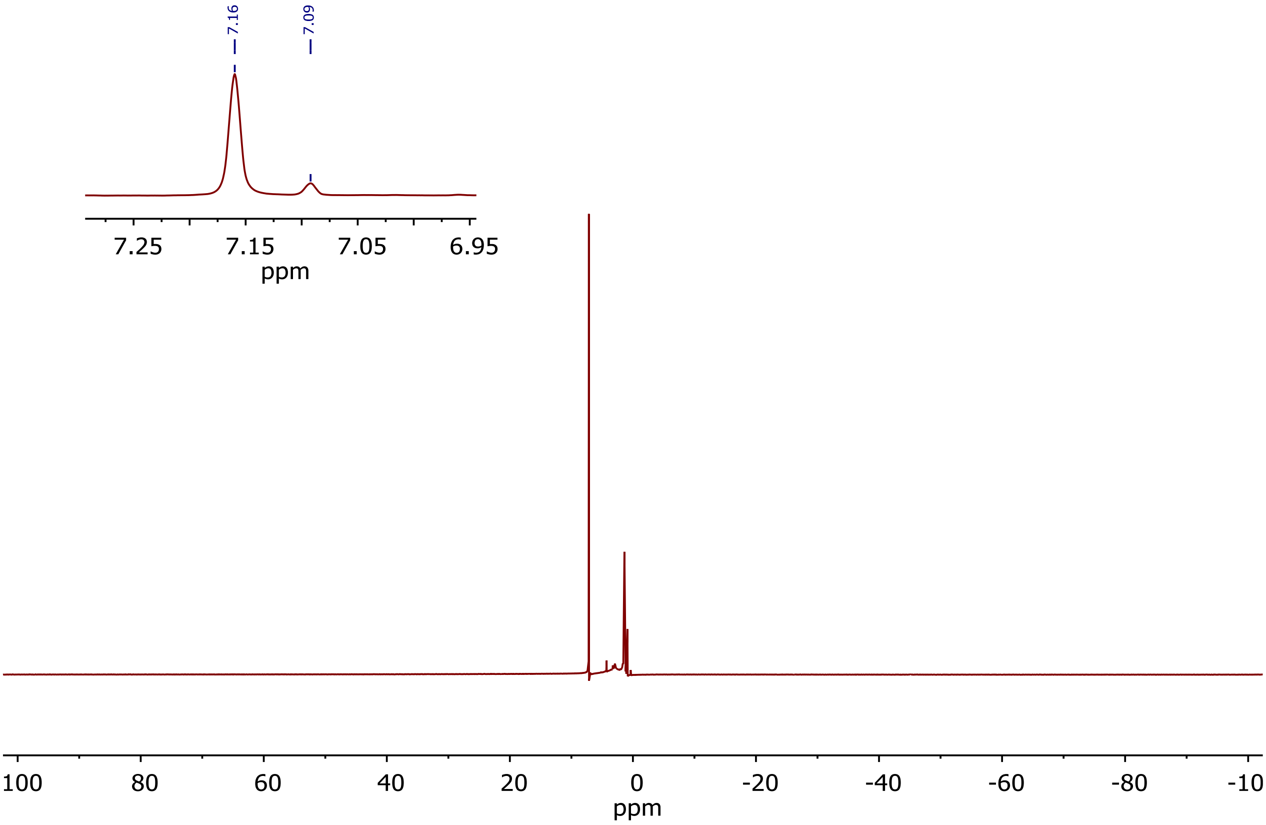

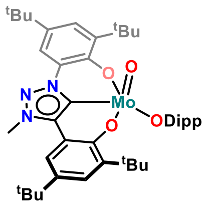

Figure S 41: Evans NMR of **7b** in C_6D_6 at 298K.

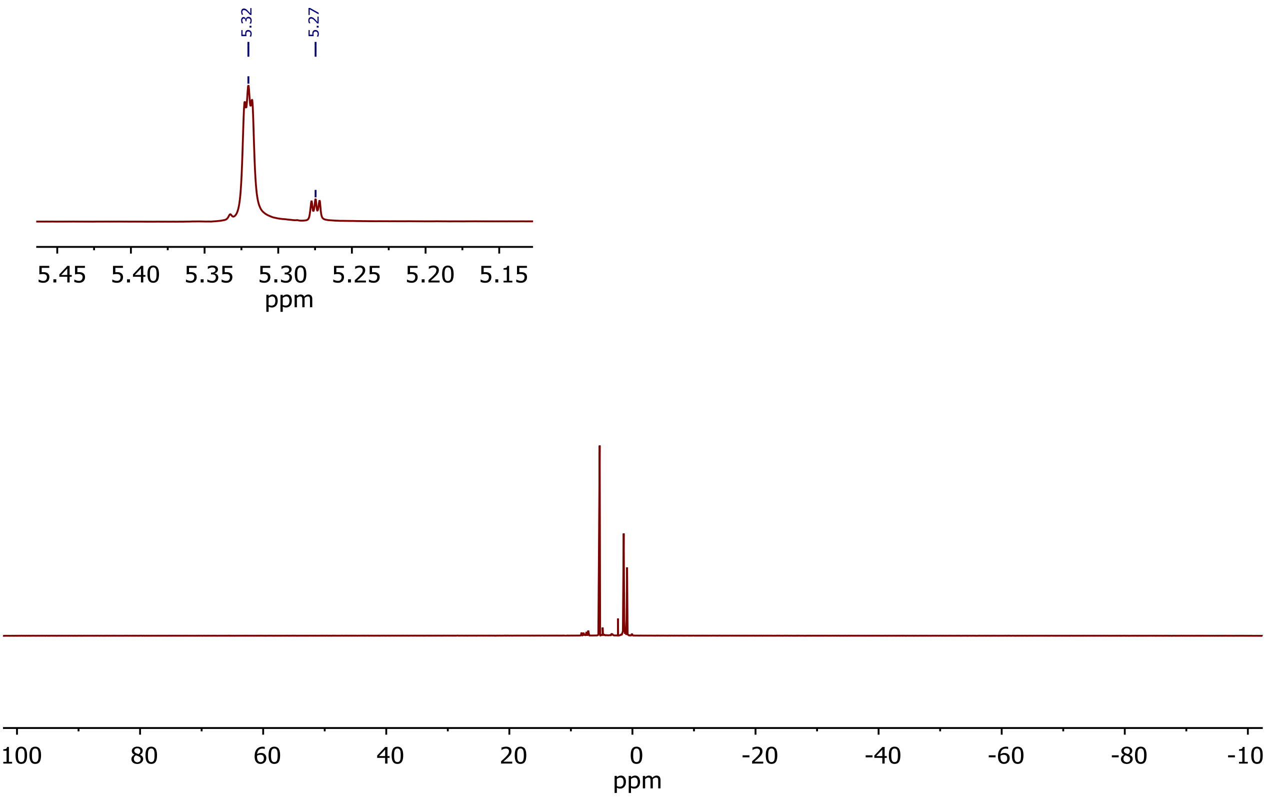

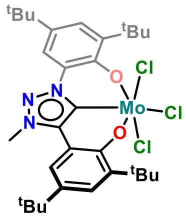

Figure S 42: Evans NMR of **8-Mo** in CD_2Cl_2 at 298K. Signals in the aromatic region most likely belong to unknown impurities.

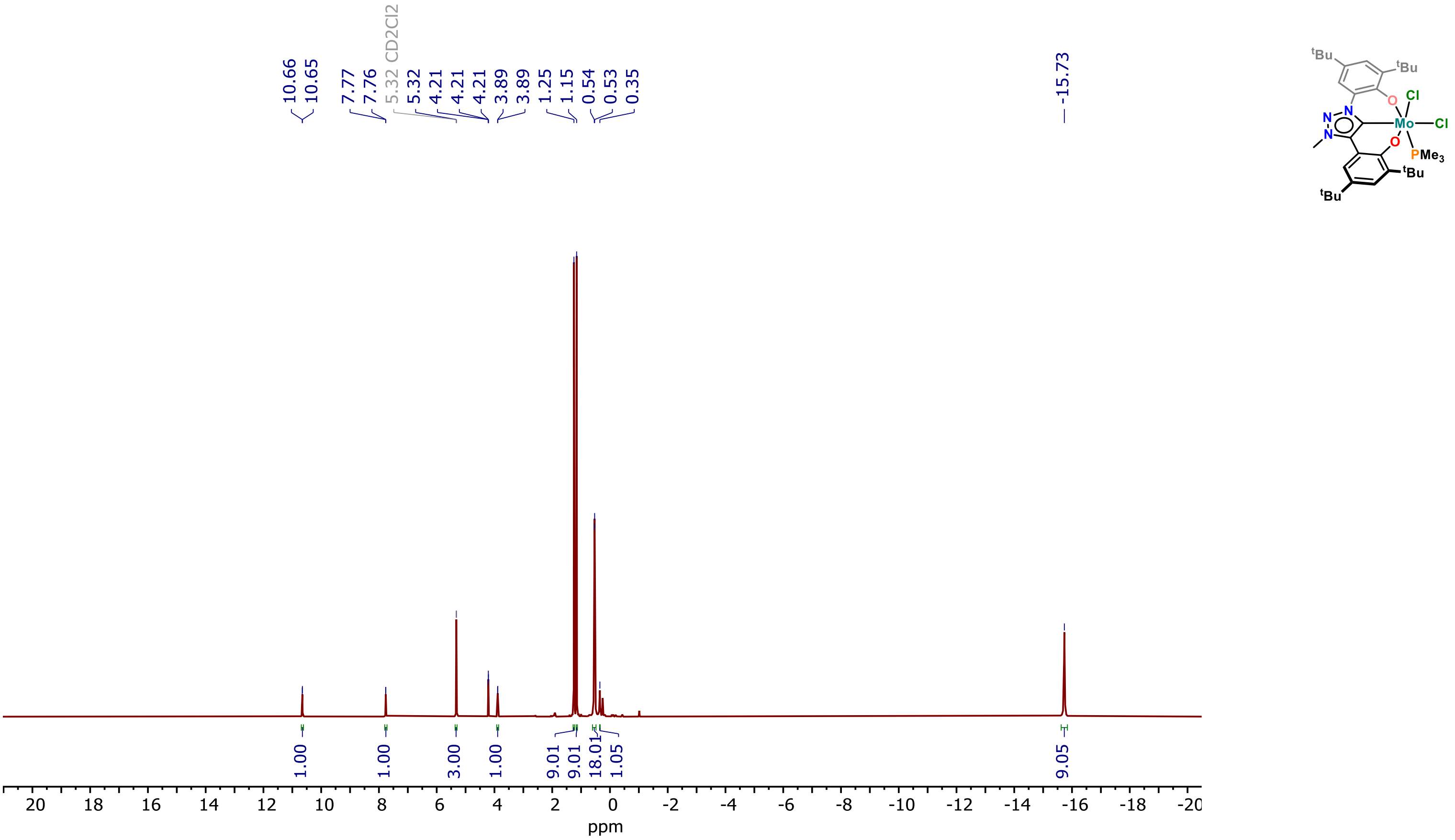

Figure S 43: ^{1}H-NMR spectrum of **9-Mo** in dichloromethane-d_2 at 298 K.

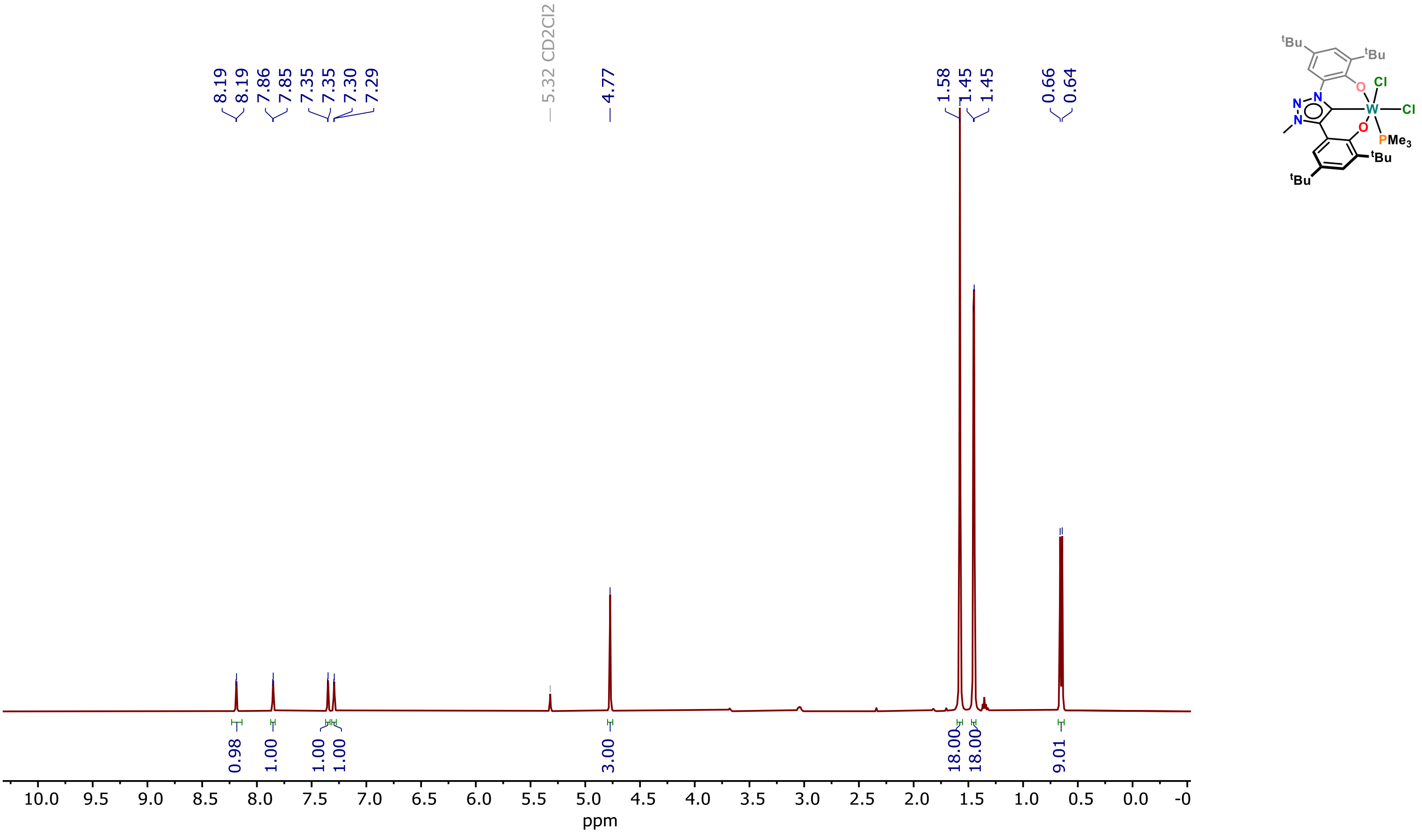

Figure S 44: ^{1}H-NMR spectrum of **9-W** in dichloromethane-d_2 at 298 K.

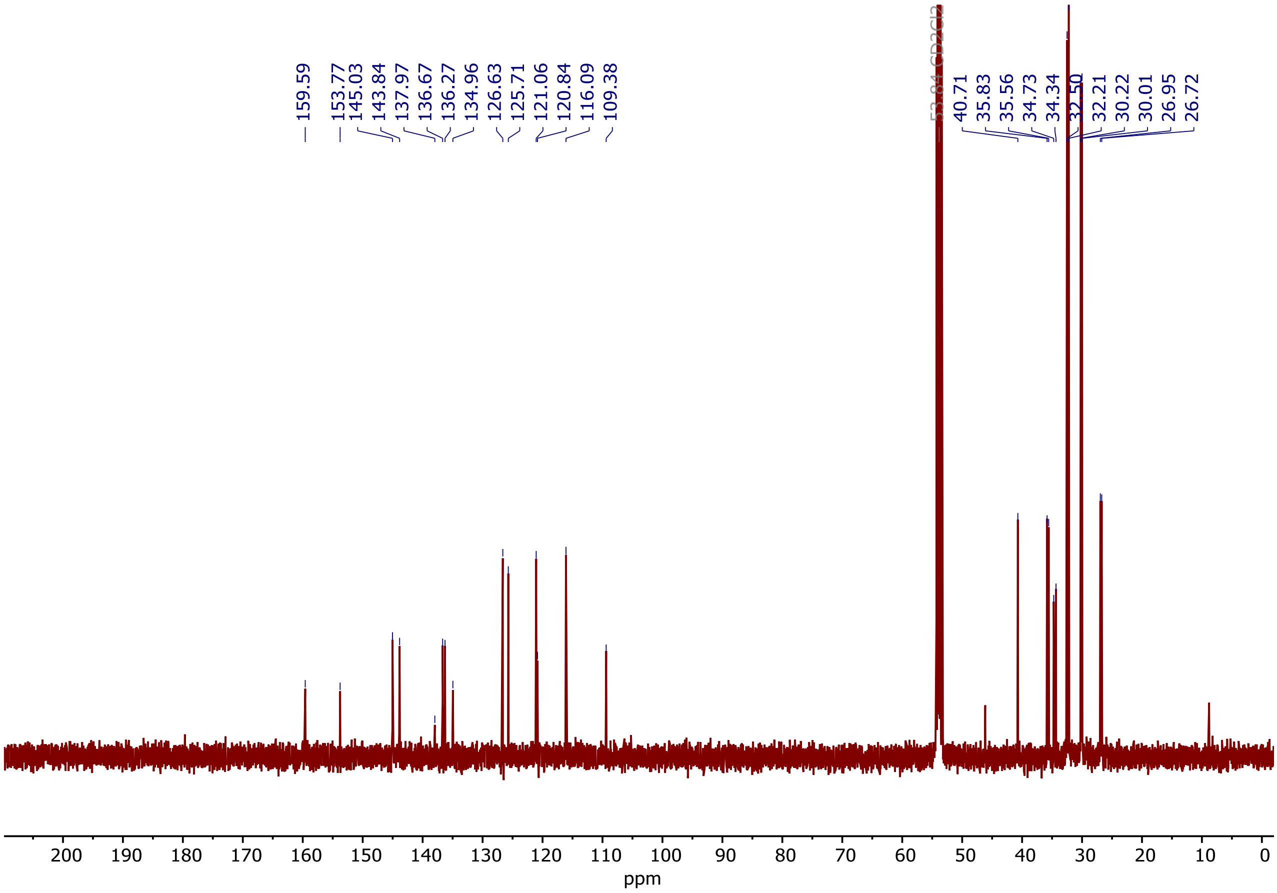

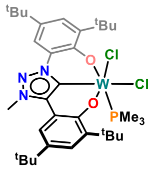

Figure S 45: ^{13}C{H}-NMR spectrum of **9-W** in dichloromethane-d_2 at 298 K.

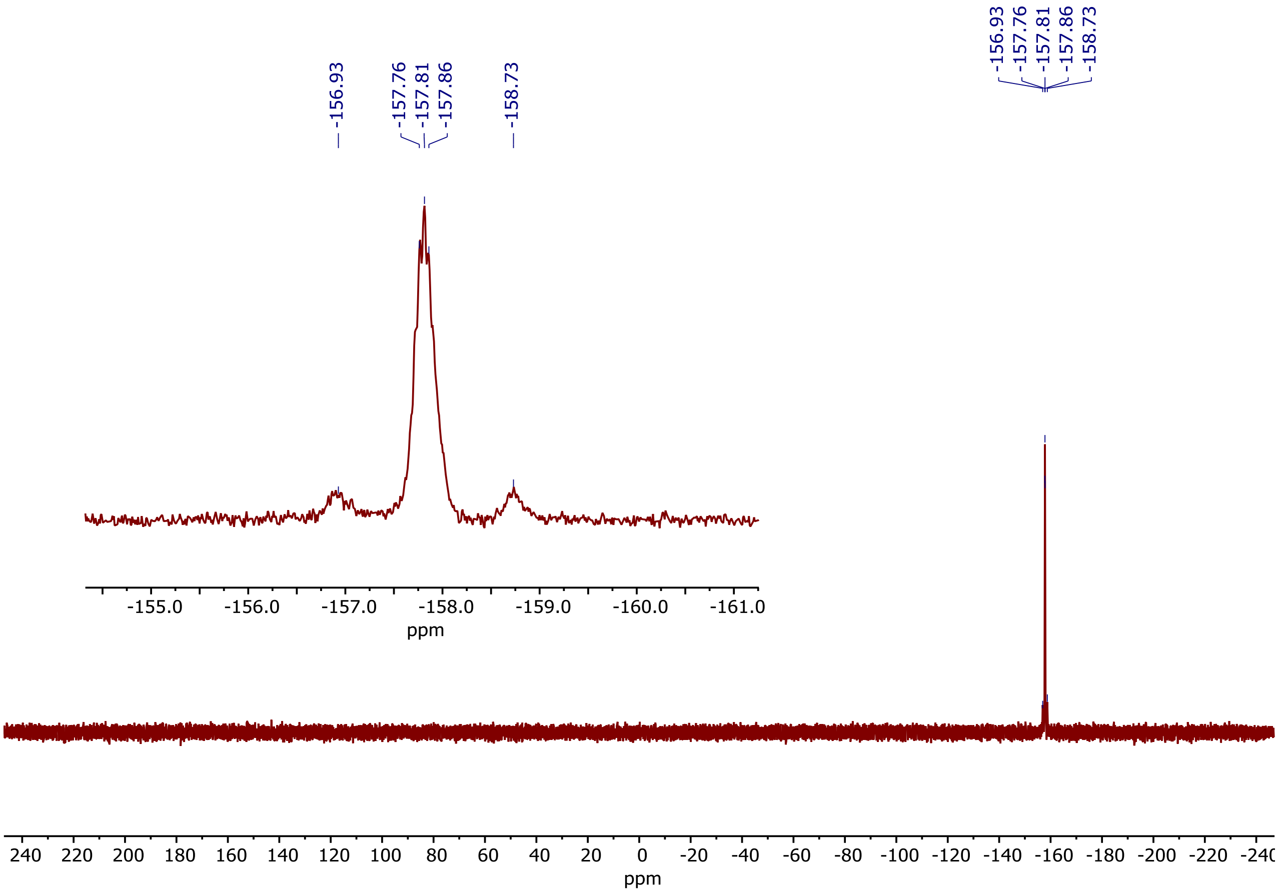

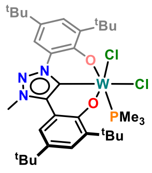

Figure S 46: ^{31}P-NMR spectrum of **9-W** in dichloromethane-d_2 at 298 K.

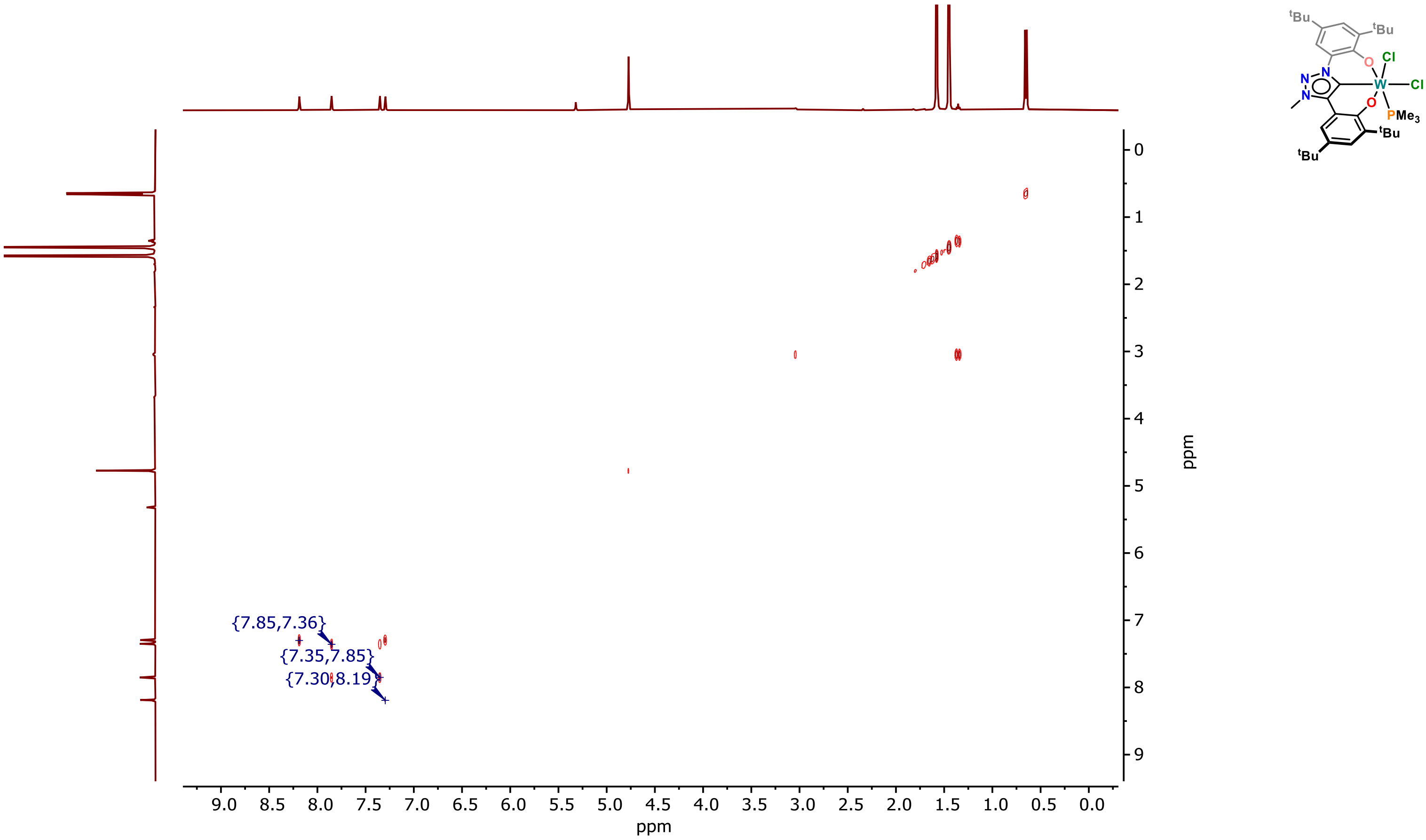

Figure S 47: ^{1}H-^{1}H COSY NMR of **9-W** in dichloromethane-d_2 at 298 K.

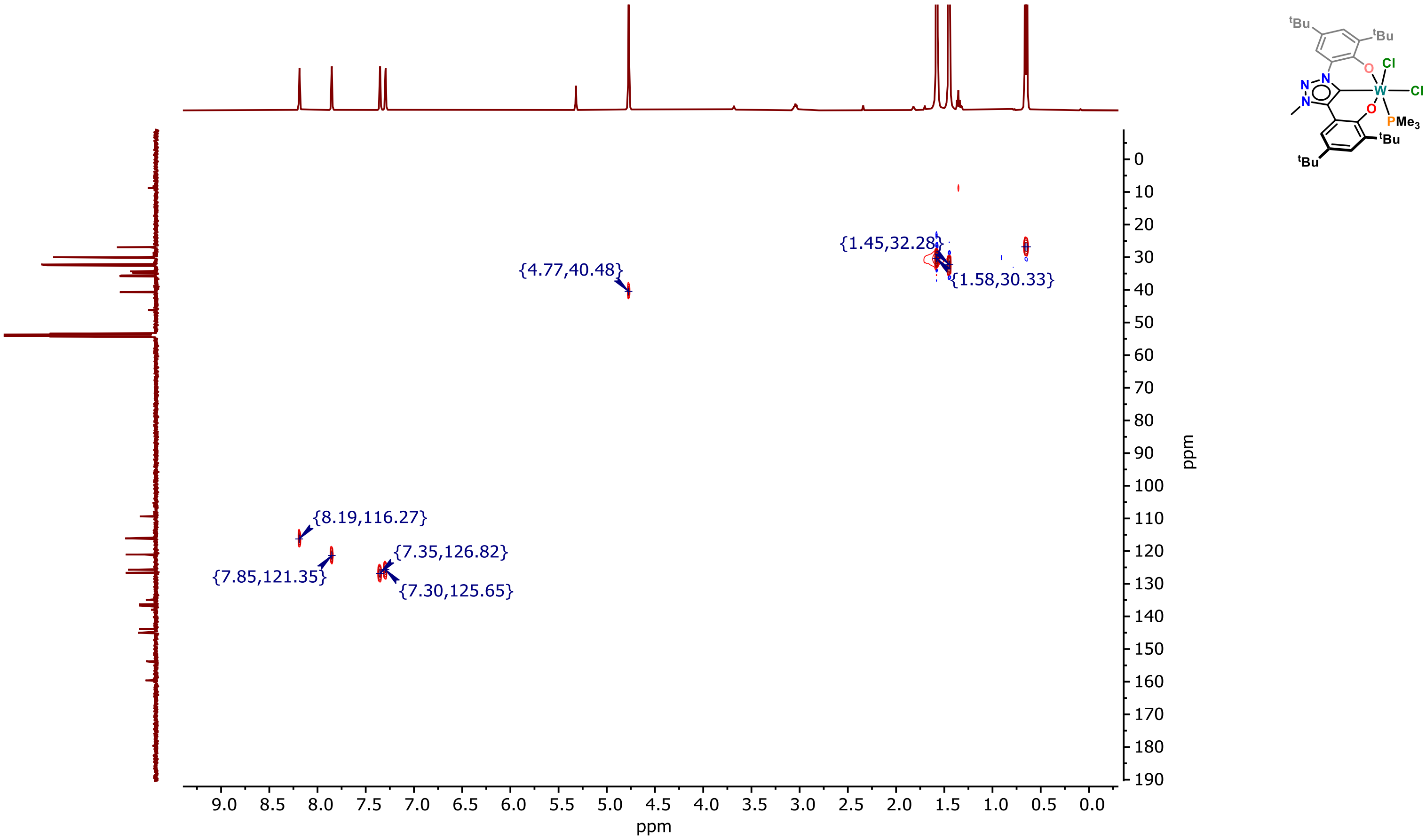

Figure S 48: HSQC-spectrum of **9-W** in dichloromethane-d_2 at 298 K.

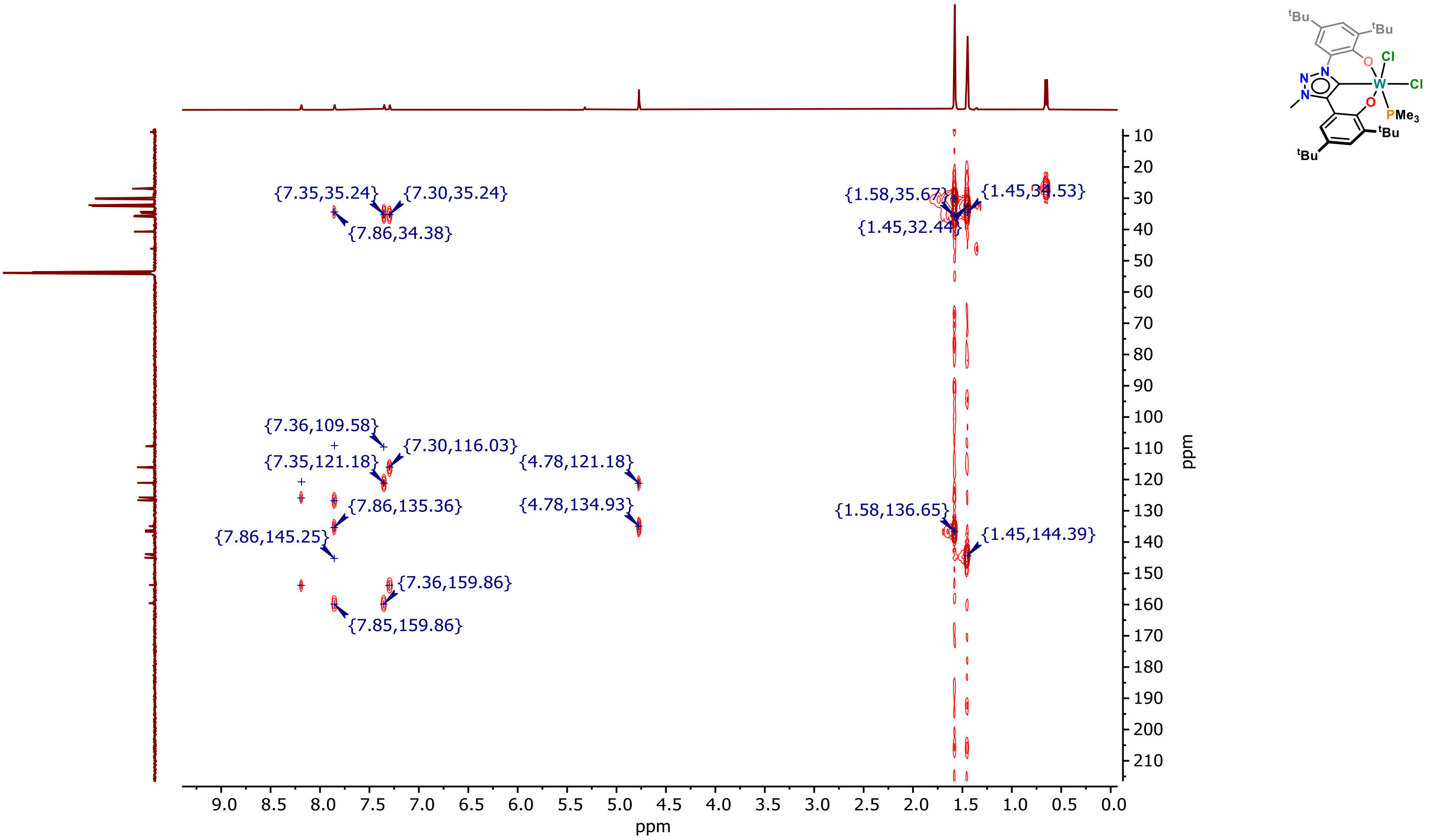

Figure S 49: HMBC-spectrum of **9-W** in dichloromethane-d_2 at 298 K.

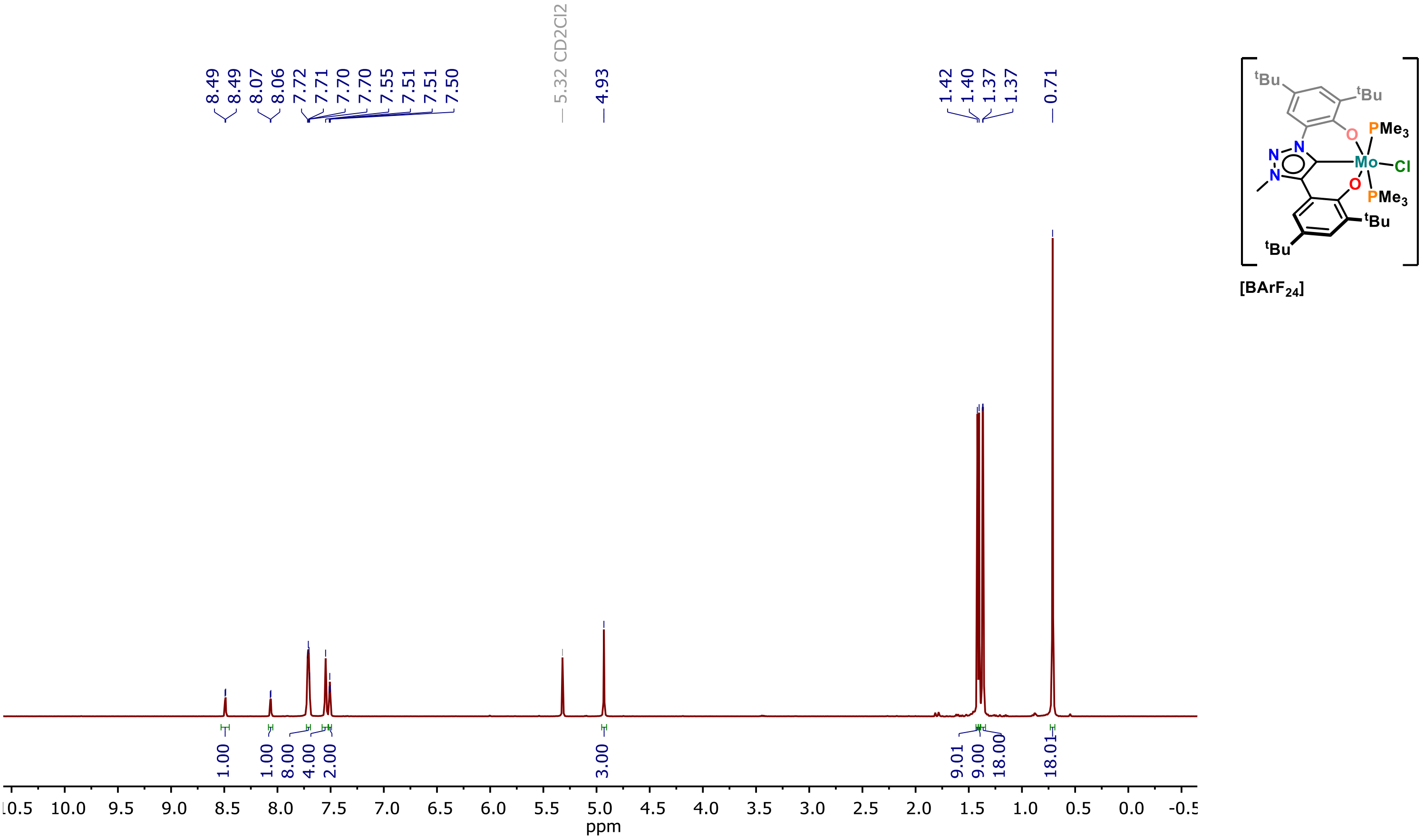

Figure S 50: ^{1}H NMR of **11-Mo** in C_6D_6 at 298K.

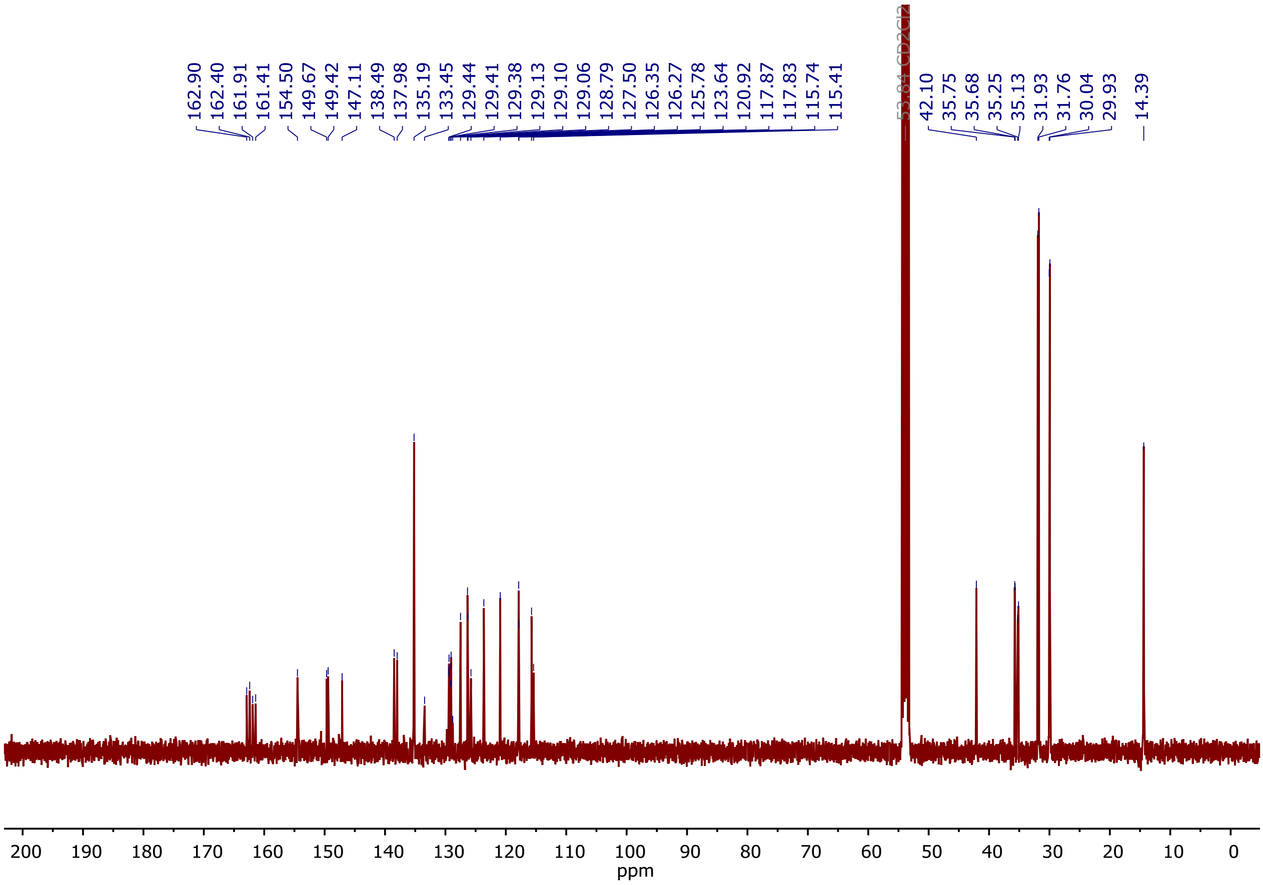

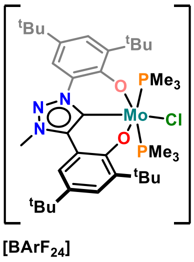

Figure S 51: ^{13}C NMR of **11-Mo** in C_6D_6 at 298K.

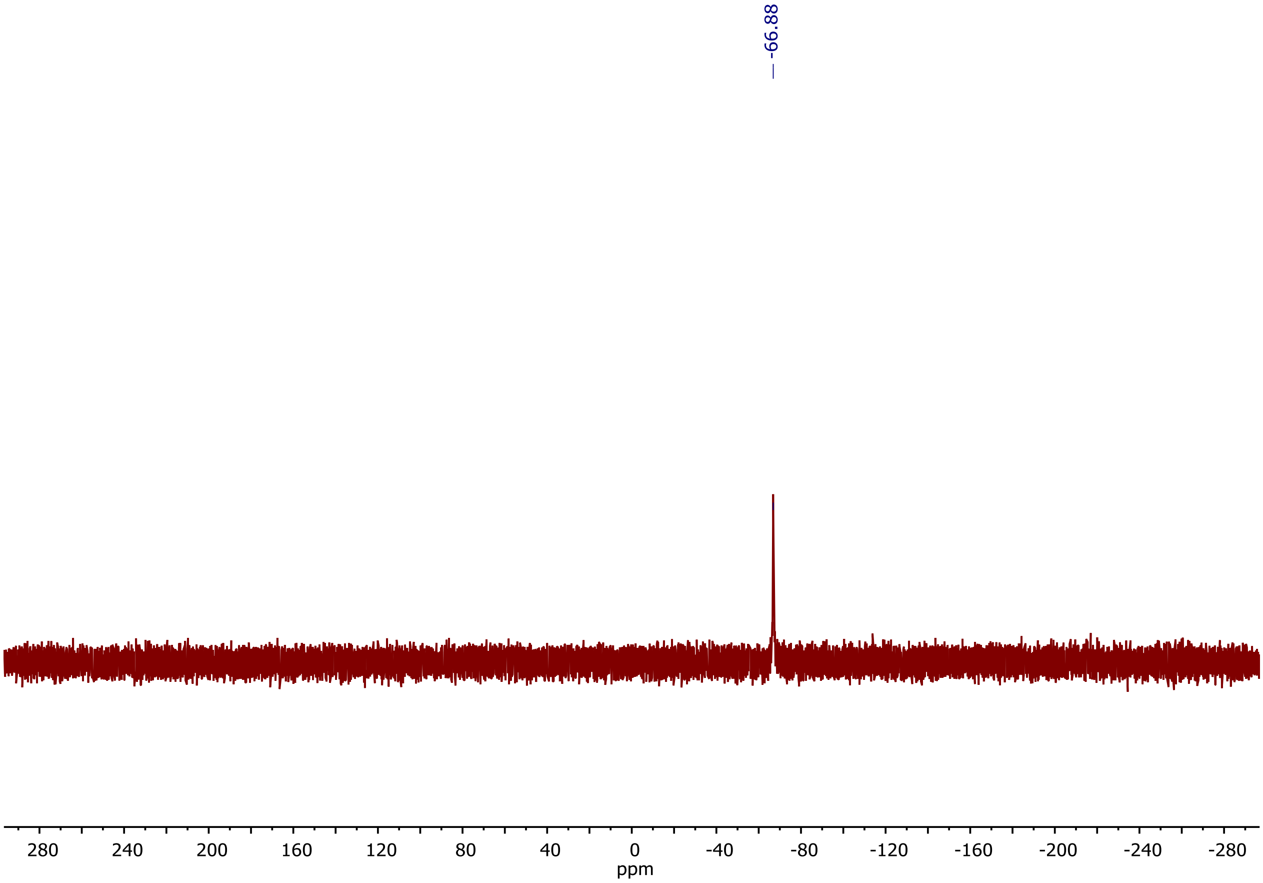

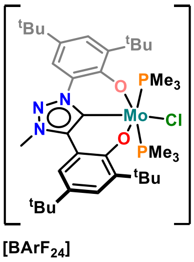

Figure S 52: ^{31}P NMR of **11-Mo** in C_6D_6 at 298K.

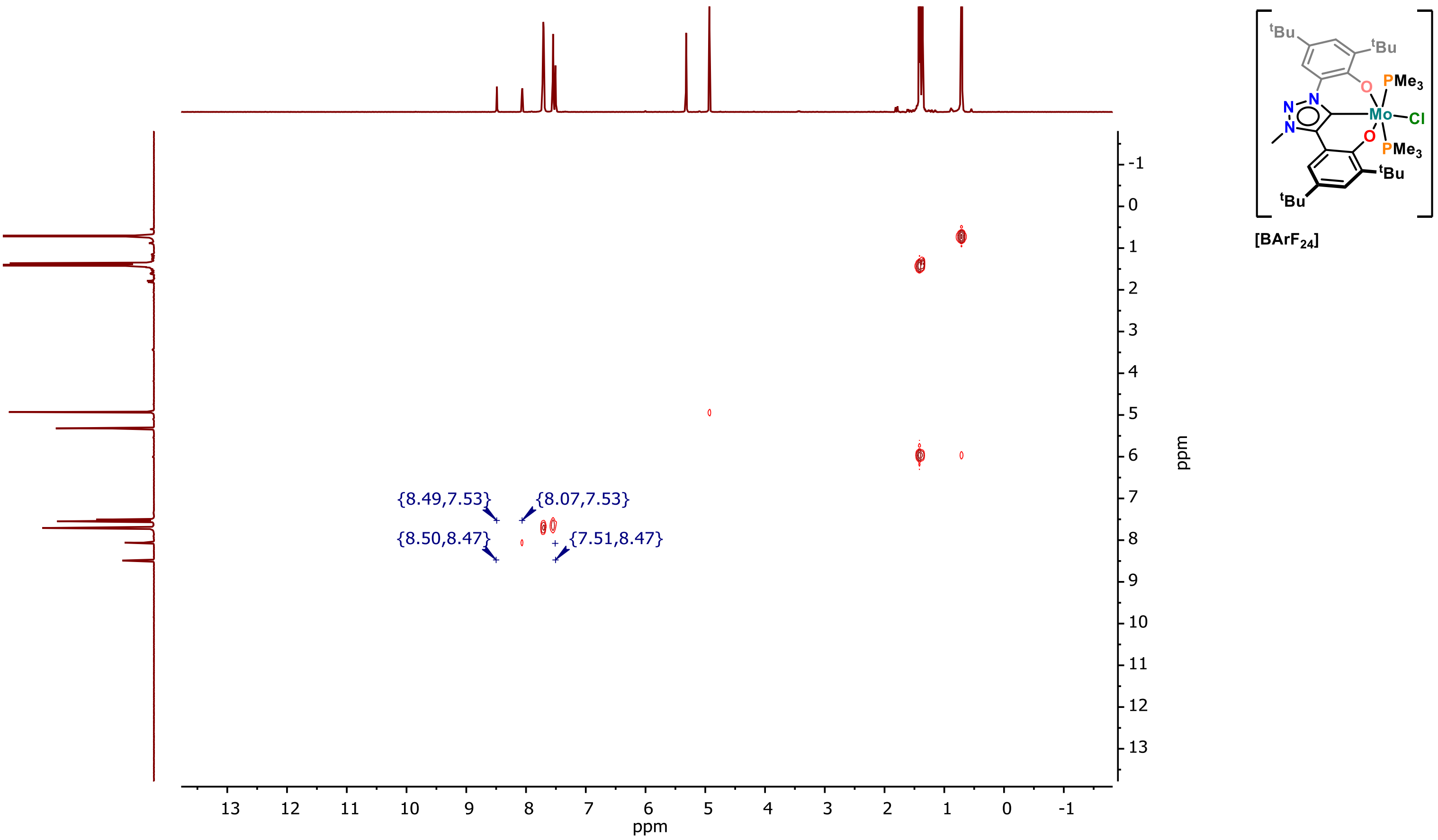

Figure S 53: ^{1}H-^{1}H COSY NMR of **11-Mo** in C_6D_6 at 298K.

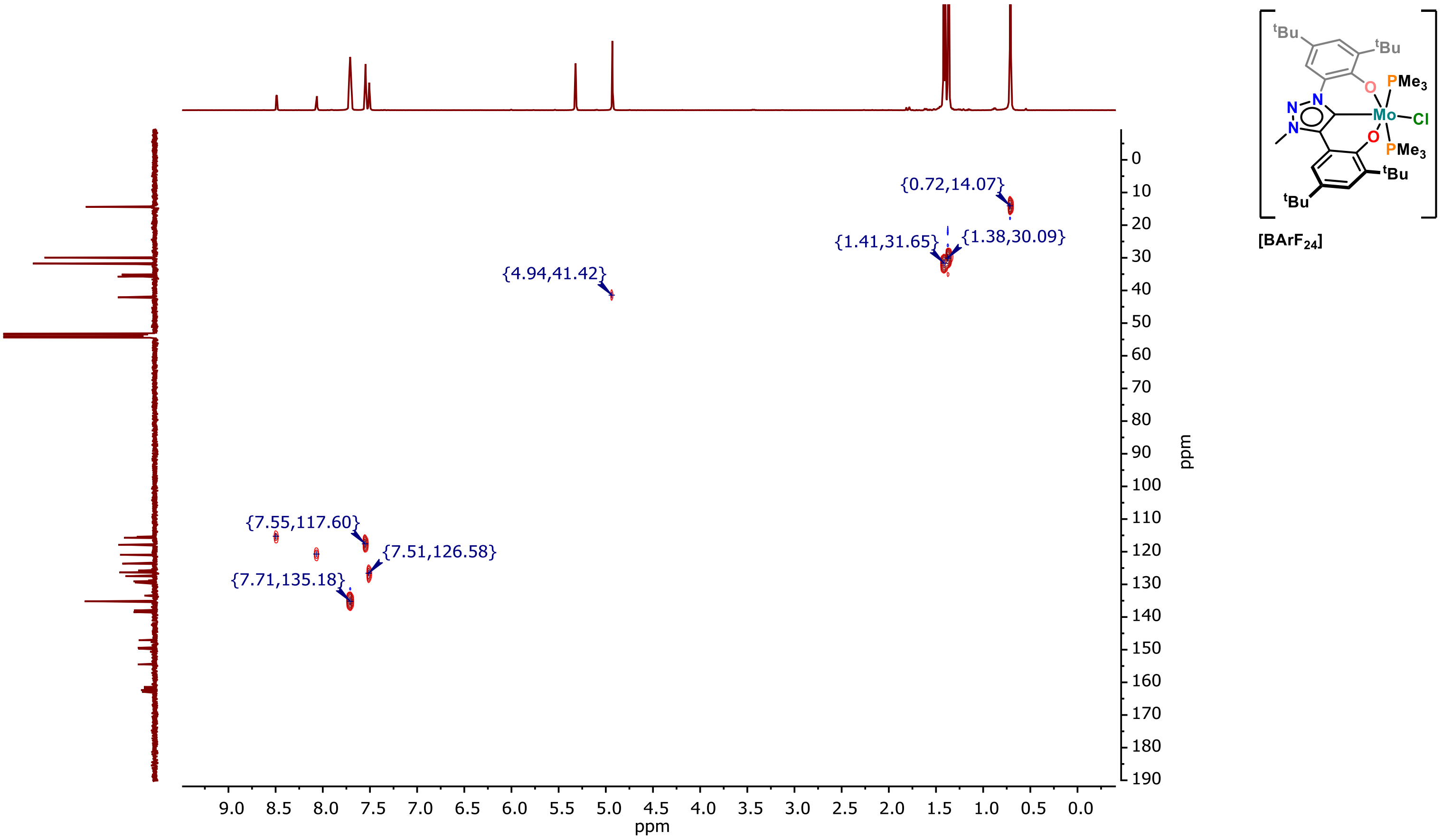

Figure S 54: ^{1}H-^{13}C HSQC NMR of **11-Mo** in C_6D_6 at 298K.

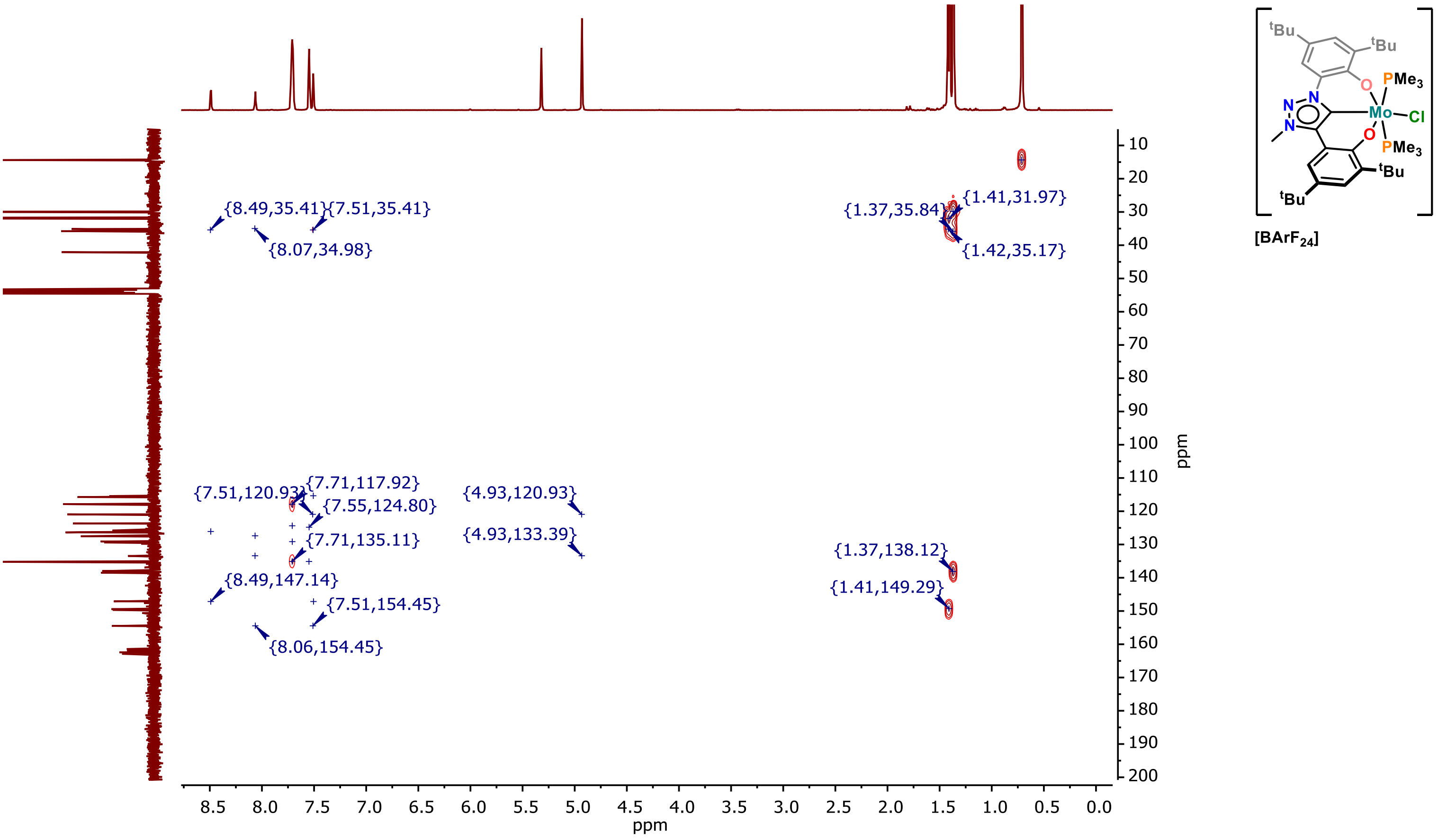

Figure S 55: ^{1}H-^{13}C HMBC NMR of **11-Mo** in C_6D_6 at 298K.

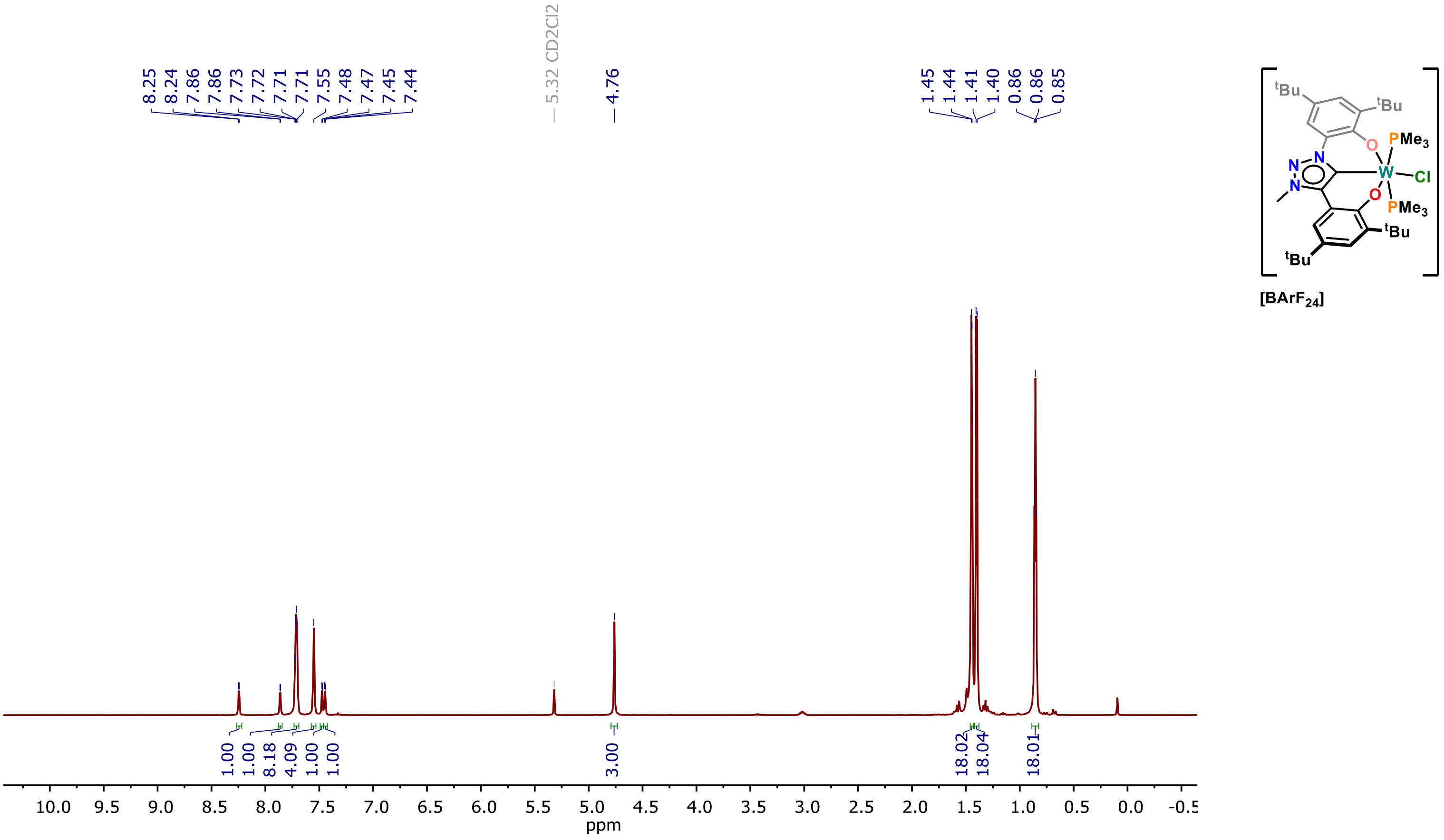

Figure S 56: ^{1}H NMR of **11-W** in C_6D_6 at 298K.

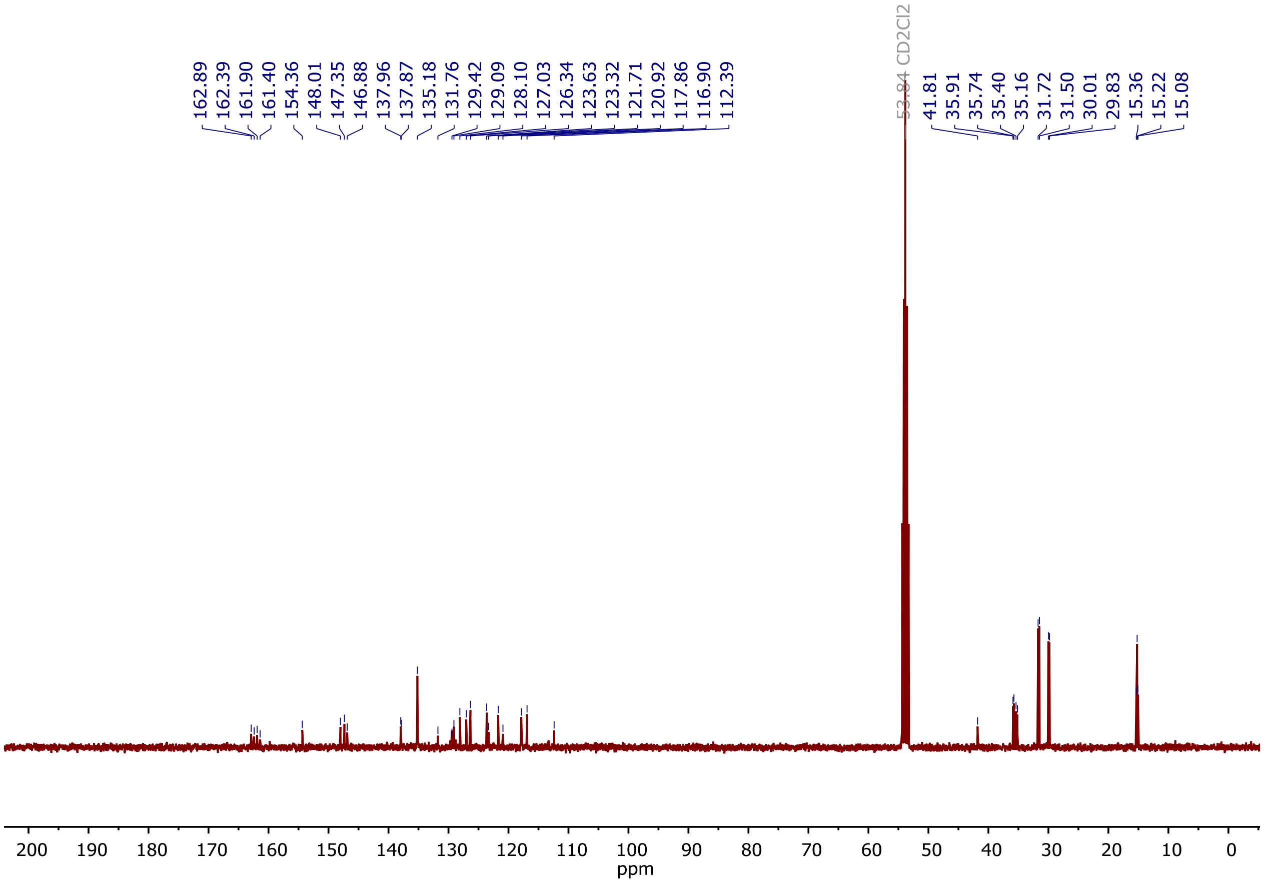

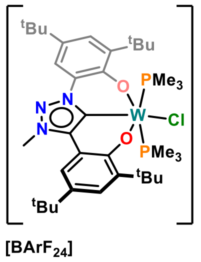

Figure S 57: ^{13}C NMR of **11-W** in C_6D_6 at 298K.

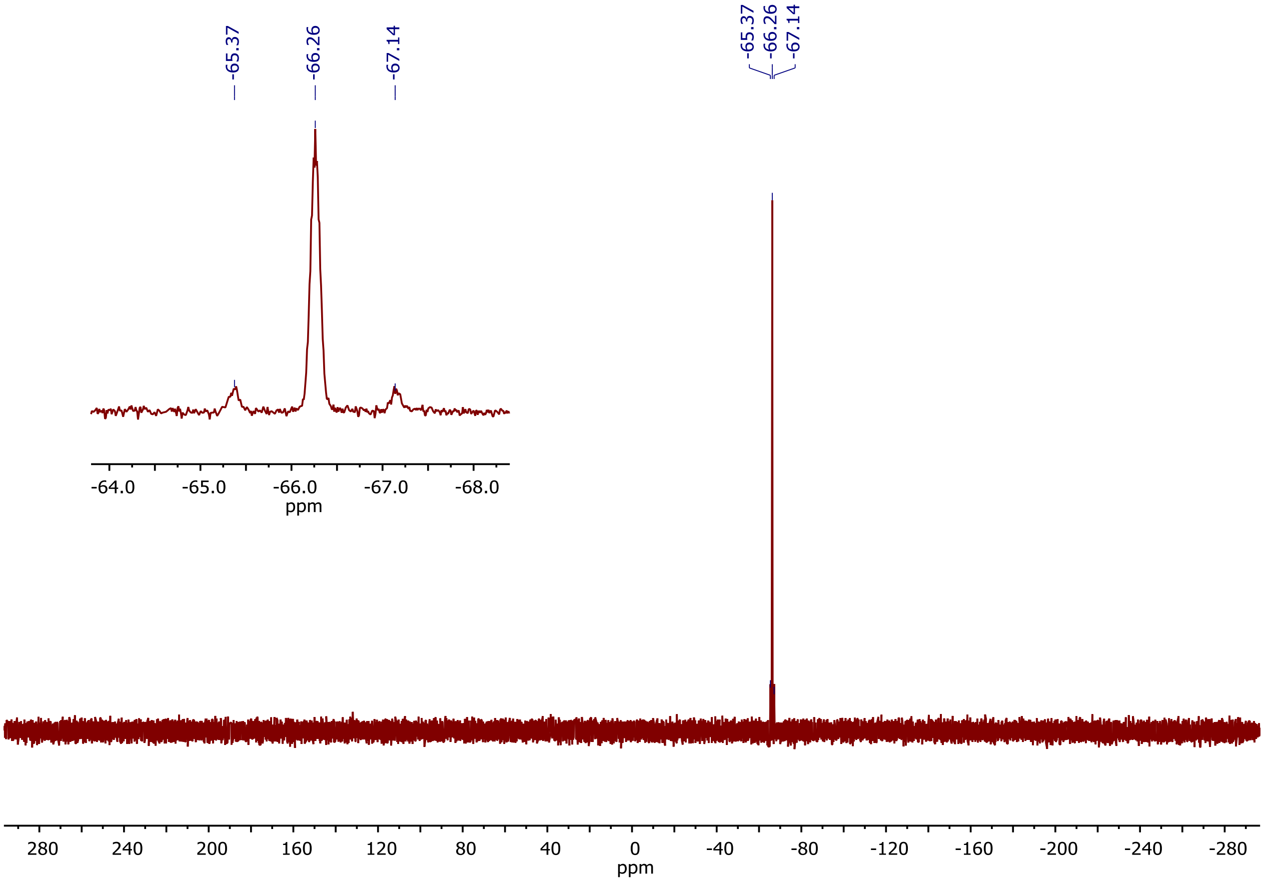

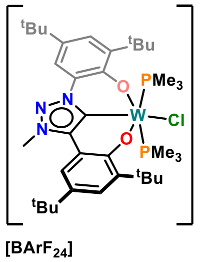

Figure S 58: ^{31}P NMR of **11-W** in C_6D_6 at 298K.

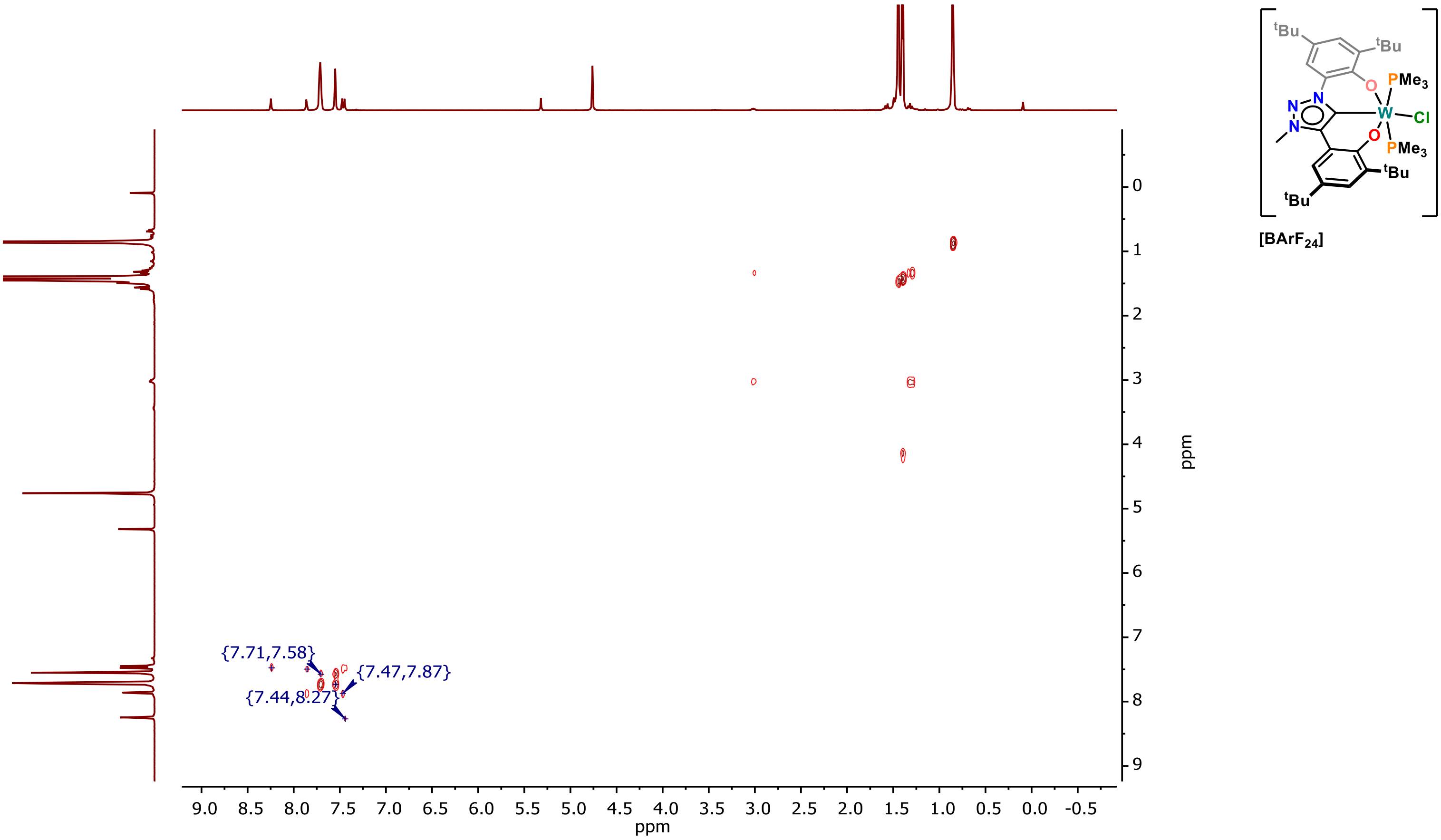

Figure S 59: ^{1}H-^{1}H COSY NMR of **11-W** in C_6D_6 at 298K.

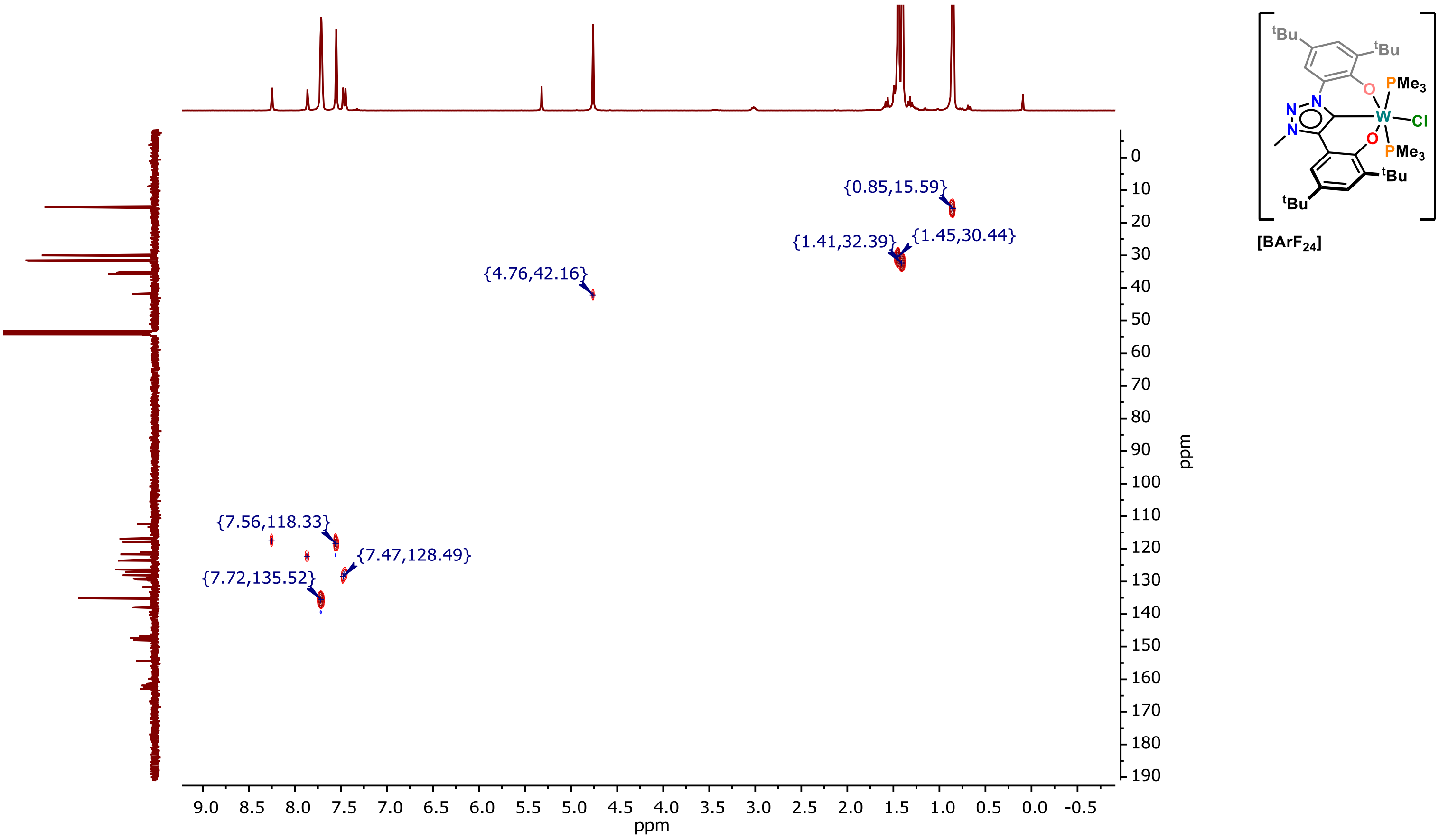

Figure S 60: ^{1}H-^{13}C HSQC NMR of **11-W** in C_6D_6 at 298K.

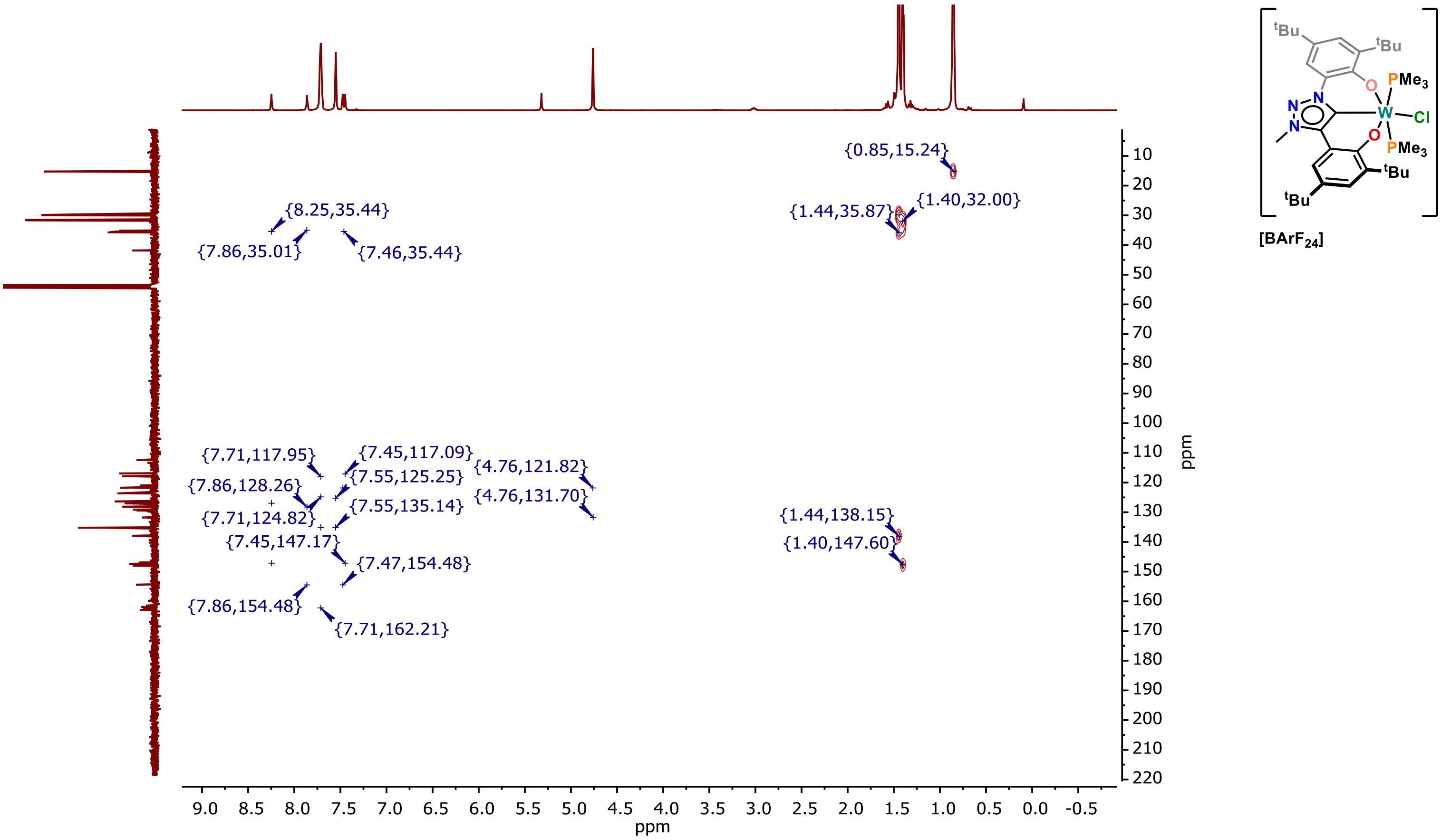

Figure S 61: ^{1}H-^{13}C HMBC NMR of **11-W** in C_6D_6 at 298K.

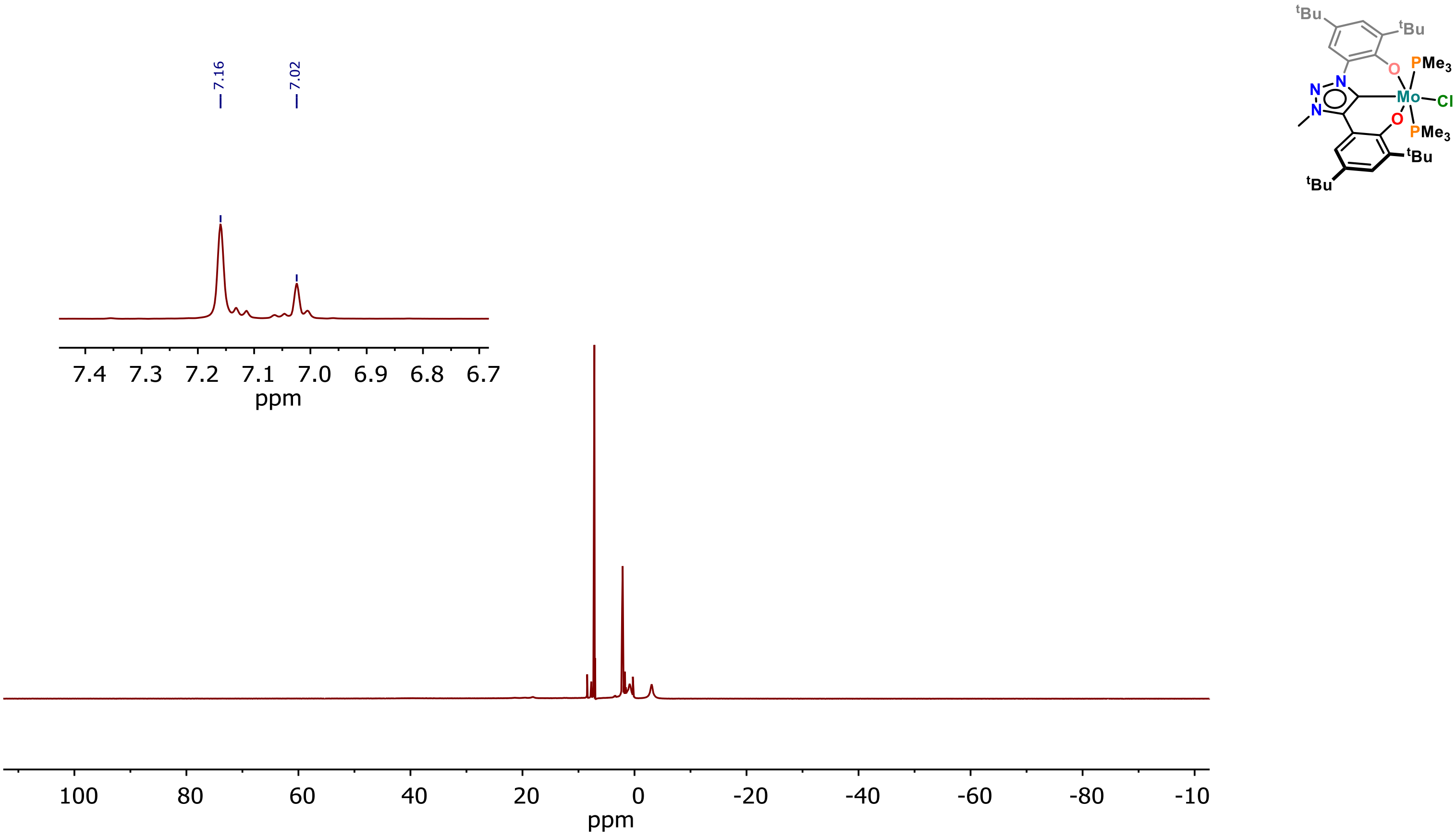

Figure S 62: Full range ^{1}H NMR of **12-Mo** with inlay, showing the peaks from Evans Method NMR measurements.

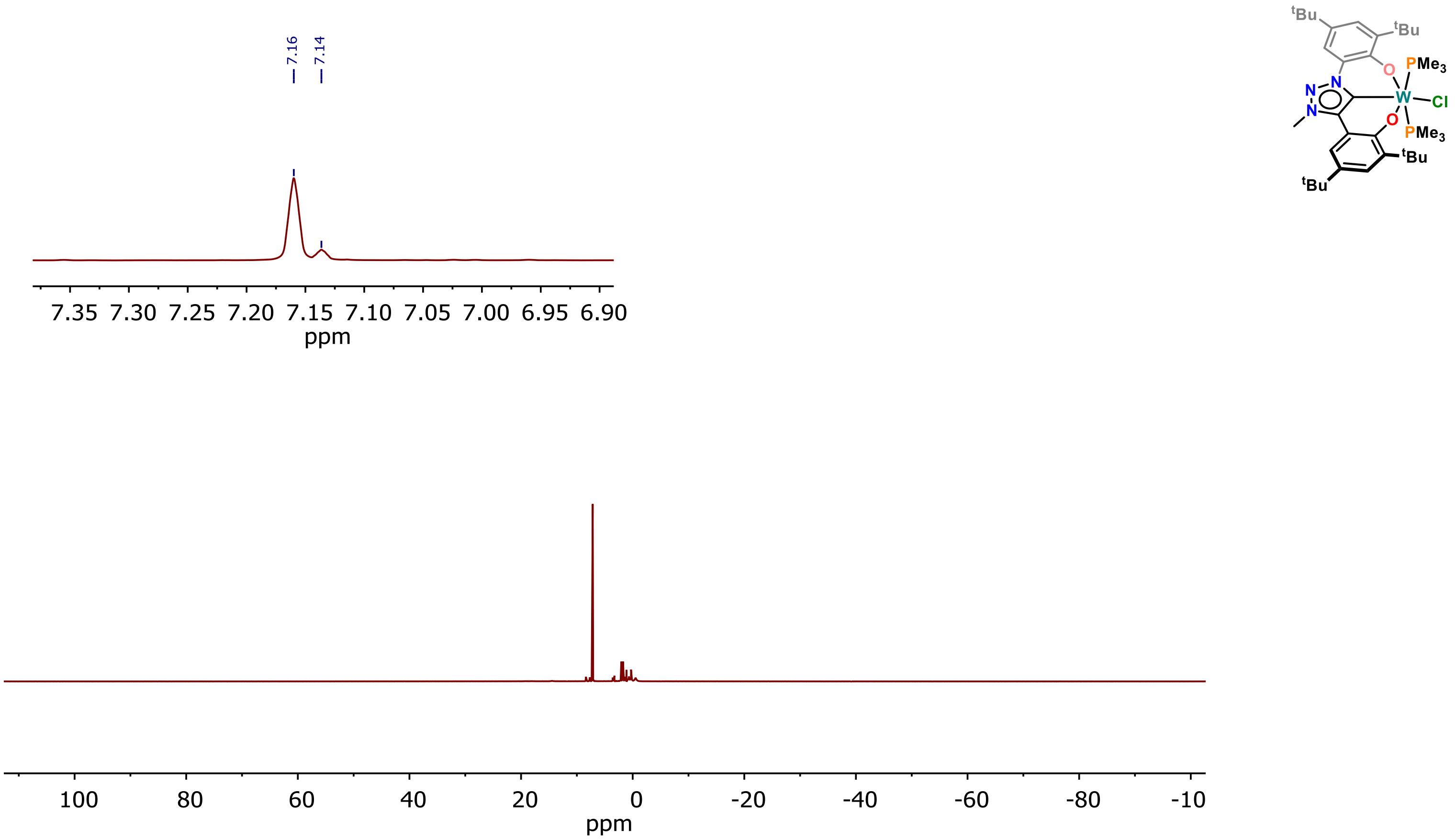

Figure S 63: Full range ^{1}H NMR of **12-Mo** with inlay, showing the peaks from Evans Method NMR measurements.

2. IR spectroscopy

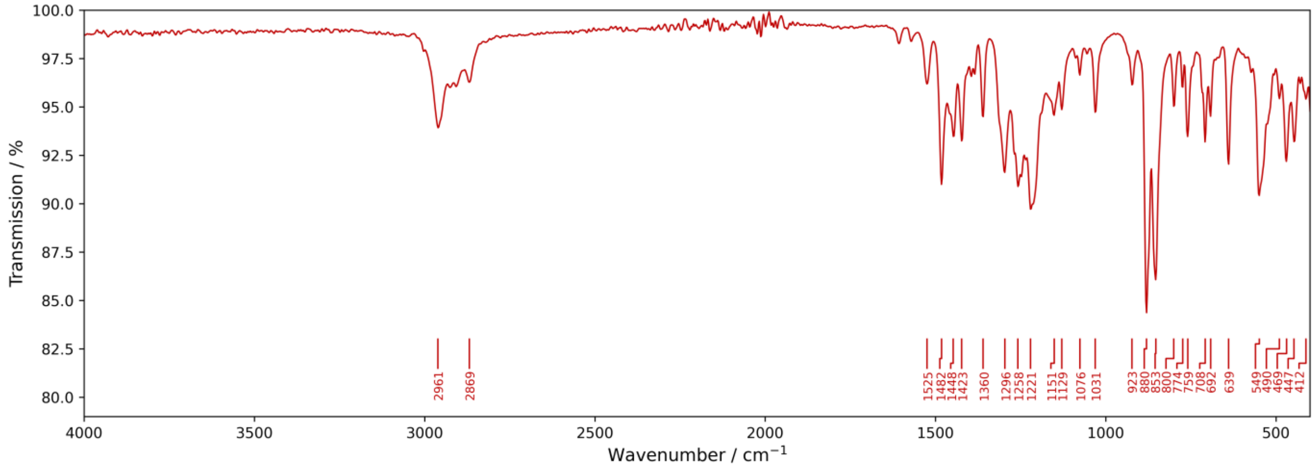

Figure S 64: IR (ATR, neat) spectrum of **2-Mo** at 298 K.

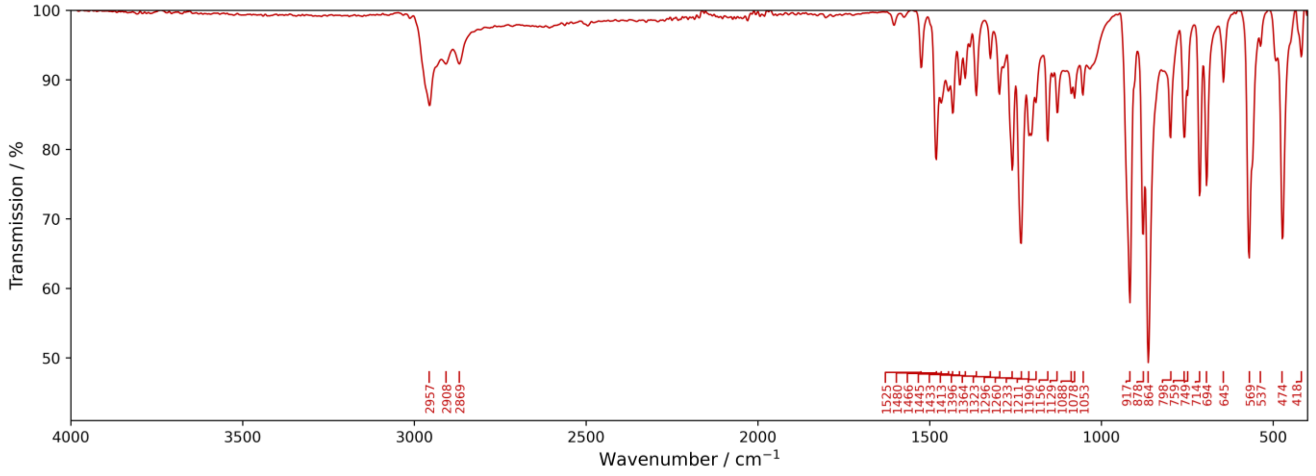

Figure S 65: IR (ATR, neat) spectrum of **3-Mo** at 298 K.

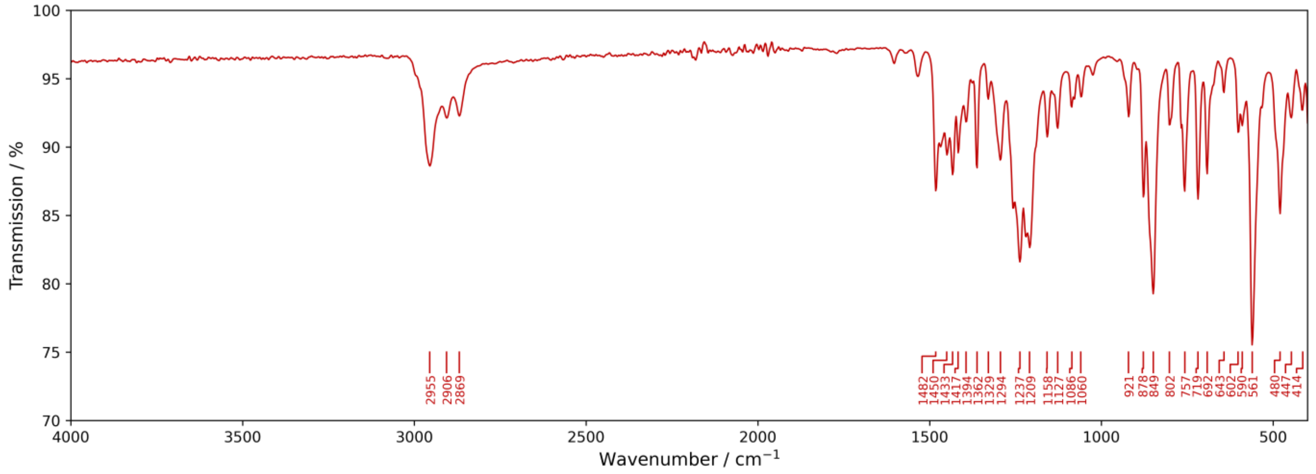

Figure S 66: IR (ATR, neat) spectrum of **4-Mo** at 298 K.

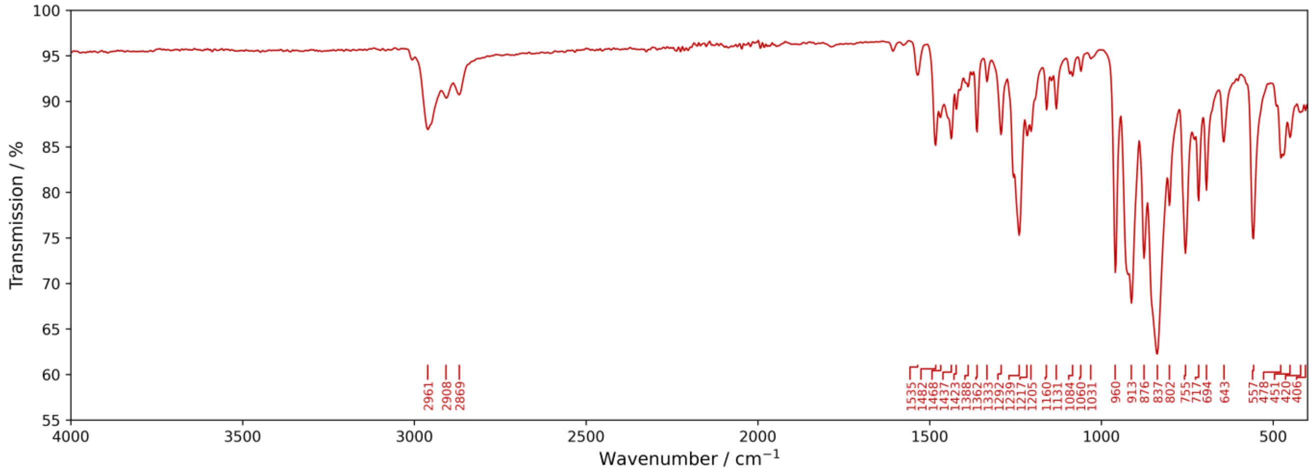

Figure S 67: IR (ATR, neat) spectrum of **3-W-OTMS** at 298 K.

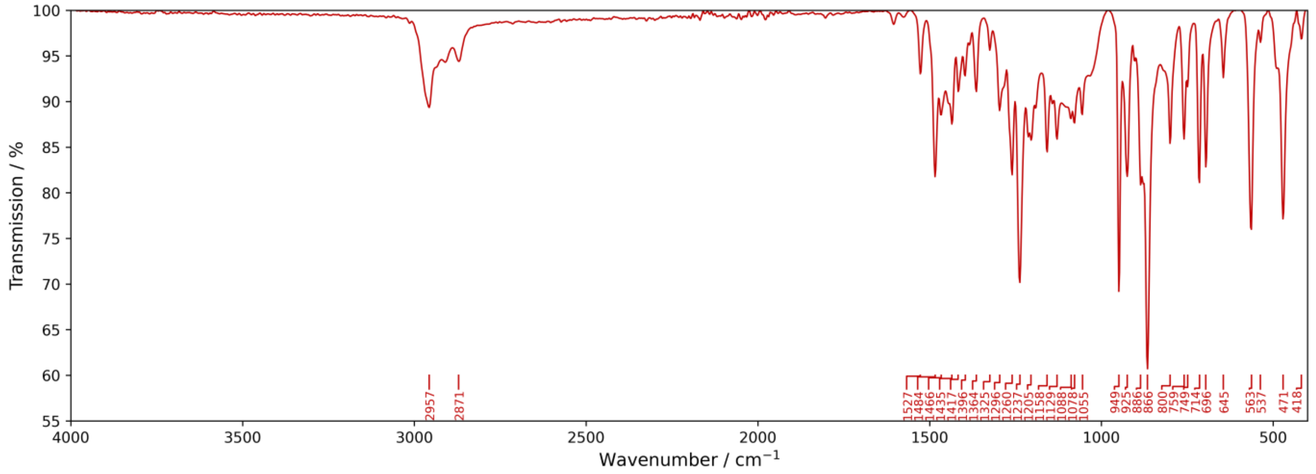

Figure S 68: IR (ATR, neat) spectrum of **3-W** at 298 K.

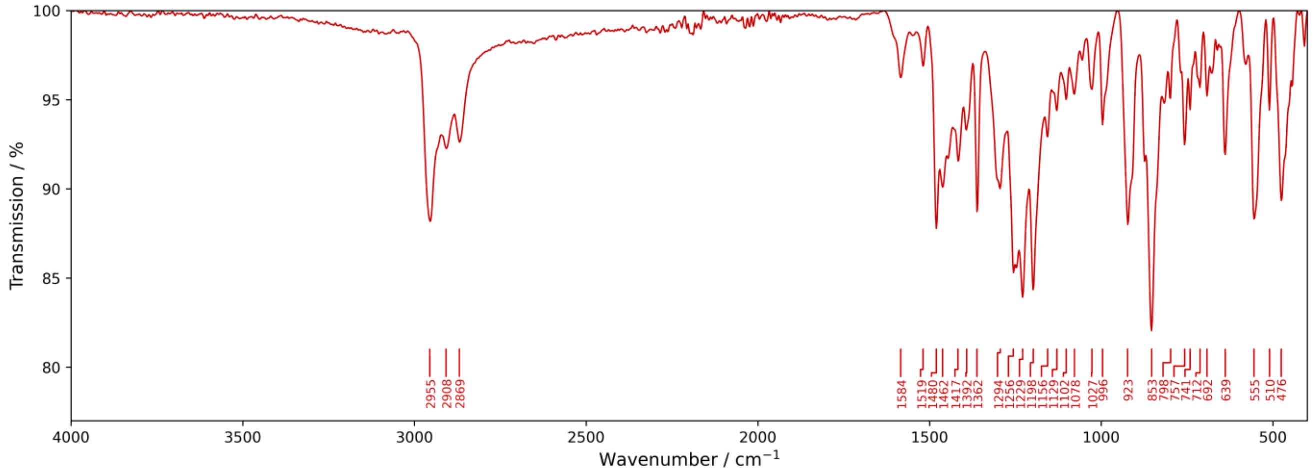

Figure S 69: IR (ATR, neat) spectrum of **5-Mo** at 298 K.

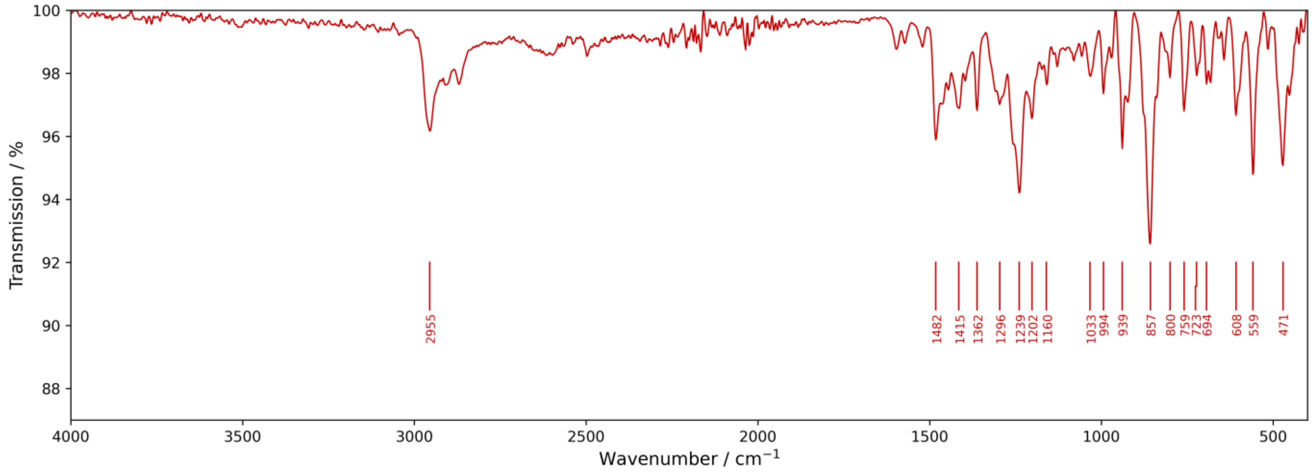

Figure S 70: IR (ATR, neat) spectrum of **5-W** at 298 K.

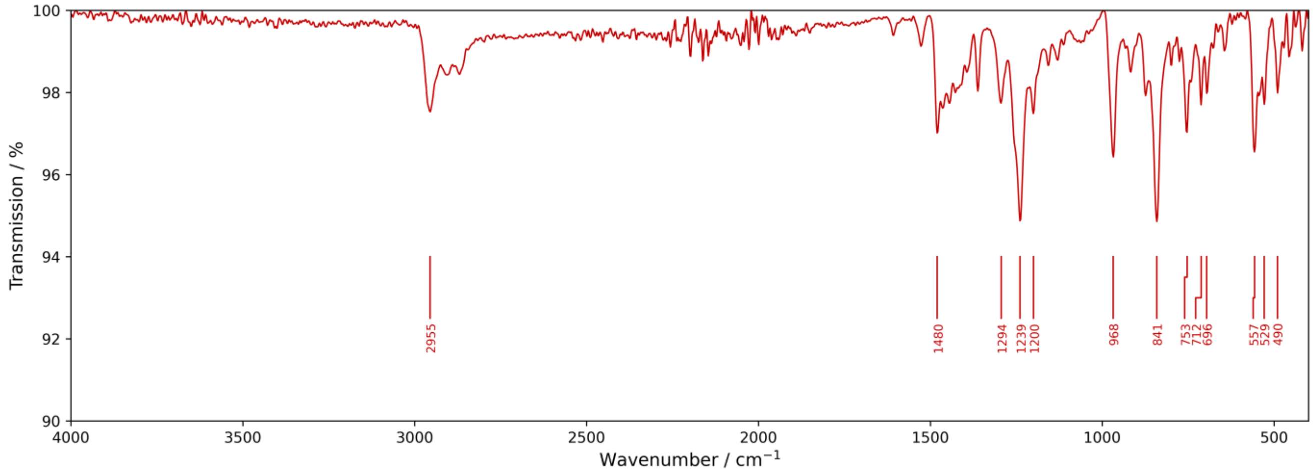

Figure S 71: IR (ATR, neat) spectrum of **6a-Mo** at 298 K.

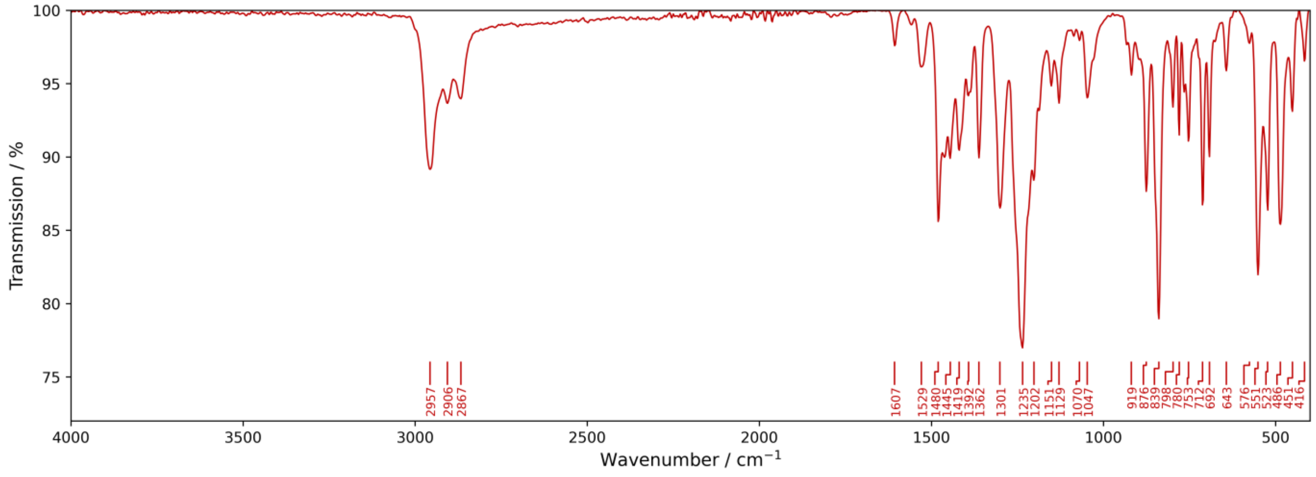

Figure S 72: IR (ATR, neat) spectrum of **6b-Mo** at 298 K.

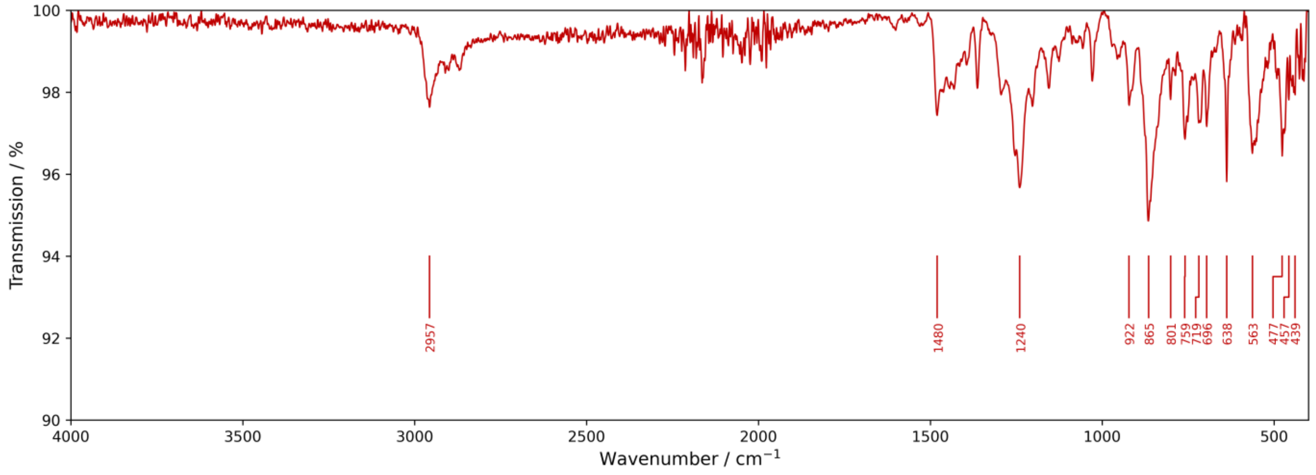

Figure S 73: IR (ATR, neat) spectrum of **6a-W** at 298 K.

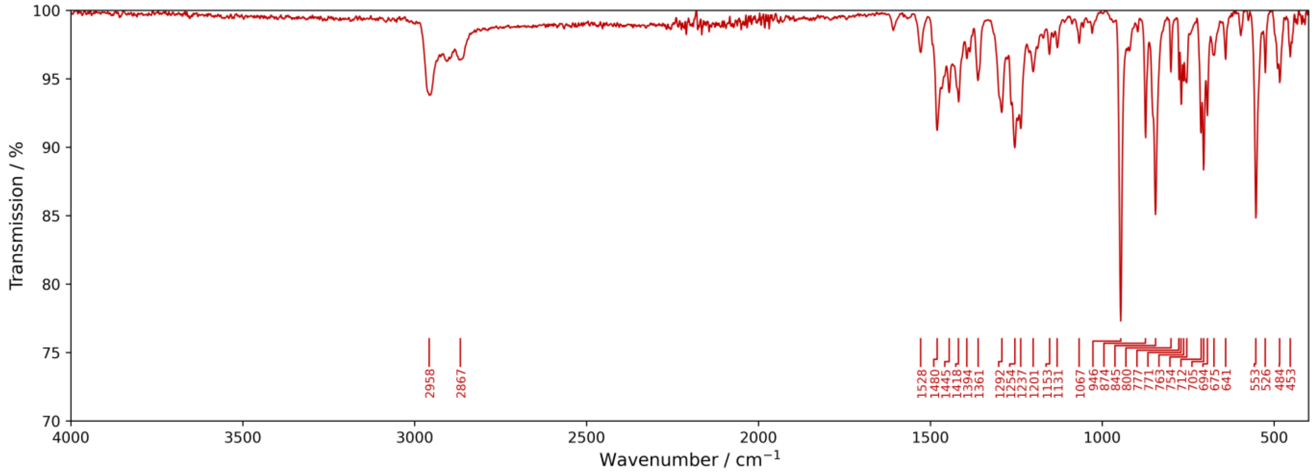

Figure S 74: IR (ATR, neat) spectrum of **7a** at 298 K.

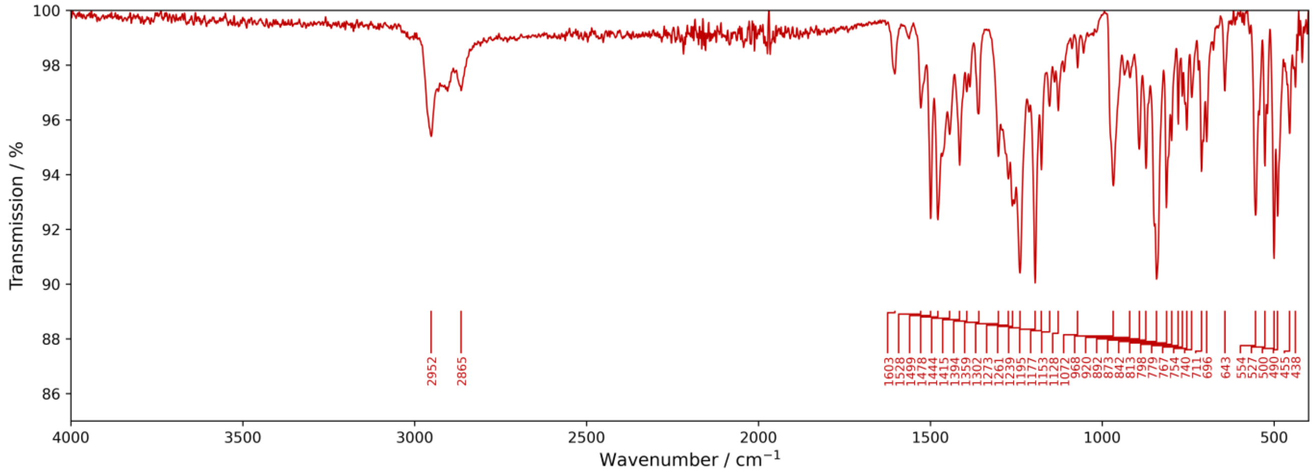

Figure S 75: IR (ATR, neat) spectrum of **7c** at 298 K.

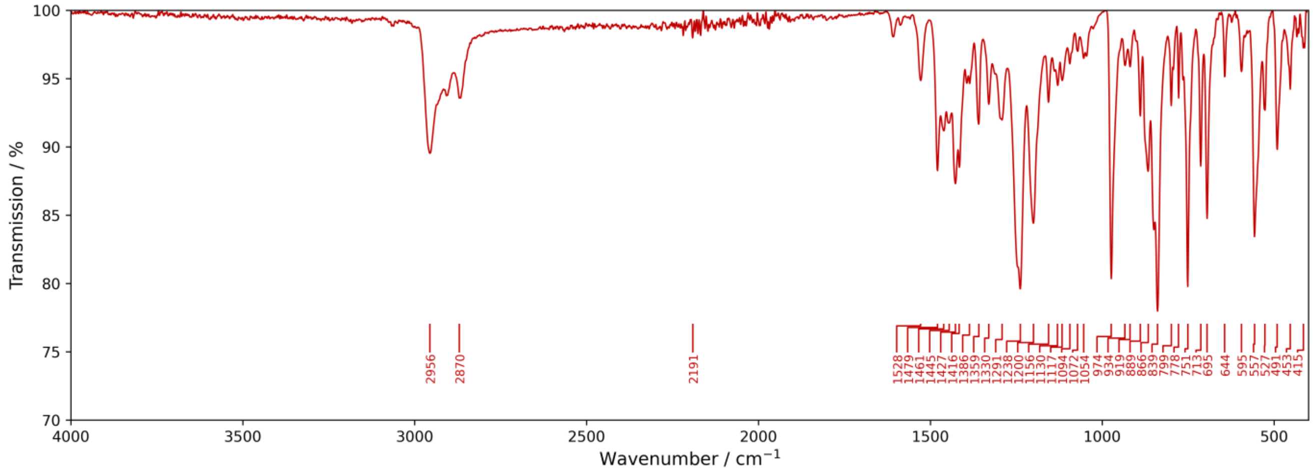

Figure S 76: IR (ATR, neat) spectrum of **7b** at 298 K.

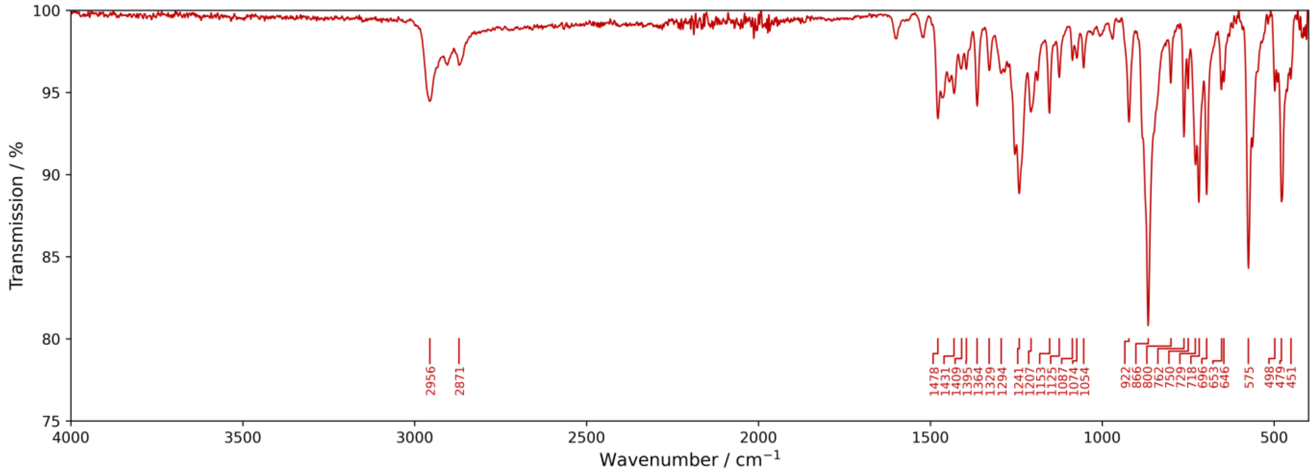

Figure S 77: IR (ATR, neat) spectrum of **8-Mo** at 298 K.

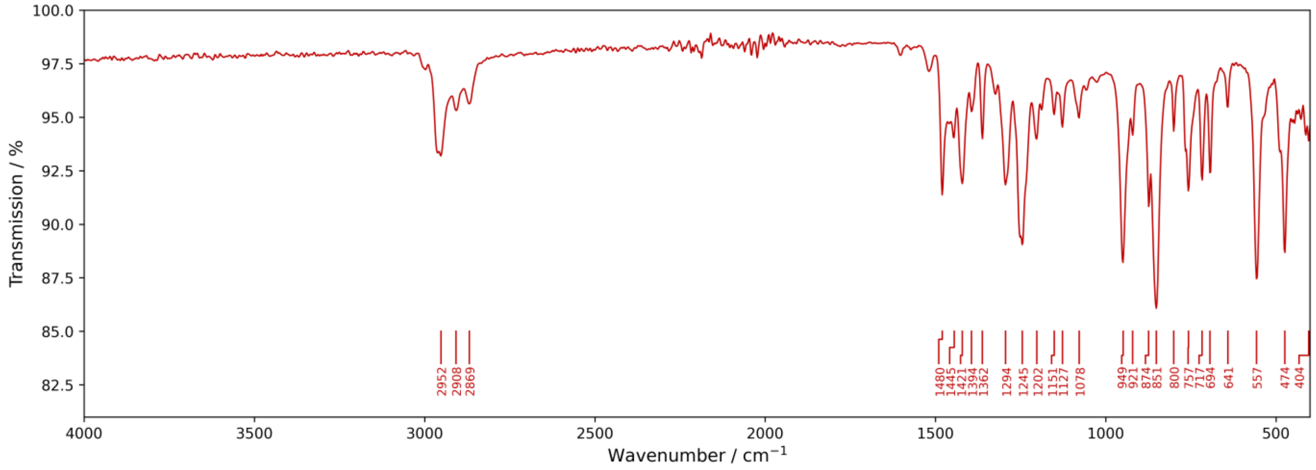

Figure S 78: IR (ATR, neat) spectrum of **9-Mo** at 298 K.

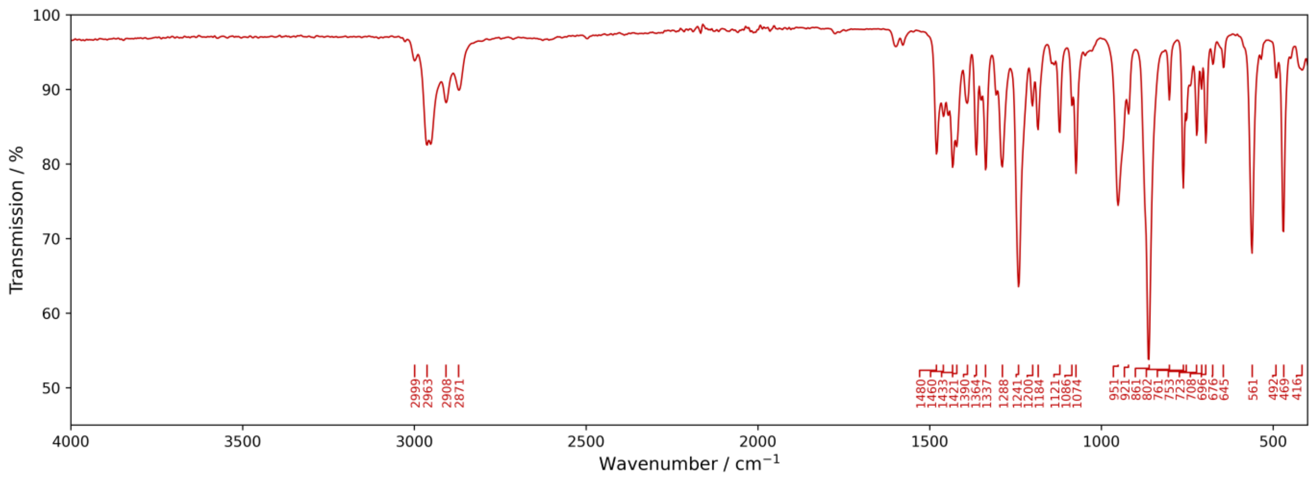

Figure S 79: IR (ATR, neat) spectrum of **9-W** at 298 K.

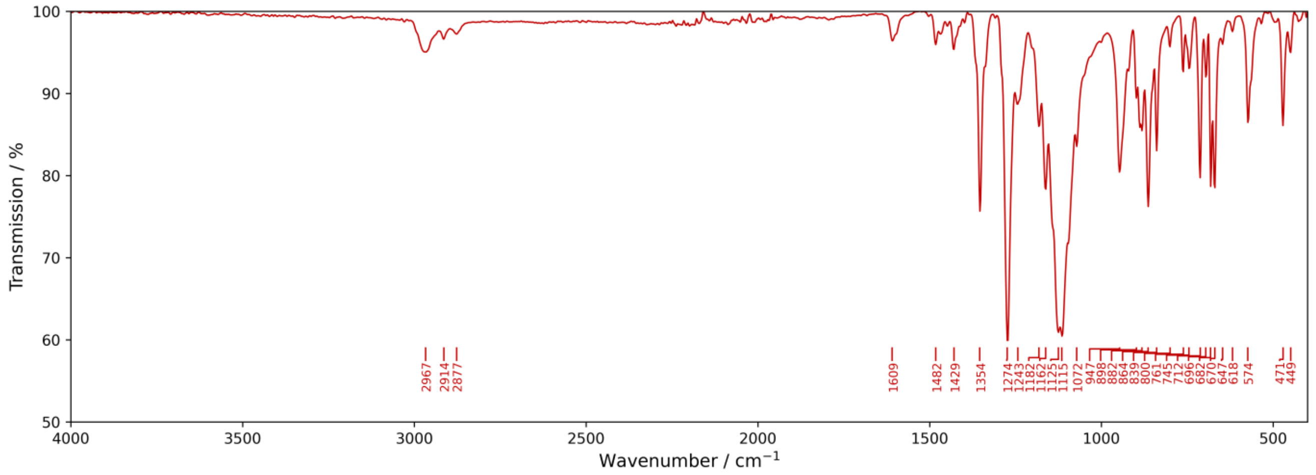

Figure S 80: IR (ATR, neat) spectrum of **11-Mo** at 298 K.

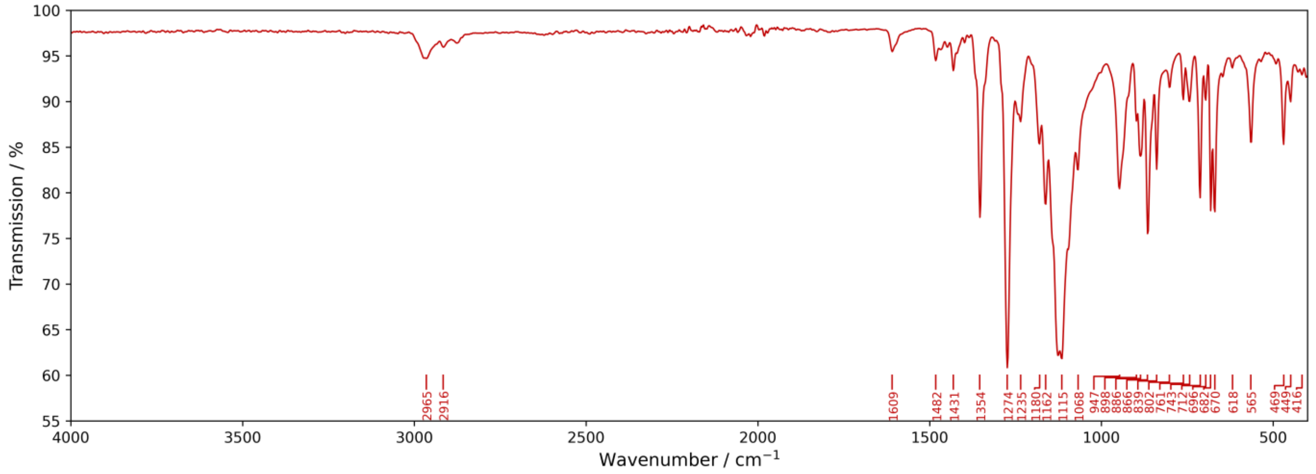

Figure S 81: IR (ATR, neat) spectrum of **11-W** at 298 K.

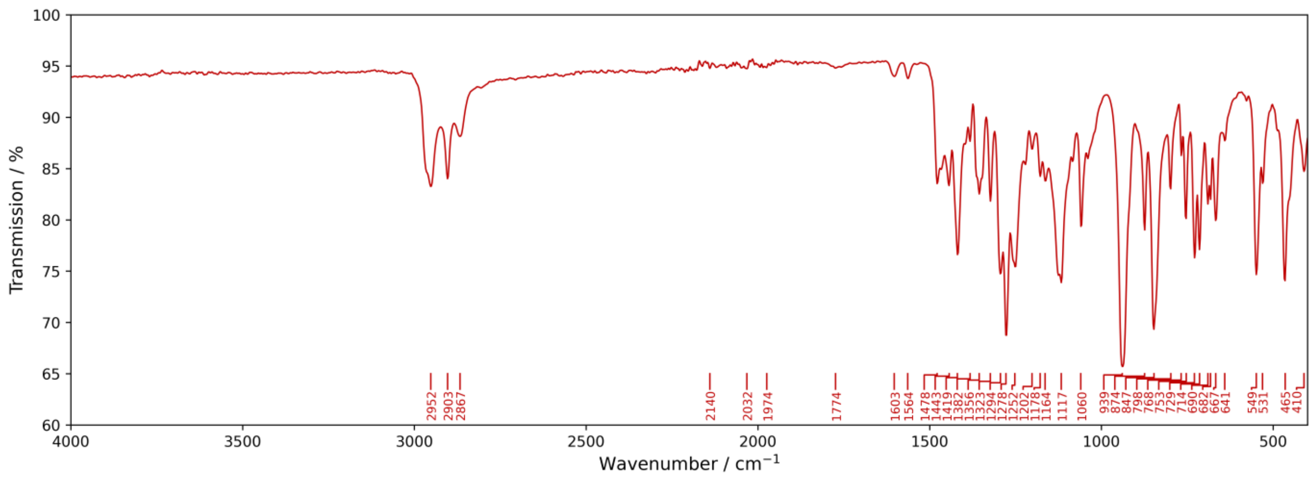

Figure S 82: IR (ATR, neat) spectrum of **12-Mo** at 298 K.

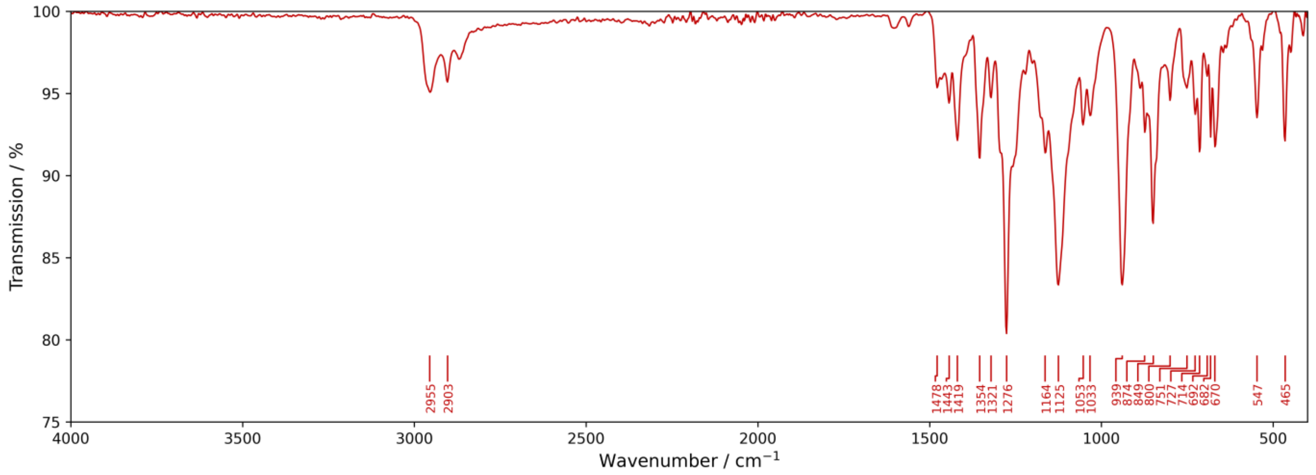

Figure S 83: IR (ATR, neat) spectrum of **12-W** at 298 K.

3. UV-Vis-NIR spectra

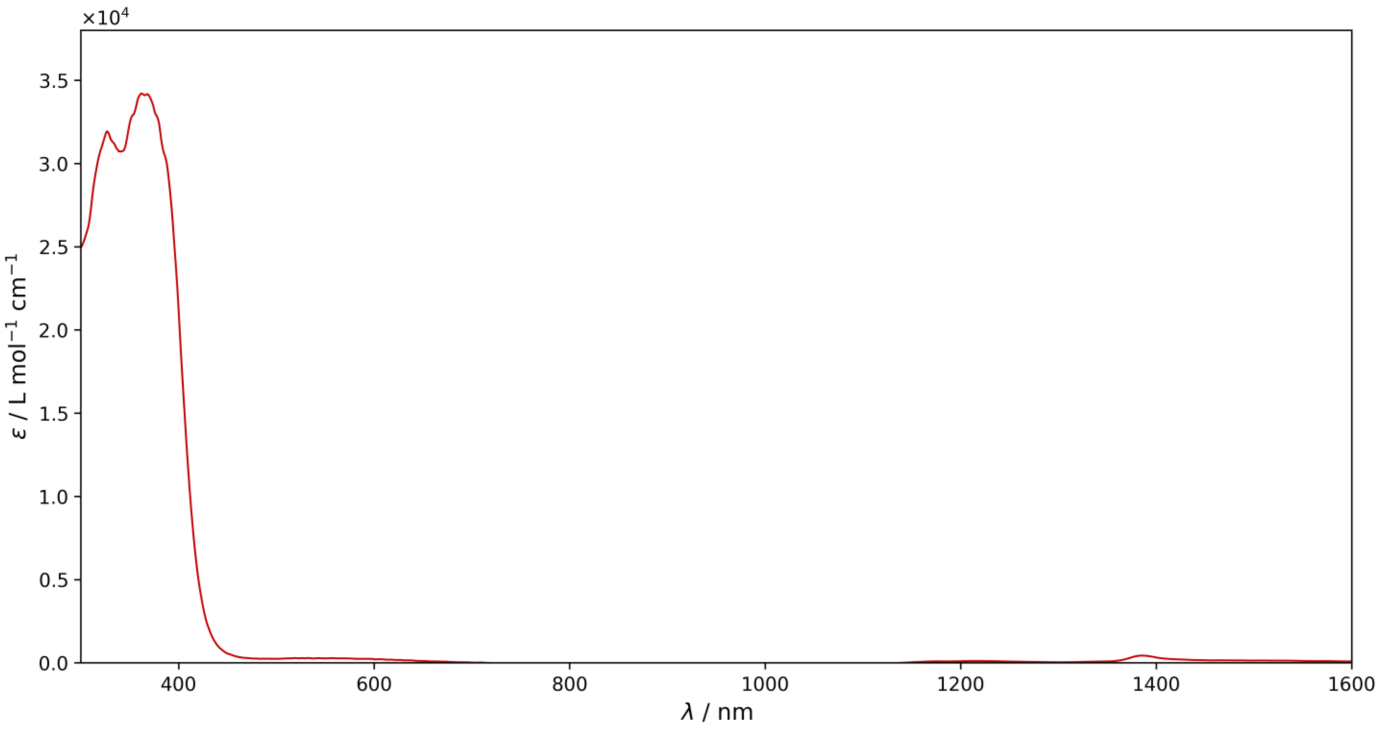

Figure S 84: UV-Vis-NIR spectrum of **2-Mo** in THF at 298 K.

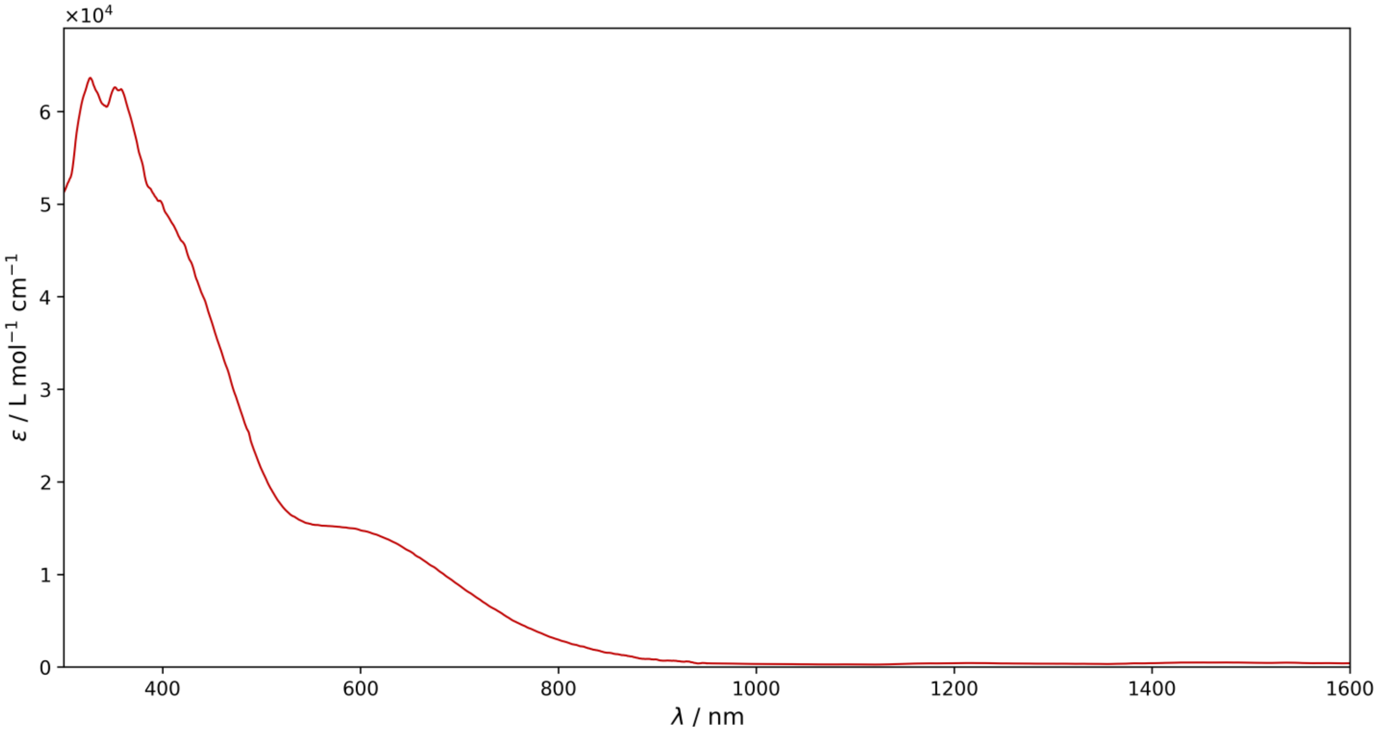

Figure S 85: UV-Vis-NIR spectrum of **3-Mo** in THF 298 K.

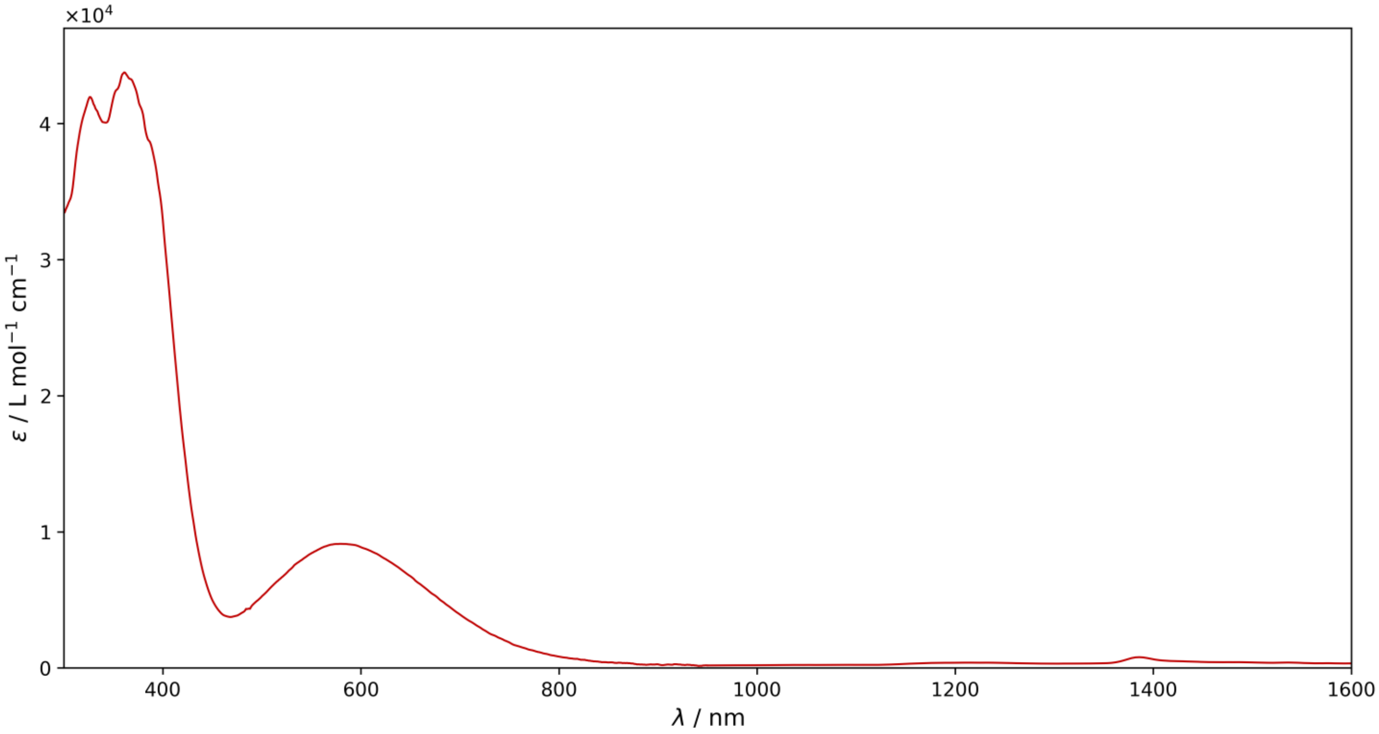

Figure S 86: UV-Vis-NIR spectrum of **4-Mo** in THF at 298 K.

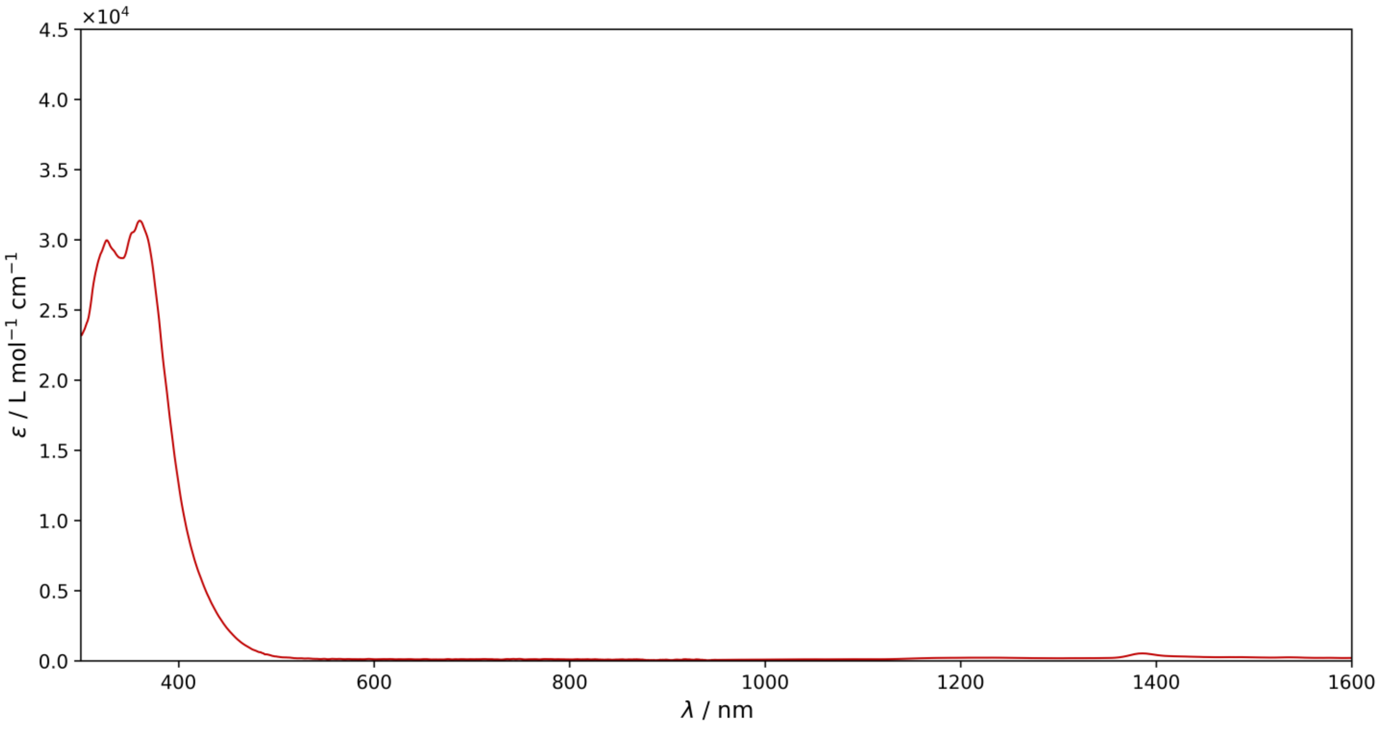

Figure S 87: UV-Vis-NIR spectrum of **3-W-(OTMS)** in THF at 298 K.

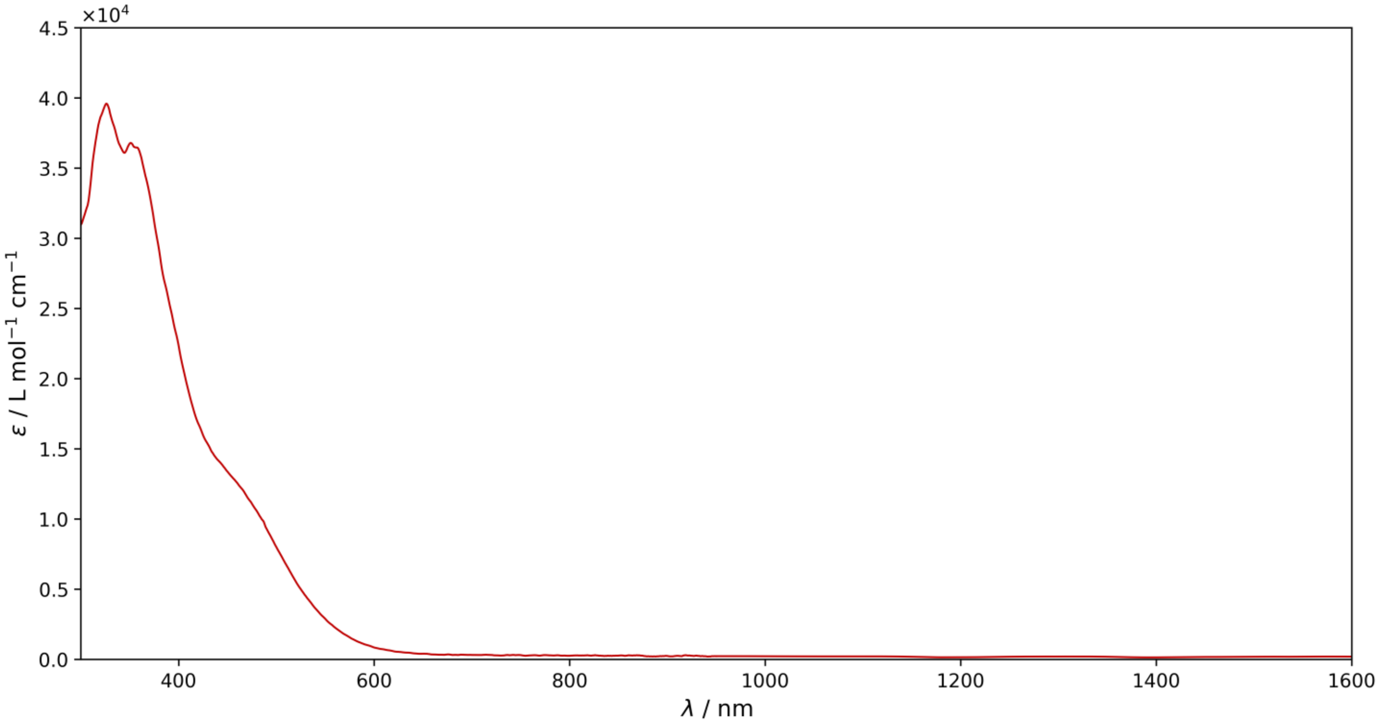

Figure S 88: UV-Vis-NIR spectrum of **3-W** in THF at 298 K.

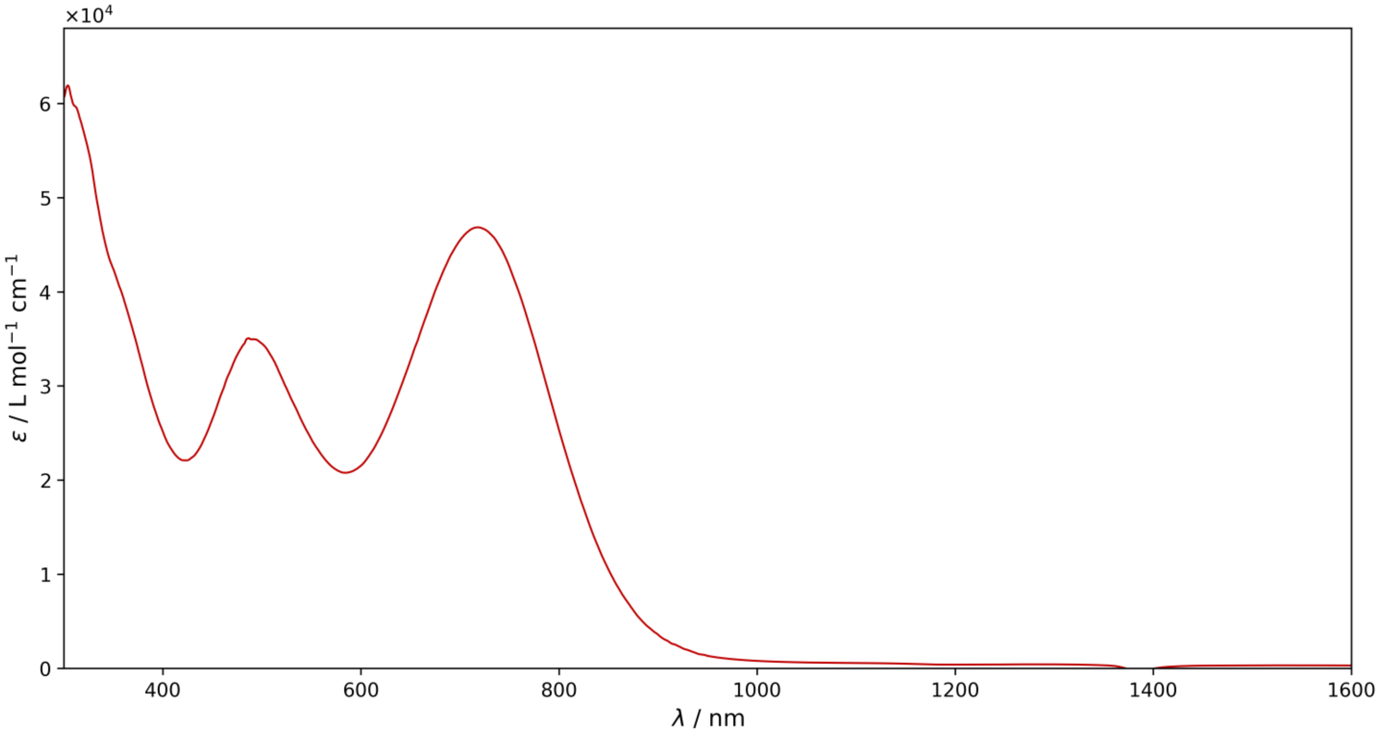

Figure S 89: UV-Vis-NIR spectrum of **5-Mo** in THF at 298 K.

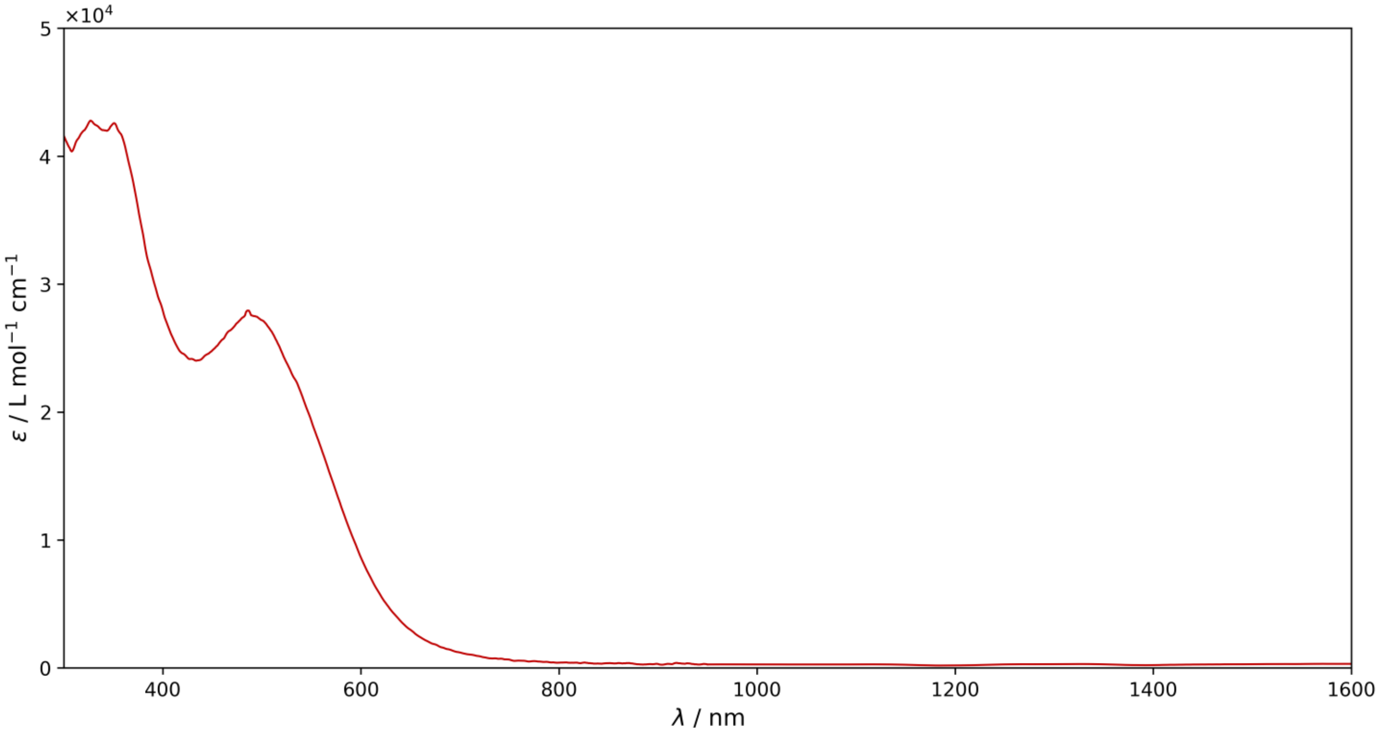

Figure S 90: UV-Vis-NIR spectrum of **5-W** in THF at 298 K.

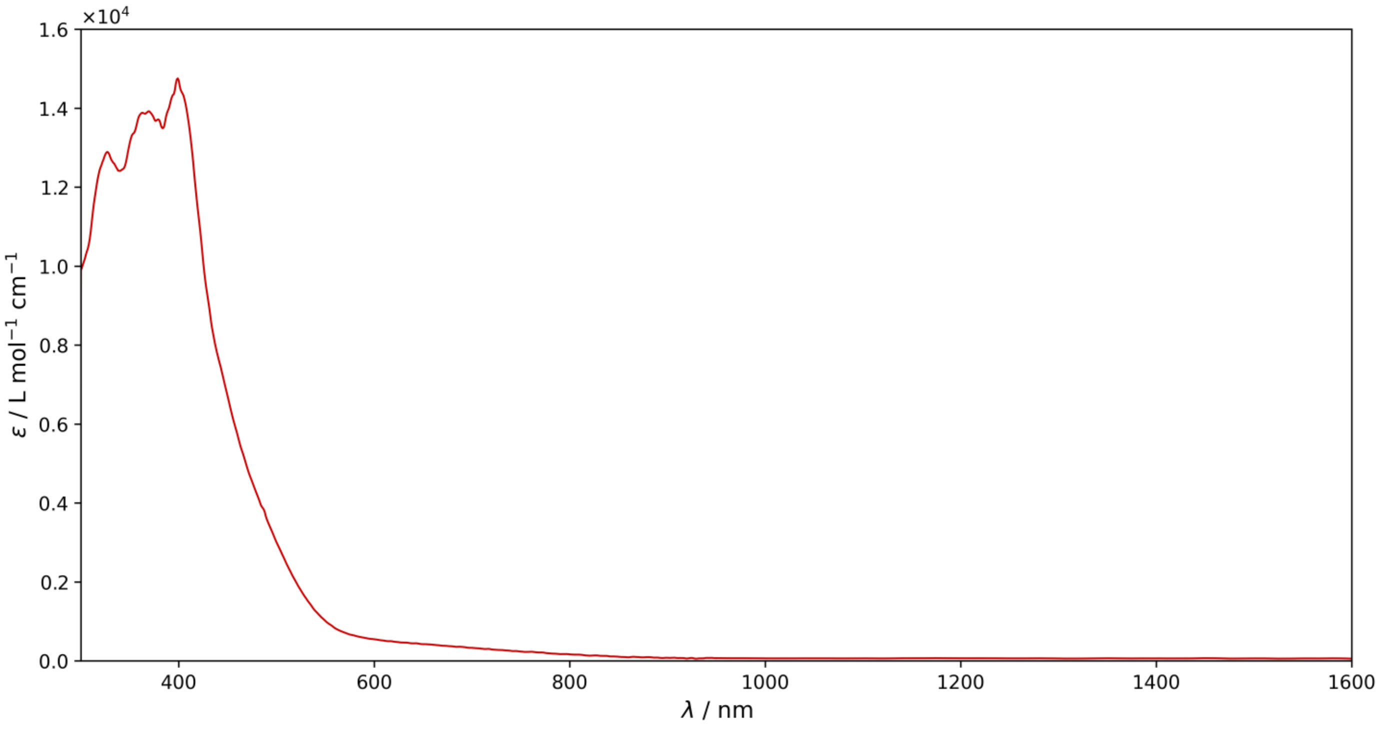

Figure S 91: UV-Vis-NIR spectrum of **6a-Mo** in THF at 298 K.

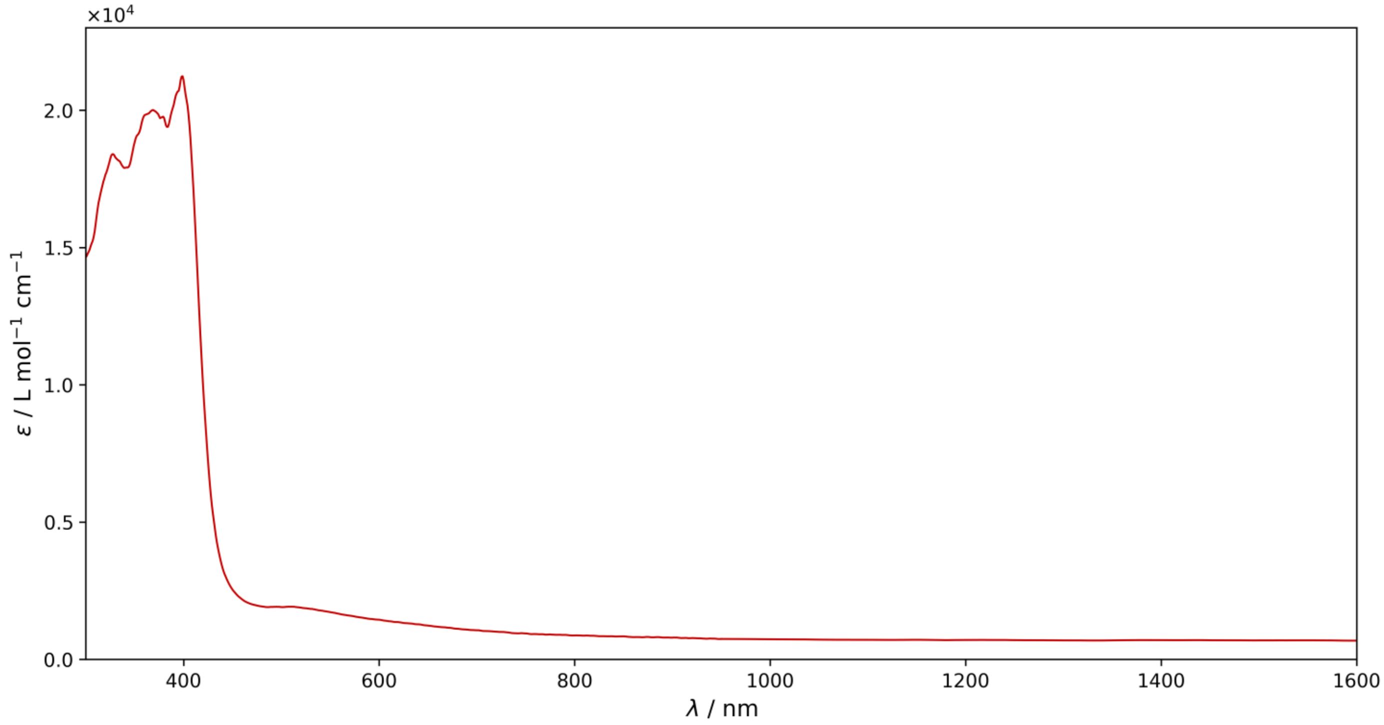

Figure S 92: UV-Vis-NIR spectrum of **6b-Mo** in THF at 298 K.

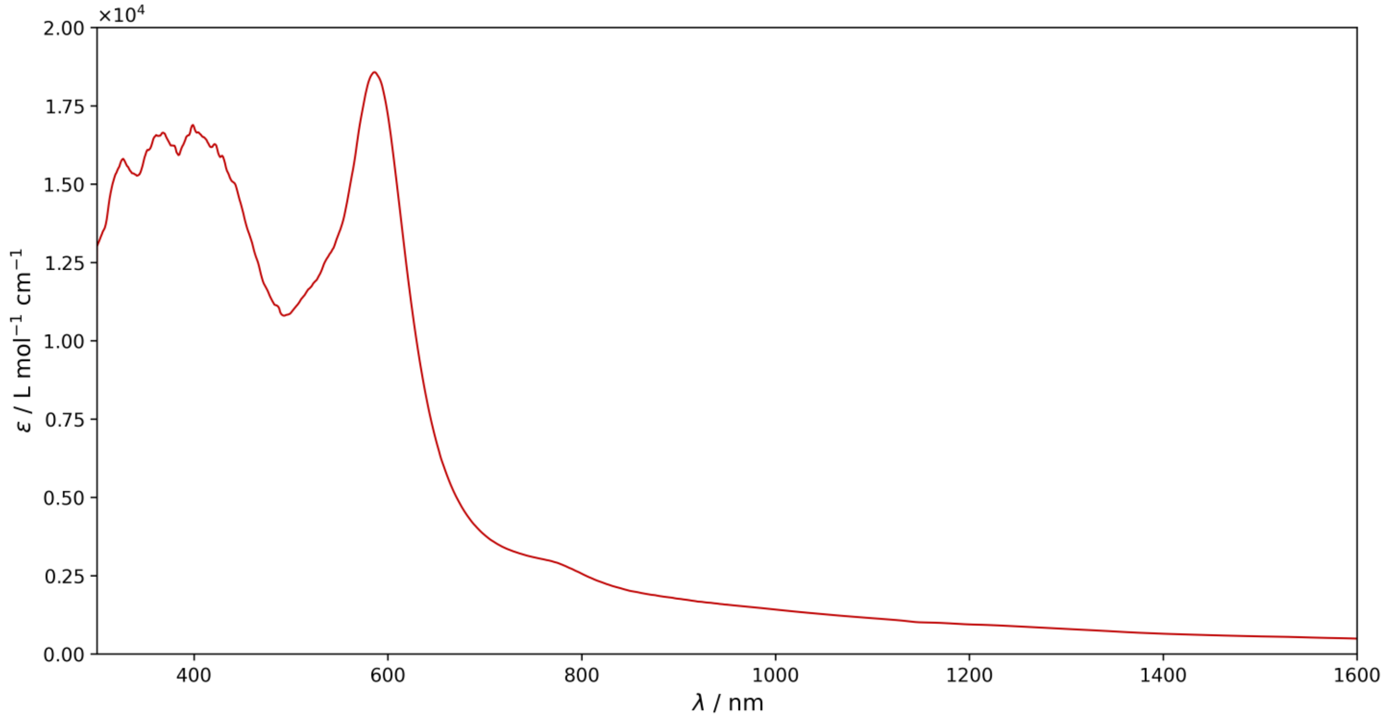

Figure S 93: UV-Vis-NIR spectrum of **6a-W** in THF at 298 K.

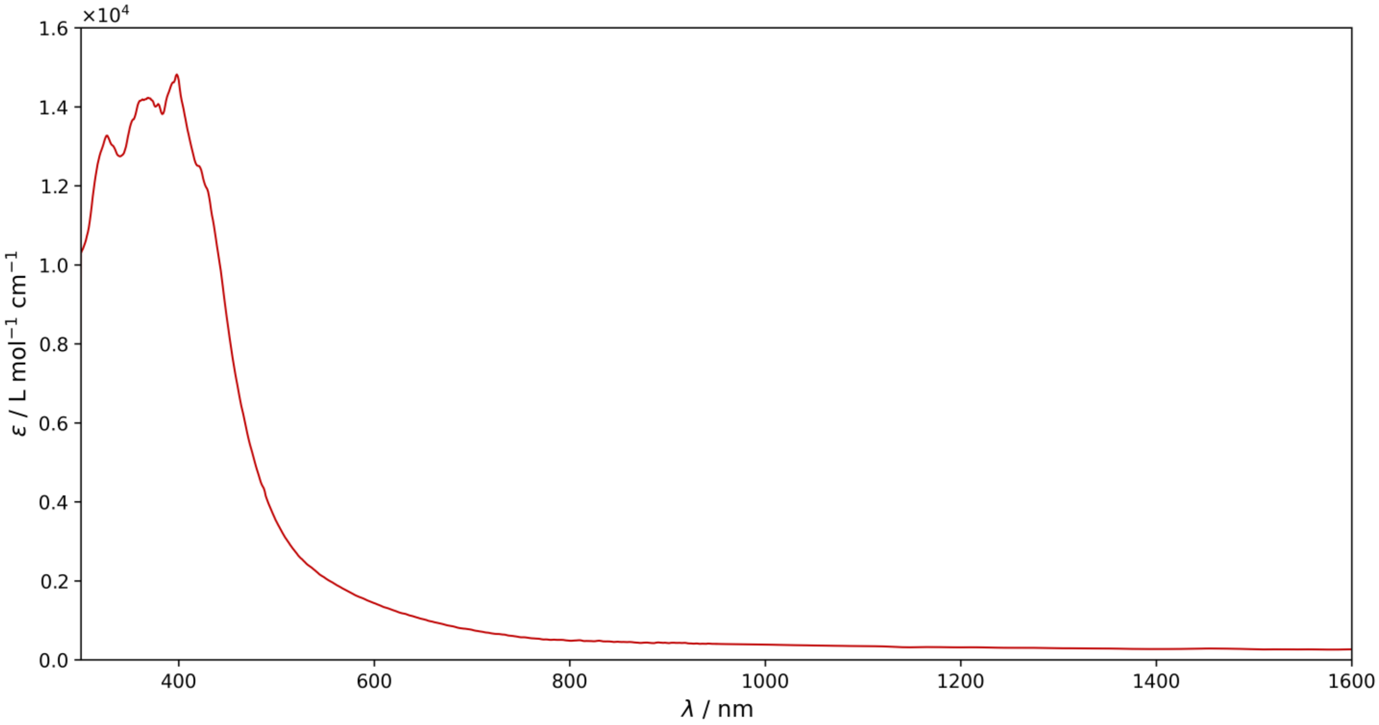

Figure S 94: UV-Vis-NIR spectrum of **7a** in THF at 298 K.

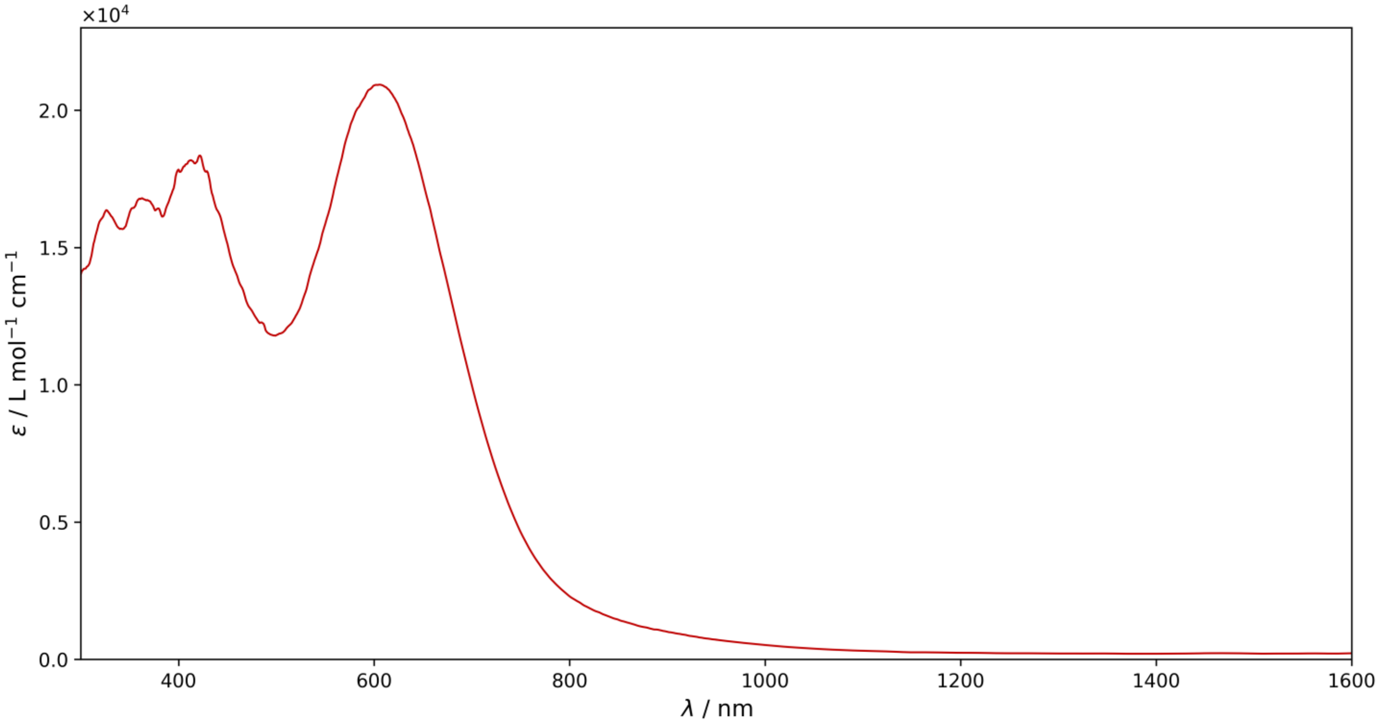

Figure S 95: UV-Vis-NIR spectrum of **7c** in THF at 298 K.

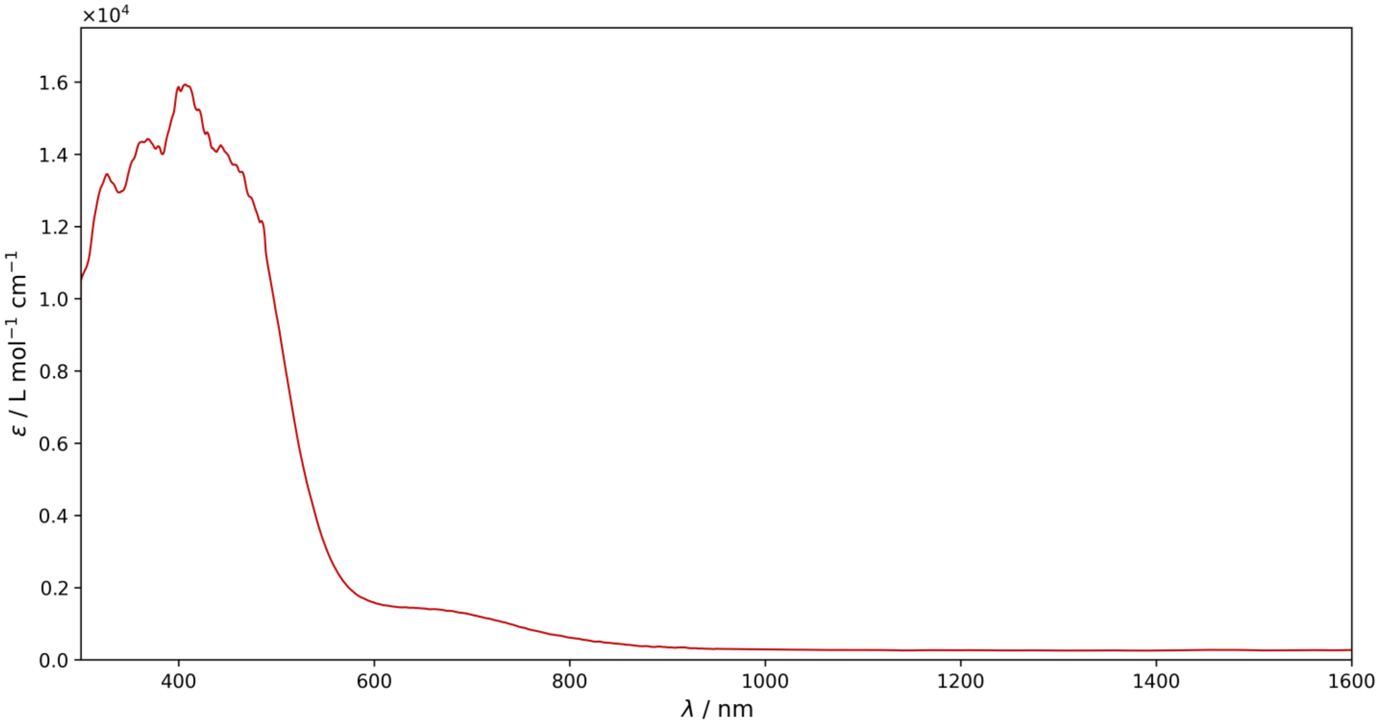

Figure S 96: UV-Vis-NIR spectrum of **7b** in THF at 298 K.

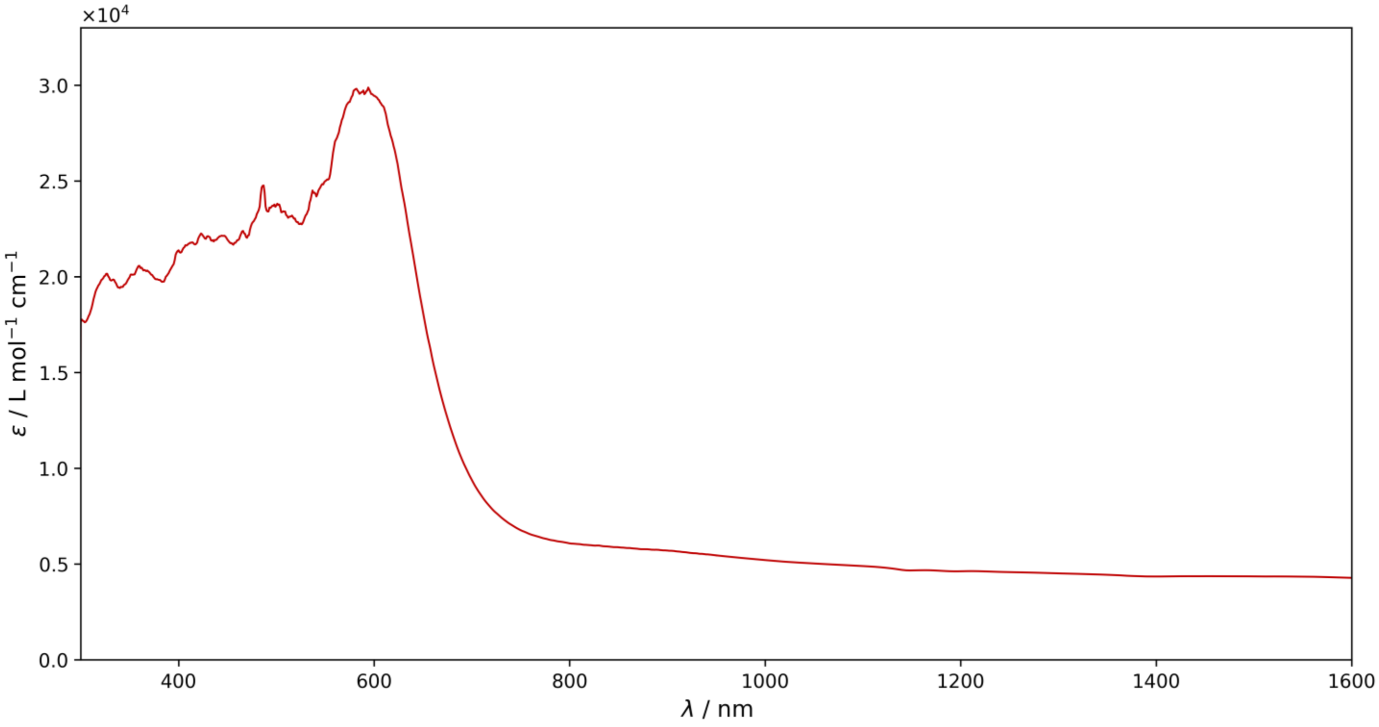

Figure S 97: UV-Vis-NIR spectrum of **8-Mo** in THF at 298 K.

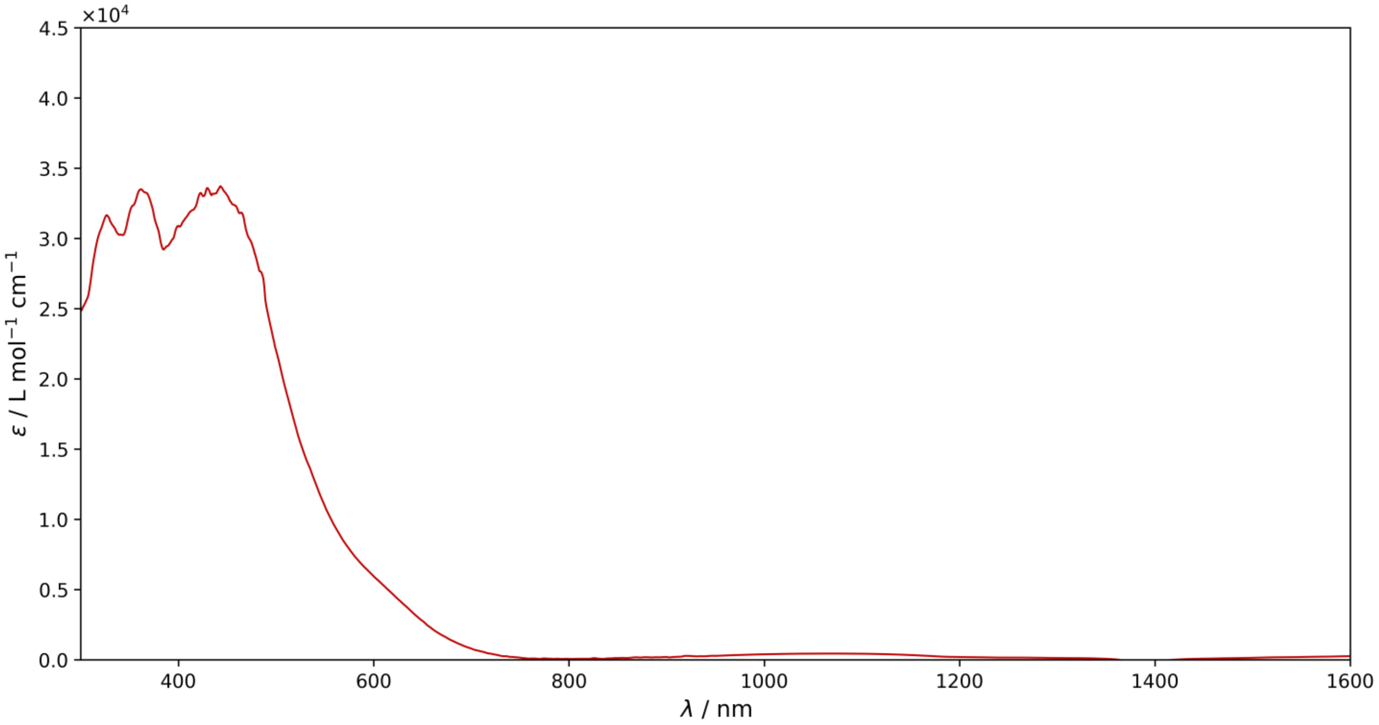

Figure S 98: UV-Vis-NIR spectrum of **9-Mo** in THF at 298 K.

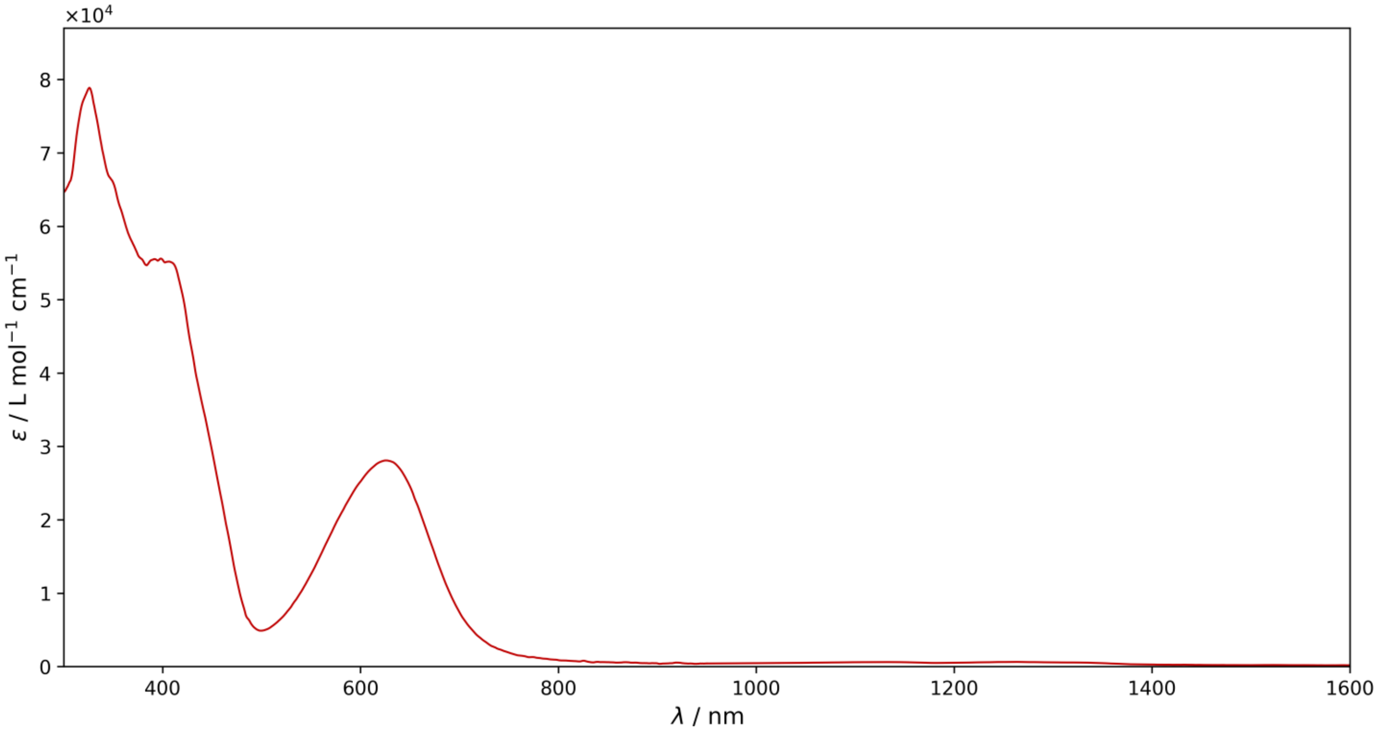

Figure S 99: UV-Vis-NIR spectrum of **9-W** in THF at 298 K.

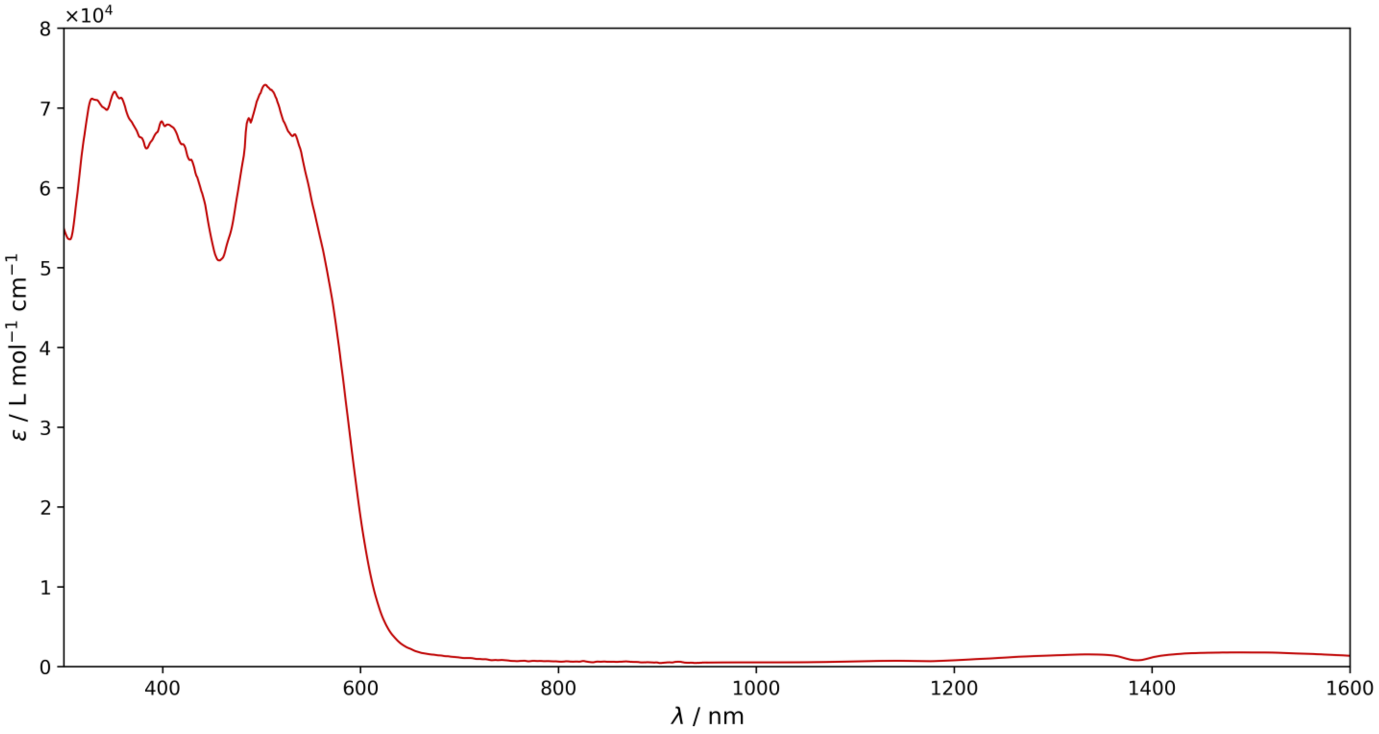

Figure S 100: UV-Vis-NIR spectrum of **11-Mo** in THF at 298 K.

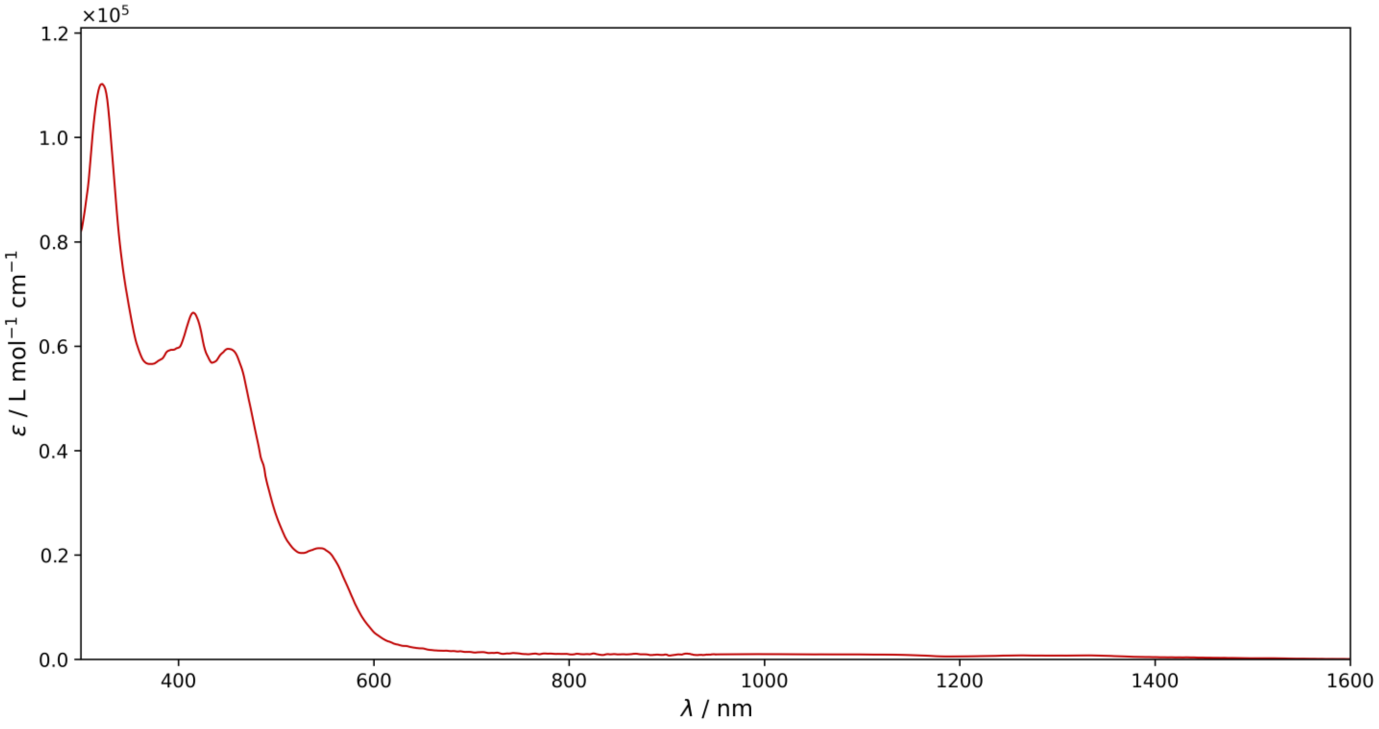

Figure S 101: UV-Vis-NIR spectrum of **11-W** in THF at 298 K.

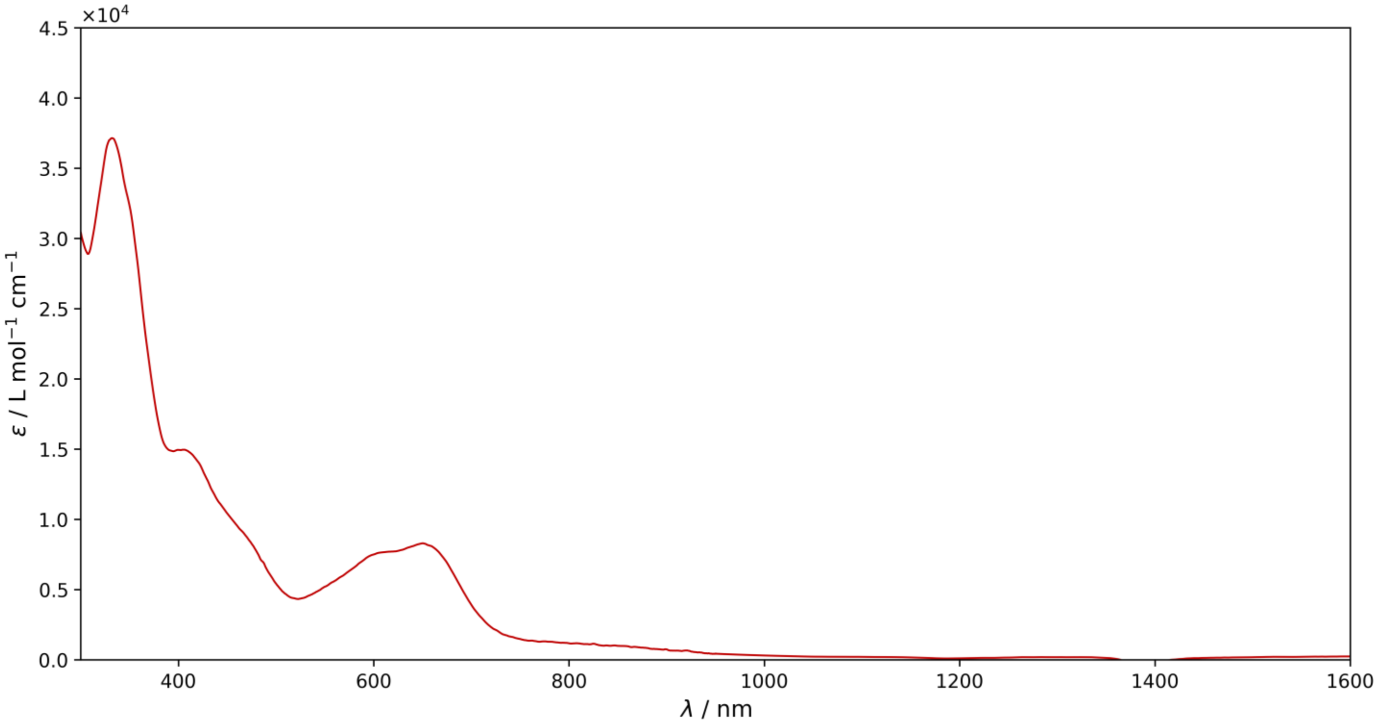

Figure S 102: UV-Vis-NIR spectrum of **12-Mo** in THF at 298 K.

4. EPR spectra

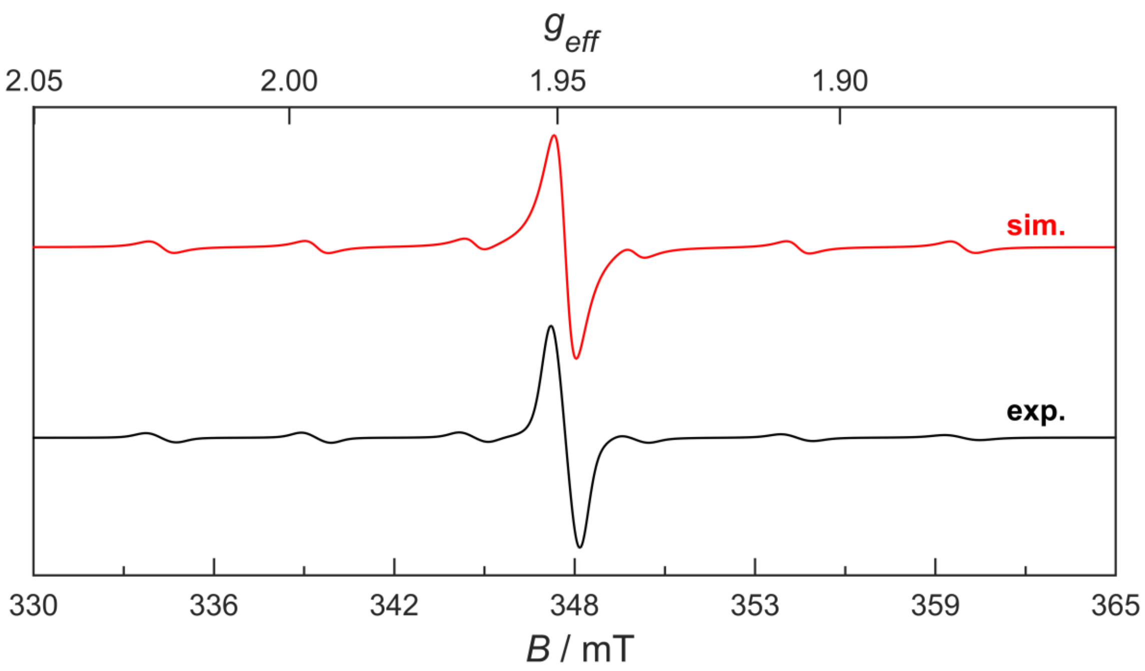

Figure S 103: Room temperature EPR spectrum of **7b** in dichloromethane. g_{iso} = 1.949; a_{iso}(Mo) = 140 MHz.

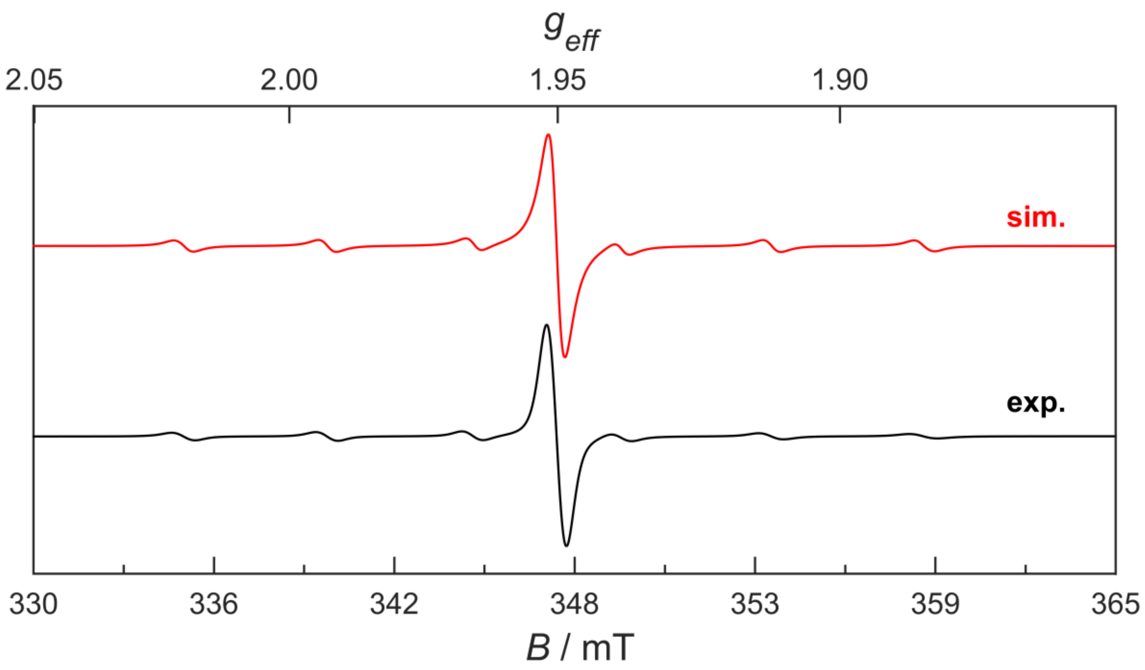

Figure S 104: Room temperature EPR spectrum of **7c** in dichloromethane. g_{iso} = 1.950; a_{iso}(Mo) = 130 MHz.

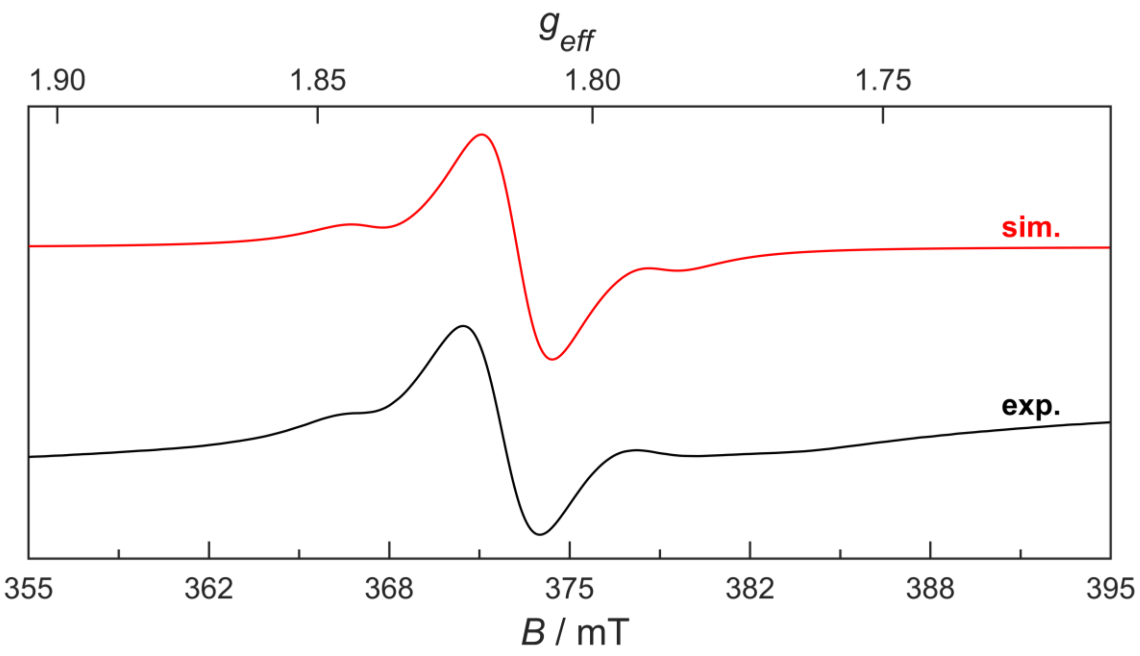

Figure S 105: X-band EPR spectrum of **6a-W** in DCM at 293 K.

5. Cyclic voltammetry

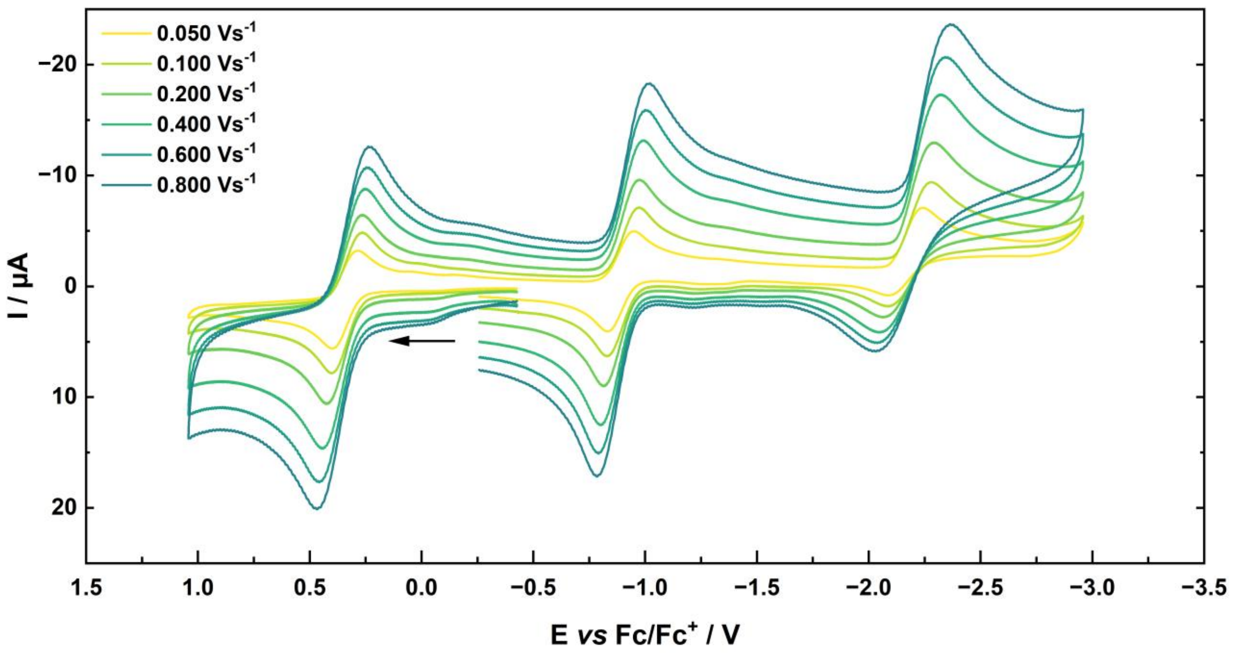

Figure S 106: Cyclic voltammogram of **11-Mo**, recorded in 0.15 M NBu_4PF_6 in THF and referenced against the ferrocene/ferrocenium redox couple.

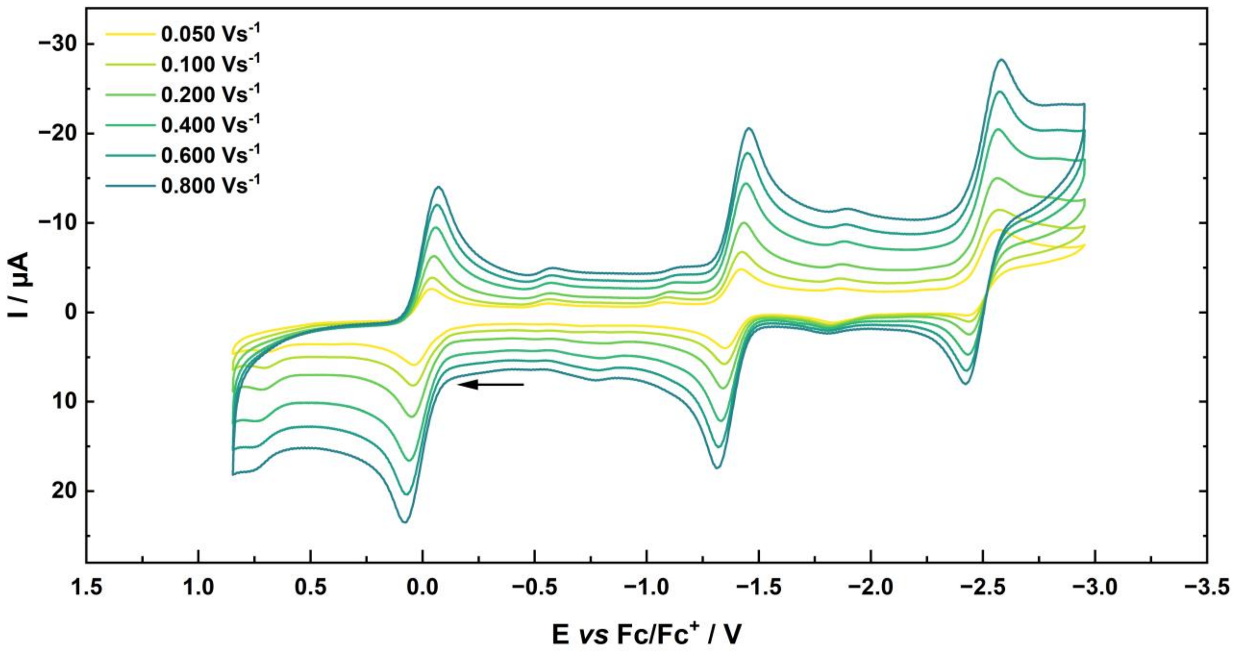

Figure S 107: Cyclic voltammogram of **11-W**, recorded in 0.15 M NBu_4PF_6 in THF and referenced against the ferrocene/ferrocenium redox couple. The small waves between the main processes result from degradation of the samples during the measurements and belong to follow-up species forming from the irreversible processes.

Table S 1: Redox potentials of compounds **11**, referenced against ferroecene.

Compound	$E_{1/2,ox1}$ / V	$E_{1/2,red1}$ / V	$E_{1/2,red2}$ / V
**11-Mo**	0.30	-0.93	-2.22
**11-W**	0.01	-1.39	-2.50

6. Crystallographic details

Table S 2: Crystallographic details of complexes

	**3-W'**	**3-Mo**	**3-W**	**4-Mo**	**5-Mo**	**5-W**	**6a-Mo**	**6b-Mo**	**7a**
Chemical Formula	$C_{34}H_{52}N_3O_4Si_1Cl_1W_1$	$C_{31}H_{43}N_3O_3Cl_2Mo_1$ 0.825 C_6H_6	$C_{31}H_{43}N_3O_3Cl_2W_1$	$C_{35}H_{52}N_4O_2Cl_2Mo_1$ C_6H_6	4($C_{45}H_{63}N_3O_5Mo_1$) $C_4H_{10}O_1$	2($C_{45}H_{63}N_3O_5W_1$) $C_4H_8O_1$, 3 C_5H_{12}	$C_{31}H_{43}N_3O_3Cl_1Mo_1$ 0.8 $C_4H_{10}O_1$	2 ($C_{39}H_{62}N_4O_3Cl_1Mo_1$) C_5H_{12}	$C_{41}H_{56}N_3O_3Mo_1$ 0.4 CH_2Cl_2
M_r (g mol^{-1})	814.17	736.96	760.43	805.75	3361.80	2108.20	696.37	1604.77	768.80
Crystal System	Triclinic	Monoclinic	Orthorhombic	Triclinic	Monoclinic	Triclinic	Orthorhombic	Monoclinic	Monoclinic
Space Group	*P*-1	$P2_1/c$	$P2_12_12_1$	*P*-1	$P2_1/c$	*P*-1	*Cmce*	$P2_1/n$	$P2_1/c$
a (Å)	9.0409(4)	31.121(2)	9.2952(7)	10.954(5)	18.8433(7)	14.5252(16)	32.3736(16)	10.3659(6)	8.8132(12)
b (Å)	13.4837(7)	12.1236(8)	11.9544(10)	13.601(6)	17.9411(8)	15.4379(14)	9.7802(6)	15.2234(8)	32.450(4)
c (Å)	15.6817(9)	19.5565(12)	29.678(3)	28.987(12)	28.8870(13)	28.374(3)	21.3275(11)	28.4220(16)	15.0330(16)
α (°)	97.982(2)	90	90	99.616(10)	90	91.116(3)	90	90	90
β (°)	95.886(2)	92.075(2)	90	92.433(10)	103.9710(10)	99.518(4)	90	100.338(2)	101.104(4)
γ (°)	103.519(2)	90	90	100.090(10)	90	117.472(3)	90	90	90
V (Å^3)	1822.51(16)	7373.8(8)	3297.8(5)	4181(3)	9476.9(7)	5533.9(10)	6752.7(6)	4412.3(4)	4218.8(9)
Z	2	4	4	4	2	2	8	2	4
Density (g cm^{-3})	1.484	1.328	1.532	1.280	1.178	1.265	1.370	1.208	1.210
F(000)	828	3077	1528	1696	3572	2204	2933	1712	1623
Radiation Type	MoKα	MoKα	MoKα	MoKα	MoKα	MoKα	MoKα	MoKα	MoKα
μ (mm^{-1})	3.314	0.538	3.699	0.479	0.321	2.134	0.508	0.396	0.399
Crystal Size (mm)	0.18x0.17x0.14	0.20x0.19x0.02	0.03x0.02x0.01	0.08x0.01x0.01	0.15x0.14x0.10	0.05x0.04x0.02	0.15x0.11x0.08	0.34x0.12x0.06	0.10x0.08x0.07
Meas. Refl.	92397	128885	35385	98761	180371	237891	73235	122760	34041
Indep. Refl.	8442	13016	6745	15387	16676	19695	3097	8128	7447
Obsvd. [$I > 2\sigma(I)$]	7720	9230	4848	10394	10499	14649	2408	6950	3953
R_{int}	0.0602	0.1137	0.1380	0.0957	0.1350	0.1040	0.1228	0.0610	0.1767
R [$F^2 > 2\sigma(F^2)$]	0.0247	0.0755	0.0514	0.0502	0.0596	0.0421	0.0460	0.0515	0.0757
wR(F^2)	0.0510	0.1524	0.1051	0.1134	0.1933	0.1166	0.1319	0.1385	0.2149
S	1.097	1.154	0.978	1.034	1.023	1.035	1.080	1.173	1.021
$\Delta\rho_{max}$	1.436	0.761	0809	0.895	0.814	1.626	0.566	0.705	0.575
$\Delta\rho_{min}$	-1.059	-2.058	-1.264	-0.552	-0.693	-2.070	-0.825	-0.482	-0.689
CCDC	2500040	2500041	2500032	2500042	2500036	2500039	2500043	2502071	2500030

	7b	7c	8-Mo	9-Mo	9-W	11-Mo	11-W	12-Mo	12-W
Chemical Formula	$C_{43}H_{60}N_3O_4Mo_1$	$C_{45}H_{57}N_4O_3Mo_1$	$C_{31}H_{43}N_3O_2Cl_3Mo_1$	2 [$C_{34}H_{52}N_3O_2P_1Cl_2Mo_1$]	$C_{34}H_{52}N_3O_2P_1Cl_2W_1$	$C_{37}H_{61}N_3O_2P_2Cl_1Mo_1$	$C_{37}H_{61}N_3O_2P_2Cl_1W_1$	$C_{37}H_{61}N_3O_2P_2Cl_1Mo_1$	$C_{37}H_{61}N_3O_2P_2Cl_1W_1$
	0.25 C_6H_{14}	$C_1H_2Cl_2$	3 CH_2Cl_2	2.5 $C_4H_8O_1$ $C_{1.4}H_{2.8}O_{0.3}$	0.7 $C_4H_{10}O_1$	$C_{32}H_{12}F_{24}B_1$	$C_{32}H_{12}F_{24}B_1$		
M_r (g mol^{-1})	800.42	882.81	946.75	1669.88	872.418	1636.44	1724.35	773.21	861.12
Crystal System	Triclinic	Triclinic	Monoclinic	Monoclinic	Orthorhombic	Monoclinic	Monoclinic	Monoclinic	Monoclinic
Space Group	*P*-1	*P*-1	$P2_1/c$	$P2_1/c$	*Pbca*	$P2_1/n$	$P2_1/n$	$P2_1/c$	$P2_1/c$
a (Å)	15.0562(6)	12.1968(10)	9.8980(7)	15.455(5)	8.0069(3)	12.9262(5)	12.8840(4)	13.900(2)	13.9202(11)
b (Å)	16.5543(6)	13.6003(13)	14.0852(8)	30.653(11)	28.4469(14)	29.4136(13)	29.4322(11)	16.924(3)	17.0250(13)
c (Å)	19.7946(7)	15.9732(14)	30.683(2)	18.562(6)	34.7626(18)	20.8378(8)	20.8526(9)	18.240(3)	18.2858(14)
α (°)	100.8630(10)	106.340(3)	90	90	90	90	90	90	90
β (°)	93.916(2)	109.774(3)	90.609(2)	96.839(11)	90	104.7570(10)	104.7170(10)	96.708(6)	97.150(3)
γ (°)	114.5780(10)	103.751(4)	90	90	90	90	90	90	90
V (Å^3)	4346.5(3)	2224.2(3)	4277.5(5)	8731(5)	7917.9(6)	7661.3(5)	7648.0(5)	4261.5(12)	4299.9(6)
Z	4	2	4	4	8	4	4	4	4
Density (g cm^{-3})	1.223	1.318	1.470	1.270	1.464	1.419	1.498	1.205	1.330
F(000)	1702	926	1940	3526	3564.988	3336	3464	1636	1764
Radiation Type	MoKα	MoKα	MoKα	MoKα	MoKα	MoKα	MoKα	MoKα	MoKα
μ (mm^{-1})	0.344	0.458	0.902	0.498	3.133	0.350	1.690	0.477	2.855
Crystal Size (mm)	0.19x0.18x0.14	0.25x0.24x0.23	0.35x0.02x0.01	0.73x0.07x0.06	0.20x0.18x0.02	0.25x0.23x0.15	0.15x0.14x0.11	0.25x0.24x0.20	0.25x0.10x0.01
Meas. Refl.	187366	65057	82422	177198	159160	194944	163150	28287	58507
Indep. Refl.	19946	10201	7598	21492	7248	15641	15664	7544	7596
Obsvd. $[I > 2\sigma(I)]$	15670	8985	6502	16358	6461	12864	13610	4434	5944
R_{int}	0.0826	0.0699	0.0576	0.0683	0.0635	0.1011	0.0588	0.0970	0.1028
R [$F^2 > 2\sigma(F^2)$]	0.0350	0.0355	0.0619	0.0437	0.0546	0.0608	0.0316	0.1003	0.0542
wR(F^2)	0.0880	0.0909	0.1632	0.1215	0.1381	0.1490	0.0775	0.2620	0.1397
S	1.037	1.037	1.174	1.028	0.9605	1.136	1.122	1.072	1.068
$\Delta\rho_{max}$	0.818	0.797	0.731	1.115	3.1410	1.120	0.846	1.829	3.602
$\Delta\rho_{min}$	-0.448	-0.843	-0.788	-0.727	-1.9132	-0.416	-0.661	-1.499	-1.240
CCDC	2500074	2500073	2500033	2500034	2500038	2500037	2500035	2500029	2500031

Table S 3: Selected bond lengths and angles

	3-W'	3-Mo	3-W	4-Mo	5-Mo	5-W	6a-Mo	6b-Mo	7a	7b	7c	8-Mo
M1-C1	2.163(2)	2.124(6)	2.126(12)	2.122(4)	2.150(5)	2.154(6)	2.103(4)	2.097(4)	2.128(6)	2.1179(19)	2.110(2)	2.118(6)
M1-O10/N40/Cl3	1.691(3)	1.680(5)	1.691(8)	1.711(3)	1.695(4)	1.715(3)	1.657(5)	1.710(3)	1.684(6)	1.6683(15)	1.6729(15)	2.3770(16)
M1-Cl1/C40/O40/N40	2.5774(8)	2.3917(18)	2.347(3)	2.4073(13)	-	-	2.379(3)	2.4262(11)	2.175(6)	1.9518(14)	2.0490(16)	2.3953(15)
M1-Cl2	-	2.5061(19)	2.530(4)	2.5167(13)	-	-	-	-	-	-	-	2.3872(16)
M1-O1	1.8862(18)	1.873(4)	1.859(7)	1.919(3)	1.911(4)	1.918(3)	1.952(2)	1.997(2)	1.964(5)	1.9631(15)	1.9955(13)	1.840(4)
M1-O2	1.9270(18)	1.882(5)	1.866(8)	1.910(3)	1.923(4)	1.920(3)	-	12.007(3)	1.970(5)	1.9816(14)	1.9746(14)	1.849(4)
M1-O40	1.8695(19)	-	-	-	1.978(3)	1.943(3)	-	-	-	-	-	-
M1-O41	-	-	-	-	2.063(4)	2.058(4)	-	-	-	-	-	-
M1-P40	-	-	-	-	-	-	-	-	-	-	-	-
M1-P50	-	-	-	-	-	-	-	-	-	-	-	-
O1-M1-O2	156.32(8)	157.77(19)	158.2(3)	159.99(11)	158.85(17)	158.04(16)	139.03(14)	154.38(11)	155.1(2)	142.79(6)	144.58(6)	159.79(18)
C1-M1-Cl1/O40/O10	165.57(10)	165.97(18)	164.2(3)	167.66(10)	163.28(17)	156.48(16)	149.92(15)	160.93(12)	129.1(3)	139.61(7)	137.91(7)	178.14(16)
X1/O10-Mo1-X1/X2*	172.8(3)	174.03(17)	177.4(3)	177.17(11)	164.19(16)	169.29(16)	-	-	-	-		178.45(6)
P40-M1-P50	-	-	-	-	-	-	-	-	-	-		-
τ_5	-	-	-	-	-	-	0.18	-	0.43	0.05	0.11	-

*X1 = Cl2, O10, N40, O41; X2 = Cl2, Cl3; ** angle is symmetry generated

	9-Mo	9-W	11-Mo	11-W	12-Mo	12-W
M1-C1	2.112(2)	2.094(8)	2.090(3)	2.087(3)	2.099(10)	2.067(8)
M1-O10/N40/Cl3	-	-	-	-	-	-
M1-Cl1	2.4422(9)	2.437(2)	2.4363(9)	2.4298(7)	2.459(3)	2.463(2)
M1-Cl2	2.4674(9)	2.468(2)	-	-	-	-
M1-O1	1.8942(19)	1.885(6)	1.843(2)	1.850(2)	1.931(7)	1.938(5)
M1-O2	1.9095(19)	1.868(5)	1.869(2)	1.876(2)	1.949(7)	1.948(5)
M1-O40	-	-	-	-	-	-
M1-O41	-	-	-	-	-	-
M1-P40	2.5074(10)	2.45(2)	2.5357(10)	2.5319(8)	2.480(3)	2.475(2)
M1-P50	-	-	2.5438(10)	2.5229(8)	2.485(3)	2.470(2)
O1-M1-O2	161.49(8)	164.6(2)	162.88(10)	163.87(10)	162.8(3)	164.7(2)
C1-M1-Cl1/O40/O10	176.69(3)	178.2(2)	177.52(10)	177.77(9)	177.3(3)	177.7(2)
X1/O10-Mo1-X2*	-	-	-	-	-	-
P40-M1-P50/Cl2	176.69(3)	176.4(4)	176.50(3)	176.78(3)	176.95(9)	176.96(7)
τ_5		-	-	-	-	-

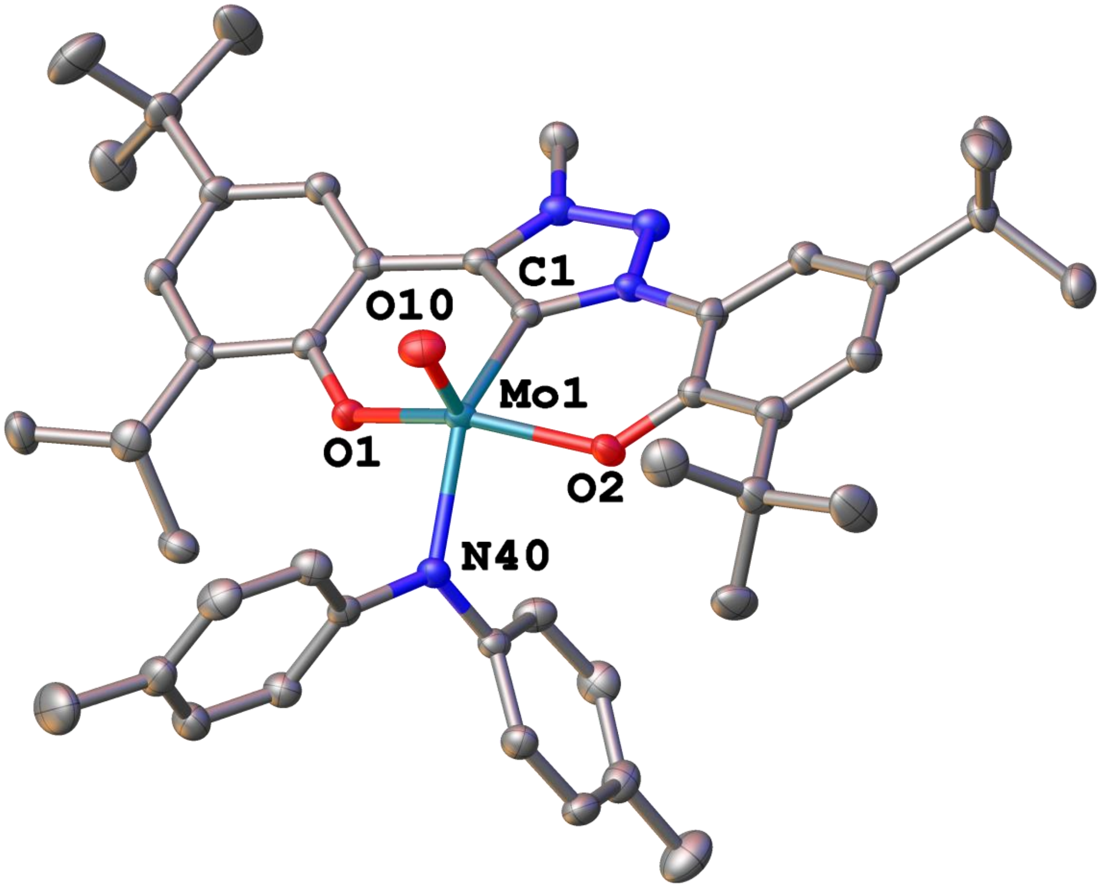

Figure S 108: Molecular structure of the anilide complex **7c**. Hydrogen atoms and lattice solvent molecules are omitted for clarity. Ellipsoids are shown at a probability level of 50%.

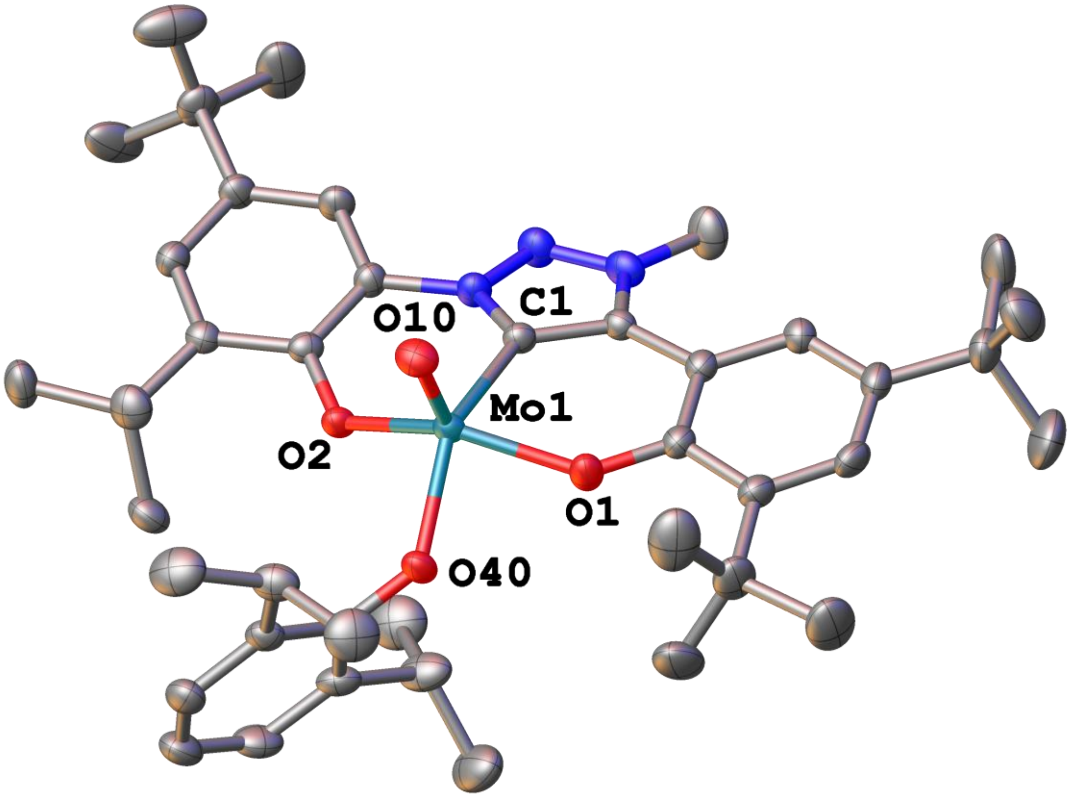

Figure S 109: Molecular structure of the anilide complex **7b**. Hydrogen atoms and lattice solvent molecules are omitted for clarity. Ellipsoids are shown at a probability level of 50%.
